# Supplementary material for: Identification of mental health and quality of life outcomes in primary care databases in the UK: a systematic review
Source: BMJ Open. 2019 Jul 2;9(7):e029227. doi: 10.1136/bmjopen-2019-029227 (PMC6609128; doi:10.1136/bmjopen-2019-029227)
Supplement: Supplementary data [file bmjopen-2019-029227supp001.pdf]

Identification of mental disorders and quality of life outcomes in primary care databases in the United Kingdom: systematic review protocol

Introduction

The number of studies conducted using electronic health records data largely increased in the last couple of decades. While these data sources are readily available and allow for studies to be conducted quicker that what it would be otherwise be possible with prospective designs, this also raises questions about variables definitions using data that are collected with a primary purpose that is not research.

Objective

To systematically review and summarise the strategies used to define such outcomes in previous studies, and the extent to which case definitions were validated.

Methods

Information sources

The following databases will be searched to identify potentially eligible studies:

- MEDLINE, via Ovid
- Embase, via Ovid
- CPRD, THIN and QResearch publications databases
- Reference lists of the eligible articles

Search strategy

- Electronic databases: search expressions provided at the end of this document.
- CPRD list of publications: hand screening.
- Backwards citation tracking: hand screening.

Outcomes of interest

- Anxiety
- Depression
- Fatigue
- Dementia
- Mild cognitive impairment
- Pain
- Female sexual dysfunction
- Sleep disorders
- Self-harm
- Suicide
- Composite outcomes of one or more of the above.

Inclusion criteria

- Studies in which patients were selected from primary health care databases in the UK
- Studies where the outcome was one of the outcomes of interest for this review

Exclusion criteria

- Conference abstracts, study protocols, or review articles
- Studies providing data only on the treatment of an eligible mental health outcome

Selection process

The references will be screened in three consecutive phases by two researchers. In the first phase, the title and the abstract of each study will be read to determine their eligibility for the study by applied the pre-defined exclusion criteria. If the information provided in the title and abstract does not allow the unequivocal exclusion of the study, the full text will be considered. In the second phase, the full-text of each study considered eligible in the first phase will be obtained and read, in order to determine the eligibility considering all the information in the paper. If the list of codes is provided, the paper will be included in the review. Studies not providing the list of codes will be assessed in a third phase, where the corresponding author of the papers will be contacted by email and invited to provide the list of the search terms involved.

Data extraction

The following data will be abstracted from the original reports, if available:

- Study design
- Adverse mental health outcome studied
- Database applied
- Use of linked data, and if yes, to which databases
- Type of codes used to define the condition (Read, ICD, OXMIS, etc.)
- Handling of previous disease episodes
- Availability of list of codes
- Medications used to define the conditions
- List of codes used to define the specific mental health outcome in the database
- Type of codes includes (symptoms, diagnoses, referrals, etc.)
- Validation of the list of codes used
- Results from the validation

Study records

All records will be imported into EndNote X7. Duplicates will be manually removed.

A backup of the search expression applied and the records obtained from each database, as well as the date of last update and run, will be kept safe.

A record of excluded/included studies, with the respective motives, will be kept in Microsoft Office Excel.

Data synthesis

Descriptive tables will be produced for each mental health outcome. The list of codes will be revised for sources of heterogeneity, and the results will be reported in text.

Search expressions

MEDLINE search expression, applied via OVID, 28 June 2018

1. CPRD.mp.
2. Clinical Practice Research.mp.
3. GPRD.mp.
4. General Practice Research Database.mp.
5. The Health Improvement Network.mp.
6. QRESEARCH.mp.
7. DIN-LINK.mp.
8. VAMP.mp.
9. Value Added Information Medical.mp.
10. (THIN adj1 (database or dataset or data)).mp.
11. (Read adj1 (term* or code# or codification)).mp.
12. (diagnostic adj1 (term* or code#)).mp.
13. Disease Analyzer.mp.
14. Primary care clinical informatics unit.mp.
15. PCCIU.mp.
16. (optimum patient care adj4 data*).mp. [mp=title, abstract, original title, name of substance word, subject heading word, floating sub-heading word, keyword heading word, protocol supplementary concept word, rare disease supplementary concept word, unique identifier, synonyms]
17. OPCRD.mp.
18. health information network.mp.
19. health improvement network.mp.
20. Q research.mp.
21. (ResearchOne or (Research One adj2 data*)).mp. [mp=title, abstract, original title, name of substance word, subject heading word, floating sub-heading word, keyword heading word, protocol supplementary concept word, rare disease supplementary concept word, unique identifier, synonyms]
22. Doctors Independent Network.mp.
23. SAIL.mp.
24. (SAIL adj4 data*).mp. [mp=title, abstract, original title, name of substance word, subject heading word, floating sub-heading word, keyword heading word, protocol supplementary concept word, rare disease supplementary concept word, unique identifier, synonyms]
25. mediplus.mp.
26. ((general practice or primary care or primary health care) adj4 data*).mp.
27. longitudinal patient database.mp.
28. ((EHR or eletronic health record*) adj4 data*).mp. [mp=title, abstract, original title, name of substance word, subject heading word, floating sub-heading word, keyword heading word, protocol supplementary concept word, rare disease supplementary concept word, unique identifier, synonyms]
29. health care database*.mp.
30. exp "mental disorders"/
31. exp "behavior and behavior mechanisms"/
32. exp "behavioral disciplines and activities"/
33. exp Psychological Phenomena/
34. exp fatigue/
35. exp pain/
36. exp "Sleep Wake Disorders"/
37. exp central nervous system depressants/

38. exp muscle relaxants, central/
39. exp psychotropic drugs/
40. exp sleep aids, pharmaceutical/
41. (anxiety or anxious* or panic or anxiolytic* or (stress not oxidat*) or depressi* or dysthymia or antidepress* or sexual or erectile or suicid* or self-harm or hopeless* or sleep or insomnia or hypnotic* or cognit* or chemo-fog or chemo-brain or pain or fatigue or (mental adj1 (disorder or disorders)) or antipsychotic).mp.
42. 30 or 31 or 32 or 33 or 34 or 35 or 36 or 37 or 38 or 39 or 40 or 41
43. exp United Kingdom/
44. (UK or Britain or British or England or English or Scotland or Scottish or Wales or Welsh Ireland).mp.
45. 43 or 44
46. or/1-29
47. 42 and 45 and 46

EMBASE search expression, applied via OVID, 28 June 2018

1. CPRD.mp.
2. Clinical Practice Research.mp.
3. GPRD.mp.
4. General Practice Research Database.mp.
5. The Health Improvement Network.mp.
6. QRESEARCH.mp.
7. DIN-LINK.mp.
8. VAMP.mp.
9. Value Added Information Medical.mp.
10. (THIN adj1 (database or dataset or data)).mp.
11. (Read adj1 (term* or code# or codification)).mp.
12. (diagnostic adj1 (term* or code#)).mp.
13. Disease Analyzer.mp.
14. Primary care clinical informatics unit.mp.
15. PCCIU.mp.
16. (optimum patient care adj4 data*).mp.
17. OPCRD.mp.
18. health information network.mp.
19. health improvement network.mp.
20. Q research.mp.
21. (ResearchOne or (Research One adj2 data*)).mp.
22. Doctors Independent Network.mp.
23. SAIL.mp.
24. (SAIL adj4 data*).mp.
25. mediplus.mp.
26. ((general practice or primary care or primary health care) adj4 data*).mp.
27. longitudinal patient database.mp.
28. ((EHR or eletronic health record*) adj4 data*).mp.
29. health care database*.mp.
30. or/1-29
31. UK.mp.
32. United Kingdom.mp.
33. England.mp.
34. Wales.mp.
35. Scotland.mp.
36. Northern Ireland.mp.

37. 31 or 32 or 33 or 34 or 35 or 36
38. exp central depressant agent/
39. exp central muscle relaxant/
40. exp psychotropic agent/
41. antidepress*.mp.
42. antipsychotic.mp.
43. anxiolytic.mp.
44. exp mental capacity/ or exp mental compliance/ or exp mental concentration/ or exp mental deficiency/ or exp mental deterioration/ or exp mental development/ or exp mental development assessment/ or exp mental disease/ or exp mental disease assessment/ or exp mental dissociation/ or mental function/ or exp mental health/ or exp mental health care/ or exp mental health center/ or exp mental health organization/ or exp mental health research/ or exp mental health service/ or exp mental hospital/ or exp mental stress/
45. depressi*.mp.
46. dysthymia.mp.
47. catatonia.mp.
48. self-injur*.mp.
49. self injury.mp.
50. self mutilation.mp.
51. suicid*.mp.
52. self-harm.mp.
53. anxious*.mp.
54. anxiety.mp.
55. panic.mp.
56. catastrophi*.mp.
57. phobia.mp.
58. phobic.mp.
59. neurotic.mp.
60. compulsive.mp.
61. bipolar.mp.
62. neurotic.mp.
63. personality.mp.
64. psychotic.mp.
65. psychosis.mp.
66. paranoid.mp.
67. delusional.mp.
68. sexual.mp.
69. insomnia.mp.
70. exp insomnia/
71. exp sleep/ or exp sleep disorder/
72. exp somatoform disorder/
73. exp substance abuse/ or exp "substance use"/
74. exp stress/
75. or/38-74
76. 30 and 37 and 75

Changes from the protocol with justification

The protocol specified that only female sexual dysfunction was of interest because this review is part of a PhD on women with a history of breast cancer. During the process of conducting the review, however, we decided to drop the gender restriction, as the review would be of more general use as a standalone publication.
