# Supplementary material for: Identification of mental health and quality of life outcomes in primary care databases in the UK: a systematic review
Source: BMJ Open. 2019 Jul 2;9(7):e029227. doi: 10.1136/bmjopen-2019-029227 (PMC6609128; doi:10.1136/bmjopen-2019-029227)
Supplement: Supplementary data [file bmjopen-2019-029227supp002.pdf]

Appendix 2. Template email sent to corresponding authors of the studies for which a list of codes wasn't provided in the publication.

Dear [corresponding author],

I'm currently doing a PhD about mental health and quality of life in breast cancer survivors. As part of my PhD, I'm conducting a systematic review of the studies that assessed mental health outcomes using electronic health records. The aim of the review is to summarise how studies have identified these outcomes in primary care databases in the UK; the review will be part of my PhD thesis and we are also planning to publish it in a peer-reviewed journal in due course.

I'm writing to you because a study in which you are the corresponding author was identified as eligible (please see title below), and I would kindly ask if you could be of assistance with the issues described below.

[title]

[description of the list of codes needed]

I look forward to hearing from you and thank you in advance for your help.

Best wishes,

Helena
