# Supplementary material for: Identification of mental health and quality of life outcomes in primary care databases in the UK: a systematic review
Source: BMJ Open. 2019 Jul 2;9(7):e029227. doi: 10.1136/bmjopen-2019-029227 (PMC6609128; doi:10.1136/bmjopen-2019-029227)
Supplement: Supplementary data [file bmjopen-2019-029227supp003.pdf]

Table 1. Main characteristics of the eligible studies: anxiety.

Author, year of publication	Study title	Type of study	Database(s)	Study period (years)	Outcome description	List of codes available	Validation of list of codes	EHR-related eligibility criteria for the study // Follow up requirements	Handling of outcomes occurring prior to exposure	Notes
Bouras, 2016 [1]	Linked and primary care database analysis of the incidence and impact of psychiatric morbidity following gastrointestinal cancer surgery in England	Cohort study	CPRD, HES	1997-2012	"Diagnosis code [for anxiety] in CPRD or HES, or a prescription code [for Diazepam or Lorazepam] between 36 months before and 12 months after surgery."	Yes	None stated	Data available for >3 years before the index date // Follow up duration: 1 year after index date	Study quantified the psychiatric morbidity before and after the index date.	-
Fardet, 2012 [2]	Suicidal behavior and severe neuropsychiatric disorders following glucocorticoid therapy in primary care	Cohort study	THIN	1990-2008	Read and Multilex list of codes for diagnoses of panic disorder or panic attack <u>excluding</u> c odes for anxiety	No	None stated	≥6 months of registration with the primary care practice	Hazards ratios adjusted for past history of neuropsychiatric disorders (yes/no)	Outcome was eligible if there was no record of the outcome in the previous 6 months
Granerod, 2016 [3]	Increased rates of sequelae post-encephalitis in individuals attending primary care practices in the United Kingdom: a population-based retrospective cohort study	Cohort study	CPRD	1998-2012	"Anxiety disorders (including both symptom codes and diagnoses such as generalised anxiety disorder, panic disorder, post-traumatic stress disorder and obsessive compulsive disorder)"	Yes	None stated	None stated // At least one contact with the GP practice in the two years after the index date	Analysis restricted to those at risk of a new-onset outcome, defined as no code in the year prior to the index date.	-
Hesdorffer, 2012 [4]	Epilepsy, suicidality, and psychiatric disorders: a bidirectional association	Matched cohort study	CPRD	1990-2008	Anxiety, not further specified	No	None stated	At least 3 years of data before the index date, 1 year after the index date, and at least 1 code for a medical or drug code in the 6 months before the index // Follow up 3 years after index date	Excluded subjects with a record of the outcome before the study date.	-

Author, year of publication	Study title	Type of study	Database(s)	Study period (years)	Outcome description	List of codes available	Validation of list of codes	EHR-related eligibility criteria for the study // Follow up requirements	Handling of outcomes occurring prior to exposure	Notes
Khan, 2010 [5]	Consulting and prescribing behaviour for anxiety and depression in long-term survivors of cancer in the UK	Cohort study	CPRD	2003-2006	Consultations for anxiety and prescriptions of benzodiazepines and Buspirone	No	None stated	None stated	History of anxiety prior to the analysis period included in the models	Outcomes occurred within a 3-year period chosen for the study
Kurd, 2005 [6]	The risk of depression, anxiety, and suicidality in patients with psoriasis: A population-based cohort study	Cohort study	CPRD	1987-2002	“clinician diagnosis of anxiety and related disorders in which anxiety symptoms are common”	No<br><br>(refers using the same codes of previous studies)	None stated	None stated	Patients with history of anxiety in the 6 months before the index date were excluded in a sensitivity analysis.	-
Lurie, 2015 [7]	Antibiotic exposure and the risk for depression, anxiety, or psychosis: A nested case-control study	Nested case control study	THIN	1995-2013	Anxiety, including codes for diagnosis of generalised anxiety disorder and phobic anxiety	Yes	None stated	Only records from patients that had been registered with the GP for more than 183 days<br>//<br>Outcomes were considered incident if occurring at more than 183 days after the index date.	Patients who had pharmacological treatment for a specific psychiatric diagnosis more than 90 days before the diagnosis was first recorded were excluded.	Patients with mixed anxiety and depression were excluded from the analysis
Martin-Merino, 2010 [8]	Prevalence, incidence, morbidity and treatment patterns in a cohort of patients diagnosed with anxiety in UK primary care	Cohort study	THIN	2002-2004	“Identification codes included all Read codes describing anxiety. These included codes ranging from mild anxiousness symptoms to other disorders such as phobia, panic attack and generalized and mixed anxiety disorders”	Yes	Yes<br><br>Record review/ questionnaires	Enrolled for at least 2 years with the practice and have received one prescription in the previous year; patients aged ≥70 years had to have at least 2 visits registered in the follow up period of >1 year.	Patients with previous anxiety diagnoses were excluded, as well as those who had 5 or more prescriptions of anxiolytics before the diagnosis.	-

Author, year of publication	Study title	Type of study	Database(s)	Study period (years)	Outcome description	List of codes available	Validation of list of codes	EHR-related eligibility criteria for the study // Follow up requirements	Handling of outcomes occurring prior to exposure	Notes
Meier, 2004 [9]	The risk of severe depression, psychosis or panic attacks with prophylactic antimalarials	Cohort study	CPRD	1990-1999	First time diagnosis of panic attack, regardless of referral or treatment, identified by OXMIS- and/or- ICD-8-codes	No	Yes<br><br>'reviewed a list of all cases to determine inclusion/ exclusion'	≥1 year of data available before index date and "some GPRD activity (diagnoses or prescriptions) recorded after the index date"<br>//<br>Patients were censored 18 months after exposure date	"The base population for person-time analyses consisted of all subjects free of (…) panic attacks at the start of follow up."	-
Schneider, 2013 [10]	Antimalarial chemoprophylaxis and the risk of neuropsychiatric disorders	Nested case control study	CPRD	2001-2009	Incident diagnosis of anxiety, not further specified	No	None stated	At least 1 year of date before the index date<br>//<br>some activity (diagnoses or prescriptions) recorded after the index date	Excluded patients with the outcome of interest observed before the index date	-
Sheehan, 2015 [11]	Mental illness, challenging behaviour, and psychotropic drug prescribing in people with intellectual disability: UK population based cohort study	Cohort study	THIN	1999-2013	Anxiety, including codes for symptoms and diagnoses	Yes	None stated	Entry in the cohort at least one year after registration with the practice	None stated	Excludes cases of mixed anxiety and depression

Author, year of publication	Study title	Type of study	Database(s)	Study period (years)	Outcome description	List of codes available	Validation of list of codes	EHR-related eligibility criteria for the study // Follow up requirements	Handling of outcomes occurring prior to exposure	Notes
Walters, 2012 [12]	Recent trends in the incidence of anxiety diagnoses and symptoms in primary care	Cohort study	THIN	1998-2008	Anxiety, including symptoms, diagnosis, mixed anxiety and depression, panic attacks and panic disorder<br><br>Results were also provided separately for:<br>- anxiety disorders (eg. chronic anxiety, generalised anxiety disorder, anxiety state);<br>- anxiety symptoms (e.g. ‘anxiousness’);<br>- mixed anxiety and depression;<br>- panic attacks and panic disorder.	No	None stated	≥1 year of data since registration with the practice and ‘consistent recording of at least one medical record (e.g. a diagnostic entry), one additional health data record (e.g. blood test result) and >1 prescriptions on average for the practice per patient per year and consistent reporting of mortality.’	Excluded patients with an entry for anxiety recorded in the previous year.	Participants could have had more than one episode during the follow up, provided that they were separated for more than 12 months.

CPRD – Clinical Practice Research Datalink; HES – Hospital Episodes Statistics; ICD-10 – International Classification of Diseases, edition 10; ND – not defined; OXMIS – Oxford Medical Information System; PCCIU – Primary Care Clinical Informatics Unit Research; THIN – The Health Improvement Network.

Table 2. Main characteristics of the eligible studies: depression.

Author, year of publication<br><br>Title	Study title	Type of study	Database(s)	Study period (years)	Outcome description	List of codes available	Validation of list of codes	EHR-related eligibility criteria for the study<br>//<br>Follow up requirements	Handling of outcomes occurring prior to exposure	Notes
Becker, 2011 [13]	Risk of incident depression in patients with Parkinson disease in the UK	Nested case-control study	CPRD	1994-2005	'To be included in the analysis as a valid depression case, a patient had to have a code recorded for an affective disorder (depression, manic disorders, bipolar disorders, or unspecified affective disorders) during follow-up.'	Yes	Yes<br><br>Pharmacological treatment with antidepressive drugs	At least 3 years of computer EHR prior to the index date<br>//<br>None stated.	Cases who had depression diagnosed prior to the index date were excluded;	-
Booth, 2015 [14]	Impact of bariatric surgery on clinical depression. Interrupted time series study with matched controls	Controlled interrupted time-series study	CPRD	2000-2012	Clinical depression was identified through medical diagnoses for depression recorded in clinical or referral records as well as through prescriptions for anti-depressant drugs.	Yes	None stated	At least one year of registration with the practice prior to the index date	Not applicable	-
Bornand, 2016 [15]	The risk of new onset depression in association with influenza - A population-based observational study	Nested case-control study	CPRD	2000-2013	Minimum of three prescriptions for one or more antidepressant drugs recorded after the incident major depression diagnosis (i.e. the index date), identified by READ-codes based on ICD-10 codes (F32), if they started the antidepressant therapy within 90 days of the depression diagnosis	Yes	None stated	A minimum of three years of history before the index date.	Excluded patients with more than two prescriptions for antidepressants at any time prior to the index date.<br>Adjusted for history of affective disorders in the models.	Provides data for depression severity: general depression; mild depression; moderate depression; severe depression; other.

Author, year of publication<br><br>Title	Study title	Type of study	Database(s)	Study period (years)	Outcome description	List of codes available	Validation of list of codes	EHR-related eligibility criteria for the study<br>//<br>Follow up requirements	Handling of outcomes occurring prior to exposure	Notes
Bouras, 2016 [1]	Linked Hospital and Primary Care Database Analysis of the Incidence and Impact of Psychiatric Morbidity Following Gastrointestinal Cancer Surgery in England	Cohort study	CPRD<br>HES	1997-2012	"Codes for the diagnoses of (…) depression were measured in CPRD (…) In HES, ICD-10 codes recorded in the first position of a hospital episode (signifying the main condition treated) for the diagnoses of depression. (…) Prescription data (…) including antidepressants (Fluoxetine, Paroxetine, Sertraline, Citalopram, Escitalopram, Mitrazapine, and Venlafaxine), (…) anxiolytics (Diazepam and Lorazepam)."	Yes	None stated	Data available for >3 years before the study index date<br><br>//<br><br>Follow up duration: 1 year post diagnosis	Not applicable	-
Claxton, 2000 [16]	Selective serotonin reuptake inhibitor treatment in the UK: Risk of relapse or recurrence of depression	Cohort study	MediPlus	1993-1995	Re-initiation of any antidepressant after a gap of at least 6 months with no antidepressant prescription; suicide attempt, referral to psychotherapy or psychiatrist, admission to a mental health facility, emergency room use related to mental disorders, or electroconvulsive therapy and re-initiation of antidepressant one of the above.<br><br>Depression first defined as treatment with a SSRI and a Read code within 1 month of the prescription.	Yes	None stated	Only patients with contact with the services during the previous 2 years of the index date;<br>//<br>Follow up duration of 18 months post index date	Not applicable (all patients had been treated with SSRI)	Patients dementia, schizophrenia, psychosis and manic depression were excluded.
Clifford, 2002 [17]	Drug or symptom-induced depression in men treated with apha1-blockers for benign prostatic hyperplasia? A nested-case control study	Nested case-control study	CPRD	1992-1999	Proxy of antidepressant prescription (first prescription of antidepressant)	No	None stated	Registered in CPRD for at least 12 months<br>//<br>ND	None stated	-

Author, year of publication<br><br>Title	Study title	Type of study	Database(s)	Study period (years)	Outcome description	List of codes available	Validation of list of codes	EHR-related eligibility criteria for the study<br>//<br>Follow up requirements	Handling of outcomes occurring prior to exposure	Notes
Dave, 2010 [18]	Incidence of Maternal and Paternal Depression in Primary Care	Cohort study	THIN	1993-2007	“Read code entry for unipolar depression and/or a prescription for an antidepressant at the appropriate therapeutic dose for treatment of depression on a given consultation date (…) we eliminated those who had an entry for anxiety or panic disorder but had no entry for depression in their entire computerized medical record.”<br><br>New episode was considered when no diagnosis or prescription had been registered in the past year.	No<br><br>Available on request	None stated	None stated	Results stratified by history of previous mental disorder	Mixed anxiety and depression was included.
Fardet, 2012 [2]	Suicidal behavior and severe neuropsychiatric disorders following glucocorticoid therapy in primary care	Cohort study	THIN	1990-2008	‘Read code for unipolar depression, for symptoms of depression, or for a prescription for an antidepressant. Diagnoses were considered first; prescriptions of antidepressants were used in defining the outcome only when there was no recorded diagnosis of a neuropsychiatric illness and no other recorded indication for the prescription. To exclude patients who may have received prescriptions for antidepressants for anxiety rather than for depression, we eliminated those who had an entry for anxiety or panic disorder but had no entry for depression in their entire computerized medical record.’	No	None stated	≥6 months of registration with the primary care practice.	Hazards ratios adjusted for past history of neuropsychiatric disorders (yes/no)	Outcome was eligible if there was no record of the outcome in the previous 6 months

Author, year of publication<br><br>Title	Study title	Type of study	Database(s)	Study period (years)	Outcome description	List of codes available	Validation of list of codes	EHR-related eligibility criteria for the study<br>//<br>Follow up requirements	Handling of outcomes occurring prior to exposure	Notes
Gunnell, 2009 [19]	Varenicline and suicidal behaviour: a cohort study based on data from the General Practice Research Database	Cohort study	CPRD	2006-2008	Depression defined as the start of antidepressant therapy	No	None stated	At least 1 years of CPRD record before index date<br>//<br>ND	People who had been prescribed an antidepressant within the previous 6 months before index date were excluded. Previous psychiatric consultations considered in the models.	-
Granerod, 2016 [3]	Increased rates of sequelae post-encephalitis in individuals attending primary care practices in the United Kingdom: a population-based retrospective cohort study	Matched cohort study	CPRD	1998-2012	Depression, consisting of codes for depression diagnosis and symptoms if evidence of pharmacological treatment was present.	Yes	None stated	None stated<br>//<br>At least one contact with the GP practice in the two years after the index date.	Analysis restricted to those at risk of a new-onset outcome, defined as no code in the year prior to the index date.	-
Hagberg, 2017 [20]	Risk of Incident Antidepressant-Treated Depression Associated with Use of 5alpha-Reductase Inhibitors Compared with Use of alpha-Blockers in Men with Benign Prostatic Hyperplasia: A Population-Based Study Using the Clinical Practice Research Datalink	Nested case-control study	CPRD	1992-2013	A Read code for a depression diagnosis and a prescription for an antidepressant within 90 days of the depression diagnosis.	No	None stated	At least 1 year of recorded history in the database before cohort entry;	Excluded men with a diagnosis of depression or suicidal behaviors (ideation or attempts), or men who received prescriptions for antidepressant medications prior to cohort entry.	-

Author, year of publication<br><br>Title	Study title	Type of study	Database(s)	Study period (years)	Outcome description	List of codes available	Validation of list of codes	EHR-related eligibility criteria for the study<br>//<br>Follow up requirements	Handling of outcomes occurring prior to exposure	Notes
Hagberg, 2016 [21]	Incidence rates of suicidal behaviors and treated depression in patients with and without psoriatic arthritis using the Clinical Practice Research Datalink	Nested case control study	CPRD	1998-2012	'A patient was required to have at least one prescription for an antidepressant drug in addition to a diagnosis code for depression within 60 days of each other to qualify as a case of treated depression.'	No	Yes, record review	At least one year of registration with the practice prior to the index date	Any patient who had a diagnosis of depression or a prescription for an antidepressant drug recorded before the cohort entry date was excluded from the Treated Depression sub-cohort.	-
Harris, 2011 [22]	Depression indicators in a national sample of older community and care home patients: applying the Quality and Outcomes Framework	Cohort study	THIN	ND-2008	(i) depression case finding with assessment tool validated for primary care;<br>(ii) assessment of depression severity in patients with a new depression episode.<br><br>'Quality and Outcomes Framework Read Codes were used in both cases, but no account was taken of exceptions recorded by GPs, as these may bias comparisons between community and care home samples.'	Yes	None stated	Patients registered for at least 90 days with a new diagnosis of depression in period 91-450 days from end of follow up and no depression severity assessment >365 days before end of follow up.	Not applicable	Only practices contributing with data up to at least March 2008 were included.
Hesdorffer, 2012 [4]	Epilepsy, suicidality, and psychiatric disorders: a bidirectional association	Matched cohort study	CPRD	1990-2008	Incident major depression, not further specified	No	None stated	At least 3 years of data before the index date, 1 year after the index date, and at least 1 code for a medical or drug code in the 6 months before the index<br>//<br>Follow up 3 years after index date	Excluded subjects with a record of the outcome before the study date.	-

Author, year of publication<br><br>Title	Study title	Type of study	Database(s)	Study period (years)	Outcome description	List of codes available	Validation of list of codes	EHR-related eligibility criteria for the study<br>//<br>Follow up requirements	Handling of outcomes occurring prior to exposure	Notes
Jacob, 2017 [23]	Depression Risk in Patients with Rheumatoid Arthritis in the United Kingdom	Cohort study	Disease Analyser database	2000-2014	Diagnoses of depression, according to the ICD-10 codes	No	None stated	None stated<br>//<br>Incidence in the first 5 years of the index date	None stated	-
Jenkins-Jones, 2018 [24]	Poor compliance and increased mortality, depression and healthcare costs in patients with congenital adrenal hyperplasia	Matched cohort study	CPRD,<br>ONS,<br>HES inpatient and outpatient	?	Depression defined by Read Codes in CPRD, by ICD-10 code in HES inpatient data, or by the prescription of antidepressants.<br><br>Depression was identified not only from ongoing records but also from patients' clinical histories dating from before the start of data follow-up.<br><br>A sensitivity analysis considered only those depression outcomes identified by both diagnostic (Read or ICD-10) code and at least one antidepressant prescription.	Yes	None stated	None stated	Not applicable: the outcome was lifetime prevalence of depression rather than incident occurrence.	-

Author, year of publication<br><br>Title	Study title	Type of study	Database(s)	Study period (years)	Outcome description	List of codes available	Validation of list of codes	EHR-related eligibility criteria for the study<br>//<br>Follow up requirements	Handling of outcomes occurring prior to exposure	Notes
John, 2016 [25]	Recent trends in primary-care antidepressant prescribing to children and young people: an e-cohort study	Cohort study	SAIL	2003-2013	Incident episode: a Read code for a diagnosis or symptom of depression, or antidepressant, with no record of the given subtype (antidepressant prescription or depression diagnosis or depression symptom) in the previous 12 months; Participants could have more than one episode recorded across the study period as long a period of at least 12 months existed between entries within that subtype.<br><br>Prevalent episode: any record of the given subtype (antidepressant prescription or depression diagnosis or depression symptom) in a target year<br><br>Annual recurrent episode: defined as the first record in a given year of a given subtype where a record of that subtype exists previously in that individuals GP record.	Yes	None stated	Registered with the GP practice for at least 1 year	Not applicable	Explored the indication of the antidepressants

Author, year of publication<br><br>Title	Study title	Type of study	Database(s)	Study period (years)	Outcome description	List of codes available	Validation of list of codes	EHR-related eligibility criteria for the study<br>//<br>Follow up requirements	Handling of outcomes occurring prior to exposure	Notes
Kendrick, 2015 [26]	Changes in rates of recorded depression in English primary care 2003-2013: time trend analyses of effects of the economic recession, and the GP contract quality outcomes framework	Cohort study	CPRD	2003-2013	"had clinical or referral events recorded which included a Read code for non-psychotic depressive symptoms or diagnoses, or for assessment using depression symptom questionnaires."<br><br>Prevalence of depression: Read code present in the year or quarter;<br><br>Incidence of depression: limited to patients who had no code for depression recorded in the previous 12 months.<br><br>Incidence of first-ever depression: no previous code for depression diagnosis, symptoms or antidepressant treatment recorded within 10 year study period, and no previous record of depression or antidepressant treatment recorded.	Yes	None stated	None stated	Not applicable	"We excluded patients with psychotic diagnoses including bipolar disorder, psychotic depression, and schizoaffective psychosis, and patients prescribed antidepressants for other indications besides depression."
Khan, 2010 [5]	Consulting and prescribing behaviour for anxiety and depression in long-term survivors of cancer in the UK	Matched cohort study	CPRD	2003-2006	Consultations for depression and prescriptions of as tricyclics, SSRIs and MAOIs	No<br><br>(Available on request)	None stated	None stated<br><br>//<br><br>Follow up duration: 3 years	History of anxiety prior to the analysis period was included in the models	-
Kotz, 2017 [27]	Cardiovascular and neuropsychiatric risks of varenicline and bupropion in smokers with chronic obstructive pulmonary disease	Matched cohort study	QResearch	2001-2012	Depression, not further specified	No	None stated	Registered for >12 months before data extraction<br>//<br>Follow up duration 6 months	History of neuropsychiatric events before the index date were considered as confounders	-
Kotz, 2015 [28]	Cardiovascular and neuropsychiatric risks of varenicline: a retrospective cohort study	Matched cohort study	QResearch	2007-2012	Depression, not further specified	No	None stated	Registered for >12 months before data extraction<br>//<br>Follow up duration 6 months	History of neuropsychiatric events before the index date were considered as confounders	-

Author, year of publication<br><br>Title	Study title	Type of study	Database(s)	Study period (years)	Outcome description	List of codes available	Validation of list of codes	EHR-related eligibility criteria for the study<br>//<br>Follow up requirements	Handling of outcomes occurring prior to exposure	Notes
Kurd, 2005 [6]	The risk of depression, anxiety, and suicidality in patients with psoriasis: A population-based cohort study	Matched cohort study	CPRD	1987-2002	Depression included all clinician diagnoses of depressive symptomology including bipolar disorder, defined by diagnostic Read or OXMIS codes	No<br><br>(refers using the same codes of previous studies)	None stated	None stated	Patients with history of anxiety in the 6 months before the index date were excluded in a sensitivity analysis.	-
Lurie, 2015 [7]	Antibiotic exposure and the risk for depression, anxiety, or psychosis: A nested case-control study	Nested case control study	THIN	1995-2013	At least one Read code of depression; not further specified.	Yes	None stated	Only records from patients that had been registered with the GP for more than 183 days<br>//<br>Outcomes were considered incidence if occurring at more than 183 days after the index date.	Patients who had pharmacological treatment for a specific psychiatric diagnosis more than 90 days before the diagnosis was first recorded were excluded.	Patients with mixed anxiety and depression were excluded from the analysis
Martin-Merino, 2010 [29]	Study of a cohort of patients newly diagnosed with depression in general practice: Prevalence, incidence, comorbidity, and treatment patterns	Cohort study, descriptive and analytical	THIN	2002-2004	Patients with incident depression during the follow up but excluding those who had 5 or more prescriptions of an antidepressant before the depression diagnosis.	No	Yes<br><br>GP questionna ires	Registered for >2 years with their GP practice prior to the index date; Excluded patients aged >69 years who had fewer than 2 visits during the follow-up.	All subjects who had a record of depression before the index date were excluded.	Prevalence of depression calculated as the sum of all patients who had the outcome during the study, plus those who had the outcome in the two years before the study start date.

**Author, year of publication**<br><br>**Title**	**Study title**	**Type of study**	**Database(s)**	**Study period (years)**	**Outcome description**	**List of codes available**	**Validation of list of codes**	**EHR-related eligibility criteria for the study**<br>**//**<br>**Follow up requirements**	**Handling of outcomes occurring prior to exposure**	**Notes**
Meier, 2004 [9]	The Risk of Severe Depression, Psychosis or Panic Attacks with Prophylactic Antimalarials	Cohort study	CPRD	1990-1999	Depression referred to specialist or hospital, or if received treatment with antidepressants at or after the diagnosis date identified by OXMIS- and/or- ICD-8-codes	No	Yes<br><br>'reviewed a list of all cases to determine inclusion/ exclusion'	≥1 year of data available before index date and "some GPRD activity (diagnoses or prescriptions) recorded after the index date"<br>//<br>Patients were censored 18 months after exposure date	"The base population for person-time analyses consisted of all subjects free of depression at the start of follow up."	-
Millson, 2000 [30]	Are triptans with enhanced lipophilicity used for the acute treatment of migraine associated with an increased consulting rate for depressive illness?	Cohort study, analytical	CPRD, data for the West Midlands	1993-1997	'consulting at least once for depression'	Yes	No	None stated	None Stated	-
Milojevic, 2017 [31]	Mental health impacts of flooding: a controlled interrupted time series analysis of prescribing data in England	Cohort study	General practice prescribing data	2010-2015	Antidepressants prescription	No<br><br>(but list of antidepress ants provided in appendix)	Not applicable	None stated	Not applicable	-
Moore, 2009 [32]	Explaining the rise in antidepressant prescribing: a descriptive study using the general practice research database	Cohort study	CPRD	1993-2005	Depression: "first ever antidepressant prescription for depression diagnosed up to 180 days before or 90 days after the prescribing event, or received a first ever diagnosis of depression without an associated prescription for antidepressants."	No	None stated	Registered with practices contributing with up to standard quality data for the entire study period	Patients only included if it was the first ever events during the follow up period.	Provides data for treatment patterns: chronic treatment; intermittent treatment; short-term treatment; delayed treatment; no treatment.

Author, year of publication<br><br>Title	Study title	Type of study	Database(s)	Study period (years)	Outcome description	List of codes available	Validation of list of codes	EHR-related eligibility criteria for the study<br>//<br>Follow up requirements	Handling of outcomes occurring prior to exposure	Notes
Morgan, 2014 [33]	General practice-recorded depression and antidepressant use in young people with newly diagnosed Type 1 diabetes: a cohort study using the Clinical Practice Research Datalink	Matched cohort study	CPRD	1988-2010	Depression identified from diagnosis codes (Oxford Medical Information System and Read), along with at least one antidepressant prescription (monoamine oxidase inhibitors, selective serotonin reuptake inhibitors, tricyclic antidepressants or other antidepressants).	No	None stated	None stated	Participants with depression diagnoses or prescriptions prior to diabetes diagnosis (or prior to the start date for control subjects) were excluded.	-
Petersen, 2006 [34]	Risk and predictors of fatigue after infectious mononucleosis in a large primary-care cohort	Nested case control cohort study	CPRD	1989-2000	Depression, not further specified	No<br><br>(available from the authors)	None stated	At least one year of data before diagnosis<br>//<br>At least one year of complete follow up after diagnosis	Included patients who did not have fatigue in the year before onset	-
Rait, 2009 [35]	Recent trends in the incidence of recorded depression in primary care	Cohort study	THIN	1996-2006	"Diagnoses of depression (e.g. 'depressive disorder') and recorded depressive symptoms (e.g. 'low mood'). new episode of diagnosed depression was defined as an entry in the records where there was no previous diagnosis of depression coded in the previous year. A new episode of depressive symptoms was also defined as an entry where there had been no previous recorded depressive symptom code in the previous year."	No	None stated	At least one year of follow up data<br>//<br>ND	None stated	Provides results separately for diagnoses and symptoms of depression.
Shah, 2016 [36]	The mental health and mortality impact of death of a partner with dementia	Nested case control study	THIN	2005-2008	Depression, not further specified	Yes	None stated	At least one year of data with the practice	None stated	-

Author, year of publication<br><br>Title	Study title	Type of study	Database(s)	Study period (years)	Outcome description	List of codes available	Validation of list of codes	EHR-related eligibility criteria for the study<br>//<br>Follow up requirements	Handling of outcomes occurring prior to exposure	Notes
Schneider, 2013 [10]	Antimalarial chemoprophylaxis and the risk of neuropsychiatric disorders	Nested case control study	CPRD	2001-2009	Incident diagnosis of depression, not further specified	Yes	None stated	At least 1 year of date before the index date<br>//<br>some activity (diagnoses or prescriptions) recorded after the index date	Excluded patients with the outcome of interest observed before the index date	Excluded patients with history of cancer, alcoholism, and rheumatoid arthritis.
Schneider, 2010 [37]	COPD and the risk of depression	Cohort study	CPRD	1995-2005	Diagnosis of depression, not further specified<br><br>In sensitivity analysis, only cases with an incident diagnosis of depression who receive pharmacological treatment within 6 months of diagnosis were included	Yes	None stated	Excluded patients with less than 3 years of active recording history prior to the date of the COPD diagnosis	Patients with previous history of depression, suicide, suicidal ideation, etc., prior to the index date were excluded	Excluded patients with history of cancer, HIV, drug abuse, or alcoholism prior to the index date
Sheehan, 2015 [11]	Mental illness, challenging behaviour, and psychotropic drug prescribing in people with intellectual disability: UK population based cohort study	Cohort study, analytical	THIN	1999-2013	Read codes for depression (including mixed depression-anxiety) that the authors' used in previous studies.	Yes	None stated	Entry in the cohort at least one year after registration with the practice	None stated	Excludes cases of mixed anxiety and depression
Smeeth, 2008 [38]	Effect of statins on a wide range of health outcomes: A cohort study validated by comparison with randomized trials	Cohort study	THIN	1995-2006	Depression defined as the start of antidepressant pharmacotherapy	No	None stated	None stated	People with a previous history of the outcome prior to the index date were excluded from the analysis of that outcome.	First year of follow up was excluded.

Author, year of publication<br><br>Title	Study title	Type of study	Database(s)	Study period (years)	Outcome description	List of codes available	Validation of list of codes	EHR-related eligibility criteria for the study<br>//<br>Follow up requirements	Handling of outcomes occurring prior to exposure	Notes
Smith, 2014 [39]	Depression and multimorbidity: a cross-sectional study of 1,751,841 patients in primary care	Cross-sectional	PCCIU	ND-2007	"Read code for depression recorded within last year and/or 4 or more antidepressant prescriptions (excluding low-dose tricyclic antidepressants) within the last year. Low-dose tricyclic antidepressants were excluded because they are commonly prescribed for chronic pain syndromes rather than depression."	No	None stated	Patients alive and permanently registered with a general practice at the date of the study	None stated	-
Thomas, 2013 [40]	Smoking cessation treatment and risk of depression, suicide, and self harm in the Clinical Practice Research Datalink: prospective cohort study	Nested case-control study	CPRD<br>HES<br>ONS	2006-2011	"Incident episodes of depression as measured by the date that antidepressant treatment was initiated (treated depression)"<br><br>//<br><br>For comparison with previous study, depression was also defined with Read codes only in CPRD	Yes	None stated	At least one year of registration with the practice prior to the index date<br>//<br>ND	Previous psychiatric illness, and use of psychotropic medication considered as potential confounders.	-
Tyrer, 1999 [41]	A study of cardiovascular disease, depression and antidepressants on a computerised general practice database	Cohort study	UK MediPlus	1995-1996	'new diagnosis of depression and treatment, classified by the first antidepressant prescribed.'	No	None stated	At least 12 months of data before the index date<br>//<br>Follow up duration 12 months	Patients with depression in the 12 months period before the cardiac event were excluded.	-
Vallerand, 2018 [42]	Risk of depression among patients with acne in the U.K.: a population-based cohort study	Cohort study, analytical	THIN	1986-2012	Read code for major depressive disorder	Yes	None stated	None stated<br>//<br>Follow up for > 2 years after index date	Patients with MDD Read code prior to the start of follow up were excluded	-

Author, year of publication<br><br>Title	Study title	Type of study	Database(s)	Study period (years)	Outcome description	List of codes available	Validation of list of codes	EHR-related eligibility criteria for the study<br>//<br>Follow up requirements	Handling of outcomes occurring prior to exposure	Notes
Walters, 2011 [43]	The relationship between asthma and depression in primary care patients: A historical cohort and nested case control study	Cohort study and nested case control study	CPRD	1995-2006	GP recorded diagnosis of depression, as defined by Read/OXMIS codes) during the study period	No<br><br>(codes available from the authors)	None stated	All patients had at least 24 months of ‘up to standard’ data prior to the index date of the case.	All cases with a recorded medical diagnosis of depression or depressive symptoms before the index date were excluded from the cohort.	-
Yang, 2003 [44]	Lipid-lowering drugs and the risk of depression and suicidal behavior	Matched cohort study	CPRD	1991 onwards	Treated depression (with antidepressants) referred to a consultant or patient hospitalized for depression; excluded patients who also had diagnosis of anxiety or with specific causes for depression (e.g. post-partum depression)	No	Yes<br><br>List of patients referred to hospital or consultant were manually reviewed; referral letters reviewed.	≥1 year of data available before index date	Patients with history of depression prior to study start were excluded	-

CPRD – Clinical Practice Research Datalink; EHR – electronic health records; HES – Hospital Episodes Statistics; ICD-10 – International Classification of Diseases, edition 10; ND – not defined; PCCIU – Primary Care Clinical Informatics Unit Research; SAIL – SAIL Databank - The Secure Anonymised Information Linkage Databank; THIN – The Health Improvement Network; ONS – Office for National Statistics.

Table 3. Main characteristics of the eligible studies: composite outcomes, anxiety and depression.

Author, year of publication<br><br>Title	Study title	Type of study	Database(s)	Study period (years)	Outcome description	List of codes available	Validation of list of codes	EHR-related eligibility criteria for the study<br>//<br>Follow up requirements	Handling of outcomes occurring prior to exposure	Notes
John, 2016 [45]	Case-finding for common mental disorders of anxiety and depression in primary care: an external validation of routinely collected data	Validation study using patient reported outcomes	SAIL	2000-2009	Outcome: Common mental disorders, defined as anxiety and depression.<br><br>Read codes for: i) anxiety diagnosis, e.g. generalised anxiety disorder; ii) anxiety symptoms e.g. anxiousness; iii) mixed anxiety and depression; iv) panic attacks and panic disorders; v) depression diagnoses; vi) depression symptoms.<br><br>Treatment defined as having at least one prescription for an antidepressant, anxiolytic or hypnotic within the year around the date of the survey answer.<br><br>Excluded codes for other psychosis, phobias, obsessive compulsive disorders, post traumatic stress disorder, behavioural disorders, hyperkinetic disorders, conduct disorders, disorders of social functioning, and adjustment disorders.<br><br>Defined 12 algorithms with current and historical symptoms, diagnosis and treatment.	Yes	Yes<br><br>Patient reported outcomes	At least 6 months after registration with the practice	Not applicable (no exposure under study).<br><br>Incidence outcome were those where no previous entry had been recorded around 1 year of the date of the survey answer.<br><br>Other outcomes were considered historical.	-

Author, year of publication<br><br>Title	Study title	Type of study	Database(s)	Study period (years)	Outcome description	List of codes available	Validation of list of codes	EHR-related eligibility criteria for the study<br>//<br>Follow up requirements	Handling of outcomes occurring prior to exposure	Notes
Turner, 2016 [46]	Ongoing impairments following transient ischaemic attack: retrospective cohort study	Nested case control study	THIN	2009-2013	Outcome: Psychological impairment, defined as anxiety, depression and post-traumatic stress disorder.<br><br>First consultation after the index date with a Read code for symptoms or diagnosis of anxiety, depression or post-traumatic stress, plus a first prescription of an antianxiety or antidepressant drug	Yes	None stated	Practice with at least one year of up to standard data, and patients registered for at least one year with the practice.<br>//<br>Patient alive and registered with the practice 1 month after index	Not stated.	-

SAIL – SAIL Databank - The Secure Anonymised Information Linkage Databank; THIN – The Health Improvement Network.

Table 4. Main characteristics of the eligible studies: dementia.

Author, year of publication<br><br>Title	Study title	Type of study	Database(s)	Study period (years)	Outcome description	List of codes available	Validation of list of codes	EHR-related eligibility criteria for the study // Follow up requirements	Handling of outcomes occurring prior to exposure	Notes
Brown, 2016 [47]	Comparison of dementia recorded in routinely collected hospital admission data in England with dementia recorded in primary care	Validation study	CPRD<br>HES	1990-2012	Read codes for dementia and/or a code for a drug specifically prescribed for dementia (i.e. donepezil, galantamine, memantine and rivastigmine).	Yes	Yes<br><br>GP questionnaire	At least 12 months before and 12 months after the first HES record of dementia	Not applicable	-
Davies, 2014 [48]	Associations of anti-hypertensive treatments with Alzheimer's disease, vascular dementia, and other dementias	Nested case-control study	CPRD	1997-2008	Alzheimer's disease, vascular dementia, or unspecified/other dementias<br><br>Created four categories: probable Alzheimer's disease, possible Alzheimer's disease, vascular dementia, combine unspecified or other dementia.	Yes	No	None stated	Not applicable	-
Donegan, 2017 [49]	Trends in diagnosis and treatment for people with dementia in the UK from 2005 to 2015: a longitudinal retrospective cohort study	Cohort study	CPRD	2005-2015	Dementia defined using Read codes listed for the condition in the Quality and Outcomes Framework	No	No	Patients were eligible for analysis if registered with the practice for the entire quarter of the year being analysed;	Not applicable (descriptive study of the incident cases of dementia)	-
Dregan, 2015 [50]	Are Inflammation and Related Therapy Associated with All-Cause Dementia in a Primary Care Population?	Matched cohort study	CPRD	2002-2013	"Medical diagnostic codes were used to identify new diagnoses of dementia including Alzheimer's disease, vascular dementia, Lewy body dementia, frontotemporal dementia, dementia in other conditions, and unspecified dementia."	No	No	None stated	Considered incident cases only	-

Author, year of publication<br><br>Title	Study title	Type of study	Database(s)	Study period (years)	Outcome description	List of codes available	Validation of list of codes	EHR-related eligibility criteria for the study<br>//<br>Follow up requirements	Handling of outcomes occurring prior to exposure	Notes
Dregan, 2015 [51]	Patterns of anti-inflammatory drug use and risk of dementia: a matched case-control study	Matched case control study	CPRD	1992-2014	"Medical diagnostic codes were used to identify new diagnoses of dementia and include non-specific dementia (Eu02z), Alzheimer disease (F110), vascular dementia (Eu01), Lewy body dementia (Eu025), senile dementia (E00) and dementia in other conditions (Eu02)."	Yes	No	None stated	Considered incident diagnosis	-
Dunn, 2005 [52]	Does lithium therapy protect against the onset of dementia?	Nested case control study	CPRD	1992-2002	'Cases with definite diagnosis of Alzheimer disease, vascular dementia (with which there is a diagnostic overlap), and those with uncertain cause of dementia'	No	Yes<br><br>GP Questionn aire	"At least 4 years o research standard data preceding the date of the diagnosis"<br>//	Considered incident cases only	-
Dunn, 2005 [53]	Association between dementia and infectious disease: evidence from a case-control study	Nested case control study	CPRD	1992-2002	"We identified cases as all those patients with an incident dementia, as diagnosed by their GP, a GP colleague, or a hospital specialist (…) We included cases with a recorded diagnosis of Alzheimer disease, vascular dementia (with which there is diagnostic overlap), and those with uncertain cause of dementia. Other specified causes of dementia were excluded (eg, dementia in Parkinson's disease)."	No	Yes<br><br>GP questionna ires	"At least 4 years of research standard data preceding the date of first diagnosis (median time from onset of symptoms to diagnosis has been estimated at 4 years)."<br>//<br>ND	Considered incident cases only	Cases further classified as probable or possible cases of dementia.
Emdin, 2016 [54]	Blood Pressure and Risk of Vascular Dementia: Evidence From a Primary Care Registry and a Cohort Study of Transient Ischemic Attack and Stroke	Cohort study	CPRD<br>HES<br>ONS	1990-2003	First record of vascular dementia, in one of the databases. For the primary analysis, cases of Alzheimer's disease and vascular dementia (i.e. mixed dementia) were included.	Yes	No	Registered with the GP practice for at least one year<br>//<br>First 4 years of follow up excluded from analysis	First 4 years of follow up excluded from analysis	-

Author, year of publication<br><br>Title	Study title	Type of study	Database(s)	Study period (years)	Outcome description	List of codes available	Validation of list of codes	EHR-related eligibility criteria for the study<br>//<br>Follow up requirements	Handling of outcomes occurring prior to exposure	Notes
Goh, 2014 [55]	Angiotensin receptor blockers and risk of dementia: cohort study in UK Clinical Practice Research Datalink	Cohort study	CPRD	1995-2010	New diagnosis of dementia, defined by Read codes, excluding specific causes of dementia (e.g. dementia in neoplastic disease).	Yes	No	At least 6 months of registration with the GP practice<br>//<br>Outcome occurring in the first year after the index date were not considered	Excluded all individuals with a record of dementia or cognitive impairment prior to the date of entry in the study	-
Granerod, 2016 [3]	Increased rates of sequelae post-encephalitis in individuals attending primary care practices in the United Kingdom: a population-based retrospective cohort study	Nested case control study	CPRD	1998-2012	“cognitive problems (including memory loss, aphasia, difficulty processing information, difficulty reasoning, difficulty concentrating and learning disability)”	Not provided in the original study but obtained from the authors	None stated	None stated<br>//<br>At least one contact with the GP practice in the two years after the index date.	Analysis restricted to those at risk of a new-onset outcome, defined as no code in the year prior to the index date.	Dementia diagnosis were considered separately
Hippisley-Cox, 2010 [56]	Unintended effects of statins in men and women in England and Wales: population based cohort study using the QResearch database	Cohort study	QResearch	2002-2008	Dementia, defined with Read codes, not further specified.	No; states that code list is available on request	No	At least one year of registration with the practice<br>//<br>ND	Incident cases included in the study	-

Author, year of publication<br><br>Title	Study title	Type of study	Database(s)	Study period (years)	Outcome description	List of codes available	Validation of list of codes	EHR-related eligibility criteria for the study<br>//<br>Follow up requirements	Handling of outcomes occurring prior to exposure	Notes
Imfeld, 2012 [57]	Metformin, other antidiabetic drugs, and risk of Alzheimer's disease: a population-based case-control study	Nested case control study	CPRD	1998-2008	"either a diagnosis of AD followed by at least one prescription for an AD drug or vice versa; a diagnosis of dementia followed by at least two prescriptions for an AD drug; at least two recordings of an AD diagnosis; an AD diagnosis after a specific dementia test (e.g., Mini-Mental State Examination (MMSE), Clock Drawing Test (CDT), or Abbreviated Mental Test (7-min screen)), a referral to a specialist (e.g., neurologist, geriatrician, or psychogeriatrician), an assessment based on a neuroimaging technique (e.g., magnetic resonance imaging (MRI), computed tomography (CT), or single-photon emission CT (SPECT)); or an AD diagnosis preceded or followed by any recorded dementia symptoms (e.g., memory impairment, aphasia, apraxia, or agnosia)."	No	Yes<br><br>GP questionnaire	Patients with >3 years of active history in the database were excluded.	First time diagnosis of Alzheimer's disease included	-

Author, year of publication<br><br>Title	Study title	Type of study	Database(s)	Study period (years)	Outcome description	List of codes available	Validation of list of codes	EHR-related eligibility criteria for the study<br>//<br>Follow up requirements	Handling of outcomes occurring prior to exposure	Notes
Imfeld, 2013 [58]	Epidemiology, co-morbidities, and medication use of patients with Alzheimer's disease or vascular dementia in the UK	Descriptive cohort study	CPRD	1998-2008	"A patient was required to have either: 1) a diagnosis of AD followed by at least one prescription for an AD drug or vice versa; 2) a diagnosis of unspecific dementia followed by at least two prescriptions for an AD drug; 3) at least two recordings of an AD diagnosis; 4) an AD diagnosis after a specific dementia test (e.g., Mini Mental State Examination, Clock Drawing Test, or Abbreviated Mental Test [7-Minute Screen]), a referral to a specialist (e.g., neurologist, geriatrician or psycho-geriatrician), or an assessment based on neuroimaging technique (e.g., magnetic resonance imaging, computed tomography, or single photon emission computed tomography); or 5) an AD diagnosis preceded or followed by any recorded dementia symptoms (e.g., memory impairment, aphasia, apraxia, or agnosia). In addition, AD patients with a recording of any other specific dementia diagnosis (e.g., VaD, Pick's disease, or dementia with Lewy bodies) after the index date were not eligible, as well as those AD patients with a recording of stroke within two years prior to the index date."	No	Yes<br><br>GP questionnaires	None stated	Considered incident cases during the study period only	"cases were not eligible if they had had a stroke before the index date (because this is more indicative of a diagnosis of vascular dementia (VaD)16) or a recording of any other specific dementia diagnosis (e.g., VaD, Pick's disease, or Lewy body dementia) after the index date."

Author, year of publication<br><br>Title	Study title	Type of study	Database(s)	Study period (years)	Outcome description	List of codes available	Validation of list of codes	EHR-related eligibility criteria for the study<br>//<br>Follow up requirements	Handling of outcomes occurring prior to exposure	Notes
Imfeld, 2015 [59]	Benzodiazepine Use and Risk of Developing Alzheimer's Disease or Vascular Dementia: A Case-Control Analysis	Case control	CPRD	1998-2013	"first time diagnosis of AD, VaD, or any unspecified dementia (based on Read codes) (…), or who received a first-time prescription for an acetylcholinesterase inhibitor (i.e. donepezil, rivastigmine, galantamine, or tacrine) or the N-methyl-D-aspartate (NMDA) receptor antagonist memantine (…) To increase the probability of including only well-defined AD or VaD cases, a validated algorithm was applied (…) this algorithm was based on recordings of specific dementia tests [e.g. Mini-Mental State Examination (MMSE), Clock Drawing Test (CDT), or Abbreviated Mental Test (7-Minute Screen)], referrals to specialists (e.g. neurologists, geriatricians or psycho-geriatricians), brain imaging [computed tomography (CT), magnetic resonance imaging (MRI), or single photon emission computed tomography (SPECT)], or dementia symptoms (memory impairment, aphasia, apraxia, or agnosia) supportive of a diagnosis of a specific dementia subtype (i.e. AD or VaD)."	No	Yes<br><br>GP questionnaire	At least three years of active history in the database before diagnosis<br>//<br>ND	Considered incident cases during the study period only	-
Jick, 2000 [60]	Statins and the risk of dementia	Nested case control study	CPRD	1992-1998	First time diagnosis of dementia or Alzheimer's disease	No	No but refers to previous study where the validity of the same list of codes was assessed	None stated	Incidence cases considered only	-

Author, year of publication<br><br>Title	Study title	Type of study	Database(s)	Study period (years)	Outcome description	List of codes available	Validation of list of codes	EHR-related eligibility criteria for the study<br>//<br>Follow up requirements	Handling of outcomes occurring prior to exposure	Notes
Judge, 2017 [61]	Protective effect of anti-rheumatic drugs on dementia in rheumatoid arthritis patients		CPRD	1995-2011	Dementia, including Alzheimer's dementia, vascular dementia, and mixed dementia.	Yes	No	At least one year of up to standard registration data before the index date<br>//<br>ND	Considered incident cases only	-
Khan, 2011 [62]	Long-term health outcomes in a British cohort of breast, colorectal and prostate cancer survivors	Cohort study	CPRD	2003-2006	Dementia, defined with Read codes, not further specified	No; states that codes are available upon request	No	None stated	Only incident cases were considered	-
Khosrow-Khavar, 2017 [63]	Androgen deprivation therapy and the risk of dementia in patients with prostate cancer	Cohort study	CPRD	1988-2016	All incident cases of dementia, including Alzheimer's disease	Yes	None stated (refers to validation in previous studies)	Patients with less than 1 year of history in CPRD were excluded<br>//<br>All patients had to have at least 1 year of follow up data	Excluded patients with previous diagnosis of any dementia	-
Lu, 2016 [64]	Gout and the risk of Alzheimer's disease: a population-based, BMI-matched cohort study	Cohort study	THIN	1995-2013	Alzheimer's disease	Yes	Not in the original study; refers to a previous validation study	None stated	First diagnoses only	-
Mehta, 2017 [65]	Association of Hypoglycaemia With Subsequent Dementia in Older Patients With Type 2 Diabetes Mellitus	Cohort study	CPRD	2002-2012	Dementia defined by Read codes; used the same list of codes as another previous study.	No	None stated	None stated	Excluded patients with dementia diagnosed in the year prior to the index date	-

Author, year of publication<br><br>Title	Study title	Type of study	Database(s)	Study period (years)	Outcome description	List of codes available	Validation of list of codes	EHR-related eligibility criteria for the study<br>//<br>Follow up requirements	Handling of outcomes occurring prior to exposure	Notes
Mehta, 2016 [66]	Development and validation of the RxDx-Dementia risk index to predict dementia in patients with type 2 diabetes and hypertension	Cohort study	CPRD	2003-2012	Dementia, defined with a previously validated algorithm (refers to previous publication)	No	None stated	None stated	Considered incident cases only	Sensitivity analysis include the definition of dementia as clinical diagnosis or drug prescription
Perera, 2018 [67]	Dementia prevalence and incidence in a federation of European Electronic Health Record databases: The European Medical Informatics Framework resource	Cohort study	THIN	2004-2012	‘Codes that clearly indicated a dementia diagnosis, rather than those that were suggestive’	Yes	No	None stated	Not applicable (calculated incidence estimates)	-
Qizilbash, 2015 [68]	BMI and risk of dementia in two million people over two decades: a retrospective cohort study	Cohort study	CPRD	1992-2007	“Patients were classified as having dementia if, any of the following terms were recorded during follow-up: dementia, Alzheimer, Lewy body disease, Pick’s disease. Dementia recorded on a death certificate was also used.”	No	None stated	None stated	Excluded patients with dementia diagnosed in the year prior to the index date	-
Seshadri, 2001 [69]	Postmenopausal estrogen replacement therapy and the risk of Alzheimer disease	Cohort study	CPRD	1992-1998	First-time diagnosis of Alzheimer’s disease, senile dementia or presenile dementia	No	Yes<br><br>Case review	None stated	Only first time diagnosis were included	-

Author, year of publication<br><br>Title	Study title	Type of study	Database(s)	Study period (years)	Outcome description	List of codes available	Validation of list of codes	EHR-related eligibility criteria for the study<br>//<br>Follow up requirements	Handling of outcomes occurring prior to exposure	Notes
Strom, 2015 [70]	Statin therapy and risk of acute memory impairment	Nested case-control study	THIN	1987-2013	"The outcome for this study was the onset of acute, reversible memory impairment. Using Read codes Clinical Terms, version 2 (…), we sought codes with descriptions specifically pertaining to memory loss including amnesia, amnesia symptom, memory loss symptom, temporary loss of memory, short-term memory loss, transient global amnesia, drug-induced amnestic syndrome, non-alcoholic amnestic syndrome, amnesia (retrograde), memory lapses, minor memory lapses, and mild memory disturbance."	Yes	Yes<br><br>GP questionnaire for a random sample	At least one 365 of registration with the GP practice	Patients with codes for acute memory loss before the index date were excluded.	Excluded patients diagnosed with dementia
Turner, 2016 [46]	Ongoing impairments following transient ischaemic attack: retrospective cohort study	Matched-cohort study	THIN	2009-2013	Read codes for diagnoses (such as 28E0.00: mild cognitive impairment) and symptoms (such as 1B1A.12: memory loss symptom) related to overall cognitive impairment and impaired individual cognitive domains. 'Cognitive impairment included memory, attention, spatial awareness, perception, apraxia and executive functioning impairments but not a diagnosis of dementia.'	Not provided in the original study but obtained from the authors	None stated	Patients alive and registered with the practice 1 month after the index date	Excluded patients with fatigue recorded on the index date	Dementia diagnosis were not included
Walters, 2016 [71]	Predicting dementia risk in primary care: development and validation of the Dementia risk score using routinely collected data		THIN	2000-2011	Newly recorded dementia diagnoses, including Alzheimer's disease, vascular dementia, and unspecified or mixed dementia.<br><br>Excluding dementia associated with Parkinson's disease, Lewy body dementia, Huntingdon, Picks, HIV and drug induced and alcohol-related dementia.	No but available upon request from the authors	No	Excluded patients with less than one year of follow up data<br>//<br>follow up restricted to a maximum of 5 years	Excluded patients with dementia, cognitive impairment or memory symptoms at baseline	-

CPRD – Clinical Practice Research Datalink; GP – general practitioner; HES – Hospital Episodes Statistics; THIN – The Health Improvement Network; ONS – Office for National Statistics.

Table 5. Main characteristics of the eligible studies: fatigue.

Author, year of publication<br><br>Title	Study title	Type of study	Database(s)	Study period (years)	Outcome description	List of codes available	Validation of list of codes	EHR-related eligibility criteria for the study<br>//<br>Follow up requirements	Handling of outcomes occurring prior to exposure	Notes
Donegan, 2013 [72]	Bivalent human papillomavirus vaccine and the risk of fatigue syndromes in girls in the UK	Ecological and self-controlled case series	CPRD	2000-2011 and 2008-2011	Chronic fatigue syndrome including chronic fatigue syndrome/myalgic asthenia, post-viral fatigue syndrome, fibromyalgia, and neurasthenia.	No<br><br>(Obtained from the authors)	None stated	None stated<br>//<br>At least one year of follow up available	None stated	Sensitivity analysis considered 'incident fatigue' at the earliest recording of symptoms, referrals or diagnoses.
Collin, 2017 [73]	Trends in the incidence of chronic fatigue syndrome and fibromyalgia in the UK, 2001-2013: a Clinical Practice Research Datalink Study	Descriptive cohort study	CPRD	2001-2013	Diagnoses of chronic fatigue syndrome, fibromyalgia, post-viral fatigue syndrome, or asthenia/ debility diagnosis or referral to a specialist service	Yes	None stated	At least 12 months of up to standard data	Only new first ever diagnosis were considered	Symptoms of fatigue were considered separately.
Gallagher, 2004 [74]	Incidence of fatigue symptoms and diagnoses presenting in UK primary care from 1990 to 2001.	Descriptive cohort study	CPRD	1990-2001	Read codes for diagnosis of chronic fatigue syndrome; post-viral fatigue syndrome; asthenia/debility. Read codes for symptoms were classified separately.	No<br><br>(Stated that was available from the authors but could not be obtained)	None stated	Data prior to 1990 not shown due to lower numbers than expected and being the first years of data collection of the database.	Fatigue diagnosis was incident if there was no record of diagnoses in the previous year, with or without previous symptoms; symptoms were incident if there was no symptoms or diagnoses in the previous year.	-
Hamilton, 2009 [75]	Risk markers for both chronic fatigue and irritable bowel syndromes: A prospective case-control study in primary care	Nested case control study	CPRD	1988-2001	List of diagnostic codes for post-viral fatigue syndrome;<br>chronic fatigue syndrome.	No<br><br>(refers to another publication)	None stated	Only patients with complete records for 3 years prior to the index date were included	Not applicable	-

Petersen, 2006 [34]	Risk and predictors of fatigue after infectious mononucleosis in a large primary-care cohort	Nested case control cohort study	CPRD	1989-2000	Read codes for diagnoses of post-viral debility, post-viral fatigue syndromes, post-influenza debility and syndrome post-viral; symptoms of tiredness, malaise, lethargy, debility, and fatigue.	No<br><br>(Stated that was available from the authors but could not be obtained)	None stated	At least one year of data before diagnosis<br>//<br>At least one year of complete follow up after diagnosis	Included patients who did not have fatigue in the year before onset	-
Turner, 2016 [46]	Ongoing impairments following transient ischaemic attack: retrospective cohort study	Nested case-cohort study	THIN	2009-2013	First consultation after the index date with a Read code for symptoms or diagnosis of fatigue	No<br><br>Obtained from the authors	None stated	Practice with at least one year of up to standard data, and patients registered for at least one year with the practice.<br>//<br>Patient alive and registered with the practice 1 month after index	Excluded patients with fatigue recorded on the index date.	-

CPRD – Clinical Practice Research Datalink; THIN – The Health Improvement Network.

Table 6. Main characteristics of the eligible studies: pain.

Author, year of publication	Study title	Type of study	Database(s)	Study period (years)	Outcome description	List of codes available	Validation of list of codes	EHR-related eligibility criteria for the study // Follow up requirements	Handling of outcomes occurring prior to exposure	Notes
Becker, 2007 [76]	Migraine incidence, comorbidity and health resource utilization in the UK	Nested case-control study	CPRD	1994-2001	Migraine diagnosis	No	Yes<br><br>GP questionnaire	None stated	First-time diagnosis only	-
Campbell, 2015 [77]	In sickness and in health: A cross-sectional analysis of concordance for musculoskeletal pain in 13,507 couples	Descriptive	CiPCA	2005-2006	Widespread body pain:<br>"All relevant codes were formed into the five most common consultation body regions (back, knee, neck, shoulder, foot), as well as codes for osteoarthritis consultations. A further category of 'any musculoskeletal' consultations were formed inclusive of the above body regions and conditions, as well as consultations for unspecified pain (e.g. arthralgia), widespread pain conditions and other single body regions where the proportion of consultations were too few to perform meaningful separate analysis (e.g. head, arm, elbow, wrist, hand, hip, pelvis, thigh and buttock)."	Yes	None stated	None stated	None stated	-

Author, year of publication	Study title	Type of study	Database(s)	Study period (years)	Outcome description	List of codes available	Validation of list of codes	EHR-related eligibility criteria for the study // Follow up requirements	Handling of outcomes occurring prior to exposure	Notes
Hall, 2006 [78]	Epidemiology and treatment of neurophatic pain: the UK primary care perspective	Descriptive	CPRD	1992-2002	"A post-herpetic neuralgia record was a specific term for post-herpetic neuralgia or an acute herpes zoster term plus either neuropathy, or neuropathic pain, 3-6 months after the first acute herpes zoster entry. A trigeminal neuralgia record had a specific term for this diagnosis. Phantom limb pain was defined as a specific term or a term for amputation plus either a neuropathy or neuropathic pain record 3–24 months after the first amputation code. Patients were included in the painful diabetic neuropathy cohort if their record contained a specific term; a term for diabetic neuropathy with a prescription for a treatment for pain current at the date of diagnosis; a record of diabetes and neuropathic pain or record of diabetes and both neuralgia and a treatment for pain current on the date of the neuralgia code."	No	None stated	At least year of up to standard data	None stated	-

Author, year of publication	Study title	Type of study	Database(s)	Study period (years)	Outcome description	List of codes available	Validation of list of codes	EHR-related eligibility criteria for the study // Follow up requirements	Handling of outcomes occurring prior to exposure	Notes
Hall, 2013 [79]	An observational descriptive study of the epidemiology and treatment of neuropathic pain in a UK general population	Cohort study, descriptive	CPRD	2005-2010	"Five neuropathic pain cohorts (post-herpetic neuralgia, painful diabetic neuropathy, phantom limb pain, neuropathic back pain and neuropathic postoperative pain) were identified from (…) a single specific Read code, or a combination of Read and therapy codes as specified in a case definition. Postherpetic neuralgia was defined as a specific code for post-herpetic neuralgia, or a code for acute zoster plus either a code for neuropathy, or neuropathic pain, between three and six months after the first acute zoster entry. Phantom limb pain (PLP) was defined as a specific code, or a code for amputation plus either a code for neuropathy or neuropathic pain between three and twenty-four months after the first amputation code. The painful diabetic neuropathy cohort included patients with a specific code for painful diabetic neuropathy; those with a code for diabetes and a general code for neuropathic pain and a third group with a code for diabetic neuropathy (or diabetes and neuralgia) with a prescription for a neuropathic pain treatment which was initiated within 28 days of the date of the neuropathy/ neuralgia code."	No	Yes<br><br>GP questionnaires	At least of 1 year of data of good quality for research		None stated

Author, year of publication	Study title	Type of study	Database(s)	Study period (years)	Outcome description	List of codes available	Validation of list of codes	EHR-related eligibility criteria for the study // Follow up requirements	Handling of outcomes occurring prior to exposure	Notes
Mansfield, 2017 [80]	Identifying patients with chronic widespread pain in primary care	Cross sectional	CiPCA	2005-2009	(A) Recurrent region pain, define as: "In a period of 5 consecutive years, a patient fulfils all of 1-4:<br>1. At least 1 consultation for a musculoskeletal complaint in the axial skeleton (neck and back);<br>2. At least 1 consultation for an upper- or lower-limb complaint;<br>3. At least 1 consultation for a regional musculoskeletal complaint in each of 3 separate years;<br>4. At least 4 consultations for regional musculoskeletal complaints during the 5-year period."<br><br>(B) "Non specific generalized pain conditions, including fibromyalgia, fibrositis, rheumatism, myalgia, arthralgia, and polyalgia"	Yes	Yes	Patients not registered with the practice for the full 5-year period were excluded	Not applicable (cross sectional analysis)	-
Ruigomez, 2006 [81]	Chest pain in general practice: incidence, comorbidity and mortality	Case control study	CPRD	1996	Chest pain:<br>Codes for chest pain that did specify the type or location of the symptom.	Yes	None stated	Registered with the GP for at least 2 years<br>//<br>At least one entry in the records in the last 3 years before the study	Excluded patients with history of pain the past 2 years	-
Wallander, 2007 [82]	Unspecified abdominal pain in primary care: The role of gastrointestinal morbidity	Matched cohort study	CPRD	Before 1996	Abdominal pain:<br>Diagnosis of abdominal pain	Yes	None stated	At least one entry to the data in the three years prior to the study start.	Patients were excluded if they had a record of abdominal pain of any abdominal site or type in the 2 years before the study started.	-

Author, year of publication	Study title	Type of study	Database(s)	Study period (years)	Outcome description	List of codes available	Validation of list of codes	EHR-related eligibility criteria for the study // Follow up requirements	Handling of outcomes occurring prior to exposure	Notes
Zondervan, 1999 [83]	Prevalence and incidence of chronic pelvic pain in primary care: evidence from a national general practice database	Cohort study	Mediplus UK primary care database	1991-1995	Chronic pelvic pain: pain in the lower abdominal region persisting for at least six months.<br><br>"Episode of chronic pelvic pain: pelvic pain on two or more contacts, with at least six months (≥ 183 days) between the first and the last contact but with no period of more than one year (> 365 days) without a pelvic pain contact. An episode of chronic pelvic pain was defined as starting six months after the first contact and finishing at the contact preceding a pain-free, one-year interval (if any)."<br><br>"Excluded pain due to malignancy; chronic inflammatory and other defined bowel diseases such as Crohn's, coeliac disease, ulcerative colitis; acute conditions verified by having surgery such as appendicectomy, cholecystectomy; or pregnancy. Women with pelvic pain occurring only during menstruation (dysmenorrhoea) or sexual intercourse (dyspareunia) were also excluded."	No	None stated	None stated	Not applicable. Monthly incidence estimated as the number of new episodes in a given month as a proportion of	-

CiPCA - The Consultations in Primary Care Archive; CPRD – Clinical Practice Research Datalink;. NSAIDS - Nonsteroidal anti-inflammatory drug.

Table 7. Main characteristics of the eligible studies: sexual dysfunction.

Author, year of publication<br><br>Title	Study title	Type of study	Database(s)	Study period (years)	Outcome description	List of codes available	Validation of list of codes	EHR-related eligibility criteria for the study<br>//<br>Follow up requirements	Handling of outcomes occurring prior to exposure	Notes
Blumentals, 2003 [84]	Antihypertensive treatment and erectile dysfunction in a cohort of type II diabetes patients	Nested case-control study	CPRD	1987-2001	Erectile dysfunction, ascertained from the diagnosis codes, not further specified	No	None stated	None stated	None stated	-
Hagberg, 2016 [85]	Risk of erectile dysfunction associated with use of 5-alpha reductase inhibitors for benign prostatic hyperplasia or alopecia: population based studies using the Clinical Practice Research Datalink	Nested case control study	CPRD	1992-2011	Erectile dysfunction: "diagnosis of erectile dysfunction or impotence, prescription for a phosphodiesterase type 5 inhibitor (eg, sildenafil, tadalafil, or vardenafil) where the strength and quantity prescribed was indicated for treatment of erectile dysfunction, or record of procedures for treatment of erectile dysfunction (eg, penile prosthesis, penile injection, or other operations for treatment of erectile dysfunction)."<br>Non-erectile dysfunction: including ejaculatory disorder, psychosexual dysfunction, or low libido	No	None stated	At least 3 years of history before the cohort entry date;	Incident cases considered after the index date.	-
Khan, 2011 [62]	Long-term health outcomes in a British cohort of breast, colorectal and prostate cancer survivors: a database study	Cohort study	CPRD	2003-2006	Erectile dysfunction: "new prescriptions for sildenafil (Viagra, Pfizer, NY, United States), apomorphone hydrochloride, vardenafil (Levitra, Bayer Healthcare Pharmaceuticals, New Haven, USA), alprostadil (an injectable treatment) and tadalafil (Cialis, Lilly, USA)"	No	None stated	None stated	Excluded patients with erectile dysfunction recorded before the index date	-
Morant, 2008 [86]	Increased sexual dysfunction in men with storage and voiding lower urinary tract symptoms	Cross-sectional analysis	THIN	2000-2007	Male sexual dysfunction	Yes	None stated	None stated	None stated	-
Schlesinger, 2018 [87]	Gout and the Risk of Incident Erectile Dysfunction: A Body Mass Index-matched Population-based Study	Cohort study	THIN	1995-2012	Erectile dysfunction noted by the presence of the Read code E227311	Yes	None stated	At least one year of registration with the practice before the index date	Excluded prevalent cases at baseline	-

Author, year of publication<br><br>Title	Study title	Type of study	Database(s)	Study period (years)	Outcome description	List of codes available	Validation of list of codes	EHR-related eligibility criteria for the study<br>//<br>Follow up requirements	Handling of outcomes occurring prior to exposure	Notes
Sultan, 2017 [88]	Gout and subsequent erectile dysfunction: a population-based cohort study from England	Cohort study	CPRD, HES	1998-2004	Erectile dysfunction, ascertained from the medical codes, not further specified	Yes	None stated	At least 1 year of follow up data	Only incident cases after the index date were considered; cases in the first 6 months of registration were considered prevalent.	-

CPRD – Clinical Practice Research Datalink; HES – Hospital Episodes Statistics; THIN – The Health Improvement Network.

Table 8. Main characteristics of the eligible studies: sleep disorder.

Author, year of publication<br><br>Title	Study title	Type of study	Database(s)	Study period (years)	Outcome description	List of codes available	Validation of list of codes	EHR-related eligibility criteria for the study<br>//<br>Follow up requirements	Handling of outcomes occurring prior to exposure	Notes
Roddy, 2013 [89]	The association of gout with sleep disorders: A cross-sectional study in primary care	Matched cohort study	CiPCA and PiPCA	2001-2008	Read codes for sleep disorders, excluding codes for sleep apnoea which were classified separately	Yes	None stated	None stated	None stated	-
Wallander, 2007 [90]	Morbidity associated with sleep disorders in primary care: A longitudinal cohort study	Cohort study	CPRD	1996	Read codes for sleep disorder including insomnia, hypersomnia, and sleep disturbance	Yes	None stated	Registered with a general practitioner for at least 2 years and having at least 1 entry in CPRD in the previous 3 years.	Patients with a consultation for sleep disorders during the 2 years before the start of the study were excluded.	-

CPRD – Clinical Practice Research Datalink; CiPCA - The Consultations in Primary Care Archive; PiPCA - The Prescriptions in Primary Care Archive.

Table 9. Main characteristics of the eligible studies: fatal and non-fatal self-harm.

Author, year of publication<br><br>Title	Study title	Type of study	Database(s)	Study period (years)	Outcome description	List of codes available	Validation of list of codes	EHR-related eligibility criteria for the study<br>//<br>Follow up requirements	Handling of outcomes occurring prior to exposure (applicable to self-harm only)	Notes
Andersohn, 2010 [91]	Use of antiepileptic drugs in epilepsy and the risk of self-harm or suicidal behaviour	Nested case-control study	CPRD	1990-2005	"Potential cases were identified using predefined medical codes of self-harm (i.e., without a clear suicidal intention [intentional self-harm]) or suicidal behaviour (i.e., with a clear suicidal intention [attempted suicide]). Patients who died were also considered as potential cases if suicidal thoughts were recorded within 4 weeks before death."	Yes	Yes, record review	At least one year of data with the GP practice	Models adjusted for history of self-harm at baseline	In an additional analysis, the patients with codes for self-harm without explicitly mention to suicidal behaviour were excluded.
Arana, 2010 [92]	Suicide-related events in patients treated with antiepileptic drugs	Cohort study	THIN	1988-2008	"Cases of suicide-related events were based on codes for suicide, attempted suicide, and intentional self-inflicted injuries plus suicide. A completed suicide was defined as a code for suicidality followed by a code for death in the following month and a final date of any administrative activity in the database or disenrollment within 6 months after the suicidality code. If the disenrollment date occurred more than 6 months after a suicidality code, we reviewed the patient's profile. Patients with a last medical or other health related code that was recorded within 1 month after the suicide date were also considered to have completed suicide."	No	Yes<br><br>GP questionnaire, record and death certificate review	Patients were eligible if they were enrolled with a clinical practice for at least 6 months during the study period	Patients with personal or family history of suicide attempt were excluded	-
Carr, 2016 [93]	The epidemiology of self-harm in a UK-wide primary care patient cohort, 2001-2013	Descriptive cohort study	CPRD	2001-2013	"Read codes incorporating all cases across the spectrum from milder forms of non-suicidal behaviour through to near-fatal suicide attempts (…) We excluded codes that referred only to thoughts of self-harm or suicidal ideation and alcohol-related codes, unless intent to actively harm oneself was specified.	Yes	None stated	None stated	None stated	None stated

Author, year of publication<br><br>Title	Study title	Type of study	Database(s)	Study period (years)	Outcome description	List of codes available	Validation of list of codes	EHR-related eligibility criteria for the study<br>//<br>Follow up requirements	Handling of outcomes occurring prior to exposure (applicable to self-harm only)	Notes
Carr, 2017 [94]	Premature Death Among Primary Care Patients With a History of Self-Harm	Cohort study	CPRD ONS	2001-2013	Suicide, ascertained with ICD-10 codes in the ONS mortality data; including open verdicts.	Yes	Not applicable	At least one year of registration with the practice prior to the index date;	Not applicable.	-
Coupland, 2011 [95]	Antidepressant use and risk of adverse outcomes in older people: Population based cohort study	Cohort study	QResearch, ONS	1996-2007	Read codes for attempted suicide or self-harm, not further specified	No	None stated	At least one year of registration with the practice prior to the index date;	None stated	Suicide was also an outcome, but data for this outcome was not analysed due to small numbers.
Coupland, 2015 [96]	Antidepressant use and risk of suicide and attempted suicide or self-harm in people aged 20 to 64: cohort study using a primary care database	Cohort study	QResearch, ONS	2000-2011	"code for suicide or an open verdict in their linked death certificate, or patients who had a Read code for attempted suicide or self-harm who died within 30 days.	No<br><br>(refers to codes used in other studies	None stated	At least one year of registration with the practice prior to the index date;<br>//<br>Most analysis restricted to the first 5 years of follow up.	Excluded patients with a previous attempted suicide or self-harm event recorded at baseline	-
Fardet, 2012 [2]	Suicidal behavior and severe neuropsychiatric disorders following glucocorticoid therapy in primary care	Cohort study	THIN	1990-2008	Cases of suicide or suicide attempt	No	Yes<br><br>Review of death certificates	≥6 months of registration with the primary care practice	Hazards ratios adjusted for past history of neuropsychiatric disorders (yes/no)	Outcome was eligible if there was no record of the outcome in the previous 6 months
Donovan, 1996 [97]	The use of the General Practice Research Database (GPRD) to examine potential links between antidepressant medication and the incidence of suicide	Cohort study	CPRD	1988-1993	Suicide, not further specified	No	None stated	None stated	Prior suicidal history considered as a risk factor for suicide	-
Doyle, 2016 [98]	Suicide risk in primary care patients diagnosed with a personality disorder: a nested case control study	Nested case control study	CPRD, ONS	2002-2011	Suicides and open verdicts ascertained via linkage to ONS mortality data	Yes<br><br>(ICD-10 codes)	Not applicable<br><br>(Data linked to the gold-standard)	At least one year of up to standard CPRD data	Not applicable	-

Author, year of publication<br><br>Title	Study title	Type of study	Database(s)	Study period (years)	Outcome description	List of codes available	Validation of list of codes	EHR-related eligibility criteria for the study<br>//<br>Follow up requirements	Handling of outcomes occurring prior to exposure (applicable to self-harm only)	Notes
Gao, 2013 [99]	Association between body mass index and suicide, and suicide attempt among British adults: The health improvement network database	Cohort study	THIN	2000-2007	Suicide: "defined in two ways (…): 1) patients with a Read code of suicide attempt confirmed by death within 3 months or 2) patients who did not have a Read code of suicide attempt, but whose cause of death might be suicide. To identify patients using the second approach, we first searched for all deaths (…) the cause of death was reviewed. In addition, free text records were searched for potential suicide as cause of death, including suicide, deliberate drug overdose or self-harm, self-inflicted injuries, poisoning, asphyxiation, trauma, and acute or multiple organ failure. Death certificates for these patients were requested for the final verdict of the cause of death.<br><br>Suicide attempt: "identified using the Read codes for suicide and then by confirming that the patient was still alive for at least 3 months from the time of the event."	No	None stated	None stated	None stated	-
Gunnell, 2009 [19]	Varenicline and suicidal behaviour: a cohort study based on data from the General Practice Research Database	Cohort study	CPRD	2006-2008	OXMIS and Read terms for fatal and non-fatal self-harm.	No	Yes<br><br>Record review	At least 1 years of CPRD records before index date;	Considered the potential confounding effect of previous self-harm or suicidal thoughts in analysis.	-

**Author, year of publication**<br><br>**Title**	**Study title**	**Type of study**	**Database(s)**	**Study period (years)**	**Outcome description**	**List of codes available**	**Validation of list of codes**	**EHR-related eligibility criteria for the study // Follow up requirements**	**Handling of outcomes occurring prior to exposure (applicable to self-harm only)**	**Notes**
Hall, 2009 [100]	Validation of death and suicide recording on the THIN UK primary care database	Validation study	THIN	2002-2004	"Coded and free text records were searched to identify any entry for suicide or a medical cause of death which might indicate suicide, including trauma, poisoning, overdose, asphyxiation, acute or multiple organ failure or a suggestion that death was intentional. An electronic record of suicide was accepted."	Yes	Yes<br><br>(Validation study, record review)	None stated	Not applicable (validation study)	-
Hagberg, 2016 [21]	Incidence rates of suicidal behaviors and treated depression in patients with and without psoriatic arthritis using the Clinical Practice Research Datalink	Cohort study	CPRD	1998-2012	Suicidal behaviours, defined as: "diagnosis of suicidal ideation, suicide attempt, and/or suicide recorded after the cohort entry date. (…) If a patient had a code for suicide, but had not died, the event was classified as a suicide attempt. Suicidal ideation, attempts, and suicide were considered separately; thus a patient may have been included in more than one analysis.	No	Yes, record review	At least one year of registration with the practice prior to the index date	Patients with suicidal behaviors were included "because patients could recover from suicidal behaviors with treatment or consultation."	-
Hayes, 2016 [101]	Self-harm, Unintentional Injury, and Suicide in Bipolar Disorder During Maintenance Mood Stabilizer Treatment: A UK Population-Based Electronic Health Records Study	Cohort study	THIN	1995-2013	"emergency department or primary care attendance for self-harm during the period of drug exposure and the 3 months afterward. This outcome included Read codes for intentional poisoning, intentional self injurious behavior, and self-harm acts of uncertain intent. (…) Secondary outcomes were unintentional injury (eg, falls or motor vehicle crashes) seen in primary or secondary care and a record of the patient's suicide during this period"	No	Refers to previous validation of the list of codes	None stated	Not applicable (the outcome was diagnoses during the exposure period)	-

Author, year of publication<br><br>Title	Study title	Type of study	Database(s)	Study period (years)	Outcome description	List of codes available	Validation of list of codes	EHR-related eligibility criteria for the study<br>//<br>Follow up requirements	Handling of outcomes occurring prior to exposure (applicable to self-harm only)	Notes
Hesdorffer, 2012 [4]	Epilepsy, suicidality, and psychiatric disorders: a bidirectional association	Cohort study	CPRD	1990-2008	Read codes for suicidality including codes for attempted suicide and completed suicide.	No	None stated	At least 3 years of data before the index date, and at least 1 code for a medical or drug code in the 6 months before the index<br>//<br>At least 1 day of data after index date; follow up duration: 3 years after index date	Excluded subjects with a record of the outcome before the study date.	-
Hesdorffer, 2016 [102]	Occurrence and Recurrence of Attempted Suicide Among People With Epilepsy	Cohort study	CPRD	1988-2013	Diagnoses of attempted suicide identified with Read codes, not further specified.<br><br>Suicide attempts divided into incident or recurrent.	No	None stated	At least 6 month of complete records before the index date and ≥1 medical or drug codes for a condition other than epilepsy in the 6 months before the index date.<br>//<br>At least 1 day of data post index date	None stated	-

Author, year of publication<br><br>Title	Study title	Type of study	Database(s)	Study period (years)	Outcome description	List of codes available	Validation of list of codes	EHR-related eligibility criteria for the study<br>//<br>Follow up requirements	Handling of outcomes occurring prior to exposure (applicable to self-harm only)	Notes
Haste, 1998 [103]	Potential for suicide prevention in primary care? An analysis of factors associated with suicide.	Matched cohort study	CPRD	1991-1993	Suicide: "were identified from the database in two ways: 1. Patients with a record of suicide in the notes; and 2. Patients who did not have a record of suicide, but whose record of cause of death on the database suggested that suicide might be possible. These causes included death from carbon monoxide poisoning (excluding accidental poisoning), hanging, suicidal or accidental overdose, and reference to self-inflicted injury. (…) Cases where the verdict was open were included."	No	Yes<br><br>GP confirmation, review of death certificates	None stated	Not applicable (cases of completed suicide only)	-
Jick, 1995 [104]	Antidepressants and suicide	Cohort study	CPRD	1988-1993	"Cases of suicide were identified from the computer record from among all the study subjects who died. When the cause of death was recorded as suicide or was considered to be uncertain, we obtained further information from the general practitioner and the death certificate to determine the final diagnosis and means of committing suicide."	No	Yes, GP questionnaire	At least 6 month of registration with the practice prior to the index date	Not applicable (cases of completed suicide only)	-
Jick, 1998 [105]	A study of the relation of exposure to quinolones and suicidal behaviour	Nested case control study	CPRD	1991-1995	Three case groups:<br>1) committed suicide;<br>2) had a diagnosis of attempted suicide;<br>3) had a diagnosis of suicidal ideation.<br><br>"If the subject had more than one case diagnosis during the study period, only the first such diagnosis was considered."	Yes	None stated	>18 months of information on drugs prescribed and diagnoses recorded prior to the index.	None stated	-

Author, year of publication<br><br>Title	Study title	Type of study	Database(s)	Study period (years)	Outcome description	List of codes available	Validation of list of codes	EHR-related eligibility criteria for the study<br>//<br>Follow up requirements	Handling of outcomes occurring prior to exposure (applicable to self-harm only)	Notes
Jick, 2000 [106]	Isotretinoin use and risk of depression, psychotic symptoms, suicide, and attempted suicide	Cohort study	CPRD	?	Suicide and inpatient or outpatient code for attempted suicide	Yes	None stated	Between 5 years and 6 months of clinical records before the index date<br>//<br>At least 1 year of records post index date	Controlled for history of attempted suicide	-
Jick, 2004 [107]	Antidepressants and risk of suicidal behaviours	Cohort study	CPRD	1993-1999	Nonfatal suicidal behaviour: “Cases were those who (1) had a first-time recorded diagnosis of nonfatal suicidal ideation (…) or attempted suicide”<br><br>Suicide: patients who committed suicide, not further specified.	Yes	None stated	At least 2 years of registration with the practice prior to the index date	None stated	-
Jick, 2009 [108]	Rate of suicide in patients taking montelukast	Cohort study	CPRD	1998-2007	‘computer recorded diagnosis of suicide’	No	None stated	None stated	None stated	
Kurd, 2010 [6]	The risk of depression, anxiety, and suicidality in patients with psoriasis: A population-based cohort study	Matched cohort study	CPRD	1987-2002	‘Suicidality was defined as diagnosis of suicidal ideation, suicide attempt, or suicide’, defined by diagnostic Read or OXMIS codes.	No<br><br>Refers to diagnostic codes used in other publications.	None stated	None stated	In sensitivity analysis, the patients with a diagnosis of the outcome measured prior to or within six months of the index date were excluded.	-
Kotz, 2015 [28]	Cardiovascular and neuropsychiatric risks of varenicline: a retrospective cohort study	Cohort study	QResearch	2007-2012	Fatal and non-fatal self-harm, not further specified	None stated	None stated	Registered for >12 months before data extraction<br>//<br>Follow up duration 6 months	History of neuropsychiatric events considered as confounders	-

Author, year of publication<br><br>Title	Study title	Type of study	Database(s)	Study period (years)	Outcome description	List of codes available	Validation of list of codes	EHR-related eligibility criteria for the study<br>//<br>Follow up requirements	Handling of outcomes occurring prior to exposure (applicable to self-harm only)	Notes
Kotz, 2017 [27]	Cardiovascular and neuropsychiatric risks of varenicline and bupropion in smokers with chronic obstructive pulmonary disease	Cohort study	QResearch	2001-2012	Fatal and non-fatal self-harm, not further specified	None stated	None stated	None stated<br>//<br>Follow up duration 6 months	History of neuropsychiatric events considered as confounders	-
Lalmohamed, 2012 [109]	Causes of death in patients with multiple sclerosis and matched referent subjects: A population-based cohort study	Cohort study	CPRD<br>HES<br>ONS mortality	2001-2008	Death by accident or suicide ascertained in the ONS mortality database	Yes (ICD-10 codes)	Not applicable (ONS mortality database, ICD-10 codes)	≥1 year of data available before index date	None stated	-
Martinez, 2005 [110]	Antidepressant treatment and the risk of fatal and non-fatal self harm in first episode of depression: nested case-control study	Nested case control study	CPRD	1995-2001	Non-fatal self-harm (drug overdose, deliberate self-laceration, poisoning, and non-fatal suicide attempts using other methods)<br><br>Suicide, identified by OXMIS and Read codes	No<br><br>Obtained from the authors	Yes,<br><br>Review of the death certificates and free text entries	≥1 year of data available before index date	Model adjusted for history of non-fatal self-harm	"People with an episode of non-fatal self harm were not censored in analyses with suicide as the end point."
Meier, 2004 [9]	The Risk of Severe Depression, Psychosis or Panic Attacks with Prophylactic Antimalarials	Cohort study	CPRD	1990-1999	Suicide defined by OXMIS and/or ICD-8 codes	No	Yes<br><br>'reviewed a list of all cases to determine inclusion/ exclusion'	≥1 year of data available before index date and "some GPRD activity (diagnoses or prescriptions) recorded after the index date"<br>//<br>Patients were censored 18 months after exposure date	Not applicable (suicide as an outcome only)	-
Mines, 2005 [111]	Prevalence of risk factors for suicide in patients prescribed venlafaxine, fluoxetine, and citalopram	Cohort study	CPRD	1995-2002	Suicidal behaviour in the year prior to index date	Yes	None stated	≥1 year of data available	None stated	-

Author, year of publication<br><br>Title	Study title	Type of study	Database(s)	Study period (years)	Outcome description	List of codes available	Validation of list of codes	EHR-related eligibility criteria for the study<br>//<br>Follow up requirements	Handling of outcomes occurring prior to exposure (applicable to self-harm only)	Notes
Osborn, 2008 [112]	Suicide and severe mental illnesses. Cohort study within the UK general practice research database	Matched cohort study	CPRD	1987-2002	Suicide, not further specified	No	None stated	None stated	None stated	
Rubino, 2007 [113]	Risk of suicide during treatment with venlafaxine, citalopram, fluoxetine, and dothiepin: retrospective cohort study	Cohort study	CPRD	1995-2005	Completed suicide: coding for death associated with mention of suicide in free text or by code for suicide in the medical record and a statement of death in the administrative record (30 days either way);<br><br>First attempted suicide (non-fatal event).	Yes	Yes<br><br>Free text search	At least one year of registration with the practice prior to the index date	Suicide attempts considered as a confounder in the models	"excluded records that at the review of free text notes did not seem to represent attempted suicide - for example, unintentional overdose or self harm without suicidal intent"
Schneider, 2010 [37]	COPD and the risk of depression	Cohort study	CPRD	1995-2005	'incident suicide or suicidal ideation', not further specified	No<br><br>Obtained from the authors	None stated	Patients with less than 3 years of active recording history prior to the date of the COPD diagnosis were excluded;	Patients with previous history of depression, suicide, suicidal ideation, etc., prior to the index date were excluded	-

Author, year of publication<br><br>Title	Study title	Type of study	Database(s)	Study period (years)	Outcome description	List of codes available	Validation of list of codes	EHR-related eligibility criteria for the study<br>//<br>Follow up requirements	Handling of outcomes occurring prior to exposure (applicable to self-harm only)	Notes
Schuerch, 2016 [114]	Impact of varying outcomes and definitions of suicidality on the associations of antiepileptic drugs and suicidality: comparisons from UK Clinical Practice Research Datalink (CPRD) and Danish national registries (DNR)	Validation study	CPRD<br>HES<br>ONS	1996-2009	<u>Suicidal ideation/intent:</u><br>"Recorded medical terms from the clinical and referral module, plus reasons for transfer out of the general practices to identify patients with one of these outcomes"<br><br><u>Suicide attempt:</u><br>"Recorded medical terms from the clinical and referral module, plus reasons for transfer out of the GP-practice to identify patients with one of these outcomes from CPRD."<br><br><u>Completed suicide:</u><br>"Term of suicidal attempt or ideation occurring simultaneously with a recording of death (+/- 4 weeks), death recorded as reason for leaving the practice (registering out), and a final date of any administrative activity in the database of disenrollment within six months after suicidality code."	Yes	Yes (validation study)	At least 6 months of data before the index date<br>//	Patients with history of suicide attempts, self-harm or suicidal ideation/intent in the 6 months before index were excluded	-
Thomas, 2013 [40]	Smoking cessation treatment and risk of depression, suicide, and self harm in the Clinical Practice Research Datalink: prospective cohort study	Nested case-control study	CPRD, HES and ONS mortality data	2006-2011	Incidence fatal and non-fatal self-harm (as measured by death from suicide in the ONS mortality database) and hospital admission for self-harm (as recorded in the HES database). Includes open verdicts.	Yes for ONS database; no for Read codes.	None stated	At least one year of registration with the practice prior to the index date	Previous self-harm considered as a potential confounder in analysis	-

Author, year of publication<br><br>Title	Study title	Type of study	Database(s)	Study period (years)	Outcome description	List of codes available	Validation of list of codes	EHR-related eligibility criteria for the study<br>//<br>Follow up requirements	Handling of outcomes occurring prior to exposure (applicable to self-harm only)	Notes
Thomas, 2013 [115]	Validation of suicide and self-harm records in the Clinical Practice Research Datalink	Validation study	CPRD, HES, ONS mortality data, and Multicentre study of self-harm	1998-2010	“Cases of suicide and self-harm (the ‘events’) were identified by extracting all records with Read codes for suicide, attempted suicide and self-harm (…). Given that suicide-related Read codes may refer to both fatal and nonfatal suicide attempts, completed suicides within the CPRD were identified using the conventional CPRD approach of linking patient deaths to Read codes for suicide that were recorded within 95 days of the CPRD derived death dates”	Yes	Yes<br><br>(Validation study)	Registered with a practice contributing with up to standard data, and having acceptable records for research.	Not applicable (descriptive study on the incidence of self-harm)	-
Tyrrell, 2016 [116]	Changes in poisonings among adolescents in the UK between 1992 and 2012: a population based cohort study	Cohort study	THIN	1992-2012	“poisonings [categorised] as intentional, unintentional, unknown intent or alcohol related. Where Read codes explicitly described the intent using the words ‘deliberate’ or ‘intentional’ (intentional); ‘accidental’ (unintentional); and ‘unknown’ or ‘unspecified’ (unknown) then they were classified as such. The words ‘suicide’, ‘self-inflicted’, ‘self-poisoning’ or ‘overdose’ were also used to categorise a poisoning as intentional, unless otherwise specified.”	No	None stated	None stated	First poisoning within the observation period	Multiple events occurring in the same individual within a month were counted as one event only
Webb, 2012 [117]	Risk of self-harm in physically ill patients in UK primary care	Nested case control study	CPRD	2001-2008	Suicidal ideation, defined by Read coding descriptions that included the terms ‘suicide and self-inflicted,’ ‘suicide and self-harm,’ ‘para-suicide’ or ‘attempted suicide.’ Cases that died following the code were excluded.	No	None stated	At least two years of up to standard CPRD data	None stated	-
Webb, 2012 [118]	Suicide risk in primary care patients with major physical diseases: A case-control study	Nested case control study	CPRD and ONS mortality data	2001-2008	Suicides as mentioned in the death certificate, and open verdicts.	Yes	Not applicable (ONS mortality data)	At least two years of up to standard CPRD data	Not applicable (study of suicide only)	-

Author, year of publication<br><br>Title	Study title	Type of study	Database(s)	Study period (years)	Outcome description	List of codes available	Validation of list of codes	EHR-related eligibility criteria for the study<br>//<br>Follow up requirements	Handling of outcomes occurring prior to exposure (applicable to self-harm only)	Notes
Wijlaars, 2013 [119]	Suicide-related events in young people following prescription of SSRIs and other antidepressants: A self-controlled case series analysis	Nested case control study	THIN	1995-2009	Suicide attempts, suicidal ideation and completed suicide.<br><br>Completed suicides: "Read codes that were confirmed by a date of death within 2 weeks of the suicide event date. We searched a cause of death, if available."<br>Excluded cases classified as open verdicts.	No<br><br>Refers to a previous list, that was updated for the study	Yes<br><br>Record review	At least 6 months of up to standard CPRD data	Accounted for in analysis	-
Windfuhr, 2016 [120]	Suicide risk linked with clinical consultation frequency, psychiatric diagnoses and psychotropic medication prescribing in a national study of primary-care patients	Nested case-control study	CPRD and ONS mortality data	2002-2011	International Classification of Disease version 10 (ICD-10) codes X60-84, Y10-34 (excluding Y33.9), Y87.0, Y87.2<br><br>Includes open verdicts	Yes	Not applicable (ONS mortality data)	≥1 year of data available after up to standard data	None stated	-
Yang, 2003 [44]	Lipid-lowering drugs and the risk of depression and suicidal behaviour	Matched cohort study	CPRD	1991 onwards	Suicidal behaviour, ideation suicidal, suicidal plan, suicidal thoughts, attempted suicide, threat suicide, suicidal drug overdose, drug overdose, para suicide and suicide.	Yes	No<br><br>(refers to previous studies assessing suitability of the records)	Patients with history of suicidal behaviour prior to study start were excluded	None stated	-

CPRD – Clinical Practice Research Datalink; THIN – The Health Improvement Network; PCCIU – Primary Care Clinical Informatics Unit; SSRI - selective serotonin re-uptake inhibitors; GP – General practice; MAOIs - monoamine oxidase inhibitors; CiPCA - Consultations in primary care archive; PiPCA - Prescriptions in primary care archive.

References

1. Bouras G, SR Markar, EM Burns, HA Mackenzie, et al. Linked Hospital and Primary Care Database Analysis of the Incidence and Impact of Psychiatric Morbidity Following Gastrointestinal Cancer Surgery in England. Ann Surg 2016:264:93-9.

2. Fardet L, I Petersen, and I Nazareth. Suicidal behavior and severe neuropsychiatric disorders following glucocorticoid therapy in primary care. Am J Psychiatry 2012:169:491-7.

3. Granerod J, NW Davies, PP Ramanuj, A Easton, et al. Increased rates of sequelae post-encephalitis in individuals attending primary care practices in the United Kingdom: a population-based retrospective cohort study. J Neurol 2017:264:407-415.

4. Hesdorffer DC, L Ishihara, L Mynepalli, DJ Webb, et al. Epilepsy, suicidality, and psychiatric disorders: a bidirectional association. Ann Neurol 2012:72:184-91.

5. Khan NF, AM Ward, E Watson, and PW Rose. Consulting and prescribing behaviour for anxiety and depression in long-term survivors of cancer in the UK. Eur J Cancer 2010:46:3339-44.

6. Kurd SK, AB Troxel, P Crits-Christoph, and JM Gelfand. The risk of depression, anxiety, and suicidality in patients with psoriasis: a population-based cohort study. Arch Dermatol 2010:146:891-5.

7. Lurie I, YX Yang, K Haynes, R Mamtani, et al. Antibiotic exposure and the risk for depression, anxiety, or psychosis: a nested case-control study. J Clin Psychiatry 2015:76:1522-8.

8. Martin-Merino E, A Ruigomez, MA Wallander, S Johansson, et al. Prevalence, incidence, morbidity and treatment patterns in a cohort of patients diagnosed with anxiety in UK primary care. Fam Pract 2010:27:9-16.

9. Meier CR, K Wilcock, and SS Jick. The risk of severe depression, psychosis or panic attacks with prophylactic antimalarials. Drug Saf 2004:27:203-13.

10. Schneider C, M Adamcova, SS Jick, P Schlagenhauf, et al. Antimalarial chemoprophylaxis and the risk of neuropsychiatric disorders. Travel Med Infect Dis 2013:11:71-80.

11. Sheehan R, A Hassiotis, K Walters, D Osborn, et al. Mental illness, challenging behaviour, and psychotropic drug prescribing in people with intellectual disability: UK population based cohort study. BMJ 2015:351:h4326.

12. Walters K, G Rait, M Griffin, M Buszewicz, et al. Recent trends in the incidence of anxiety diagnoses and symptoms in primary care. PLoS One 2012:7:e41670.

13. Becker C, GP Brobert, S Johansson, SS Jick, et al. Risk of incident depression in patients with Parkinson disease in the UK. Eur J Neurol 2011:18:448-53.

14. Booth H, O Khan, AT Prevost, M Reddy, et al. Impact of bariatric surgery on clinical depression. Interrupted time series study with matched controls. J Affect Disord 2015:174:644-9.

15. Bornand D, S Toovey, SS Jick, and CR Meier. The risk of new onset depression in association with influenza--A population-based observational study. Brain Behav Immun 2016:53:131-137.

16. Claxton AJ, Z Li, and J McKendrick. Selective serotonin reuptake inhibitor treatment in the UK: risk of relapse or recurrence of depression. Br J Psychiatry 2000:177:163-8.

17. Clifford GM and RD Farmer. Drug or symptom-induced depression in men treated with alpha 1-blockers for benign prostatic hyperplasia? A nested case-control study. Pharmacoepidemiol Drug Saf 2002:11:55-61.

18. Dave S, I Petersen, L Sherr, and I Nazareth. Incidence of maternal and paternal depression in primary care: a cohort study using a primary care database. Arch Pediatr Adolesc Med 2010:164:1038-44.

19. Gunnell D, D Irvine, L Wise, C Davies, et al. Varenicline and suicidal behaviour: a cohort study based on data from the General Practice Research Database. BMJ 2009:339:b3805.

20. Hagberg KW, HA Divan, JC Nickel, and SS Jick. Risk of Incident Antidepressant-Treated Depression Associated with Use of 5alpha-Reductase Inhibitors Compared with Use of alpha-Blockers in Men with Benign Prostatic Hyperplasia: A Population-Based Study Using the Clinical Practice Research Datalink. Pharmacotherapy 2017:37:517-527.

21. Hagberg KW, L Li, M Peng, K Shah, et al. Incidence rates of suicidal behaviors and treated depression in patients with and without psoriatic arthritis using the Clinical Practice Research Datalink. Mod Rheumatol 2016:26:774-9.

22. Harris T, SM Shah, IM Carey, S DeWilde, et al. Depression indicators in a national sample of older community and care home patients: applying the Quality and Outcomes Framework. Br J Gen Pract 2011:61:135-8.

23. Jacob L, T Rockel, and K Kostev. Depression Risk in Patients with Rheumatoid Arthritis in the United Kingdom. Rheumatol Ther 2017:4:195-200.

24. Jenkins-Jones S, L Parviainen, J Porter, M Withe, et al. Poor compliance and increased mortality, depression and healthcare costs in patients with congenital adrenal hyperplasia. Eur J Endocrinol 2018:178:309-320.

25. John A, AL Marchant, DL Fone, JI McGregor, et al. Recent trends in primary-care antidepressant prescribing to children and young people: an e-cohort study. Psychol Med 2016:46:3315-3327.

26. Kendrick T, B Stuart, C Newell, AW Geraghty, et al. Changes in rates of recorded depression in English primary care 2003-2013: Time trend analyses of effects of the economic recession, and the GP contract quality outcomes framework (QOF). J Affect Disord 2015:180:68-78.

27. Kotz D, W Viechtbauer, CR Simpson, OCP van Schayck, et al. Cardiovascular and neuropsychiatric risks of varenicline and bupropion in smokers with chronic obstructive pulmonary disease. Thorax 2017:72:905-911.

28. Kotz D, W Viechtbauer, C Simpson, OC van Schayck, et al. Cardiovascular and neuropsychiatric risks of varenicline: a retrospective cohort study. Lancet Respir Med 2015:3:761-8.

29. Martin-Merino E, A Ruigomez, S Johansson, MA Wallander, et al. Study of a cohort of patients newly diagnosed with depression in general practice: prevalence, incidence, comorbidity, and treatment patterns. Prim Care Companion J Clin Psychiatry 2010:12:PCC 08m00764.

30. Millson D, M Frischer, P Croft, and PJ Goadsby. Are triptans with enhanced lipophilicity used for the acute treatment of migraine associated with an increased consulting rate for depressive illness? Cephalalgia 2000:20:732-7.

31. Milojevic A, B Armstrong, and P Wilkinson. Mental health impacts of flooding: a controlled interrupted time series analysis of prescribing data in England. J Epidemiol Community Health 2017:71:970-973.

32. Moore M, HM Yuen, N Dunn, MA Mullee, et al. Explaining the rise in antidepressant prescribing: a descriptive study using the general practice research database. BMJ 2009:339:b3999.

33. Morgan E, CC Patterson, and CR Cardwell. General practice-recorded depression and antidepressant use in young people with newly diagnosed Type 1 diabetes: a cohort study using the Clinical Practice Research Datalink. Diabet Med 2014:31:241-5.

34. Petersen I, JM Thomas, WT Hamilton, and PD White. Risk and predictors of fatigue after infectious mononucleosis in a large primary-care cohort. QJM 2006:99:49-55.

35. Rait G, K Walters, M Griffin, M Buszewicz, et al. Recent trends in the incidence of recorded depression in primary care. Br J Psychiatry 2009:195:520-4.

36. Shah SM, IM Carey, T Harris, S DeWilde, et al. The mental health and mortality impact of death of a partner with dementia. Int J Geriatr Psychiatry 2016:31:929-37.

37. Schneider C, SS Jick, U Bothner, and CR Meier. COPD and the risk of depression. Chest 2010:137:341-7.

38. Smeeth L, I Douglas, AJ Hall, R Hubbard, et al. Effect of statins on a wide range of health outcomes: a cohort study validated by comparison with randomized trials. Br J Clin Pharmacol 2009:67:99-109.

39. Smith DJ, H Court, G McLean, D Martin, et al. Depression and multimorbidity: a cross-sectional study of 1,751,841 patients in primary care. J Clin Psychiatry 2014:75:1202-8; quiz 1208.

40. Thomas KH, RM Martin, NM Davies, C Metcalfe, et al. Smoking cessation treatment and risk of depression, suicide, and self harm in the Clinical Practice Research Datalink: prospective cohort study. BMJ 2013:347:f5704.

41. Tyrer F, RA Lawrenson, and RDT Farmer. A study of cardiovascular disease, depression and antidepressants on a computerised general practice database. Human Psychopharmacology: clinical & experimental 1999:14:233-237.

42. Vallerand IA, RT Lewinson, LM Parsons, MW Lowerison, et al. Risk of depression among patients with acne in the U.K.: a population-based cohort study. Br J Dermatol 2018:178:e194-e195.

43. Walters P, P Schofield, L Howard, M Ashworth, et al. The relationship between asthma and depression in primary care patients: a historical cohort and nested case control study. PLoS One 2011:6:e20750.

44. Yang CC, SS Jick, and H Jick. Lipid-lowering drugs and the risk of depression and suicidal behavior. Arch Intern Med 2003:163:1926-32.

45. John A, J McGregor, D Fone, F Dunstan, et al. Case-finding for common mental disorders of anxiety and depression in primary care: an external validation of routinely collected data. BMC Med Inform Decis Mak 2016:16:35.

46. Turner GM, M Calvert, MG Feltham, R Ryan, et al. Ongoing impairments following transient ischaemic attack: retrospective cohort study. Eur J Neurol 2016:23:1642-1650.

47. Brown A, O Kirichek, A Balkwill, G Reeves, et al. Comparison of dementia recorded in routinely collected hospital admission data in England with dementia recorded in primary care. Emerg Themes Epidemiol 2016:13:11.

48. Davies NM, PG Kehoe, Y Ben-Shlomo, and RM Martin. Associations of anti-hypertensive treatments with Alzheimer's disease, vascular dementia, and other dementias. J Alzheimers Dis 2011:26:699-708.

49. Donegan K, N Fox, N Black, G Livingston, et al. Trends in diagnosis and treatment for people with dementia in the UK from 2005 to 2015: a longitudinal retrospective cohort study. Lancet Public Health 2017:2:e149-e156.

50. Dregan A, P Chowienczyk, and MC Gulliford. Are Inflammation and Related Therapy Associated with All-Cause Dementia in a Primary Care Population? J Alzheimers Dis 2015:46:1039-47.

51. Dregan A, P Chowienczyk, and D Armstrong. Patterns of anti-inflammatory drug use and risk of dementia: a matched case-control study. Eur J Neurol 2015:22:1421-8.

52. Dunn N, C Holmes, and M Mullee. Does lithium therapy protect against the onset of dementia? Alzheimer Dis Assoc Disord 2005:19:20-2.

53. Dunn N, M Mullee, VH Perry, and C Holmes. Association between dementia and infectious disease: evidence from a case-control study. Alzheimer Dis Assoc Disord 2005:19:91-4.

54. Emdin CA, PM Rothwell, G Salimi-Khorshidi, A Kiran, et al. Blood Pressure and Risk of Vascular Dementia: Evidence From a Primary Care Registry and a Cohort Study of Transient Ischemic Attack and Stroke. Stroke 2016:47:1429-35.

55. Goh KL, K Bhaskaran, C Minassian, SJ Evans, et al. Angiotensin receptor blockers and risk of dementia: cohort study in UK Clinical Practice Research Datalink. Br J Clin Pharmacol 2015:79:337-50.

56. Hippisley-Cox J and C Coupland. Unintended effects of statins in men and women in England and Wales: population based cohort study using the QResearch database. BMJ 2010:340:c2197.

57. Imfeld P, M Bodmer, SS Jick, and CR Meier. Metformin, other antidiabetic drugs, and risk of Alzheimer's disease: a population-based case-control study. J Am Geriatr Soc 2012:60:916-21.

58. Imfeld P, YB Brauchli Pernus, SS Jick, and CR Meier. Epidemiology, co-morbidities, and medication use of patients with Alzheimer's disease or vascular dementia in the UK. J Alzheimers Dis 2013:35:565-73.

59. Imfeld P, M Bodmer, SS Jick, and CR Meier. Benzodiazepine Use and Risk of Developing Alzheimer's Disease or Vascular Dementia: A Case-Control Analysis. Drug Saf 2015:38:909-19.

60. Jick H, GL Zornberg, SS Jick, S Seshadri, et al. Statins and the risk of dementia. Lancet 2000:356:1627-31.

61. Judge A, C Garriga, NK Arden, S Lovestone, et al. Protective effect of antirheumatic drugs on dementia in rheumatoid arthritis patients. Alzheimers Dement (N Y) 2017:3:612-621.

62. Khan NF, D Mant, L Carpenter, D Forman, et al. Long-term health outcomes in a British cohort of breast, colorectal and prostate cancer survivors: a database study. Br J Cancer 2011:105 Suppl 1:S29-37.

63. Khosrow-Khavar F, S Rej, H Yin, A Aprikian, et al. Androgen Deprivation Therapy and the Risk of Dementia in Patients With Prostate Cancer. J Clin Oncol 2017:35:201-207.

64. Lu N, M Dubreuil, Y Zhang, T Neogi, et al. Gout and the risk of Alzheimer's disease: a population-based, BMI-matched cohort study. Ann Rheum Dis 2016:75:547-51.

65. Mehta HB, V Mehta, and JS Goodwin. Association of Hypoglycemia With Subsequent Dementia in Older Patients With Type 2 Diabetes Mellitus. J Gerontol A Biol Sci Med Sci 2017:72:1110-1116.

66. Mehta HB, V Mehta, CL Tsai, H Chen, et al. Development and Validation of the RxDx-Dementia Risk Index to Predict Dementia in Patients with Type 2 Diabetes and Hypertension. J Alzheimers Dis 2016:49:423-32.

67. Perera G, L Pedersen, D Ansel, M Alexander, et al. Dementia prevalence and incidence in a federation of European Electronic Health Record databases: The European Medical Informatics Framework resource. Alzheimers Dement 2018:14:130-139.

68. Qizilbash N, J Gregson, ME Johnson, N Pearce, et al. BMI and risk of dementia in two million people over two decades: a retrospective cohort study. Lancet Diabetes Endocrinol 2015:3:431-436.

69. Seshadri S, GL Zornberg, LE Derby, MW Myers, et al. Postmenopausal estrogen replacement therapy and the risk of Alzheimer disease. Arch Neurol 2001:58:435-40.

70. Strom BL, R Schinnar, J Karlawish, S Hennessy, et al. Statin Therapy and Risk of Acute Memory Impairment. JAMA Intern Med 2015:175:1399-405.

71. Walters K, S Hardoon, I Petersen, S Iliffe, et al. Predicting dementia risk in primary care: development and validation of the Dementia Risk Score using routinely collected data. BMC Med 2016:14:6.

72. Donegan K, R Beau-Lejdstrom, B King, S Seabroke, et al. Bivalent human papillomavirus vaccine and the risk of fatigue syndromes in girls in the UK. Vaccine 2013:31:4961-7.

73. Collin SM, IJ Bakken, I Nazareth, E Crawley, et al. Trends in the incidence of chronic fatigue syndrome and fibromyalgia in the UK, 2001-2013: a Clinical Practice Research Datalink study. J R Soc Med 2017:110:231-244.

74. Gallagher AM, JM Thomas, WT Hamilton, and PD White. Incidence of fatigue symptoms and diagnoses presenting in UK primary care from 1990 to 2001. J R Soc Med 2004:97:571-5.

75. Hamilton WT, AM Gallagher, JM Thomas, and PD White. Risk markers for both chronic fatigue and irritable bowel syndromes: a prospective case-control study in primary care. Psychol Med 2009:39:1913-21.

76. Becker C, GP Brobert, PM Almqvist, S Johansson, et al. Migraine incidence, comorbidity and health resource utilization in the UK. Cephalalgia 2008:28:57-64.

77. Campbell P, M Shraim, KP Jordan, and KM Dunn. In sickness and in health: A cross-sectional analysis of concordance for musculoskeletal pain in 13,507 couples. Eur J Pain 2016:20:438-46.

78. Hall GC, D Carroll, D Parry, and HJ McQuay. Epidemiology and treatment of neuropathic pain: the UK primary care perspective. Pain 2006:122:156-62.

79. Hall GC, SV Morant, D Carroll, ZL Gabriel, et al. An observational descriptive study of the epidemiology and treatment of neuropathic pain in a UK general population. BMC Fam Pract 2013:14:28.

80. Mansfield KE, J Sim, P Croft, and KP Jordan. Identifying patients with chronic widespread pain in primary care. Pain 2017:158:110-119.

81. Ruigomez A, LA Rodriguez, MA Wallander, S Johansson, et al. Chest pain in general practice: incidence, comorbidity and mortality. Fam Pract 2006:23:167-74.

82. Wallander MA, S Johansson, A Ruigomez, and LA Garcia Rodriguez. Unspecified abdominal pain in primary care: the role of gastrointestinal morbidity. Int J Clin Pract 2007:61:1663-70.

83. Zondervan KT, PL Yudkin, MP Vessey, MG Dawes, et al. Prevalence and incidence of chronic pelvic pain in primary care: evidence from a national general practice database. Br J Obstet Gynaecol 1999:106:1149-55.

84. Blumentals WA, RR Brown, and A Gomez-Caminero. Antihypertensive treatment and erectile dysfunction in a cohort of type II diabetes patients. Int J Impot Res 2003:15:314-7.

85. Hagberg KW, HA Divan, R Persson, JC Nickel, et al. Risk of erectile dysfunction associated with use of 5-alpha reductase inhibitors for benign prostatic hyperplasia or alopecia: population based studies using the Clinical Practice Research Datalink. BMJ 2016:354:i4823.

86. Morant S, G Bloomfield, V Vats, and C Chapple. Increased sexual dysfunction in men with storage and voiding lower urinary tract symptoms. J Sex Med 2009:6:1103-1110.

87. Schlesinger N, N Lu, and HK Choi. Gout and the Risk of Incident Erectile Dysfunction: A Body Mass Index-matched Population-based Study. J Rheumatol 2018:45:1192-1197.

88. Abdul Sultan A, C Mallen, R Hayward, S Muller, et al. Gout and subsequent erectile dysfunction: a population-based cohort study from England. Arthritis Res Ther 2017:19:123.

89. Roddy E, S Muller, R Hayward, and CD Mallen. The association of gout with sleep disorders: a cross-sectional study in primary care. BMC Musculoskelet Disord 2013:14:119.

90. Wallander MA, S Johansson, A Ruigomez, LA Garcia Rodriguez, et al. Morbidity associated with sleep disorders in primary care: a longitudinal cohort study. Prim Care Companion J Clin Psychiatry 2007:9:338-45.

91. Andersohn F, R Schade, SN Willich, and E Garbe. Use of antiepileptic drugs in epilepsy and the risk of self-harm or suicidal behavior. Neurology 2010:75:335-40.

92. Arana A, CE Wentworth, JL Ayuso-Mateos, and FM Arellano. Suicide-related events in patients treated with antiepileptic drugs. N Engl J Med 2010:363:542-51.

93. Carr MJ, DM Ashcroft, E Kontopantelis, Y Awenat, et al. The epidemiology of self-harm in a UK-wide primary care patient cohort, 2001-2013. BMC Psychiatry 2016:16:53.

94. Carr MJ, DM Ashcroft, E Kontopantelis, D While, et al. Premature Death Among Primary Care Patients With a History of Self-Harm. Ann Fam Med 2017:15:246-254.

95. Coupland C, P Dhiman, R Morriss, A Arthur, et al. Antidepressant use and risk of adverse outcomes in older people: population based cohort study. BMJ 2011:343:d4551.

96. Coupland C, T Hill, R Morriss, A Arthur, et al. Antidepressant use and risk of suicide and attempted suicide or self harm in people aged 20 to 64: cohort study using a primary care database. BMJ 2015:350:h517.

97. Donovan S. The Use of the General Practice Research Database (Gprd) to Examine Potential Links between Antidepressant Medication and the Incidence of Suicide. Therapeutic Innovation & Regulatory Science 1996:30:785-792.

98. Doyle M, D While, PL Mok, K Windfuhr, et al. Suicide risk in primary care patients diagnosed with a personality disorder: a nested case control study. BMC Fam Pract 2016:17:106.

99. Gao S, J Juhaeri, S Reshef, and WS Dai. Association between body mass index and suicide, and suicide attempt among British adults: the health improvement network database. Obesity (Silver Spring) 2013:21:E334-42.

100. Hall GC. Validation of death and suicide recording on the THIN UK primary care database. Pharmacoepidemiol Drug Saf 2009:18:120-31.

101. Hayes JF, A Pitman, L Marston, K Walters, et al. Self-harm, Unintentional Injury, and Suicide in Bipolar Disorder During Maintenance Mood Stabilizer Treatment: A UK Population-Based Electronic Health Records Study. JAMA Psychiatry 2016:73:630-7.

102. Hesdorffer DC, L Ishihara, DJ Webb, L Mynepalli, et al. Occurrence and Recurrence of Attempted Suicide Among People With Epilepsy. JAMA Psychiatry 2016:73:80-6.

103. Haste F, J Charlton, and R Jenkins. Potential for suicide prevention in primary care? An analysis of factors associated with suicide. Br J Gen Pract 1998:48:1759-63.

104. Jick SS, AD Dean, and H Jick. Antidepressants and suicide. BMJ 1995:310:215-8.

105. Jick SS, C Vasilakis, C Martinez, and H Jick. A study of the relation of exposure to quinolones and suicidal behaviour. Br J Clin Pharmacol 1998:45:77-81.

106. Jick SS, HM Kremers, and C Vasilakis-Scaramozza. Isotretinoin use and risk of depression, psychotic symptoms, suicide, and attempted suicide. Arch Dermatol 2000:136:1231-6.

107. Jick H, JA Kaye, and SS Jick. Antidepressants and the risk of suicidal behaviors. JAMA 2004:292:338-43.

108. Jick H, KW Hagberg, and P Egger. Rate of suicide in patients taking montelukast. Pharmacotherapy 2009:29:165-6.

109. Lalmohamed A, MT Bazelier, TP Van Staa, BM Uitdehaag, et al. Causes of death in patients with multiple sclerosis and matched referent subjects: a population-based cohort study. Eur J Neurol 2012:19:1007-14.

110. Martinez C, S Rietbrock, L Wise, D Ashby, et al. Antidepressant treatment and the risk of fatal and non-fatal self harm in first episode depression: nested case-control study. BMJ 2005:330:389.

111. Mines D, D Hill, H Yu, and L Novelli. Prevalence of risk factors for suicide in patients prescribed venlafaxine, fluoxetine, and citalopram. Pharmacoepidemiol Drug Saf 2005:14:367-72.

112. Osborn D, G Levy, I Nazareth, and M King. Suicide and severe mental illnesses. Cohort study within the UK general practice research database. Schizophr Res 2008:99:134-8.

113. Rubino A, N Roskell, P Tennis, D Mines, et al. Risk of suicide during treatment with venlafaxine, citalopram, fluoxetine, and dothiepin: retrospective cohort study. BMJ 2007:334:242.

114. Schuerch M, C Gasse, NJ Robinson, Y Alvarez, et al. Impact of varying outcomes and definitions of suicidality on the associations of antiepileptic drugs and suicidality: comparisons from UK Clinical Practice Research Datalink (CPRD) and Danish national registries (DNR). Pharmacoepidemiol Drug Saf 2016:25 Suppl 1:142-55.

115. Thomas KH, N Davies, C Metcalfe, F Windmeijer, et al. Validation of suicide and self-harm records in the Clinical Practice Research Datalink. Br J Clin Pharmacol 2013:76:145-57.

116. Tyrrell EG, E Orton, and LJ Tata. Changes in poisonings among adolescents in the UK between 1992 and 2012: a population based cohort study. Inj Prev 2016:22:400-406.

117. Webb RT, E Kontopantelis, T Doran, P Qin, et al. Risk of self-harm in physically ill patients in UK primary care. J Psychosom Res 2012:73:92-7.

118. Webb RT, E Kontopantelis, T Doran, P Qin, et al. Suicide risk in primary care patients with major physical diseases: a case-control study. Arch Gen Psychiatry 2012:69:256-64.

119. Wijlaars LP, I Nazareth, HJ Whitaker, SJ Evans, et al. Suicide-related events in young people following prescription of SSRIs and other antidepressants: a self-controlled case series analysis. BMJ Open 2013:3:e003247.

120. Windfuhr K, D While, N Kapur, DM Ashcroft, et al. Suicide risk linked with clinical consultation frequency, psychiatric diagnoses and psychotropic medication prescribing in a national study of primary-care patients. Psychol Med 2016:46:3407-3417.
