# Supplementary material for: Identification of mental health and quality of life outcomes in primary care databases in the UK: a systematic review
Source: BMJ Open. 2019 Jul 2;9(7):e029227. doi: 10.1136/bmjopen-2019-029227 (PMC6609128; doi:10.1136/bmjopen-2019-029227)
Supplement: Supplementary data [file bmjopen-2019-029227supp004.pdf]

Table 1. List of Read codes used in the studies of anxiety.

Read code	Description	Number of studies
Eu41.00	[X]Other anxiety disorders	5
Eu41100	[X]Generalized anxiety disorder	5
Eu41z11	[X]Anxiety NOS	5
Eu41000	[X]Panic disorder [episodic paroxysmal anxiety]	4
Eu05400	[X]Organic anxiety disorder	4
Eu41112	[X]Anxiety reaction	4
Eu41111	[X]Anxiety neurosis	4
Eu41z00	[X]Anxiety disorder, unspecified	4
E202.12	Phobic anxiety	4
E200200	Generalised anxiety disorder	4
E200.00	Anxiety states	4
E200000	Anxiety state unspecified	4
E200z00	Anxiety state NOS	4
Eu40.00	[X]Phobic anxiety disorders	3
Eu40z00	[X]Phobic anxiety disorder, unspecified	3
Eu41012	[X]Panic state	3
Eu41011	[X]Panic attack	3
Eu41y00	[X]Other specified anxiety disorders	3
Eu41300	[X]Other mixed anxiety disorders	3
Eu41211	[X]Mild anxiety depression	3
Eu41113	[X]Anxiety state	3
E200500	Recurrent anxiety	3
E200100	Panic disorder	3
E200111	Panic attack	3
E200400	Chronic anxiety	3
1B1V.00	C/O - panic attack	3
1B13.11	Anxiousness - symptom	3
1B13.00	Anxiousness	3
E200300	Anxiety with depression	3
Eu93200	[X]Social anxiety disorder of childhood	2
Eu34114	[X]Persistant anxiety depression	2
Eu40012	[X]Panic disorder with agoraphobia	2
Eu40y00	[X]Other phobic anxiety disorders	2
Eu41200	[X]Mixed anxiety and depressive disorder	2
Eu93y12	[X]Childhood overanxious disorder	2
Eu41y11	[X]Anxiety hysteria	2
E2D0.00	Disturbance of anxiety and fearfulness childhood/adolescent	2
E2D0z00	Disturbance anxiety and fearfulness childhood/adolescent NOS	2
E202100	Agoraphobia with panic attacks	2
E292400	Adjustment reaction with anxious mood	2
E280.00	Acute panic state due to acute stress reaction	2
Z4I7.00	Acknowledging anxiety	2
Eu40100	[X]Social phobias	1
Eu40112	[X]Social neurosis	1
Eu93000	[X]Separation anxiety disorder of childhood	1
Eu42100	[X]Predominantly compulsive acts [obsessional rituals]	1
Eu93100	[X]Phobic anxiety disorder of childhood	1
Eu42.12	[X]Obsessive-compulsive neurosis	1
Eu42.00	[X]Obsessive - compulsive disorder	1
Eu45215	[X]Nosophobia	1
Eu46z11	[X]Neurosis NOS	1

Read code	Description	Number of studies
Eu40300	[X]Needle phobia	1
Eu45213	[X]Hypochondriacal neurosis	1
Eu45212	[X]Dysmorphophobia nondelusional	1
Eu51511	[X]Dream anxiety disorder	1
Eu40213	[X]Claustrophobia	1
Eu60600	[X]Anxious [avoidant] personality disorder	1
Eu42.11	[X]Anankastic neurosis	1
Eu40011	[X]Agoraphobia without history of panic disorder	1
Eu40000	[X]Agoraphobia	1
R2y2.00	[D]Nervousness	1
R2y2.12	[D]Nervous tension	1
R2y2.11	[D]Nerves	1
1BK..00	Worried	1
1B12.12	Tension - nervous	1
E202300	Social phobia, fear of eating in public	1
E112000	Single major depressive episode, unspecified	1
E292000	Separation anxiety disorder	1
Z4I7211	Reducing anxiety	1
Z4I7100	Recognising anxiety	1
E29y100	Other post-traumatic stress disorder	1
E203z00	Obsessive-compulsive disorder NOS	1
E203100	Obsessional neurosis	1
225J.00	O/E - panic attack	1
2258.00	O/E - anxious	1
1465.00	H/O: depression	1
1466.00	H/O: anxiety state	1
1B1..00	General nervous symptoms	1
E275711	Compulsive water drinking	1
E203000	Compulsive neurosis	1
1P3..00	Compulsive behaviour	1
E202800	Claustrophobia	1
E2D0000	Childhood and adolescent overanxiousness disturbance	1
1B13.12	Anxious	1
8G94.00	Anxiety management training	1
Z4L1.00	Anxiety counselling	1
E203.11	Anancastic neurosis	1
Z4I7200	Alleviating anxiety	1
E202200	Agoraphobia without mention of panic attacks	1
1B12.00	'Nerves' - nervousness	1
1B12.11	'Nerves'	1

Table 2. ICD codes used in the studies of anxiety.

Study	Databases	ICD version	List of codes
Bouras, 2016	CPRD + HES	ICD-10	F40-F48

CPRD – Clinical Practice Research Datalink; HES – Hospital Episode Statistics; ICD - International Classification of Diseases.

Table 3. List of Read codes used in the studies of depression.

Read code	Description	Number of studies
E112.00	Single major depressive episode	14
E112000	Single major depressive episode, unspecified	14
E112100	Single major depressive episode, mild	14
E112200	Single major depressive episode, moderate	14
E112300	Single major depressive episode, severe, without psychosis	14
E112z00	Single major depressive episode NOS	14
E135.00	Agitated depression	14
E112.11	Agitated depression	13
E112.12	Endogenous depression first episode	13
E112.13	Endogenous depression first episode	13
E112.14	Endogenous depression	13
E112500	Single major depressive episode, partial or unspec remission	13
E2B..00	Depressive disorder NEC	13
E2B1.00	Chronic depression	13
Eu32.00	[X]Depressive episode	13
E113.00	Recurrent major depressive episode	12
E11y200	Atypical depressive disorder	12
E11z200	Masked depression	12
Eu32.11	[X]Single episode of depressive reaction	12
Eu32.12	[X]Single episode of psychogenic depression	12
Eu32.13	[X]Single episode of reactive depression	12
Eu32000	[X]Mild depressive episode	12
Eu32100	[X]Moderate depressive episode	12
Eu32200	[X]Severe depressive episode without psychotic symptoms	12
Eu32400	[X]Mild depression	12
Eu32y00	[X]Other depressive episodes	12
Eu32z00	[X]Depressive episode, unspecified	12
Eu33.00	[X]Recurrent depressive disorder	12
E112400	Single major depressive episode, severe, with psychosis	11
E113.11	Endogenous depression - recurrent	11
E130.11	Psychotic reactive depression	11
Eu32212	[X]Single episode major depression w'out psychotic symptoms	11
Eu32y11	[X]Atypical depression	11
Eu32z11	[X]Depression NOS	11
Eu32z12	[X]Depressive disorder NOS	11
1B17.00	Depressed	10
E112600	Single major depressive episode, in full remission	10
E113000	Recurrent major depressive episodes, unspecified	10
E113100	Recurrent major depressive episodes, mild	10
E113200	Recurrent major depressive episodes, moderate	10
E113300	Recurrent major depressive episodes, severe, no psychosis	10
E113500	Recurrent major depressive episodes,partial/unspec remission	10
E113700	Recurrent depression	10
E113z00	Recurrent major depressive episode NOS	10
E130.00	Reactive depressive psychosis	10
E200300	Anxiety with depression	10
E291.00	Prolonged depressive reaction	10
Eu32211	[X]Single episode agitated depressn w'out psychotic symptoms	10
Eu32213	[X]Single episode vital depression w'out psychotic symptoms	10
Eu32311	[X]Single episode of major depression and psychotic symptoms	10
Eu32313	[X]Single episode of psychotic depression	10

Read code	Description	Number of studies
Eu32y12	[X]Single episode of masked depression NOS	10
Eu32z13	[X]Prolonged single episode of reactive depression	10
Eu33.11	[X]Recurrent episodes of depressive reaction	10
Eu33.12	[X]Recurrent episodes of psychogenic depression	10
Eu33.13	[X]Recurrent episodes of reactive depression	10
Eu33000	[X]Recurrent depressive disorder, current episode mild	10
Eu33100	[X]Recurrent depressive disorder, current episode moderate	10
Eu33211	[X]Endogenous depression without psychotic symptoms	10
Eu33y00	[X]Other recurrent depressive disorders	10
Eu33z00	[X]Recurrent depressive disorder, unspecified	10
Eu34114	[X]Persistant anxiety depression	10
1B1U.00	Symptoms of depression	9
1B1U.11	Depressive symptoms	9
E113600	Recurrent major depressive episodes, in full remission	9
Eu32300	[X]Severe depressive episode with psychotic symptoms	9
Eu32312	[X]Single episode of psychogenic depressive psychosis	9
Eu32314	[X]Single episode of reactive depressive psychosis	9
Eu32500	[X]Major depression, mild	9
Eu32600	[X]Major depression, moderately severe	9
Eu32700	[X]Major depression, severe without psychotic symptoms	9
Eu32z14	[X] Reactive depression NOS	9
Eu33214	[X]Vital depression, recurrent without psychotic symptoms	9
Eu33311	[X]Endogenous depression with psychotic symptoms	9
Eu33z11	[X]Monopolar depression NOS	9
Eu34113	[X]Neurotic depression	9
Eu41200	[X]Mixed anxiety and depressive disorder	9
Eu41211	[X]Mild anxiety depression	9
1B17.11	C/O - feeling depressed	8
E113400	Recurrent major depressive episodes, severe, with psychosis	8
E204.00	Neurotic depression reactive type	8
Eu33200	[X]Recurr depress disorder cur epi severe without psyc sympt	8
Eu33212	[X]Major depression, recurrent without psychotic symptoms	8
Eu33400	[X]Recurrent depressive disorder, currently in remission	8
Eu34111	[X]Depressive neurosis	8
Eu3y111	[X]Recurrent brief depressive episodes	8
1BT..00	Depressed mood	7
2257.00	O/E - depressed	7
E11..12	Depressive psychoses	7
Eu32800	[X]Major depression, severe with psychotic symptoms	7
Eu33.14	[X]Seasonal depressive disorder	7
Eu33313	[X]Recurr severe episodes/major depression+psychotic symptom	7
Eu33314	[X]Recurr severe episodes/psychogenic depressive psychosis	7
Eu33315	[X]Recurrent severe episodes of psychotic depression	7
Eu33316	[X]Recurrent severe episodes/reactive depressive psychosis	7
9H90.00	Depression annual review	6
9H91.00	Depression medication review	6
9H92.00	Depression interim review	6
9HA0.00	On depression register	6
E290.00	Brief depressive reaction	6
Eu33300	[X]Recurrent depress disorder cur epi severe with psyc symp	6
Eu34100	[X]Dysthymia	6
E118.00	Seasonal affective disorder	5
E204.11	Postnatal depression	5

Read code	Description	Number of studies
E290z00	Brief depressive reaction NOS	5
Eu53011	[X]Postnatal depression NOS	5
Eu53012	[X]Postpartum depression NOS	5
1465.00	H/O: depression	4
1BQ..00	Loss of capacity for enjoyment	4
8HHq.00	Referral for guided self-help for depression	4
E02y300	Drug-induced depressive state	4
Eu33.15	[X]SAD - Seasonal affective disorder	4
R007z13	[D]Postoperative depression	4
1BP0.00	Loss of interest in previously enjoyable activity	3
1BT..11	Low mood	3
1BU..00	Loss of hope for the future	3
212S.00	Depression resolved	3
8CAa.00	Patient given advice about management of depression	3
9Ov0.00	Depression monitoring first letter	3
9Ov1.00	Depression monitoring second letter	3
9Ov2.00	Depression monitoring third letter	3
9Ov3.00	Depression monitoring verbal invite	3
9Ov4.00	Depression monitoring telephone invite	3
9k4..00	Depression - enhanced services administration	3
E001300	Presenile dementia with depression	3
E002100	Senile dementia with depression	3
E2B0.00	Postviral depression	3
Eu20400	[X]Post-schizophrenic depression	3
Eu34112	[X]Depressive personality disorder	3
Eu92000	[X]Depressive conduct disorder	3
1JJ..00	Suspected depression	2
8BK0.00	Depression management programme	2
9HA1.00	Removed from depression register	2
9Ov..00	Depression monitoring administration	2
9k40.00	Depression - enhanced service completed	2
9kQ..00	On full dose long term treatment depression - enh serv admin	2
E115.00	Bipolar affective disorder, currently depressed	2
E11y.00	Other and unspecified manic-depressive psychoses	2
E211200	Depressive personality disorder	2
Eu25100	[X]Schizoaffective disorder, depressive type	2
Eu25111	[X]Schizoaffective psychosis, depressive type	2
Eu25112	[X]Schizophreniform psychosis, depressive type	2
Eu32B00	[X]Antenatal depression	2
Eu33213	[X]Manic-depress psychosis,depressd,no psychotic symptoms	2
Eu34y00	[X]Other persistent mood affective disorders	2
Eu34z00	[X]Persistent mood affective disorder, unspecified	2
Eu3y100	[X]Other recurrent mood affective disorders	2
Eu3z.00	[X]Unspecified mood affective disorder	2
1645.00	Excessive fluid intake	1
1B1J.00	Emotional problem	1
1B1J.11	Emotional upset	1
1BO..00	Mood swings	1
1BP..00	Loss of interest	1
1BT..12	Sad mood	1
1S4..00	Mood observations	1
1S40.00	Dysphoric mood	1
388J.00	Hospital anxiety and depression scale	1

Read code	Description	Number of studies
388K.00	Geriatric depression scale	1
388P.00	HAD scale: depression score	1
388g.00	Beck depression inventory second edition score	1
62T1.00	Puerperal depression	1
6896.00	Depression screening using questions	1
8O82.00	Emotional and psychosocial support and advice	1
9ON3.00	Stress monitoring default	1
9ON4.00	Stress monitoring 1st letter	1
9ON5.00	Stress monitoring 2nd letter	1
9ON6.00	Stress monitoring 3rd letter	1
9ON7.00	Stress monitoring verbal inv.	1
9ON8.00	Stress monitoring phone invite	1
9ON9.00	Stress monitoring deleted	1
9ONA.00	Stress monitoring check done	1
9ONZ.00	Stress monitoring admin.NOS	1
E002.00	Senile dementia with depressive or paranoid features	1
E002z00	Senile dementia with depressive or paranoid features NOS	1
E004300	Arteriosclerotic dementia with depression	1
E115.11	Manic-depressive - now depressed	1
E115000	Bipolar affective disorder, currently depressed, unspecified	1
E115100	Bipolar affective disorder, currently depressed, mild	1
E115200	Bipolar affective disorder, currently depressed, moderate	1
E115300	Bipolar affect disord, now depressed, severe, no psychosis	1
E115400	Bipolar affect disord, now depressed, severe with psychosis	1
E115500	Bipolar affect disord, now depressed, part/unspec remission	1
E115600	Bipolar affective disorder, now depressed, in full remission	1
E115z00	Bipolar affective disorder, currently depressed, NOS	1
E11y000	Unspecified manic-depressive psychoses	1
E11z100	Rebound mood swings	1
E222		1
E283.00	Other acute stress reactions	1
E283z00	Other acute stress reaction NOS	1
E284.00	Stress reaction causing mixed disturbance of emotion/conduct	1
E28z.00	Acute stress reaction NOS	1
E292.00	Adjustment reaction, predominant disturbance other emotions	1
E292400	Adjustment reaction with anxious mood	1
E292y00	Adjustment reaction with mixed disturbance of emotion	1
E292z00	Adjustment reaction with disturbance of other emotion NOS	1
E294.00	Adjustment reaction with disturbance emotion and conduct	1
E2C4.00	Mixed disturbance of conduct and emotion	1
E2C4z00	Mixed disturbance of conduct and emotion NOS	1
E35		1
E4J5		1
Eu02z16	[X] Senile dementia, depressed or paranoid type	1
Eu3..00	[X]Mood - affective disorders	1
Eu31.11	[X]Manic-depressive illness	1
Eu31300	[X]Bipolar affect disorder cur epi mild or moderate depressn	1
Eu31400	[X]Bipol aff disord, curr epis sev depress, no psychot symp	1
Eu31500	[X]Bipolar affect dis cur epi severe depres with psyc symp	1
Eu31600	[X]Bipolar affective disorder, current episode mixed	1
Eu31y00	[X]Other bipolar affective disorders	1
Eu31y11	[X]Bipolar II disorder	1
Eu31z00	[X]Bipolar affective disorder, unspecified	1

Read code	Description	Number of studies
Eu33312	[X]Manic-depress psychosis,depressed type+psychotic symptoms	1
Eu34.00	[X]Persistent mood affective disorders	1
Eu3y.00	[X]Other mood affective disorders	1
Eu3y000	[X]Other single mood affective disorders	1
Eu3y011	[X]Mixed affective episode	1
Eu3yy00	[X]Other specified mood affective disorders	1
Eu4..00	[X]Neurotic, stress - related and somoform disorders	1
Eu43.00	[X]Reaction to severe stress, and adjustment disorders	1
Eu43000	[X]Acute stress reaction	1
Eu43012	[X]Acute reaction to stress	1
Eu43y00	[X]Other reactions to severe stress	1
Eu43z00	[X]Reaction to severe stress, unspecified	1
Eu92.11	[X]Emotional behavioural problems	1
R007z14	[D]Work stress	1
ZR2A.00	Beck depression inventory	1
ZR2A.11	BDI - Beck depression inventory	1
ZR2B.00	Beck hopelessness scale	1
ZR2G.00	Behaviour and mood disturbance scale	1
ZR2h.00	Brief depression rating scale	1
ZR7..00	Depression anxiety scale	1
ZR8..00	Depression self rating scale	1
ZR8..11	DSRS - Depression self rating scale	1
ZRBY.00	Edinburgh postnatal depression scale	1
ZRBY.11	EPDS - Edinburgh postnatal depression scale	1
ZRL6.00	Geriatric depression scale	1
ZRL6.11	GDS - Geriatric depression scale	1
ZRL6.12	Geriatric depression score	1
ZRLU.00	Hamilton rating scale for depression	1
ZRLU.11	HAMD - Hamilton rating scale for depression	1
ZRLU.12	HRSD - Hamilton rating scale for depression	1
ZRLfH00	Health of the Nation Outcome Scale item 7 - depressed mood	1
ZRLfI00	Health of the Nation Outcome Scale item 7 - depressed mood	1
ZRLn.00	Hopelessness scale	1
ZRLr.00	Hospital anxiety and depression scale	1
ZRLr.11	HAD - Hospital anxiety and depression scale	1
ZRLr.12	HADS - Hospital anxiety and depression scale	1
ZRVM.00	Leeds scale for the self-assessment of anxiety & depression	1
ZRaH.00	Mood affective checklist	1
ZRaH.11	MACL - Mood affective checklist	1
ZRbS.00	Positive and negative affect schedule	1
ZRby.00	Profile of mood states	1
ZRby.11	POMS - Profile of mood states	1
ZRrI.00	Wakefield self-assessment depression inventory	1
ZRrY.00	WHO depression scale	1
ZRrc.00	Zung self-rating depression scale	1
ZRrc.11	SDS - Zung self-rating depression scale	1
ZV11100	[V]Personal history of affective disorder	1
ZV11111	[V]Personal history of manic-depressive psychosis	1
ZV11112	[V]Personal history of manic-depressive psychosis	1

Table 4. ICD codes used in the studies of depression.

Study	Databases	ICD version	List of codes
Bouras, 2016	CPRD + HES	ICD-10	F32, F33
Jenkins-Jones, 2018	CPRD + HES	ICD-10	F32, F33, F41.2, F92.0

CPRD – Clinical Practice Research Datalink; HES – Hospital Episode Statistics; ICD - International Classification of Diseases.

Table 5. List of Read codes used in the studies of composite outcomes of anxiety and depression.

Read code	Description	Number of studies
Eu32200	[X]Severe depressive episode without psychotic symptoms	2
Eu33z00	[X]Recurrent depressive disorder, unspecified	2
Eu33400	[X]Recurrent depressive disorder, currently in remission	2
Eu33100	[X]Recurrent depressive disorder, current episode moderate	2
Eu33000	[X]Recurrent depressive disorder, current episode mild	2
Eu33.00	[X]Recurrent depressive disorder	2
Eu33200	[X]Recurr depress disorder cur epi severe without psyc sympt	2
Eu41000	[X]Panic disorder [episodic paroxysmal anxiety]	2
Eu41y00	[X]Other specified anxiety disorders	2
Eu33y00	[X]Other recurrent depressive disorders	2
Eu32y00	[X]Other depressive episodes	2
Eu41.00	[X]Other anxiety disorders	2
Eu32100	[X]Moderate depressive episode	2
Eu41200	[X]Mixed anxiety and depressive disorder	2
Eu32000	[X]Mild depressive episode	2
Eu32400	[X]Mild depression	2
Eu41100	[X]Generalized anxiety disorder	2
Eu34100	[X]Dysthymia	2
Eu32z00	[X]Depressive episode, unspecified	2
Eu32.00	[X]Depressive episode	2
Eu41z00	[X]Anxiety disorder, unspecified	2
1B1U.00	Symptoms of depression	2
E112000	Single major depressive episode, unspecified	2
E112300	Single major depressive episode, severe, without psychosis	2
E112500	Single major depressive episode, partial or unspec remission	2
E112200	Single major depressive episode, moderate	2
E112100	Single major depressive episode, mild	2
E112600	Single major depressive episode, in full remission	2
E112z00	Single major depressive episode NOS	2
E112.00	Single major depressive episode	2
E118.00	Seasonal affective disorder	2
E113500	Recurrent major depressive episodes,partial/unspec remission	2
E113000	Recurrent major depressive episodes, unspecified	2
E113300	Recurrent major depressive episodes, severe, no psychosis	2
E113200	Recurrent major depressive episodes, moderate	2
E113100	Recurrent major depressive episodes, mild	2
E113600	Recurrent major depressive episodes, in full remission	2
E113z00	Recurrent major depressive episode NOS	2
E113.00	Recurrent major depressive episode	2
E113700	Recurrent depression	2
E200500	Recurrent anxiety	2
E291.00	Prolonged depressive reaction	2
E2B0.00	Postviral depression	2
E200100	Panic disorder	2
2257.00	O/E - depressed	2
E204.00	Neurotic depression reactive type	2

Read code	Description	Number of studies
E200200	Generalised anxiety disorder	2
E2B..00	Depressive disorder NEC	2
1BT..00	Depressed mood	2
1B17.00	Depressed	2
E2B1.00	Chronic depression	2
E200400	Chronic anxiety	2
E200300	Anxiety with depression	2
E200.00	Anxiety states	2
E200000	Anxiety state unspecified	2
E200z00	Anxiety state NOS	2
E135.00	Agitated depression	2
Eu33214	[X]Vital depression, recurrent without psychotic symptoms	1
Eu3z.00	[X]Unspecified mood affective disorder	1
Eu40100	[X]Social phobias	1
Eu40112	[X]Social neurosis	1
Eu32213	[X]Single episode vital depression w'out psychotic symptoms	1
Eu32314	[X]Single episode of reactive depressive psychosis	1
Eu32.13	[X]Single episode of reactive depression	1
Eu32313	[X]Single episode of psychotic depression	1
Eu32312	[X]Single episode of psychogenic depressive psychosis	1
Eu32.12	[X]Single episode of psychogenic depression	1
Eu32y12	[X]Single episode of masked depression NOS	1
Eu32311	[X]Single episode of major depression and psychotic symptoms	1
Eu32.11	[X]Single episode of depressive reaction	1
Eu32212	[X]Single episode major depression w'out psychotic symptoms	1
Eu32211	[X]Single episode agitated depressn w'out psychotic symptoms	1
Eu32300	[X]Severe depressive episode with psychotic symptoms	1
Eu33.14	[X]Seasonal depressive disorder	1
Eu33.15	[X]SAD - Seasonal affective disorder	1
Eu06y11	[X]Right hemispheric organic affective disorder	1
Eu33316	[X]Recurrent severe episodes/reactive depressive psychosis	1
Eu33315	[X]Recurrent severe episodes of psychotic depression	1
Eu33.13	[X]Recurrent episodes of reactive depression	1
Eu33.12	[X]Recurrent episodes of psychogenic depression	1
Eu33.11	[X]Recurrent episodes of depressive reaction	1
Eu33300	[X]Recurrent depress disorder cur epi severe with psyc symp	1
Eu3y111	[X]Recurrent brief depressive episodes	1
Eu33314	[X]Recurr severe episodes/psychogenic depressive psychosis	1
Eu33313	[X]Recurr severe episodes/major depression+psychotic symptom	1
Eu43z00	[X]Reaction to severe stress, unspecified	1
Eu43.00	[X]Reaction to severe stress, and adjustment disorders	1
Eu32z13	[X]Prolonged single episode of reactive depression	1
Eu42100	[X]Predominantly compulsive acts [obsessional rituals]	1
Eu20400	[X]Post-schizophrenic depression	1
Eu43100	[X]Post - traumatic stress disorder	1
Eu40.00	[X]Phobic anxiety disorders	1
Eu34.00	[X]Persistent mood affective disorders	1
Eu34z00	[X]Persistent mood affective disorder, unspecified	1
Eu34114	[X]Persistant anxiety depression	1
Eu41012	[X]Panic state	1
Eu40012	[X]Panic disorder with agoraphobia	1
Eu41011	[X]Panic attack	1
Eu3yy00	[X]Other specified mood affective disorders	1

Read code	Description	Number of studies
Eu3y000	[X]Other single mood affective disorders	1
Eu3y100	[X]Other recurrent mood affective disorders	1
Eu43y00	[X]Other reactions to severe stress	1
Eu34y00	[X]Other persistent mood affective disorders	1
Eu42y00	[X]Other obsessive-compulsive disorders	1
Eu3y.00	[X]Other mood affective disorders	1
Eu41300	[X]Other mixed anxiety disorders	1
Eu31y00	[X]Other bipolar affective disorders	1
Eu05300	[X]Organic mood [affective] disorders	1
Eu05400	[X]Organic anxiety disorder	1
Eu60513	[X]Obsessive-compulsive personality disorder	1
Eu42.12	[X]Obsessive-compulsive neurosis	1
Eu42z00	[X]Obsessive-compulsive disorder, unspecified	1
Eu42.00	[X]Obsessive - compulsive disorder	1
Eu4..00	[X]Neurotic, stress - related and somoform disorders	1
Eu34113	[X]Neurotic depression	1
Eu3..00	[X]Mood - affective disorders	1
Eu33z11	[X]Monopolar depression NOS	1
Eu3y011	[X]Mixed affective episode	1
Eu41211	[X]Mild anxiety depression	1
Eu31.13	[X]Manic-depressive reaction	1
Eu31.12	[X]Manic-depressive psychosis	1
Eu31.11	[X]Manic-depressive illness	1
Eu33312	[X]Manic-depress psychosis,depressed type+psychotic symptoms	1
Eu33213	[X]Manic-depress psychosis,depressd,no psychotic symptoms	1
Eu32700	[X]Major depression, severe without psychotic symptoms	1
Eu32800	[X]Major depression, severe with psychotic symptoms	1
Eu33212	[X]Major depression, recurrent without psychotic symptoms	1
Eu32600	[X]Major depression, moderately severe	1
Eu32500	[X]Major depression, mild	1
Eu33211	[X]Endogenous depression without psychotic symptoms	1
Eu33311	[X]Endogenous depression with psychotic symptoms	1
Eu51511	[X]Dream anxiety disorder	1
Eu34112	[X]Depressive personality disorder	1
Eu34111	[X]Depressive neurosis	1
Eu32z12	[X]Depressive disorder NOS	1
Eu92000	[X]Depressive conduct disorder	1
Eu32z11	[X]Depression NOS	1
Eu60511	[X]Compulsive personality disorder	1
Eu63011	[X]Compulsive gambling	1
Eu43013	[X]Combat fatigue	1
Eu31z00	[X]Bipolar affective disorder, unspecified	1
Eu31700	[X]Bipolar affective disorder, currently in remission	1
Eu31600	[X]Bipolar affective disorder, current episode mixed	1
Eu31900	[X]Bipolar affective disorder type II	1
Eu31800	[X]Bipolar affective disorder type I	1
Eu31.00	[X]Bipolar affective disorder	1
Eu31300	[X]Bipolar affect disorder cur epi mild or moderate depressn	1
Eu31500	[X]Bipolar affect dis cur epi severe depres with psyc symp	1
Eu31y11	[X]Bipolar II disorder	1
Eu31911	[X]Bipolar II disorder	1
Eu31400	[X]Bipol aff disord, curr epis sev depress, no psychot symp	1
Eu32y11	[X]Atypical depression	1

Read code	Description	Number of studies
Eu41113	[X]Anxiety state	1
Eu41112	[X]Anxiety reaction	1
Eu41111	[X]Anxiety neurosis	1
Eu41y11	[X]Anxiety hysteria	1
Eu41z11	[X]Anxiety NOS	1
Eu42.11	[X]Anankastic neurosis	1
Eu40011	[X]Agoraphobia without history of panic disorder	1
Eu40000	[X]Agoraphobia	1
Eu3z.11	[X]Affective psychosis NOS	1
Eu34011	[X]Affective personality disorder	1
Eu43000	[X]Acute stress reaction	1
Eu43012	[X]Acute reaction to stress	1
Eu32z14	[X] Reactive depression NOS	1
ZV11112	[V]Personal history of manic-depressive psychosis	1
ZV11111	[V]Personal history of manic-depressive psychosis	1
ZV11100	[V]Personal history of affective disorder	1
R2y2.00	[D]Nervousness	1
1BK..00	Worried	1
E11y000	Unspecified manic-depressive psychoses	1
E117400	Unspecified bipolar affective disorder,severe with psychosis	1
E117000	Unspecified bipolar affective disorder, unspecified	1
E117300	Unspecified bipolar affective disorder, severe, no psychosis	1
E117200	Unspecified bipolar affective disorder, moderate	1
E117100	Unspecified bipolar affective disorder, mild	1
E117600	Unspecified bipolar affective disorder, in full remission	1
E117z00	Unspecified bipolar affective disorder, NOS	1
E117.00	Unspecified bipolar affective disorder	1
E117500	Unspecified bipolar affect disord, partial/unspec remission	1
E11z000	Unspecified affective psychoses NOS	1
E211000	Unspecified affective personality disorder	1
1JJ..00	Suspected depression	1
E284.00	Stress reaction causing mixed disturbance of emotion/conduct	1
67J..00	Stress counselling	1
E202.11	Social phobic disorders	1
E112400	Single major depressive episode, severe, with psychosis	1
E002z00	Senile dementia with depressive or paranoid features NOS	1
E002.00	Senile dementia with depressive or paranoid features	1
E002100	Senile dementia with depression	1
1BT..12	Sad mood	1
8HHq.00	Referral for guided self-help for depression	1
8HHp.00	Referral for guided self-help for anxiety	1
Z4I7211	Reducing anxiety	1
E113400	Recurrent major depressive episodes, severe, with psychosis	1
Z4I7100	Recognising anxiety	1
E11z100	Rebound mood swings	1
E130.00	Reactive depressive psychosis	1
E130.11	Psychotic reactive depression	1
E001300	Presenile dementia with depression	1
E2A2.11	Post-traumatic brain syndrome	1
E202.00	Phobic disorders	1
E202.12	Phobic anxiety	1
E202000	Phobia unspecified	1
8CAa.00	Patient given advice about management of depression	1

Read code	Description	Number of studies
E200111	Panic attack	1
E29y100	Other post-traumatic stress disorder	1
E11y300	Other mixed manic-depressive psychoses	1
E11yz00	Other and unspecified manic-depressive psychoses NOS	1
E11y.00	Other and unspecified manic-depressive psychoses	1
E11z.00	Other and unspecified affective psychoses	1
E11zz00	Other affective psychosis NOS	1
E283.00	Other acute stress reactions	1
E283z00	Other acute stress reaction NOS	1
E03y200	Organic affective syndrome	1
9kQ..00	On full dose long term treatment depression - enh serv admin	1
9HA0.00	On depression register	1
E203.00	Obsessive-compulsive disorders	1
E203z00	Obsessive-compulsive disorder NOS	1
E214100	Obsessional personality	1
E203100	Obsessional neurosis	1
225J.00	O/E - panic attack	1
2259.00	O/E - nervous	1
225K.00	O/E - fearful mood	1
2253.00	O/E - distressed	1
2258.00	O/E - anxious	1
E116000	Mixed bipolar affective disorder, unspecified	1
E116300	Mixed bipolar affective disorder, severe, without psychosis	1
E116400	Mixed bipolar affective disorder, severe, with psychosis	1
E116500	Mixed bipolar affective disorder, partial/unspec remission	1
E116200	Mixed bipolar affective disorder, moderate	1
E116100	Mixed bipolar affective disorder, mild	1
E116600	Mixed bipolar affective disorder, in full remission	1
E116z00	Mixed bipolar affective disorder, NOS	1
E116.00	Mixed bipolar affective disorder	1
E11z200	Masked depression	1
E115.11	Manic-depressive - now depressed	1
13Y3.00	Manic-depression association member	1
1BT..11	Low mood	1
1BU..00	Loss of hope for the future	1
1BQ..00	Loss of capacity for enjoyment	1
146D.00	H/O: manic depressive disorder	1
1465.00	H/O: depression	1
1466.00	H/O: anxiety state	1
1B1H.00	Frightened	1
Z522600	Flooding - obsessional compulsive disorder	1
1B1T.00	Feeling stressed	1
16ZB100	Feeling low or worried	1
E202D00	Fear of death	1
1B1H.11	Fear	1
9hC..00	Exception reporting: depression quality indicators	1
9hC0.00	Excepted from depression quality indicators: Patient unsuita	1
9hC1.00	Excepted from depression quality indicators: Informed dissen	1
E112.13	Endogenous depression first episode	1
E112.12	Endogenous depression first episode	1
E113.11	Endogenous depression - recurrent	1
E112.14	Endogenous depression	1
9N54.00	Encounter for fear	1

Read code	Description	Number of studies
1B1U.11	Depressive symptoms	1
E11..12	Depressive psychoses	1
E211200	Depressive personality disorder	1
9Ov3.00	Depression monitoring verbal invite	1
9Ov2.00	Depression monitoring third letter	1
9Ov4.00	Depression monitoring telephone invite	1
9Ov1.00	Depression monitoring second letter	1
9Ov0.00	Depression monitoring first letter	1
9Ov..00	Depression monitoring administration	1
9H91.00	Depression medication review	1
8BK0.00	Depression management programme	1
9H92.00	Depression interim review	1
9H90.00	Depression annual review	1
9k4..00	Depression - enhanced services administration	1
9k40.00	Depression - enhanced service completed	1
E214.00	Compulsive personality disorders	1
E214z00	Compulsive personality disorder NOS	1
E203000	Compulsive neurosis	1
1P3..00	Compulsive behaviour	1
E28..11	Combat fatigue	1
1B1V.00	C/O - panic attack	1
1B17.11	C/O - feeling depressed	1
E290z00	Brief depressive reaction NOS	1
E290.00	Brief depressive reaction	1
E11..11	Bipolar psychoses	1
E115600	Bipolar affective disorder, now depressed, in full remission	1
E115000	Bipolar affective disorder, currently depressed, unspecified	1
E115200	Bipolar affective disorder, currently depressed, moderate	1
E115100	Bipolar affective disorder, currently depressed, mild	1
E115z00	Bipolar affective disorder, currently depressed, NOS	1
E115.00	Bipolar affective disorder, currently depressed	1
E115300	Bipolar affect disord, now depressed, severe, no psychosis	1
E115400	Bipolar affect disord, now depressed, severe with psychosis	1
E115500	Bipolar affect disord, now depressed, part/unspec remission	1
E11y200	Atypical depressive disorder	1
E004300	Arteriosclerotic dementia with depression	1
1B1H.12	Apprehension	1
1B13.00	Anxiousness	1
8G94.00	Anxiety management training	1
Z4L1.00	Anxiety counselling	1
173f.00	Anxiety about breathlessness	1
6659000	Antidepressant drug treatment started	1
E214000	Anankastic personality	1
E203.11	Anancastic neurosis	1
Z4I7200	Alleviating anxiety	1
E202200	Agoraphobia without mention of panic attacks	1
E202100	Agoraphobia with panic attacks	1
E112.11	Agitated depression	1
E11..00	Affective psychoses	1
E211.00	Affective personality disorder	1
E292400	Adjustment reaction with anxious mood	1
E282.00	Acute stupor state due to acute stress reaction	1
E28z.00	Acute stress reaction NOS	1

Read code	Description	Number of studies
E28..00	Acute reaction to stress	1
E283100	Acute posttrauma stress state	1
E280.00	Acute panic state due to acute stress reaction	1
E281.00	Acute fugue state due to acute stress reaction	1
Z4I7.00	Acknowledging anxiety	1
1B12.00	'Nerves' - nervousness	1
9kQ..11		1

Table 6. List of Read codes used in the studies of cognitive impairment.

Read code	Description	Number of studies
Eu01.00	[X]Vascular dementia	13
Eu01200	[X]Subcortical vascular dementia	13
Eu00112	[X]Senile dementia,Alzheimer's type	13
Eu00011	[X]Presenile dementia,Alzheimer's type	13
Eu01100	[X]Multi-infarct dementia	13
Eu00.00	[X]Dementia in Alzheimer's disease	13
Eu01.11	[X]Arteriosclerotic dementia	13
Eu00z11	[X]Alzheimer's dementia unspec	13
E000.00	Uncomplicated senile dementia	13
E004.11	Multi infarct dementia	13
E004.00	Arteriosclerotic dementia	13
F110000	Alzheimer's disease with early onset	13
F110.00	Alzheimer's disease	13
Eu01z00	[X]Vascular dementia, unspecified	12
Eu01000	[X]Vascular dementia of acute onset	12
Eu00012	[X]Primary degen dementia, Alzheimer's type, presenile onset	12
Eu00113	[X]Primary degen dementia of Alzheimer's type, senile onset	12
Eu01111	[X]Predominantly cortical dementia	12
Eu00z00	[X]Dementia in Alzheimer's disease, unspecified	12
Eu00100	[X]Dementia in Alzheimer's disease with late onset	12
Eu00000	[X]Dementia in Alzheimer's disease with early onset	12
Eu00200	[X]Dementia in Alzheimer's dis, atypical or mixed type	12
Eu00013	[X]Alzheimer's disease type 2	12
Eu00111	[X]Alzheimer's disease type 1	12
E004000	Uncomplicated arteriosclerotic dementia	12
E004200	Arteriosclerotic dementia with paranoia	12
E004300	Arteriosclerotic dementia with depression	12
E004100	Arteriosclerotic dementia with delirium	12
E004z00	Arteriosclerotic dementia NOS	12
F110100	Alzheimer's disease with late onset	12
Eu01y00	[X]Other vascular dementia	11
Eu01300	[X]Mixed cortical and subcortical vascular dementia	11
Eu02.00	[X]Dementia in other diseases classified elsewhere	11
Eu02300	[X]Dementia in Parkinson's disease	11
Eu02z00	[X] Unspecified dementia	11
Eu02z14	[X] Senile dementia NOS	11
E00..12	Senile/presenile dementia	11
E002000	Senile dementia with paranoia	11
E002100	Senile dementia with depression	11
E001.00	Presenile dementia	11
E041.00	Dementia in conditions EC	11
Fyu3000	[X]Other Alzheimer's disease	10
Eu02500	[X]Lewy body dementia	10
Eu02y00	[X]Dementia in other specified diseases classif elsewhere	10
Eu04100	[X]Delirium superimposed on dementia	10
Eu02z16	[X] Senile dementia, depressed or paranoid type	10
Eu02z13	[X] Primary degenerative dementia NOS	10
Eu02z11	[X] Presenile dementia NOS	10
E001000	Uncomplicated presenile dementia	10
E002z00	Senile dementia with depressive or paranoid features NOS	10
E002.00	Senile dementia with depressive or paranoid features	10

Read code	Description	Number of studies
E003.00	Senile dementia with delirium	10
E00..11	Senile dementia	10
E001200	Presenile dementia with paranoia	10
E001300	Presenile dementia with depression	10
E001100	Presenile dementia with delirium	10
E001z00	Presenile dementia NOS	10
Eu02000	[X]Dementia in Pick's disease	9
Eu02200	[X]Dementia in Huntington's disease	9
Eu02400	[X]Dementia in human immunodef virus [HIV] disease	8
Eu02100	[X]Dementia in Creutzfeldt-Jakob disease	8
F112.00	Senile degeneration of brain	8
F116.00	Lewy body disease	8
F111.00	Pick's disease	7
E012.00	Other alcoholic dementia	6
E012.11	Alcoholic dementia NOS	6
Eu10711	[X]Alcoholic dementia NOS	5
E00z.00	Senile or presenile psychoses NOS	5
6AB..00	Dementia annual review	5
Eu02z15	[X] Senile psychosis NOS	4
Eu02z12	[X] Presenile psychosis NOS	4
ZS7C500	Language disorder of dementia	4
E02y100	Drug-induced dementia	4
66h..00	Dementia monitoring	4
R00z011	[D]Memory deficit	3
e000.00	Uncomplicated senile dementia	3
Z7CEG00	Transient memory loss	3
Z7CF811	Short-term memory loss	3
E00..00	Senile and presenile organic psychotic conditions	3
2900	SENILE DEMENTIA	3
Z7CF800	Poor short-term memory	3
Z7CEH15	Poor memory	3
2901A	PRESENILE DEMENTIA	3
E00y.00	Other senile and presenile organic psychoses	3
3A30.00	Memory: present place not knwn	3
Z7CEH14	Memory problem	3
1B1A.12	Memory loss symptom	3
1B1A.00	Memory loss - amnesia	3
Z7CE611	Memory loss	3
Z7CEJ00	Memory lapses	3
Z7CEH00	Memory impairment	3
1B1A.13	Memory disturbance	3
Z7CEC11	Loss of memory for recent events	3
Z7CE615	Loss of memory	3
1461.00	H/O: dementia	3
3AE6.00	GDS level 7 - very severe cognitive decline	3
3AE5.00	GDS level 6 - severe cognitive decline	3
3AE4.00	GDS level 5 - moderately severe cognitive decline	3
3AE3.00	GDS level 4 - moderate cognitive decline	3
3AE2.00	GDS level 3 - mild cognitive decline	3
3AE1.00	GDS level 2 - very mild cognitive decline	3
2930	DEMENTIA ARTERIOSCLEROTIC	3
299 G	DEMENTIA AGGRESSIVE	3
299 B	DEMENTIA	3

Read code	Description	Number of studies
E012000	Chronic alcoholic brain syndrome	3
1B1A.11	Amnesia symptom	3
ZR1K.00	Alzheimer's disease assessment scale	3
2901B	ALZHEIMER'S DISEASE	3
ZR1K.11	ADAS - Alzheimer's disease assessment scale	3
Eu80200	[X]Receptive language disorder	2
R00z000	[D]Amnesia (retrograde)	2
ZS78D00	Wernicke's dysphasia	2
ZS78D13	Wernicke's aphasia	2
Z7C3500	Unable to use verbal reasoning	2
Z7C3200	Unable to reason	2
Z7C5100	Unable to concentrate	2
1B1A000	Temporary loss of memory	2
Z7CEF00	Temporary loss of memory	2
Z7C5313	Short concentration span	2
Z7C5312	Short attention span	2
1BR0.11	Short attention span	2
8HTY.00	Referral to memory clinic	2
8Hla.00	Referral to dementia care advisor	2
Z7C5300	Reduced concentration span	2
1BR..00	Reduced concentration	2
Z7C5311	Reduced attention span	2
E00y.11	Presbyophrenic psychosis	2
Z7CEB12	Poor memory for remote events	2
Z7CFO00	Poor long-term memory	2
1BW..00	Poor concentration	2
Z7CEC12	No memory for recent events	2
Z7CEK00	Minor memory lapses	2
Z7CEL00	Mild memory disturbance	2
E2A1000	Mild memory disturbance	2
3A40.00	Memory: present year not known	2
3A20.00	Memory: present time not known	2
3A10.00	Memory: own age not known	2
3A50.00	Memory: own DOB not known	2
3A70.00	Memory: important event not kn	2
3A80.00	Memory: import.person not knwn	2
3A91.00	Memory: count down unsuccess.	2
3AA1.00	Memory: address recall unsucc.	2
Z7A1500	Memory retraining	2
Z7CE412	Memory loss symptom	2
Z7CE614	Memory loss - amnesia	2
Z7CE413	Memory loss - amnesia	2
Z7CE612	Memory gone	2
Z7CEH11	Memory dysfunction	2
Z7CE400	Memory disturbance (& amnesia (& symptom))	2
Z7CE414	Memory disturbance	2
Z7CEH12	Memory deficit	2
Z7CFx00	Memory aided by use of labels	2
Z7CE415	Loss of memory	2
Z7CFO11	Long-term memory loss	2
Z7C5111	Lack of concentration	2
Z7CE616	LOM - Loss of memory	2
2901D	JACOB- CREUZFELDT DISEASE WITH DEMENTIA	2

Read code	Description	Number of studies
Z7CEN11	Invents experiences to compensate for loss of memory	2
Z7CEA11	Impairment of working memory	2
Z7CEA13	Impairment of primary memory	2
Z7C1.00	Impaired cognition	2
129B.00	FH: Alzheimer's disease	2
Z7A1A00	Executive functions training	2
9hD0.00	Excepted from dementia quality indicators: Patient unsuitabl	2
9hD1.00	Excepted from dementia quality indicators: Informed dissent	2
1S21.00	Disturbance of memory for order of events	2
Z7C3C00	Difficulty using visuospatial reasoning	2
Z7C3600	Difficulty using verbal reasoning	2
Z7C3300	Difficulty reasoning	2
Z7C4A00	Difficulty processing information at normal speed	2
Z7C4700	Difficulty processing information accurately	2
Z7C4300	Difficulty processing information	2
Z7CI100	Difficulty making plans	2
ZR3V.13	Dementia rating scale	2
9Ou4.00	Dementia monitoring verbal invite	2
9Ou3.00	Dementia monitoring third letter	2
9Ou5.00	Dementia monitoring telephone invite	2
9Ou2.00	Dementia monitoring second letter	2
9Ou1.00	Dementia monitoring first letter	2
3A...12	Dementia assessment	2
Z7CGP00	Delayed verbal memory	2
ZR3V.11	DRS - Clinical dementia rating scale	2
Y0601JS	DEMENTIA CLINIC ATTENDANCE	2
Y060 JS	DEMENTIA CLINIC	2
2919	DEMENTIA ALCOHOLIC	2
Z73..00	Cognitive intervention strategies	2
28E..00	Cognitive decline	2
ZR3V.00	Clinical dementia rating scale	2
ZR3V.12	CDR - Clinical dementia rating scale	2
Z7CEH13	Bad memory	2
ZR2X.12	BDRS - Blessed dementia rating scale	2
Z7A1400	Attention training	2
Z7CE600	Amnesia	2
EU02y..0		2
X0034		2
AE..00		2
Ryu5.00	[X]Symptoms/signs inv cognit, percept, emotion state & behav	1
Eu80000	[X]Specific speech articulation disorder	1
Eu81500	[X]Severe learning disability	1
Eu81700	[X]Profound learning disability	1
Ryu5000	[X]Other amnesia	1
Ryu5100	[X]Oth & unspec symptom/sign involv cognit funct/awareness	1
Eu81600	[X]Mild learning disability	1
Eu05700	[X]Mild cognitive disorder	1
Eu81z12	[X]Learning disorder NOS	1
Eu81z11	[X]Learning disability NOS	1
Eu81z13	[X]Learn acquisition disab NOS	1
Eu03.11	[X]Korsakov's psychosis, nonalcoholic	1
Eu10611	[X]Korsakov's psychosis, alcohol induced	1
Eu80014	[X]Functional speech articulation disorder	1

Read code	Description	Number of studies
Eu80100	[X]Expressive language disorder	1
Eu90000	[X]Disturbance of activity and attention	1
Eu44000	[X]Dissociative amnesia	1
Ryu5700	[X]Disorientation, unspecified	1
Eu10712	[X]Chronic alcoholic brain syndrome	1
Eu80600	[X]Auditory processing disorder	1
Eu90011	[X]Attention deficit hyperactivity disorder	1
Eu9y700	[X]Attention deficit disorder	1
ZS91.12	[X]Attention deficit disorder	1
Eu04.12	[X]Acute / subacute confusional state, nonalcoholic	1
ZV40000	[V]Problems with learning	1
R045100	[D]Dysphasia	1
R00zX00	[D]Disorientation, unspecified	1
R043.00	[D]Aphasia	1
R00z500	[D]Anterograde amnesia	1
E011200	Wernicke-Korsakov syndrome	1
C253.00	Wernicke's encephalopathy	1
C251.11	Wernicke's encephalopathy	1
Z7CMB00	Visuospatial agnosia	1
Z7A1800	Visual processing training	1
F481J00	Visual disorientation syndrome	1
F584000	Unspecified other abnormal auditory perception	1
Z7CH300	Unrealistic planning	1
Z7C7200	Unable to write	1
ZT46200	Unable to use verbal communication	1
ZT49200	Unable to use non-verbal communication	1
ZT47200	Unable to use language	1
Z7CI500	Unable to use decision-making strategies	1
Z7C6200	Unable to tell the time	1
ZT4f200	Unable to responds to communication by others	1
Z7C2600	Unable to recognise surroundings	1
Z7C2200	Unable to recognise sounds	1
Z7C2D00	Unable to recognise parts of own body	1
Z7C2L11	Unable to recognise objects visually	1
Z7C2L00	Unable to recognise objects by sight	1
Z7C2H00	Unable to recognise objects	1
Z7C2T00	Unable to recognise familiar people	1
Z7C2R00	Unable to recognise faces by sight	1
Z7C2P00	Unable to recognise faces	1
Z7CFC00	Unable to recall five digit number at five minutes	1
Z7C8200	Unable to read	1
Z7C4900	Unable to process information at normal speed	1
Z7C4600	Unable to process information accurately	1
Z7C4200	Unable to process information	1
ZM18200	Unable to plan meals	1
Z7CH200	Unable to plan	1
ZN28200	Unable to organise a journey	1
Z7CI900	Unable to make considered choices	1
Z7C4C00	Unable to analyse information	1
1B1S.00	Transient global amnesia	1
Z7CE700	Transient global amnesia	1
ZS78600	Transcortical sensory dysphasia	1
ZS78411	Transcortical motor aphasia	1

Read code	Description	Number of studies
Z7CE711	TGA - Transient global amnesia	1
ZS78300	Subcortical aphasia	1
E031.00	Subacute confusional state	1
Z7A2200	Strategy training for perceptual skills	1
ZS5..00	Speech and language dyspraxias	1
ZS...00	Speech and language disorder	1
ZS73.00	Specific language impairment	1
ZS73.11	Specific language disorder	1
Z7CC700	Spatial disorientation	1
Z7CD400	Slow learner	1
F483200	Simultaneous visual perception without fusion	1
1B1A100	Short-term memory loss	1
28E2.00	Severe cognitive impairment	1
ZS78A00	Semantic dysphasia	1
9Nk1.00	Seen in memory clinic	1
9N0y.00	Seen in learning disabilities clinic	1
342 CP	SENILE PARKINSONISM	1
Z7CE900	Retrograde amnesia	1
8HHP.00	Referral to learning disability team	1
8H4f.00	Referral to learning disabilities psychiatrist	1
1BR0.00	Reduced concentration span	1
ZS72.00	Receptive language impairment	1
ZS78C00	Receptive dysphasia	1
ZS78C11	Receptive aphasia	1
Z7A1700	Reality orientation	1
Z7A1711	RO - Reality orientation	1
Z7CE911	RA - Retrograde amnesia	1
ZRbf.00	Psycholinguistic assessments of language process in aphasia	1
ZD38300	Promoting aphasics communication effectiveness programme	1
8G96.00	Problem solving therapy	1
ZS78F00	Posterior dysphasia	1
1B1Y.00	Poor visual sequential memory	1
1B1a.00	Poor auditory sequential memory	1
F591z00	Perceptive hearing loss NOS	1
F591.14	Perceptive hearing loss	1
F591.13	Perceptive deafness	1
Z7CL700	Perception that things appear grey	1
Z7CL800	Perception that things appear flat	1
Z7CLE00	Perception of things changing size	1
Z7CLH00	Perception of things changing shape	1
Z7CL911	Perception of things changing colour	1
E2F2.00	Other specific learning difficulty	1
F584z00	Other abnormal auditory perception NOS	1
F584.00	Other abnormal auditory perception	1
Z7A1600	Orientation training	1
Z7CC312	Orientation poor	1
Z7CC311	Orientation confused	1
E2A1100	Organic memory impairment	1
918e.00	On learning disability register	1
29J4.00	O/E - sensory inattention	1
2B46.11	O/E - sensory dysphasia	1
2B43.00	O/E - sensory aphasia	1
2BM3.11	O/E - perceptive deafness	1

Read code	Description	Number of studies
2B45.11	O/E - motor dysphasia	1
2B42.00	O/E - motor aphasia	1
2B46.00	O/E - dysphasia - sensory	1
2B45.00	O/E - dysphasia - motor	1
2B47.00	O/E - dysphasia - NOS	1
2B4..12	O/E - dysphasia	1
2B44.00	O/E - aphasia NOS	1
2B4..11	O/E - aphasia	1
ZS78I00	Non-fluent dysphasia	1
ZS78I11	Non-fluent aphasia	1
28E1.00	Moderate cognitive impairment	1
ZS78200	Mixed transcortical dysphasia	1
ZS78900	Mixed dysphasia	1
ZS78911	Mixed aphasia	1
Z7C2700	Mistakes people's identity	1
28E0.00	Mild cognitive impairment	1
3A60.00	Memory: present month not knwn	1
3A11.00	Memory: own age known	1
Z7A1300	Memory skills training	1
Z7CFz00	Memory aided by use of lists	1
Z7CFw00	Memory aided by use of diary	1
ZS34.11	Learning disability	1
9HB2.00	Learning disabilities health action plan reviewed	1
9HB1.00	Learning disabilities health action plan offered	1
9HB0.00	Learning disabilities health action plan declined	1
9HB4.00	Learning disabilities health action plan completed	1
9HB6.11	Learning disabilities annual health check declined	1
9HB6.00	Learning disabilities annual health assessment declined	1
9HB5.00	Learning disabilities annual health assessment	1
Z7CD200	Learning difficulties	1
ZS3..00	Language-related cognitive disorder	1
ZS7..00	Language impairment	1
ZS7C600	Language disorder associated with thought disorder	1
13ZA.00	Language difficulty	1
E011100	Korsakov's alcoholic psychosis with peripheral neuritis	1
E011000	Korsakov's alcoholic psychosis	1
E040.11	Korsakoff's non-alcoholic psychosis	1
ZS78D12	Jargon dysphasia	1
ZS78D11	Jargon aphasia	1
A411.00	Jakob-Creutzfeldt disease	1
ZS78212	Isolation dysphasia	1
Z7CEA12	Impairment of immediate recall	1
Z7CD300	Impaired ability to learn new material	1
E201700	Hysterical amnesia	1
Z7CL100	Heightened visual perception	1
Z7CL511	Heightened perception of touch	1
Z7CLO00	Heightened perception of taste	1
Z7CLP11	Heightened perception of sound	1
Z7CLN11	Heightened perception of smells	1
Z7CLN12	Heightened perception of odours	1
Z7CLN00	Heightened olfactory perception	1
Z7CLP00	Heightened auditory perception	1
ZRLfE00	Health of the Nation Outcome Scale item 4 - cognitive probl	1

Read code	Description	Number of studies
Z7CF200	Has delayed recall	1
ZS78800	Global dysphasia	1
3AE..00	Global deterioration scale: assessment of prim deg dementia	1
ZS78811	Global aphasia	1
Z7CE500	Forgetful	1
ZS78E11	Fluent aphasia	1
1281.00	FH: Senile dementia	1
ZS71.00	Expressive language impairment	1
ZS84.00	Expressive language disorder	1
ZS78G11	Expressive aphasia	1
9hD..00	Exception reporting: dementia quality indicators	1
ZS78K00	Efferent motor dysphasia	1
ZS78K11	Efferent motor aphasia	1
ZS78.00	Dysphasia	1
ZT46400	Does not use verbal communication	1
ZT4J400	Does not use the elements of language	1
ZT49400	Does not use non-verbal communication	1
ZT4f400	Does not respond to communication by others	1
Z7C2900	Does not recognise self	1
Z7C2A00	Does not recognise photographs of self	1
Z7CEM00	Distortion of memory	1
Z7CC600	Disorientation for person	1
Z7C7300	Difficulty writing	1
ZT46500	Difficulty using verbal communication	1
ZT49500	Difficulty using non-verbal communication	1
Z7CI600	Difficulty using decision-making strategies	1
ZT4A500	Difficulty using a non-speech system for communication	1
Z7CJ100	Difficulty solving problems	1
Z7C8300	Difficulty reading	1
ZM18500	Difficulty planning meals	1
Z7C9300	Difficulty performing logical sequencing	1
ZN28500	Difficulty organising a journey	1
Z7CI200	Difficulty making decisions	1
Z7CIA00	Difficulty making considered choices	1
ZT4g500	Difficulty imitating forms of communication	1
Z7C4D00	Difficulty analysing information	1
9HB7.11	Did not attend learning disabilities annual health check	1
9HB7.00	Did not attend learning disabilities annual health assessmnt	1
9Ou..00	Dementia monitoring administration	1
ZS93.11	DAMP - Deficits in attention motor control and perception	1
ZS78B11	Conduction aphasia	1
F591y00	Combined perceptive hearing loss	1
Z7A1.00	Cognitive skills training	1
ZD15.00	Cognitive neuropsychological language therapy	1
ZD38200	Cognitive behavioural language therapy	1
E2E0z00	Child attention deficit disorder NOS	1
E2E0.00	Child attention deficit disorder	1
Z7CLK00	Changed perception of time	1
F11x700	Cerebral degeneration due to Jakob - Creutzfeldt disease	1
ZS78H00	Broca's dysphasia	1
ZS78H11	Broca's aphasia	1
ZS76.00	Auditory processing disorder	1
E2E0000	Attention deficit without hyperactivity	1

Read code	Description	Number of studies
ZS91.00	Attention deficit disorder	1
ZS78.11	Aphasia	1
Z7CE811	Antegrade amnesia	1
ZS78511	Anomic aphasia	1
ZS78500	Anomia	1
Z7CEB00	Amnesia for remote events	1
Z7CEC00	Amnesia for recent events	1
Z7CEE00	Amnesia for important personal information	1
Z7CED00	Amnesia for day to day facts	1
13Y7.00	Alzheimer's disease society member	1
ZS7C.00	Acquired language disorder	1
ZS78100	Acquired dysphasias	1
9OlA.00	ADHD monitoring invitation third letter	1
9Ol8.00	ADHD monitoring invitation first letter	1
ZS91.11	ADD - Attention deficit disorder	1
Z7C2N00		1
E0120		1
6AB.00		1
Z7C3800		1
Xa25J		1
Z7CEB11		1
Z7CC800		1
Z7C2J00		1
X00Rk		1
Z7C3B00		1
Z7A1100		1
Z7C6300		1
Z7C9200		1
Z7C3900		1
Z7C2F00		1
Z7C2B00		1
Z7A1712		1
Z7A2100		1
Z7A2300		1

Table 7. ICD codes used in the studies of dementia.

Study	Databases	ICD version	List of codes
Brown, 2016	CPRD + HES	ICD-10	E512, F00, F01, F02, F03, F10.6, F10.7, G30, G31.0
Emdin, 2016	CPRD + HES	ICD-10	F01

CPRD – Clinical Practice Research Datalink; HES – Hospital Episode Statistics; ICD - International Classification of Diseases.

Table 8. List of Read codes used in the studies of fatigue.

Read code	Description	Number of studies
168..00	Tiredness symptom	3
168..11	Fatigue - symptom	3
168..12	Lethargy - symptom	3
1682.00	Fatigue	3
1683.00	Tired all the time	3
1684.11	C/O - debility - malaise	3
168Z.00	Tiredness symptom NOS	3
8HkW.00	Referral to chronic fatigue syndrome specialist team	3
8HlL.00	Referral for chronic fatigue syndrome activity management	3
8Q1..00	Activity management for chronic fatigue syndrome	3
E205.00	Neurasthenia - nervous debility	3
E205.12	Tired all the time	3
Eu46011	[X]Fatigue syndrome	3
F286.00	Chronic fatigue syndrome	3
F286.11	CFS - Chronic fatigue syndrome	3
F286.12	Postviral fatigue syndrome	3
F286.13	PVFS - Postviral fatigue syn	3
F286.14	Post-viral fatigue syndrome	3
F286.15	Myalgic encephalomyelitis	3
F286.16	ME - Myalgic encephalomyelitis	3
F286000	Mild chronic fatigue syndrome	3
F286100	Moderate chronic fatigue syndrome	3
F286200	Severe chronic fatigue syndrome	3
R007.00	[D]Malaise and fatigue	3
R007100	[D]Fatigue	3
R007211	[D]General weakness	3
R007300	[D]Lethargy	3
R007400	[D]Postviral (asthenic) syndrome	3
R007411	[D]Post viral debility	3
R007500	[D]Tiredness	3
R007z00	[D]Malaise and fatigue NOS	3
168..13	Malaise - symptom	2
1683.11	C/O - 'tired all the time'	2
1684.00	Malaise/lethargy	2
1684.13	C/O - postviral syndrome	2
1688.00	Exhaustion	2
1B3..12	Weakness symptoms	2
E205.11	Nervous exhaustion	2
Eu46000	[X]Neurasthenia	2
F03y.12	Myalgic encephalomyelitis	2
N239.00	Fibromyalgia	2
N248.00	Fibromyalgia	2
R007000	[D]Malaise	2
R007200	[D]Asthenia NOS	2
R2y3.00	[D]Debility, unspecified	2
168..14	C/O 'Muzzy head'	1
1B32.00	Weakness present	1
8HkW.11	Referral to myalgic encephalomyelitis specialist team	1
8Q1..11	Activity management for myalgic encephalopathy	1
A4zy300	Encephalitis lethargica	1
Eu46y14	[X]Psychasthenia	1

Read code	Description	Number of studies
Eu46y15	[X]Psychasthenia neurosis	1
R007600	[D]Post polio exhaustion	1
R007z11	[D]Lassitude	1
R202.00	[D]Senile asthenia	1
SN44.00	Exhaustion due to exposure	1

Table 9. List of Read codes used in the studies of pain.

Read code	Description	Number of studies
NyuAG	[X]Uns sof tis d,use/overu/prs	2
NyuA	[X]Other soft tissue disorders	2
Nyu80	[X]Other myositis	2
Nyu3	[X]Other joint disorders	2
Ryu70	[X]Other chronic pain	2
NyuAF	[X]Oth spcf soft tissu disords	2
Nyu85	[X]Oth spcf disorders/muscle	2
NyuAA	[X]Oth sft tis diso/oth dis CE	2
Nyu8A	[X]Oth disordrs/muscle/dis CE	2
Nyu9	[X]Disorders/synovium+tendon	2
Nyu8	[X]Disorders of muscles	2
Nyu8B	[X]Disorder of muscle, unspec	2
R01zz	[D]Nerv/musculoskel.sympt.NOS	2
R01z	[D]Nerv/musculoskel.symp.other	2
R01	[D]Musculoskeletal symptoms	2
R01z2	[D]Musculoskeletal pain	2
R00z2	[D]General aches and pains	2
N22z	Synovium/tendon/bursa dis.NOS	2
N0950	Stiff joint NEC-site unspecif.	2
N0958	Stiff joint NEC-other specif.	2
N240	Rheumatism/fibrositis NOS	2
N2	Rheumatism, excl.the back	2
N240z	Rheumatism or fibrositis NOS	2
N2400	Rheumatism NOS - shoulder	2
N2403	Rheumatic pain	2
N24z	Polyalgia	2
N09	Other/unspecif.joint disorders	2
N22yz	Other tendon disorder NOS	2
N233z	Other specif.musc.disorder NOS	2
N06yz	Other specif.arthropathy NOS	2
N06y9	Other spec.arthr.-multipl.site	2
N09y	Other spec. joint disorders	2
N24	Other soft tissue disorders	2
N3z	Other musculoskeletal dis. NOS	2
N247	Other musculoskel.limb sympts.	2
N23y	Other muscle/ligament/fascia	2
N23yz	Other musc./lig./fasc.dis.NOS	2
N096z	Other joint symptoms NOS	2
N0968	Other joint sympt.-other spec.	2
N0969	Other joint sympt.-multip.site	2
N09yz	Other joint disorders NOS	2
N09y0	Other joint dis.-site unspec.	2
N09y8	Other joint dis.-other specif.	2
N09y9	Other joint dis.-multiple site	2
N2y	Nonarticular rheumatism OS	2
N2z	Nonarticular rheumatism NOS	2
N39	Nonallopathic lesions, NEC	2
N39z	Nonallopathic lesion NEC NOS	2
N2411	Myositis unspecified	2
N2480	Myofascial pain syndrome	2
N241	Myalgia/myositis unspecified	2

Read code	Description	Number of studies
N241z	Myalgia/myositis NOS	2
N3y	Musculoskeletal disorders OS	2
Ny	Musculoskeletal diseases OS	2
Nz	Musculoskeletal diseases NOS	2
N2402	Muscular rheumatism	2
N23z	Muscle/ligament/fascia dis.NOS	2
N2410	Muscle pain	2
N0959	Multiple joint stiffness	2
N0450	Juv ankylosing spondylitis	2
N095z	Joint stiffness NEC NOS	2
N095	Joint stiffness NEC	2
N09zz	Joint disorders NOS	2
N09z	Joint disorder NOS	2
N09z0	Joint disord.NOS-site unspecif	2
N09z8	Joint disord.NOS-other specif.	2
N09z9	Joint disord.NOS-multiple site	2
N096	Joint crepitus	2
N2401	Fibrositis unspecified	2
N2412	Fibromyositis NOS	2
N248	Fibromyalgia	2
N239	Fibromyalgia	2
N23	Fascia disorders	2
N06zB	Chronic arthritis	2
N06z0	Arthropathy NOS-site unspecif.	2
N06z8	Arthropathy NOS-other specif.	2
N06z9	Arthropathy NOS-multiple sites	2
N06zz	Arthropathy NOS	2
N0z	Arthropathies NOS	2
N06z	Arthritis	2
N0949	Arthralgia of multiple joints	2
N094z	Arthralgia NOS	2
N0940	Arthralgia - site unspecified	2
N0948	Arthralgia - other specified	2
N094	Ache in joint	2
OX7179KB	viral myalgia /ox	1
7K6T1	release of Torticollis	1
N2452	neuropathic pain	1
OX7289CH	back pain /ox	1
Syu4E	[X]Unspecif inj should/up arm	1
NyuB8	[X]Unsp osteopor + pathol frac	1
Nyu28	[X]Unilat second gonarthrosis	1
Nyu25	[X]Unilat primary gonarthrosis	1
Nyu4	[X]Systmc connctv tis disordrs	1
Nyu4C	[X]Sys diso/connctv t/o dis CE	1
Nyu95	[X]Synovitis+tenosyn/bact d CE	1
Nyu97	[X]Synovial hypertrophy, NEC	1
Ryu3	[X]Sym/sign inv nv/muscskel sy	1
Syu12	[X]Superf inj neck part unsp	1
Syu84	[X]Sprn/str oth unsp part knee	1
Syu46	[X]Spr/str oth/un part shl gir	1
Syu36	[X]Spr/str ot/un pt lum sp/pel	1
Syu18	[X]Spr/str jt/lg ot/un pt neck	1
Nyu92	[X]Spontans ruptr/oth tendons	1

Read code	Description	Number of studies
Nyu68	[X]Spondylpthy/oth diseases CE	1
Nyu66	[X]Spondylpth/o inf+paras d CE	1
Nyu69	[X]Spondylopathy unspecified	1
Nyu6	[X]Spondylopathies	1
Nyu5B	[X]Spin osteochondrosis, unsp	1
Nyu1G	[X]Seroposit rheum arthr, unsp	1
Nyu10	[X]Rheum arthrit+inv/o org/sys	1
Nyu05	[X]Reactv arthropathy/o dis CE	1
Eu45y	[X]Psychogenic torticollis	1
NyuE4	[X]Postproc muscsk disord,unsp	1
Nyu2F	[X]Post-traum arthr oth joints	1
NyuC9	[X]Periostitis/oth inf dis CE	1
Nyu51	[X]Other+unspecified kyphosis	1
Nyu64	[X]Other spondylosis	1
Nyu2D	[X]Other specified arthrosis	1
Nyu1B	[X]Other specified arthritis	1
Nyu65	[X]Other spcfd spondylopathies	1
Nyu3D	[X]Other spcfd joint disorders	1
NyuAB	[X]Other shoulder lesions	1
Nyu54	[X]Other secondary scoliosis	1
Nyu50	[X]Other secondary kyphosis	1
Nyu17	[X]Other secondary gout	1
Nyu21	[X]Other primary coxarthrosis	1
Nyu20	[X]Other polyarthrosis	1
Nyu46	[X]Other overlap syndromes	1
NyuB1	[X]Other osteoporosis	1
NyuC	[X]Other osteopathies	1
NyuC5	[X]Other osteonecrosis	1
NyuC3	[X]Other osteomyelitis	1
Nyu82	[X]Other ossification/muscle	1
Nyu37	[X]Other meniscus derangements	1
Nyu52	[X]Other lordosis	1
Nyu15	[X]Other juvenile arthritis	1
Nyu3C	[X]Other instability of joint	1
NyuA4	[X]Other infective bursitis	1
Nyu53	[X]Other idiopathic scoliosis	1
Nyu41	[X]Other giant cell arteritis	1
Nyu56	[X]Other fusion of spine	1
Nyu55	[X]Other forms of scoliosis	1
NyuAE	[X]Other enthesopathies,NEC	1
Nyu7	[X]Other dorsopathies	1
Nyu7A	[X]Other dorsalgia	1
Nyu36	[X]Other disorders of patella	1
Nyu44	[X]Other dermatomyositis	1
Nyu35	[X]Other derangements/patella	1
NyuB6	[X]Other cyst of bone	1
NyuC2	[X]Other chronic osteomyelitis	1
Nyu18	[X]Other chondrocalcinosis	1
Nyu81	[X]Other calcification/muscle	1
NyuA1	[X]Other bursitis of knee	1
NyuA6	[X]Other bursitis NEC	1
NyuA5	[X]Other bursal cyst	1
NyuA7	[X]Other bursa disorder	1

Read code	Description	Number of studies
NyuE3	[X]Other biomechanical lesions	1
NyuB4	[X]Other adult osteomalacia	1
NyuC0	[X]Other acute osteomyelitis	1
NyuC4	[X]Other 2ndary osteonecrosis	1
Nyu47	[X]Oth syst dis/connctv tissue	1
Nyu91	[X]Oth synovitis+tenosynovitis	1
Syu40	[X]Oth sup inj should/upp arm	1
Nyu62	[X]Oth spondylosis+myelopathy	1
Nyu79	[X]Oth specified dorsopathies	1
NyuC8	[X]Oth spcfd disorders of bone	1
Nyu1A	[X]Oth spcfc arthropathies,NEC	1
Nyu12	[X]Oth spcf rheumatd arthritis	1
Nyu61	[X]Oth spcf inflam spondylpath	1
NyuD4	[X]Oth spcf disordrs/cartilage	1
Nyu59	[X]Oth spcf deform dorsopaths	1
NyuD3	[X]Oth spc osteochondropathies	1
Syu3K	[X]Oth sp inj abd/low back/pel	1
Nyu2E	[X]Oth secondary coxarthrosis	1
Nyu83	[X]Oth ruptr/muscl(nontraumtc)	1
Nyu58	[X]Oth recur vertebrl subluxtn	1
Nyu03	[X]Oth reactive arthropathies	1
Nyu13	[X]Oth psoriatic arthropathies	1
Nyu26	[X]Oth post-traum gonarthrosis	1
Nyu23	[X]Oth post-traum coxarthrosis	1
NyuB0	[X]Oth osteoporosis+patholog #	1
Nyu39	[X]Oth intrnl derangemnts/knee	1
Nyu90	[X]Oth infectve(teno)synovitis	1
Nyu60	[X]Oth infectv spondylopathies	1
Nyu45	[X]Oth forms/systemc sclerosis	1
Nyu43	[X]Oth forms/sys lup erythemat	1
NyuA9	[X]Oth fibroblastic disorders	1
Nyu22	[X]Oth dysplastic coxarthrosis	1
NyuB5	[X]Oth diso/continuity of bone	1
NyuC6	[X]Oth diso/bone dvlpmnt+grwth	1
NyuE	[X]Oth dis musculosk+connect	1
Nyu70	[X]Oth cervicl disc displacmnt	1
Nyu71	[X]Oth cervicl disc degeneratn	1
Nyu72	[X]Oth cervical disc disorders	1
Nyu3A	[X]Oth articulr cartilag disor	1
Nyu27	[X]Oth 2ndry gonarthrsis,bilat	1
Nyu24	[X]Oth 2ndry coxarthrsis,bilat	1
NyuB2	[X]Osteoporosis/oth disords CE	1
NyuBC	[X]Osteopenia	1
NyuCA	[X]Osteopathy/other inf dis CE	1
NyuCF	[X]Osteopathy/oth diseases CE	1
NyuCC	[X]Osteonecrosis/other dis CE	1
NyuCB	[X]Osteonecros/h'moglobnpth CE	1
NyuCD	[X]Osteitis defrmn/neop dis CE	1
SR1z1	[X]Op multiple fractures unsp	1
Nyu00	[X]O strep arthritis+polyarthr	1
Nyu38	[X]O spontn disrptn/lig(s)knee	1
Nyu63	[X]O spondylosis+radiculopathy	1
NyuD2	[X]O spf juvnl osteochondrosis	1

Read code	Description	Number of studies
Nyu42	[X]O spcf necrotiz vasculopath	1
Nyu3B	[X]O spcf joint derangmnts,NEC	1
Nyu77	[X]O spcf intrvrtbrl disc diso	1
Nyu94	[X]O spcf diso/synovium+tendon	1
Nyu19	[X]O spcf crystl arthropathies	1
Nyu76	[X]O spc intrvrtbl disc degenr	1
Nyu75	[X]O spc intervert disc displm	1
NyuB7	[X]O spc diso/bne dnsity+struc	1
NyuE0	[X]O spc acq defrm/muscskl sys	1
NyuA3	[X]O sft t d rl/use,overu+prss	1
Nyu11	[X]O sero+ve rheumat arthritis	1
Nyu57	[X]O recur atlantoaxl subluxtn	1
NyuE2	[X]O postproced muscskel disor	1
Nyu04	[X]O postinf arthropath/dis CE	1
NyuC7	[X]O hypertrophc osteoarthrpth	1
Nyu14	[X]O enteropathic arthrpathies	1
NyuB3	[X]O drug-indc osteomalac/adlt	1
Nyu96	[X]O diso/synovm+tendon/dis CE	1
Nyu93	[X]O contractre/tendon(sheath)	1
Nyu40	[X]O cond relt/polyarterit nod	1
NyuC1	[X]O chr h'matogens osteomylit	1
Nyu4D	[X]Necrotis vasculopathy, unsp	1
Nyu87	[X]Myosits/protzl+paras inf CE	1
Nyu88	[X]Myositis/oth infects dis CE	1
Nyu89	[X]Myositis in sarcoidosis CE	1
Nyu86	[X]Myositi/bacterial dis CE	1
Nyu84	[X]Muscle wasting and atrophy NEC	1
SyuA4	[X]Multi disloc/spr/strns,unsp	1
Nyu4F	[X]Mixed connective tissue disease	1
Nyu74	[X]Lumb+o intvt disc d+radiclp	1
Nyu73	[X]Lumb+o intrvrt disc d+mylop	1
Nyu5A	[X]Lordosis, unspecified	1
Nyu16	[X]Juvenile arthritis/o dis CE	1
Syu1	[X]Injuries to the neck	1
Syu3	[X]Injabd/low back/lum sp/pel	1
Syu4	[X]Inj to shoulder/upper arm	1
Syu8	[X]Inj to knee and lower leg	1
Nyu1	[X]Inflammatory polyarthropathies	1
Nyu0	[X]Infectious arthropathies	1
Nyu1C	[X]Gt arthpth/enz d+o inh d CE	1
Syu16	[X]Fracture other parts neck	1
Syu44	[X]Fract should/upp arm unsp	1
Syu15	[X]Fract oth spec cervic vert	1
SyuA2	[X]Fract inv oth comb bod regn	1
NyuAH	[X]Fibroblastic disord, unspec	1
NyuA8	[X]Fasciitis,NEC	1
NyuBB	[X]Erosion of bone	1
SyuA3	[X]Dsl/spr/str,oth comb bod rg	1
SyuB3	[X]Dsl/sp/st un jt/l leg,lv un	1
NyuB	[X]Disordrs/bone dens+structur	1
Nyu3E	[X]Disorder of patella, unspec	1
NyuDE	[X]Disorder cartilage, unspec	1
NyuBA	[X]Disord bone dens/struc,unsp	1

Read code	Description	Number of studies
Syu17	[X]Disloc oth unsp parts neck	1
SyuBC	[X]Disl/spr/str unsp body reg	1
Nyu4E	[X]Dermatopolymyositis, unspec	1
Nyu48	[X]Dermat(poly)myosit/neo d CE	1
Nyu5	[X]Deforming dorsopathies	1
Nyu1D	[X]Crys arthpth/o meta diso,CE	1
Nyu67	[X]Collapsd vertebra in dis CE	1
SR1z0	[X]Clsd multiple fracts unspec	1
NyuD	[X]Chondropathies	1
Nyu7B	[X]Cervical disc disord, unsp	1
Nyu4B	[X]Arthrpthy/hyprsens react CE	1
Nyu1F	[X]Arthrpth/o spcf diseases CE	1
Nyu1E	[X]Arthrpth/o en,nut+meta diso	1
Nyu2	[X]Arthrosis	1
Nyu4A	[X]Arthropathy/o bld disord CE	1
Nyu49	[X]Arthropathy/neoplast dis CE	1
Nyu02	[X]Arthrits/o inf+paras dis CE	1
Nyu01	[X]Arthrit+polyarth/o s bact a	1
NyuB9	[X]Adult osteomalacia, unspec	1
Nyu	[X]Ad muscskl+con t dis cls tm	1
NyuCE	[X]#bone/neoplastic disease CE	1
NyuE1	[X]#/bne f insrt/o i,j pr,bn p	1
EMISREQ|7N945(9)	[SO]Lumbosacral joint -Req.	1
HNGZ018	[RFC] Arthritis	1
R0420	[D]Swelling in head or neck	1
R042	[D]Swell.masslump head/neck	1
R027	[D]Spontaneous bruising	1
R065A	[D]Musculoskeletal chest pain	1
R137	[D]Musculoskel.ray/scan abnorm	1
R137z	[D]Musculosc xray/scan abn NOS	1
R022C	[D]Lump on knee	1
R04zz	[D]Head and neck symptoms NOS	1
R04	[D]Head and neck symptoms	1
R04z	[D]Head and neck other sympt.	1
R00z2-1	[D]General aches and pains	1
R065000	[D]CHEST PAIN, UNSPECIFIED	1
R065z00	[D]CHEST PAIN NOS	1
R065.00	[D]CHEST PAIN	1
R090z00	[D]Abdominal pain	1
R090.00	[D]Abdominal pain	1
DEGRADE_EVENT_1730_49	[DEGRADE Muscle Injury]	1
DEGRADE_EVENT_2469_340	[DEGRADE Knee Pain]	1
DEGRADE_EVENT_3154_40	[DEGRADE Knee Pain]	1
N135z-2	Wry neck	1
N05z3	Wrist osteoarthritis NOS	1
N0943-1	Wrist joint pain	1
N06z3-1	Wrist arthritis NOS	1
S5704	Whiplash injury	1
EGTONWE2	Wedge Compression # Of Dorsal Spine	1
EGTONWE1	Wedge Compression # Lumbar Spine	1
N2432	Weber - Christian disease	1
N2430	Weber - Christian disease	1
N0974	Walking difficulty-other spec.	1

Read code	Description	Number of studies
N0975	Walking difficulty-multip.site	1
N0970	Walking difficulty due to unspecified site	1
N2413	Viral myalgia	1
N092M	Villonodular synovitis of knee	1
N092z	Villonodular synovitis NOS	1
N092	Villonodular synovitis	1
N0920	Villonod.synovitis-site unspec	1
N0921	Villonod.synovitis-shoulder	1
N0928	Villonod.synovitis-other spec.	1
N0929	Villonod.synovitis-mult.sites	1
N2208	Villonod synovitis-tend sheath	1
N092B	Villonod synovitis-sternclav j	1
N092A	Villonod synovitis-shoulder	1
N092C	Villonod synovitis-acromclav j	1
N330B	Vertebral osteoporosis	1
N320	Vertebral epiphysitis	1
N1	Vertebral column syndromes	1
N1y	Vertebral column disorders OS	1
N1z	Vertebral column disorder NOS	1
N0966-2	Unstable knee	1
N0967-1	Unstable ankle	1
N066z	Unspecified monoarthritis NOS	1
N066	Unspecified monoarthritis	1
N1290	Unspec.disc disorder+myelop.	1
N0650	Unsp.polyarthr.-site unspecif.	1
N065000	Unsp.polyarthr.-site unspecif.	1
N0651	Unsp.polyarthr.-shoulder	1
N065800	Unsp.polyarthr.-other specif.	1
N0658	Unsp.polyarthr.-other specif.	1
N065900	Unsp.polyarthr.-multiple site	1
N0659	Unsp.polyarthr.-multiple site	1
N3020	Unsp.osteomyelitis-site unspec	1
N3021	Unsp.osteomyelitis-shoulder	1
N3028	Unsp.osteomyelitis-other spec.	1
N3029	Unsp.osteomyelitis-mult.site	1
N302z	Unsp.osteomyelitis NOS	1
N0660	Unsp.monoarthr.-site unspecif.	1
N0661	Unsp.monoarthr.-shoulder	1
N0668	Unsp.monoarthr.-other specif.	1
N1121	Two lev th spondyl-no myelop	1
N11B1	Two lev th spondyl + radiculop	1
N1131	Two lev th spondyl + myelop	1
N1141	Two lev lumbsac spond-no myelo	1
N1151	Two lev lumbsac spond + myelop	1
N1101	Two lev Cx spondyl-no myelop	1
N1191	Two lev Cx spondyl + radiculop	1
N1111	Two lev Cx spondyl + myelop	1
N018	Tuberculous arthritis	1
N3041	Tuberculosis of thoracic spine	1
N304	Tuberculosis of spine	1
N306	Tuberculosis of other bones	1
N3042	Tuberculosis of lumbar spine	1
N3040	Tuberculosis of cervical spine	1

Read code	Description	Number of studies
N3060	Tuberculosis bone-site unspec.	1
N3061	Tuberculosis bone-shoulder	1
N3064	Tuberculosis bone-other sites	1
N3065	Tuberculosis bone-multip.sites	1
N306z	Tuberculosis bone NOS	1
N22yD	Tuberc infec - tendon sheath	1
N2155	Trochanteric tendinitis	1
N2157	Trochanteric bursitis	1
N0874	Triangular fibrocartilage tear	1
N0875	Triangular fibrocartil detach	1
N118	Traumatic spondylopathy	1
N2312	Traumatic myositis ossificans	1
SE46	Traumatic haematoma	1
SK	Traumatic complicat./unsp.inj.	1
N061A	Traumatic arthropathy-shoulder	1
N061M	Traumatic arthropathy-knee	1
N061z	Traumatic arthropathy NOS	1
N061	Traumatic arthropathy	1
N0610	Traumatic arthr.-site unspecif	1
N0611	Traumatic arthr.-shoulder	1
N0618	Traumatic arthr.-other specif.	1
N0619	Traumatic arthr.-multiple site	1
N061B	Traumat arthrop-sternoclav jt	1
N061C	Traumat arthrop-acromioclav jt	1
S906	Traumat amp at shoulder joint	1
SA72	Traum.unil.amput.>knee-no comp	1
N220Q	Transient synovitis	1
N064A	Transient arthropathy-shoulder	1
N064M	Transient arthropathy-knee	1
N064z	Transient arthropathy NOS	1
N064	Transient arthropathy	1
N064B	Transient arthrop-sternoclav j	1
N0640	Transient arthr.-site unspecif	1
N0641	Transient arthr.-shoulder	1
N0648	Transient arthr.-other specif.	1
N0649	Transient arthr.-multiple site	1
N064C	Transient arthr-acromioclav jt	1
N135	Torticollis unspecified	1
Q20y9	Torticollis due to birth injury	1
16A3	Torticollis - symptom	1
N2451-1	Toe pain	1
N2162	Tibial collateral lig.bursitis	1
N2450-1	Thumb pain	1
N05z4-2	Thumb osteoarthritis NOS	1
N3735	Thoracogenic scoliosis	1
N1485	Thoraco-lumbar ankylosis	1
N144	Thoracic/lumbosacral neuritis	1
N144z	Thoracic/lumbosac.neuritis NOS	1
N112-1	Thoracic spondylosis	1
N112	Thoracic spond.-no myelopathy	1
N113	Thoracic spond.+ myelopathy	1
N148B	Thoracic spine instability	1
N1484	Thoracic spine ankylosis	1

Read code	Description	Number of studies
N1401	Thoracic spinal stenosis	1
N12A2	Thoracic postlaminectomy syndr	1
N1440	Thoracic nerve root pain	1
N12z8	Thoracic discitis	1
N121	Thoracic disc displ.-no myelop	1
N1292	Thoracic disc disord.+myelop.	1
N126	Thoracic disc degeneration	1
S571	Thoracic back sprain	1
EMISNQTH14	Thoracic back pain	1
N245-8	Thigh pain	1
N11B	Th spondyl + radiculop	1
N1406	Th spin stenos due to oth dis	1
N12C1	Th disc prolapse+radiculopathy	1
N12B1	Th disc prolapse + myelopathy	1
N2132-1	Tennis elbow	1
N2202	Tendon sheath giant cell tumor	1
ASDFGTE2	Tendon Symptoms	1
N2456	Tender heel pad	1
S46B	Tear/articulr cart/knee,currnt	1
S545	Tear of ligament of knee joint	1
N000*	Systemic lupus erythematosus	1
N000	Systemic lupus erythematosus	1
N000z	Systemic lupus erythematos.NOS	1
N0012	Syst scleros induc drugs/chems	1
N0003	Syst lup eryth + organ/sys inv	1
N2227	Syphilitic bursitis	1
N2200	Synovitis or tenosynovitis NOS	1
N220z-2	Synovitis of knee	1
N220z	Synovitis of knee	1
N220V	Synovitis of knee	1
N220	Synovitis and tenosynovitis	1
N220T	Synovitis NOS	1
N2201	Synovit./tenosynovitis+dis EC	1
N22y4	Synovial plica	1
N098	Synovial osteochondromatosis	1
N2240	Synovial cyst unspecified	1
N0980	Synov osteochondromat-shoulder	1
N098B	Synov osteochondromat-knee	1
N0981	Synov osteochondromat st-cla j	1
N0982	Synov osteochondromat ac-cla j	1
1D24	Symptom: trunk posterior	1
16J4	Swollen knee	1
16J3	Swollen joint	1
16J7	Swollen foot	1
1834-1	Swollen finger	1
1JG	Suspected inflammatory arthritis	1
N21z2	Supraspinatus tendonitis	1
N2113	Supraspinatus tendinitis	1
N230	Suppurative myositis	1
SD1y4	Supl inj bk NOS-no mj opn wnd	1
SD9	Superficialinjuriesunspecif.	1
SD097	SuperficialInjury:Neck	1
SD2y1	Superficial injury of scapular NOS	1

Read code	Description	Number of studies
SD6y2	Superficial injury of knee NOS	1
SDz	Superficial injuries NOS	1
SD0	Superficial Injury: Neck	1
SD2y0	Superfic injury shoulder NOS	1
SD2	Superf.inj.shoulder/upper arm	1
SD1z4	Superf.back inj.NOS+infect.	1
N3371	Sudek's atrophy	1
N2167	Subpatellar bursitis	1
N3y01	Subluxatn complex (vertebral)	1
N3y02	Sublux stenos of neural canal	1
N3081	Subacute osteomyelitis-th spin	1
N3082	Subacute osteomyelitis-lu spin	1
N3080	Subacute osteomyelitis-Cx spin	1
N309	Subacute osteomyelitis	1
N308	Subacute osteomyelitis	1
N2122	Subacromial impingement	1
N2116	Subacromial bursitis	1
S3z2	Stress fracture	1
N095B	Stiff sternoclavic joint NEC	1
N095A	Stiff shoulder NEC	1
16AZ	Stiff neck symptom NOS	1
16A	Stiff neck symptom	1
N135z	Stiff neck NOS	1
16A2	Stiff neck	1
N095M	Stiff knee NEC	1
N095C	Stiff acromioclavicular joint NEC	1
S5y41	Sternoclavicular sprain	1
N0108	Staphylococc arthrit/polyarthr	1
S5410	Sprn,knee jt,medial collat	1
S5400	Sprn,knee jt,lat collat lgmt	1
S520D	Sprn triangular fibrocartilage	1
S520G	Sprn shrt intrnsc lgmnt non-sp	1
S5	Sprains and strains	1
S50	Sprained shoulder	1
S54y-99	Sprained knee NOS	1
S54-99	Sprained knee	1
S560	Sprain, lumbosacral ligament	1
S564	Sprain, iliolumbar ligament	1
S501	Sprain, coraco-clav ligament	1
S506	Sprain supraspinatus tendon	1
S505	Sprain subscapularis tendon	1
S5071	Sprain shoulder joint posterior	1
S5070	Sprain shoulder joint anterior	1
S507	Sprain shoulder joint	1
S534	Sprain patellar tendon	1
S503	Sprain infraspinatus tendon	1
S500	Sprain acromio-clav ligament	1
S542	Sprain -cruciate knee ligament	1
S541	Sprain - medial knee ligament	1
S541-99	Sprain - medial knee ligament	1
S540-99	Sprain - lateral knee ligament	1
S540	Sprain - lateral knee ligament	1
SC07	Sprain - late effect	1

Read code	Description	Number of studies
S57X	Spr/str ot/un pt lum sp/pel	1
ASDFGSP5	Sports Injury	1
N082	Spontaneous joint dislocation	1
16B3	Spontaneous bruising	1
N11z0	Spondylosis-no myelopathy,NOS	1
N11zz	Spondylosis NOS	1
N11z1	Spondylosis + myelopathy, NOS	1
N388	Spondylolysis	1
OXL7561C	Spondylolisthesis /ox	1
N10z	Spondylitis NOS	1
N374	Spine curvature+other condits.	1
N374z	Spine curvature+other cond.NOS	1
N1400	Spinal stenosis unspec.region	1
N140z	Spinal stenosis NOS	1
N140-1	Spinal stenosis	1
N140	Spinal stenosis	1
N101	Spinal enthesopathy	1
N222z	Specific bursitides NOS	1
N222	Specific bursitides	1
F1382	Spasmodic Torticollis	1
N23y4	Spasm of muscle	1
N23yE	Spasm of back muscles	1
EGTON309	Sore Neck	1
N3321	Solitary bone cyst	1
N0878	Snapping shoulder	1
N32y	Slipped radial epiphysis	1
N33zA	Skeletal fluorosis	1
N002*	Sicca (Sjogren's) syndrome	1
N2125	Shoulder tendonitis	1
S50y	Shoulder sprain NOS	1
N245-7	Shoulder pain	1
N245	Shoulder pain	1
N2457	Shoulder pain	1
N0951	Shoulder joint stiffness	1
UNMAPPC0	Shoulder injury	1
S221	Shoulder fracture - open	1
N22y5	Short tendon	1
N011	Sex acquired reactive arthrop	1
N0110	Sex acqd reac arthrop-unspec	1
N0111	Sex acqd reac arthrop-shoulder	1
N011y	Sex acqd reac arthrop-oth spec	1
N011x	Sex acqd reac arthrop-multiple	1
N011z	Sex acq reac arthropathy NOS	1
SRz0	Severe multiple injuries	1
N047	Seropositive errosive RA	1
N04X	Seroposit rheum arthr unsp	1
N040P	Seronegative rheumat arthritis	1
EGTONSE2	Sero-Negative Polyarthritis	1
N04y1	Sero negative arthritis	1
N04y10	Sero negative arthritis	1
N3301	Senile osteoporosis	1
N2169	Semimembranosus tendinitis	1
EGTON131	Semi Frozen Shoulder	1

Read code	Description	Number of studies
N3y00	Segmental & somatic dysfunctn	1
N0505	Secondary multiple arthrosis	1
N050500	Secondary multiple arthrosis	1
EMISNQSC20	Scoliosis of thoracic spine	1
N374A	Scoliosis in skelet dysplasia	1
N374C	Scoliosis in neurofibromatosis	1
N374D	Scoliosis in conn tiss anomal	1
N3739	Scoliosis due to oth treatment	1
OX735AA	Scoliosis Acquired /ox	1
N3743	Scoliosis + other condition	1
N001	Scleroderma	1
OX353C	Sciatica Chronic /ox	1
N1240	Schmorl's nodes-unspec. region	1
N1241	Schmorl's nodes-thoracic regn.	1
N124z	Schmorl's nodes-region NOS	1
N1242	Schmorl's nodes-lumbar region	1
N124	Schmorl's nodes	1
N3201	Scheuermann's disease	1
N2121	Scapulohumeral fibrositis	1
N146z-1	Sacroiliac strain	1
N1466	Sacroiliac disorder	1
S5731	Sacral/coccyx sprain	1
N0004	SLE with pericarditis	1
N25	SAPHO syndrome	1
S5Q2	Rupture supraspinatus tendon	1
S5Q1	Rupture subscapularis tendon	1
S5U2	Rupture patellar tendon	1
N2250	Rupture of synovium unspecif.	1
N225z	Rupture of synovium NOS	1
N2251-99	Rupture of synovium - knee	1
N225	Rupture of synovium	1
S5Q0	Rupture infraspinatus tendon	1
N2251	Ruptur poplit space synov cyst	1
N2110	Rotator cuff syndrome unspecif	1
S504	Rotator cuff sprain	1
N211	Rotator cuff shoulder syndrome	1
N2261	Rotator cuff complete rupture	1
N3385	Rotational mal-union of #	1
182B	Rib pain	1
EGTON425	Rheumatology	1
N040N	Rheumatoid vasculitis	1
N0422	Rheumatoid nodule	1
N040R	Rheumatoid nodule	1
N0421	Rheumatoid lung disease	1
N040Q	Rheumatoid bursitis	1
N0402	Rheumatoid arthritis-shoulder	1
N0400	Rheumatoid arthritis-Cx spine	1
N040D	Rheumatoid arthritis of knee	1
N040	Rheumatoid arthritis	1
N0403	Rheumatoid arthr-sternoclav jt	1
N0404	Rheumatoid arthr-acromioclav j	1
N1351	Rheumatic torticollis	1
N0420	Rheumatic carditis	1

Read code	Description	Number of studies
OX7149A	Rheumatic Arthritis /ox	1
N040S	Rheumat arthr - multiple joint	1
N2333	Rhabdomyolysis	1
N042z	Rh.arthr.+visc/syst.dis.NOS	1
N080A	Reverse Hill-Sachs lesion	1
N0871	Reverse Bankart lesion	1
N12zA	Resorption of thoracic disc	1
N12zE	Resorption of lumbar disc	1
N12z6	Resorption of cervical disc	1
N3732	Resolving infant.idiopath.scol	1
N246	Residual soft tiss.foreign bod	1
N339	Residual foreign body in bone	1
Ny2	Repetitive strain injury	1
N33z5	Relapsing polychondritis	1
N337	Reflex sympathetic dystrophy	1
N083	Redislocation of joint	1
S46D	Recurrent subluxation of patella	1
N083D	Recurrent sublux shoulder-post	1
N083F	Recurrent sublux shoulder-inf	1
N083C	Recurrent sublux shoulder-ant	1
N083H	Recurrent sublux shoulder-ant	1
N083q	Recurrent sublux - patella	1
N083z	Recurrent joint dislocat.NOS	1
N0839	Recurrent disloc-multip joints	1
N083B	Recurrent disloc shoulder-post	1
N083E	Recurrent disloc shoulder-inf	1
N083A	Recurrent disloc shoulder-ant	1
N083G	Recurrent disloc shoulder-ant	1
N083p	Recurrent disloc - patella	1
N083n	Recurrent disloc - knee	1
N0830	Recurr.joint disloc.-site unsp	1
N0838	Recurr.joint disloc-other spec	1
N0831	Recur. disloc.- shoulder joint	1
N0836-99	Recur. disloc. - knee joint	1
N083K	Recur sublux shoulder-multidir	1
N083J	Recur disloc shoulder-multidir	1
N1y0	Rec atlantoax subl + myelopath	1
N01w0	Reactive arthropathy-shoulder	1
N01w	Reactive arthropathy unspecified	1
N01wB	Reactive arthropathy of knee	1
N038	Reactive arthropathies	1
N01w2	Reactive arthrop-sternoclav jt	1
N01w1	Reactive arthr-acromioclav jt	1
EGTON436	Radiculopathy	1
N2422	Radiculitis unspecified	1
N3734	Radiation scoliosis	1
N3711	Radiation kyphosis	1
N0706	Radial tear of medial meniscus	1
N0717	Radial tear of lateral meniscus	1
N2204	Radial styloid tenosynovitis	1
N22yC	Pyogenic infec - tendon sheath	1
N010	Pyogenic arthritis	1
N0100	Pyogenic arthr.-site unspecif.	1

Read code	Description	Number of studies
N0101	Pyogenic arthr.-shoulder regn.	1
N010y	Pyogenic arthr.-other specif.	1
N010x	Pyogenic arthr.-multiple sites	1
N010z	Pyogenic arthr.-NOS	1
S57z0	Pulled back muscle	1
OX848ML	Pulled Muscle /ox	1
E2601	Psychogenic Torticollis	1
N2373	Pseudosarcomatous fibromatosis	1
N33zC	Pseudarth after fusn/arthrodes	1
S5422	Prt tr,knee,post cruciate lgmt	1
N12C4	Prol lumb interv disc sciatic	1
N0010	Progressive systemic sclerosis	1
N2311	Progressive myositis ossific.	1
N3733	Progressive infant.idiop.scol.	1
N051B	Primary gonarthrosis, bilat	1
N050400	Primary general osteoarthrosis	1
N0504	Primary general osteoarthrosis	1
N0519	Primary coxarthrosis bilateral	1
N051C	Primary arthrosis of first carpometacarpal joints, bilateral	1
N2165	Prepatellar bursitis	1
N3736	Postural scoliosis	1
N3307	Postsurg malabsorp osteoporos	1
N3314	Postsur malab osteop+path frct	1
NyX	Postproc muscsk disord,unsp	1
N2313	Postop.heterotopic calcificat.	1
N3306	Postoophorectomy osteoporosis	1
N3312	Postoophorc osteopor+path frct	1
N3302	Postmenopausal osteoporosis	1
N331B	Postmenop osteopor+path fract	1
N0380	Postmeningococcal arthritis	1
N12Az	Postlaminectomy syndrome NOS	1
N12A	Postlaminectomy syndrome	1
N12A0	Postlaminectomy syndr.unspec.	1
N0381	Postinf arthropath in syphilis	1
N037	Postimmunization arthropathy	1
N013	Postdysenteric react arthrop	1
N0130	Postdys react arthrop-unspec	1
N0131	Postdys react arthrop-shoulder	1
N013y	Postdys react arthrop-oth spec	1
N013x	Postdys react arthrop-multiple	1
N013z	Postdys react arthrop NOS	1
16B4	Post-traumatic bruising	1
N052B	Post-traumatic arthrosis of first carpometacarpal joints, bilateral	1
N052C	Post-trauma gonarth, unilat	1
N052A	Post-traum gonarthrosis, bilat	1
N0529	Post-traum coxarthrosis, bilat	1
N3738	Post-surgical scoliosis	1
N3721	Post-laminectomy lordosis	1
N3712	Post-laminectomy kyphosis	1
N2314	Polymyositis ossificans	1
N004	Polymyositis	1

Read code	Description	Number of studies
N20*	Polymyalgia rheumatica	1
N20	Polymyalgia	1
N065-1	Polyarthropathy NEC	1
N065.11	Polyarthropathy NEC	1
N065	Polyarthropathy NEC	1
N065z	Polyarthritis	1
N065z00	Polyarthritis	1
N307z	Poliomyelitis osteopathy NOS	1
N3070	Polio.osteopathy-site unspecif	1
N3071	Polio.osteopathy-shoulder	1
N3078	Polio.osteopathy-other sites	1
N3079	Polio.osteopathy-multiple site	1
N0109	Pneumococc arthrit & polyarthr	1
N2179	Plantar fasciitis	1
N2209	Plant thorn synovitis	1
N2161	Pes anserinus tendin./bursitis	1
SJz-98	Peripheral nerve injury NOS	1
N21zz	Peripheral enthesopathy NOS	1
N21	Peripheral enthesopathies	1
N0705	Periph detach-medial meniscus	1
N0716	Periph detach-lateral meniscus	1
N303	Periostitis, no osteomyelitis	1
N303B	Periostitis, no osteomye-th sp	1
N303C	Periostitis, no osteomye-lu sp	1
N303A	Periostitis, no osteomye-Cx sp	1
N303z	Periostitis no osteomyel NOS	1
N3030	Periostitis - site unspecified	1
N3031	Periostitis - shoulder	1
N3038	Periostitis - other sites	1
N3039	Periostitis - multiple sites	1
N2120	Periarthritis of shoulder	1
N21z1	Periarthritis NOS	1
N3843	Pedicular spondylolisthesis	1
N0432	Pauciarticular juvenile R.A.	1
N0456	Pauciartic onset juv ch arth	1
N331y	Pathological fracture OS	1
N331z	Pathological fracture NOS	1
N082z	Pathological dislocation NOS	1
N331C	Pathological # cervical vert	1
N0820	Patholog.disloc.-site unspecif	1
N0821	Patholog.disloc.-shoulder	1
N0828	Patholog.disloc.-other specif.	1
N082A	Path disloc-shoulder joint	1
N082R	Path disloc-patellofem joint	1
N082B	Path disloc-oth joint-shoulder	1
N0829	Path disloc-multiple joints	1
N082Q	Path disloc-knee joint	1
N0536-1	Patellofemoral osteoarthritis	1
N0536	Patellofemoral osteoarthritis	1
N07y6	Patellofemoral maltracking	1
N09A	Patellofemoral disorder	1
N2266	Patellar tendon nontraum.rupt.	1
N2164	Patellar tendinitis	1

Read code	Description	Number of studies
N33z9	Partial epiphyseal arrest	1
S5421	Part tr,knee,ant cruciate lgmt	1
N2114	Part thickn rotator cuff tear	1
S5411	Part tear,knee,mdl collat lgmt	1
S5401	Part tear,knee,lat collat lgmt	1
N1y2	Pars interarticular strss frct	1
N0704	Parr beak tear-post/med menisc	1
N0715	Parr beak tear-post/lat menisc	1
N2316	Paralytic calcific/ossif muscl	1
N243	Panniculitis unspecified	1
N136	Panniculitis of neck	1
N243z	Panniculitis NOS	1
N093z	Palindromic rheumatism NOS	1
N093	Palindromic rheumatism	1
N0930	Palindromic rheum.-site unspec	1
N0931	Palindromic rheum.-shoulder	1
N0938	Palindromic rheum.-other spec.	1
N0939	Palindromic rheum.-multip.site	1
N211z	Painful arc syndrome	1
N211z-1	Painful arc syndrome	1
EGTON224	Painful Shoulder	1
EGTON279	Painful Right Knee	1
N131-1	Pain in cervical spine	1
N245-9	Pain in buttock	1
N2453	Pain in arm	1
OX7280AD	Pain Neck /ox	1
OX7873E	Pain Knee /ox	1
N245-95	Pain In Right Leg	1
N245-97	Pain In Right Arm	1
N245-96	Pain In Left Leg	1
N3101	Paget's disease-thoracic spine	1
N3106	Paget's disease-scapula	1
N310F	Paget's disease-patella	1
N310x	Paget's disease-multiple sites	1
N3102	Paget's disease-lumbar spine	1
N3105	Paget's disease-clavicle	1
N3100	Paget's disease-cervical spine	1
N310y	Paget's disease OS	1
N310z	Paget's disease NOS	1
N122-1	PID - prolapsed lumbar disc	1
N129	PID - prol i/v disc + myelop	1
S102y	Othr spec clsd # thorac vert	1
S411y	Othr opn trmtc disloctn shlder	1
S410y	Othr cls trmtc disloc shoulder	1
SK12z	Othershould/upperarminj.NOS	1
SE08	Othercontusionneck	1
N06..00	Other/unspecif. arthropathies	1
N12z2	Other thoracic disc disorders	1
N22	Other synovium/tendon/bursa	1
N22y	Other synovium/tendon/bursa	1
N096B	Other symptoms - sternoclav jt	1
N096A	Other symptoms - shoulder	1
N096M	Other symptoms - knee	1

Read code	Description	Number of studies
N096D	Other symptoms - elbow	1
N096C	Other symptoms - acromioclav j	1
SD9y	Other superficial injury, without mention of infection, NOS	1
S5y	Other sprains and strains	1
S5yz	Other sprains NOS	1
N11y	Other spondyloses/allied dis.	1
S5W	Other specified tendon rupture	1
S54w	Other specified knee sprain	1
SK1	Other specified injury	1
N06y	Other specified arthropathy	1
N233	Other specific muscle disorder	1
N04y	Other specif.infl.polyarthrop.	1
N32yz	Other spec.osteochondrop.NOS	1
N04yz	Other spec.infl.polyarthr.NOS	1
N00y	Other spec.diff.collagen dis.	1
N06y0	Other spec.arthr.-site unspec.	1
N06y1	Other spec.arthr.-shoulder	1
N06y8	Other spec.arthr.-other specif	1
S5yy	Other spec sprains and strains	1
S50w	Other shoulder sprain	1
SK122	Other shoulder injuries	1
N212	Other shoulder affections NEC	1
N212z	Other shoulder affect.NEC NOS	1
SK121	Other scapular region injuries	1
N042	Other rh.arthr.+visc/syst.dis.	1
N3722	Other post-surgical lordosis	1
N21y	Other periph. enthesopathies	1
N3272	Other osteochondr dissec-knee	1
S466	Other opn trm dslctn knee	1
S497	Other open trmtc dislocation	1
S49Fz	Other open subluxation NOS	1
S4J3	Other open #-subluxation	1
S4J1	Other open #-dislocation	1
N226z	Other nontraumatic tendon rupt	1
SK10y	Other neck injuries	1
SK1x	Other multiple injuries	1
N12z3	Other lumbar disc disorders	1
N07yy	Other knee lig. old disruption	1
SK170	Other knee injury	1
N368	Other knee deformity	1
N045	Other juvenile arthritis	1
N326	Other juven.osteochondroses	1
N0960	Other joint sympt.-site unspec	1
N0961	Other joint sympt.-shoulder	1
N09y1	Other joint dis.-shoulder	1
N08yz	Other joint derange.NEC NOS	1
SK112	Other interscapular injuries	1
N07yz	Other intern.knee derang.NOS	1
SK1z	Other injury NOS	1
N10y	Other inflamm.spondylopathies	1
N10yz	Other inflamm.spondylop.NOS	1
N30y	Other infections+bone disease	1

Read code	Description	Number of studies
N03x	Other general dis.+arthropathy	1
N237z	Other fibromatoses NOS	1
N237	Other fibromatoses	1
SK10z	Other face and neck injuries NOS	1
S49z	Other dislocation NOS	1
N12z0	Other disc disorders unspecif.	1
N08	Other derangement of joint	1
N36y	Other deformity of bone	1
N37y	Other curvatures of spine	1
N02yz	Other crystal arthropathy NOS	1
N02y	Other crystal arthropathies	1
N02y0	Other crystal arth.-site unsp.	1
N02y1	Other crystal arth.-shoulder	1
N02yy	Other crystal arth.-other spec	1
N02yx	Other crystal arth.-mult.sites	1
S465	Other cls trm dslctn knee	1
S49E	Other closed traumatic sublux	1
S4J2	Other closed #-sublux	1
S4J0	Other closed #-dislocation	1
N13yz	Other cervical syndromes NOS	1
N13y	Other cervical syndromes	1
N12z1	Other cervical disc disorders	1
N33	Other bone/cartilage disorders	1
N31y	Other bone involve.in dis.EC	1
SK114	Other back injuries	1
N374X	Other and unspecified kyphosis	1
N06	Other and unspecified arthropathies	1
S462	Other acute meniscus tear	1
N38yz	Other acquired deformity NOS	1
N38y	Other acquired deformity	1
N38	Other acquired deformity	1
S4J	Other #-dslc or subluxation	1
N08y0	Oth.joint deran.NEC-site unsp.	1
N08y1	Oth.joint deran.NEC-shoulder	1
N08y8	Oth.joint deran.NEC-other spec	1
N08y9	Oth.joint deran.NEC-mult.sites	1
N30y0	Oth.inf.+bone dis-site unspec.	1
N30y1	Oth.inf.+bone dis-shoulder	1
N30y8	Oth.inf.+bone dis-other sites	1
N30y9	Oth.inf.+bone dis-multip.site	1
N30yz	Oth.inf.+bone dis-NOS	1
N07y	Oth. internal knee derangement	1
S49	Oth, mlti+ill-def dislc/sublux	1
N0401	Oth rheumatoid arthritis-spine	1
S49F	Oth open traumatic subluxation	1
SRy	Oth inj inv mult body reg NEC	1
S496	Oth cls trmatic dislocation	1
S49Ez	Oth closed subluxation NOS	1
N33B	Osteoradionecrosis	1
N3746	Osteoporotic kyphosis	1
N3300	Osteoporosis unspecified	1
N330C	Osteoporosis localized spine	1
N330A	Osteoporosis in endocr disord	1

Read code	Description	Number of studies
N330z	Osteoporosis NOS	1
N330	Osteoporosis	1
N330D	Osteoporos due corticosteroid	1
N3309	Osteopor,multiple myelomatosis	1
N3319	Osteopor path # thor vertebrae	1
N3318	Osteopor path # lumb vertebrae	1
N331A	Osteopor path # cerv vertebrae	1
N3313	Osteopor of disuse + path frct	1
N09B	Osteophyte	1
N307	Osteopathy from poliomyelitis	1
ASDFGOS1	Osteopaenia	1
N334B	Osteonecrosis in caisson dis	1
N3349	Osteonecrosis due to drugs	1
N334A	Osteonecr due to prev trauma	1
N334C	Osteonecr due haemoglobinopath	1
N30	Osteomyelitis/periostitis	1
N302a	Osteomyelitis of vertebra	1
N302	Osteomyelitis NOS	1
N33zH	Osteolytic lesion	1
N33zD	Osteolysis	1
N32z3	Osteochondrosis NOS	1
N32z	Osteochondropathy NOS	1
N32zz	Osteochondropathy NOS	1
N32	Osteochondropathies	1
N32z2	Osteochondritis of knee	1
N3270	Osteochondritis dissec-patella	1
N327y	Osteochondr dissec-other site	1
N3274	Osteochondr dissec-capitellum	1
N327	Osteochond dissecans	1
OX7130E	Osteoarthrosis Shoulder /ox	1
OX7130B	Osteoarthrosis Knee(S) /ox	1
OX7131A	Osteoarthrosis Cervical Spine /ox	1
N11z	Osteoarthritis spine	1
N11D1	Osteoarthritis of thoracic spine	1
N11D3	Osteoarthritis of spine NOS	1
N11-2	Osteoarthritis of spine	1
N11D	Osteoarthritis of spine	1
N11D2	Osteoarthritis of lumbar spine	1
N11D0	Osteoarthritis of cervical spine	1
N110-2	Osteoarthritis cervical spine	1
N05z0	Osteoarthritis NOS-site unspec	1
N05z000	Osteoarthritis NOS-site unspec	1
N05z7	Osteoarthritis NOS-ankle/foot	1
N05zM	Osteoarthritis NOS, of tibio-fibular joint	1
N05zQ	Osteoarthritis NOS, of talonavicular joint	1
N05zK	Osteoarthritis NOS, of sacro-iliac joint	1
N05zR	Osteoarthritis NOS, of other tarsal joint	1
N05zF	Osteoarthritis NOS, of metacarpophalangeal joint	1
N05zT	Osteoarthritis NOS, of lesser metatarsophalangeal joint	1
N05zU	Osteoarthritis NOS, of interphalangeal joint of toe	1
N05zD	Osteoarthritis NOS, of distal radio-ulnar joint	1
N05zE	Osteoarthritis NOS of wrist	1

Read code	Description	Number of studies
N05z4	Osteoarthritis NOS of the hand	1
N05z9	Osteoarthritis NOS of shoulder	1
N05zL	Osteoarthritis NOS of knee	1
N05zJ	Osteoarthritis NOS of hip	1
N05zC	Osteoarthritis NOS of elbow	1
N05zN	Osteoarthritis NOS of ankle	1
N05zz00	Osteoarthritis NOS	1
N05zz	Osteoarthritis NOS	1
N05z1	Osteoarthritis -shoulder joint	1
N05z800	Osteoarthritis - other joint	1
N05z8	Osteoarthritis - other joint	1
N05z6-99	Osteoarthritis - knee joint	1
N05..11	Osteoarthritis	1
N094K	Osteoarthritis	1
N05	Osteoarthritis	1
N310	Osteitis deformans-Paget's dis	1
N311	Osteitis deformans+disease EC	1
N3350	Osteitis condensans ilii	1
N335	Osteitis condensans	1
N31	Osteit.deform./osteop.+dis.EC	1
N3110	Osteit deformans,neoplast dis	1
N3y03	Osseous stenos of neural canal	1
N3y06	Oss/sublx sten intervert foram	1
S900z	Opn wound shoulder+up limb,NOS	1
SA10	Opn wnd kneelg+ank-no cmplctn	1
S46A6	Opn trmtc sublux,head fibula	1
S413z	Opn trmtc sublux shoulder NOS	1
S468	Opn trmtc sublux pat-fem jt	1
S46A0	Opn trmtc sublux knee jt,unsp	1
S46A2	Opn trmtc sublux knee jt,post	1
S46A4	Opn trmtc sublux knee jt,ltrl	1
S46A1	Opn trmtc sublux knee jt,ant	1
S46A	Opn trmtc sublux knee jt	1
S4112	Opn trmtc dslctn shldr jt,post	1
S411z	Opn trmtc disloctn shouldr NOS	1
S4110	Opn trmtc dislctn shoulder jnt	1
S49F4	Opn trm sublux,st-clav jt,post	1
S49F3	Opn trm sublux,st-clav jt,ant	1
S49F2	Opn trm sublux st-clav jt	1
S4681	Opn trm sublux pat-fem jt,med	1
S4680	Opn trm sublux pat-fem jt,ltrl	1
S49F5	Opn trm sublux laryngl cartlge	1
S46A5	Opn trm sublux knee jt,rotatry	1
S46A3	Opn trm sublux knee jt,medial	1
S4131	Opn trm sublux acromio-clav jt	1
S4666	Opn trm dslctn, head fibula	1
S464	Opn trm dslctn patello-fem jt	1
S4641	Opn trm dslctn pat-fem jt,med	1
S4640	Opn trm dslctn pat-fem jt lat	1
S4660	Opn trm dslctn knee, unspec	1
S4664	Opn trm dslctn knee jt,lateral	1
S4662	Opn trm dslctn knee jt, post	1
S4663	Opn trm dslctn knee jt, medial	1

Read code	Description	Number of studies
S4661	Opn trm dslctn knee jt, ant	1
S4665	Opn trm dslct knee jt,rotatory	1
S4973	Opn trm dslc,stern-clav jt,ant	1
S4972	Opn trm dslc sterno-clav jt	1
S4974	Opn trm dsl,stern-clav jt,post	1
S4114	Opn trm dislc acromio-clav jt	1
S413	Opn traumtc subluxatn shoulder	1
S4130	Opn traumtc sublux shouldr jnt	1
S411	Opn traumtc disloctn shoulder	1
S4115	Opn traumatic disloctn scapula	1
S49B	Opn sublux thrcic+lmbr vertbra	1
S49B1	Opn sublux thoracic spine	1
S499x	Opn sublux mlti cerv vertebrae	1
S499z	Opn sublux cerv vertebra NOS	1
S4991	Opn sublux atlanto-occipitl jt	1
S493C	Opn spnl dslc+cauda equina lsn	1
S4934	Opn spnl dslc+ant thrc crd lsn	1
S4939	Opn spnl dslc+ant lmbr crd lsn	1
S491B	Opn spnl dslc+ant cerv crd lsn	1
S493B	Opn spnl dsl+post lmbr crd lsn	1
S491D	Opn spnl dsl+post cerv crd lsn	1
S4933	Opn spnl dsl+comp thrc crd lsn	1
S4938	Opn spnl dsl+comp lmbr crd lsn	1
S4919	Opn spnl dsl+cerv crd lsn,unsp	1
S4932	Opn spn dslc+thrc crd lsn,unsp	1
S4936	Opn spn dslc+post thrc crd lsn	1
S491C	Opn spn dslc+ctrl cerv crd lsn	1
S491A	Opn spn dslc+comp cerv crd lsn	1
S4935	Opn spn dslc+cent thrc crd lsn	1
S493A	Opn spn dslc+cent lmbr crd lsn	1
S1150	Opn spn # + unsp lumb crd lesn	1
S1154	Opn spn # + post lumb crd lesn	1
S1151	Opn spn # + comp lumb crd lesn	1
S1153	Opn spn # + cent lumb crd lesn	1
S1155	Opn spn # + cauda equina lesn	1
S1152	Opn spn # + ant lumbr crd lesn	1
S5P1z	Opn dvsn,thyroid regn lgmt NOS	1
S5P1	Opn dvsn,thyroid region lgmt	1
S5P12	Opn dvsn,thyroid cartilge lgmt	1
S5P30	Opn dvsn,sternoclavicular lgmt	1
S5N	Opn dvsn,lgmt other part back	1
S5P11	Opn dvsn,cricothyroid ligament	1
S5P10	Opn dvsn,cricoarytenoid lgmt	1
S5K1	Opn dvsn mdl collat lgmt knee	1
S5K0	Opn dvsn lat collat lgmt knee	1
S5F1	Opn dvsn coracoclavicular lgmt	1
S5F0	Opn dvsn acromioclavic lgmt	1
S5K2	Opn dvs post cruciate lgm knee	1
S5K3	Opn dvs ant cruciate lgmt knee	1
S4931	Opn dslc thoracic spine	1
S4975	Opn dslc laryngl cartilage	1
S1034	Opn # thorc vert-trnsvrse prcs	1
S1032	Opn # thorc vert-spondylolysis	1

Read code	Description	Number of studies
S1033	Opn # thorc vert-spinous prcs	1
S1036	Opn # thor vert-tricolumnar	1
S1035	Opn # thor vert-posterior arch	1
S1055	Opn # lumb vert,posterior arch	1
S1054	Opn # lumb vert, trnsvrse prcs	1
S1056	Opn # lumb vert, tricolumnar	1
S1052	Opn # lumb vert, spondylolysis	1
S1053	Opn # lumb vert, spinous prcs	1
S101L	Opn # cerv vert, trnsvrse prcs	1
S101N	Opn # cerv vert, tricolumnar	1
S101J	Opn # cerv vert, spondylolysis	1
S101K	Opn # cerv vert, spinous prcs	1
S101M	Opn # cerv vert, post arch	1
S101D	Opn # axis, trnsvrse process	1
S1018	Opn # atlas-isol arch/art prcs	1
S89z	Open wounds NOS	1
S90	Open wound shoulder/upper limb	1
S9020	Open wound shoulder+tendon inv	1
S9010	Open wound shoulder+complicat.	1
S9021	Open wound scapular+tendon inv	1
S900	Open wound of shoulder/upper limb without complication	1
S9000	Open wound of shoulder region	1
S87	Open wound of sacroiliac reg.	1
S84	Open wound of neck	1
SA100	Open wound of knee	1
S86	Open wound of back	1
SA110	Open wound knee+complication	1
S8z	Open wound head/neck/trunk NOS	1
S8	Open wound head/neck/trunk	1
S113z	Open thoracic #+cord lesn.NOS	1
S113	Open thoracic #+cord lesion	1
S49Dz	Open subluxation of spine NOS	1
S49B0	Open subluxation lumbar spine	1
S4998	Open subluxation C7/T1	1
S4997	Open subluxation C6/C7	1
S4996	Open subluxation C5/C6	1
S4995	Open subluxation C4/C5	1
S4994	Open subluxation C3/C4	1
S4993	Open subluxation C2/C3	1
S49D0	Open sublux spine, unspecified	1
S49D	Open sublux other vertebra	1
S4990	Open sublux cerv spine, unsp	1
S499	Open sublux cerv spine	1
S4992	Open sublux atlanto-axial jt	1
S49y	Open multiple/ill-def.disloc.	1
S2921	Open mult fract clav scap hum	1
S115	Open lumbar # + cord lesion	1
S2115	Open fracture scapula, spine	1
S2116	Open fracture scapula, neck	1
S2113	Open fracture scapula, glenoid	1
S2114	Open fracture scapula, blade	1
S321	Open fracture of the patella	1

Read code	Description	Number of studies
S105	Open fracture lumbar vertebra	1
S1260	Open fracture larynx	1
S2012	Open fracture clavicle, shaft	1
S101	Open fracture cervical spine	1
S1012	Open fracture axis	1
S1011	Open fracture atlas	1
S5N0	Open dvsn, neck ligament	1
S5Fz	Open dvsn shoulder lgmt NOS	1
S4911	Open dslc atlanto-occipital jt	1
S4912	Open dslc atlanto-axial jt	1
S5Nz	Open divisn, back ligament NOS	1
S5N1	Open division,thoracic lgmt	1
S5P	Open division, other ligament	1
S5Pz	Open division, other lig NOS	1
S5N2	Open division, lumbar ligament	1
S5F	Open division shoulder lgmt	1
S5Ky	Open division other knee lgmt	1
S5M5	Open division lumbosacral lgmt	1
S5K	Open division ligament knee	1
S5Kz	Open division knee lgmt NOS	1
S5M4	Open division iliolumbar lgmt	1
S4950	Open dislocation spine unspec.	1
S495z	Open dislocation spine NOS	1
S491	Open dislocation of neck	1
S4930	Open dislocation lumbar spine	1
S466z	Open dislocation knee NOS	1
S497z	Open dislocation NOS	1
S4918	Open dislocation C7/T1	1
S4917	Open dislocation C6/C7	1
S4916	Open dislocation C5/C6	1
S4915	Open dislocation C4/C5	1
S4914	Open dislocation C3/C4	1
S4913	Open dislocation C2/C3	1
S493	Open disloc.thoracic/lumbar	1
S493z	Open disloc.thorac./lumbar NOS	1
S495	Open disloc.other vertebra	1
S491x	Open disloc.mult.cerv.vertebra	1
S491z	Open disloc.cervical vert.NOS	1
S4910	Open disloc.cerv.spine unspec.	1
S111z	Open cervical#+cord lesion NOS	1
S111	Open cervical #+cord lesion	1
S1263	Open #trachea	1
S1262	Open #thyroid cartilage	1
S11y	Open #spine+cord lesion unsp.	1
S2110	Open #scapula-unspecified	1
S211z	Open #scapula NOS	1
S126z	Open #larynx/trachea NOS	1
S1261	Open #hyoid bone	1
S2010	Open #clavicle unspecified	1
S201z	Open #clavicle NOS	1
S3z1	Open #bones unspecified	1
S4F7	Open #-sublux,patello-fem jt	1
S4J33	Open #-sublux st-clav jt,post	1

Read code	Description	Number of studies
S4J32	Open #-sublux st-clav jt,ant	1
S4A30	Open #-sublux shoulder joint	1
S4A3	Open #-sublux shoulder	1
S4F3	Open #-sublux knee joint	1
S4A31	Open #-sublux acrom-clav joint	1
S4F5	Open #-dslc,patello-fem jt	1
S4F1	Open #-dslc, knee joint	1
S4J13	Open #-dslc st-clav jt,post	1
S4J12	Open #-dslc st-clav jt,ant	1
S4A10	Open #-dslc shoulder joint	1
S4A1	Open #-dslc shoulder	1
S4A11	Open #-dslc acrom-clav joint	1
S1010	Open # unsp cerv vertebra	1
S103	Open # thoracic vertebra	1
S1031	Open # thorac vert, wedge	1
S1030	Open # thorac vert, burst	1
S1013	Open # third cerv vertebra	1
S10y	Open # spine, unspecif	1
S1016	Open # sixth cerv vertebra	1
S1017	Open # seventh cerv vert	1
S2112	Open # scapula, coracoid	1
S2111	Open # scapula, acromion	1
S211	Open # scapula	1
S3213	Open # patella, vertical	1
S3210	Open # patella, transverse	1
S3214	Open # patella, stellate	1
S3211	Open # patella, proximal pole	1
S3212	Open # patella, distal pole	1
S1051	Open # lumbar vert, wedge	1
S1050	Open # lumbar vert, burst	1
S126	Open # larynx and trachea	1
S1014	Open # fourth cerv vertebra	1
S1015	Open # fifth cerv vertebra	1
S2011	Open # clavicle, medial end	1
S2013	Open # clavicle, lateral end	1
S201	Open # clavicle	1
S101H	Open # cerv vert, wedge	1
S101G	Open # cerv vert, burst	1
S101z	Open # cerv spine NOS	1
S101F	Open # axis, tricolumnar	1
S101B	Open # axis, spondylolysis	1
S101C	Open # axis, spinous procss	1
S101E	Open # axis, posterior arch	1
S101A	Open # axis, odontoid prcss	1
S1019	Open # atlas, comminuted	1
S4113	Op tr ds shd jt,inf(infr-glen)	1
S4111	Op tr dis shld jt,ant(sub-cor)	1
S49Bz	Op sublx thrc+lmbr vertbra NOS	1
S49B2	Op spn sublx+thrc crd lsn,unsp	1
S49B6	Op spn sublx+post thrc crd lsn	1
S49BB	Op spn sublx+post lmbr crd lsn	1
S49B7	Op spn sublx+lmbr crd lsn,unsp	1
S49B3	Op spn sublx+comp thrc crd lsn	1

Read code	Description	Number of studies
S49B8	Op spn sublx+comp lmbr crd lsn	1
S499A	Op spn sublx+comp cerv crd lsn	1
S499C	Op spn sublx+cntrl crv crd lsn	1
S4999	Op spn sublx+cerv crd lsn,unsp	1
S49B5	Op spn sublx+cent thrc crd lsn	1
S49BA	Op spn sublx+cent lmbr crd lsn	1
S49BC	Op spn sublx+cauda equina lsn	1
S49B4	Op spn sublx+ant thrc crd lsn	1
S499D	Op spn sublux+post crv crd lsn	1
S49B9	Op spn sublux+ant lmbr crd lsn	1
S499B	Op spn sublux+ant cerv crd lsn	1
S4937	Op spn dsl+lmbr crd lsn unsp	1
S1134	Op spn #+pst thor crd lsn,T1-6	1
S1133	Op spn #+cnt thor crd lsn,T1-6	1
S1132	Op spn #+ant thor crd lsn,T1-6	1
S115z	Op spn # incmp lmb crd lsn NOS	1
S1130	Op sp #+unsp thor crd lsn,T1-6	1
S1136	Op sp #+unsp thor cd lsn,T7-12	1
S113A	Op sp #+pst thor crd lsn,T7-12	1
S1139	Op sp #+cnt thor crd lsn,T7-12	1
S1131	Op sp #+cmpl thor crd lsn,T1-6	1
S1137	Op sp #+cmp thor crd lsn,T7-12	1
S1138	Op sp #+ant thor crd lsn,T7-12	1
S1501	Op multi fractur of thor spine	1
SR161	Op fract/th wth lw bck+plv+lmb	1
SR101	Op fract invol head with neck	1
S113B	Op # T7-12incomp cord lsn NOS	1
S1135	Op # T1-6 incomp cord lsn NOS	1
S1116	Op # C5-7 unspec cord lesion	1
S111A	Op # C5-7 posterior cord lesn	1
S111B	Op # C5-7 incomp cord les NOS	1
S1117	Op # C5-7 compl cord lesion	1
S1119	Op # C5-7 central cord les	1
S1118	Op # C5-7 anterior cord les	1
S1110	Op # C1-C4 unspec cord les	1
S1114	Op # C1-4 post cord lesion	1
S1115	Op # C1-4 cord les. NOS	1
S1111	Op # C1-4 compl cord lesion	1
S1113	Op # C1-4 cent cord lesion	1
S1112	Op # C1-4 ant cord lesion	1
N1120	One lev th spondyl-no myelop	1
N11B0	One lev th spondyl + radiculop	1
N1130	One lev th spondyl + myelop	1
N1140	One lev lumbsac spond-no myelo	1
N1150	One lev lumbsac spond + myelop	1
N1100	One lev Cx spondyl-no myelop	1
N1190	One lev Cx spondyl + radiculop	1
N1110	One lev Cx spondyl + myelop	1
N0542	Oligoarticular osteoarthritis, unspecified, of upper arm	1
N0545	Oligoarticular osteoarthritis, unspecified, of the pelvic region and thigh	1
N0546	Oligoarticular osteoarthritis, unspecified, of lower leg	1

Read code	Description	Number of studies
N0544	Oligoarticular osteoarthritis, unspecified, of hand	1
N0543	Oligoarticular osteoarthritis, unspecified, of forearm	1
N0547	Oligoarticular osteoarthritis, unspecified, of ankle and foot	1
N054	Oligoarticular OA unspecified	1
N0541	Oligoartic OA, unspec-shoulder	1
N0549	Oligoartic OA, unspec-multiple	1
N0540	Oligoartic OA, unsp-unsp sites	1
N0548	Oligoartic OA unspec-oth site	1
N2133	Olecranon bursitis	1
N0720	Old torn meniscus of knee	1
N070B	Old tear post horn med menis	1
N070A	Old tear of medial meniscus	1
N071C	Old tear of lateral meniscus	1
N07y9	Old post/lat caps complex tear	1
N07y3	Old post.cruciate lig.disrupt.	1
N07yF	Old part tear post cruciat lig	1
N07yA	Old part tear med collat lig	1
N07y7	Old part tear lat collat lig	1
N07yD	Old part tear ant cruciate lig	1
N07y1	Old med.collat.lig.disruption	1
N07yC	Old med capsular complex tear	1
N07y0	Old lat.collat.lig.disruption	1
N07yB	Old compl tear med collat lig	1
N07y8	Old compl tear lat collat lig	1
N07yG	Old comp tear post cruciat lig	1
N07yE	Old comp tear ant cruciate lig	1
N07y4	Old capsular knee lig.disrupt.	1
N0701	Old bucket handle tear-medial	1
N0711	Old bucket handle tear-lat men	1
N07y2	Old ant.cruciate lig.disrupt.	1
N067	Ochronotic arthropathy	1
N054z	OA,1 site +,unspecified NOS	1
N05zP	OA NOS-subtalar joint	1
N05zA	OA NOS-sternoclavicular joint	1
N05zB	OA NOS-acromioclavicular join	1
N05zG	OA NOS-PIP joint of finger	1
N05zH	OA NOS-DIP joint of finger	1
N05zS	OA NOS-1st MTP joint	1
N3381	Nonunion of fracture	1
N226	Nontraumatic tendon subluxatn	1
N23y2	Nontraumatic muscle rupture	1
N2260	Nontraum.unspec.tendon rupture	1
N392	Nonallopathic lesion-thoracic	1
N393	Nonallopathic lesion-lumbar	1
N391	Nonallopathic lesion-cervical	1
N082Z	Non-trau subl acromiocl joint	1
N044	Nodular fibrositis-chr. rheum.	1
N2372	Nodular fasciitis	1
16A1	No stiff neck	1
16C1	No backache	1
1229	No FH: Osteoporosis	1
N2471	Night cramps	1

Read code	Description	Number of studies
N11y2	Neuropathic spondylopathy	1
N2423	Neuropathic pain	1
N374B	Neuromuscular scoliosis	1
N3749	Neuromuscular lordosis	1
N3745	Neuromuscular kyphosis	1
N14A	Neurogenic claudication	1
N2421	Neuritis unspecified	1
N2420	Neuralgia unspecified	1
N242z	Neuralg./neurit./radiculit.NOS	1
N242	Neuralg./neurit./radicul.unsp.	1
SJ	Nerve/spinal cord injuries	1
EMISNQNE5	Nerve root pain present	1
SJz	Nerve and spinal cord injury NOS	1
S5700	Neck sprain unspecified	1
S570z	Neck sprain NOS	1
S570	Neck sprain	1
N12D	Narrowing disc space	1
N2332	Myositis in sarcoidosis	1
N2321	Myofibrosis	1
N241-97	Myalgia/myositis - shoulder	1
EGTON307	Myalgia	1
EMISNQMU15	Musculoskeletal symptom	1
EMISNQMU5	Musculoskeletal pain severe	1
EMISNQMU2	Musculoskeletal pain present	1
EMISNQMU4	Musculoskeletal pain moderate	1
N096-2	Musculoskeletal pain - joints	1
MAWBYMU1	Musculoskeletal Symptoms	1
N3	Musculosk.inflam/deform.+other	1
N232z	Muscle wasting/atrophy NEC NOS	1
N232	Muscle wasting and disuse atrophy NEC	1
N2322	Muscle wasting NEC	1
UNMAPM4AB	Muscle strain	1
N23yD	Muscle strain	1
N2410-2	Muscle pain	1
N231	Muscle ossification	1
S5yz1	Muscle injury / strain	1
N238	Muscle contracture	1
N231z	Muscle calcif./ossificat.NOS	1
N230D	Muscle abscess-shoulder	1
N230B	Muscle abscess-neck	1
N230C	Muscle abscess-back	1
N230A	Muscle abscess	1
ASDFGMU2	Muscle Symptoms	1
EGTON110	Muscle Injury	1
M4A8	Muscle Injury	1
N2310	Musc.calcif./ossif.unspecified	1
N0719	Multiple tears-lat meniscus	1
N0708	Multiple tears of medial meniscus	1
S101x	Multiple open # cerv vert	1
SR1z	Multiple fractures unspecified	1
S100x	Multiple clsd # cerv vert	1
N099A	Multiple clicking joints	1
S3y	Multiple #legs/arms/ribs/stern	1

Read code	Description	Number of studies
S10A2	Multip fracture/cervical spin	1
N11C2	Multi lev lumbsac spond+radicu	1
S150	Multi fractures/thoracic spine	1
S3y1	Mult.open #legs/arms/ribs	1
S3y0	Mult.closed #legs/arms/ribs	1
N1122	Mult lev th spondyl-no myelop	1
N11B2	Mult lev th spondyl+radiculop	1
N1132	Mult lev th spondyl + myelop	1
N1142	Mult lev lumbsac spond-no myel	1
N1152	Mult lev lumbsac spond + myelo	1
N1102	Mult lev Cx spondyl-no myelop	1
N1192	Mult lev Cx spondyl+radiculop	1
N1112	Mult lev Cx spondyl + myelop	1
S10B6	Mult fractur/lumbar spine+pelv	1
S292	Mult fract/clav,scapula+humrus	1
1D17	Morning stiffness - joint	1
N3323	Monostotic fibrous dysplasia	1
N0433	Monarticular juvenile R.A.	1
EMISNQMI188	Mixed connective tissue disease	1
OX6954MC	Mixed Connective Tissue Disease /ox	1
N2224	Miners' knee	1
N2172	Metatarsalgia NOS	1
N063.11	Menopausal arthritis	1
N063	Menopausal arthritis	1
N072	Meniscus derangement NEC	1
N070	Medial meniscus derangement	1
N070z	Medial meniscus derange.NOS	1
N0703	Medial menisc.post.horn derang	1
N0700	Medial menisc.derang.unspecif	1
N0702	Medial menisc.ant.horn derang.	1
N2131	Medial epicondylitis - elbow	1
16CA	Mechanical low back pain	1
N3380	Malunion of fracture	1
N338	Malunion and nonunion of fracture	1
N11C	Lumbosacral spondylosis with radiculopathy	1
N115	Lumbosacral spond.+ myelopathy	1
SJ35	Lumbosacral plexus injury	1
N1441	Lumbosacral neuritis unspecif.	1
N1463	Lumbosacral instability	1
N1460	Lumbosacral ankylosis	1
N114-2	Lumbar spondylosis	1
N148C	Lumbar spine instability	1
N1486	Lumbar spine ankylosis	1
N1402	Lumbar spinal stenosis	1
N12A3	Lumbar postlaminectomy syndr.	1
SJ321	Lumbar nerve root injury - L2	1
N12zC	Lumbar discitis	1
N122	Lumbar disc displacement	1
N1293	Lumbar disc disord.+myelopathy	1
N127	Lumbar disc degeneration	1
S572	Lumbar back sprain	1
N1420	Lumbago with sciatica	1
N142-4	Lumbago	1

Read code	Description	Number of studies
N140A	Lu spin stenos due to oth dis	1
N12C2	Lu disc prolapse+radiculopathy	1
N12B2	Lu disc prolapse + myelopathy	1
N12C3	Lu disc prol+caud eq compress	1
N142-1	Low back pain	1
EGTON264	Low Back Pain	1
N374W	Lordosis unspecified	1
N3747	Lordosis in skeletal dysplasia	1
N3748	Lordosis in hip disease	1
N3742	Lordosis + other condition	1
N0818	Loose joint body-multip joints	1
N081z	Loose joint body (ex.knee)NOS	1
N081A	Loose body, oth joint-shoulder	1
N0811	Loose body joint-shoulder	1
N0819	Loose body in shoulder joint	1
N073	Loose body in knee	1
N081	Loose body in joint-excl.knee	1
N0817	Loose body in joint, joint OS	1
N0810	Loose body in joint	1
N07yH	Locking knee	1
N07y5	Locked knee	1
N0522	Localised, secondary osteoarthritis of the upper arm	1
N0525	Localised, secondary osteoarthritis of the pelvic region and thigh	1
N0526	Localised, secondary osteoarthritis of the lower leg	1
N0524	Localised, secondary osteoarthritis of the hand	1
N0523	Localised, secondary osteoarthritis of the forearm	1
N0527	Localised, secondary osteoarthritis of the ankle and foot	1
N051E	Localised, primary osteoarthritis of toe	1
N0512	Localised, primary osteoarthritis of the upper arm	1
N0515	Localised, primary osteoarthritis of the pelvic region and thigh	1
N0513	Localised, primary osteoarthritis of the forearm	1
N0517	Localised, primary osteoarthritis of the ankle and foot	1
N051F	Localised, primary osteoarthritis of elbow	1
N052z	Localised secondary OA NOS	1
N051z	Localised primary OA NOS	1
N0532	Localised osteoarthritis, unspecified, of the upper arm	1
N0537	Localised osteoarthritis, unspecified, of the ankle and foot	1
N053z	Localised OA unspecified NOS	1
N053	Localised OA unspecified	1
N052	Local.secondary osteoarthritis	1
N0520	Local.secondary OA-site unsp.	1
N0521	Local.secondary OA-shoulder	1
N0528	Local.secondary OA-other spec.	1
N051	Local.primary osteoarthritis	1
N0510	Local.primary OA-site unspec.	1
N0511	Local.primary OA-shoulder regn	1
N0518	Local.primary OA-other specif	1
N0516	Local.primary OA-lower leg	1
N0514	Local.primary OA-hand	1

Read code	Description	Number of studies
N0530	Local.OA unsp.-site unspecif.	1
N0531	Local.OA unsp.-shoulder region	1
N0538	Local.OA unsp.-other specified	1
N0534	Local.OA unsp.-hand	1
N0533	Local.OA unsp.-forearm	1
N051D	Local prim osteoarth wrist	1
N3308	Local osteoporosis - Lequesne	1
S5z	Ligament sprain NOS	1
N0001	Libman-Sacks disease	1
N245-6	Leg pain	1
N234	Laxity of ligament	1
SJ43	Latrl cutaneous branch T12 inj	1
N071	Lateral meniscus derangement	1
N0714	Lateral meniscus derangem.NOS	1
N0713	Lateral menisc.post.horn deran	1
N0710	Lateral menisc.derang.unspecif	1
N0712	Lateral menisc.ant.horn derang	1
N2132	Lateral epicondylitis of the elbow	1
MHTBALA9	Lateral Patella Release	1
SC08	Late effect-tendon injury	1
SC05	Late effect-mult./other #bones	1
SC06	Late effect-dislocation	1
SC01	Late effect-#spine-no cord les	1
SC011	Late effect # thoracic vert	1
SC012	Late effect # lumbar vertebra	1
SC010	Late effect # cervic vertebra	1
EGTON119	Laceration Nos	1
N3744	Kyphosis in skeletal dysplasia	1
N3713	Kyphosis due to oth treatment	1
N3741	Kyphosis + other condition	1
N373z	Kyphoscoliosis or scoliosis NOS	1
N373	Kyphoscoliosis and scoliosis	1
N3241	Kohler's dis.(prim.patell.ctr)	1
N0956	Knee stiff	1
S54y	Knee sprain NOS	1
S54	Knee sprain	1
N0106	Knee pyogenic arthritis	1
N0826	Knee pathological dislocation	1
1M10	Knee pain	1
N05z6	Knee osteoarthritis NOS	1
N05z6-1	Knee osteoarthritis NOS	1
N0946-1	Knee joint pain	1
N0946	Knee joint pain	1
N0846	Knee joint contracture	1
N0856	Knee joint ankylosis	1
N0966	Knee gives way	1
N216z	Knee enthesopathy NOS	1
N06z6-1	Knee arthritis NOS	1
N06z6	Knee arthritis NOS	1
ASDFGKN2	Knee Pain?	1
ASDFGKN6	Knee Pain Does Not Affect Sleep	1
ASDFGKN4	Knee Pain Affects Sleep?	1
ASDFGKN5	Knee Pain Affects Sleep	1

Read code	Description	Number of studies
ASDFGKN3	Knee Pain	1
MUNNUKN1	Knee Pain	1
MAWBYKN1	Knee Pain	1
N0836	Knee - recurrent dislocation	1
N13y1	Klippel's disease	1
N116	Kissing spine	1
N002	Keratoconjunctivitis sicca	1
N0600	Kaschin-Beck dis.-site unspec.	1
N0601	Kaschin-Beck dis.-shoulder	1
N0608	Kaschin-Beck dis.-other specif	1
N0609	Kaschin-Beck dis.-multipl.site	1
N060z	Kaschin-Beck dis.-NOS	1
N060	Kaschin - Beck disease	1
N320z	Juvenile spine osteochondr.NOS	1
N3200	Juvenile spine osteochond.unsp	1
N0455	Juvenile rheumatoid arthritis	1
N0430	Juvenile rheumatoid arthr.unsp	1
N043z	Juvenile rheumatoid arthr.NOS	1
N3263	Juvenile osteochondrosis NOS	1
N326z	Juvenile osteochondroses NOS	1
N3262	Juvenile osteochondritis NOS	1
N3261	Juvenile epiphysitis NOS	1
N0030	Juvenile dermatomyositis	1
N3260	Juvenile apophysitis NOS	1
N043	Juvenile R.A.- Still's disease	1
OX7120DA	Juvenile Arthritis /ox	1
N3243	Juv.osteoch.secondary.pat.ctre	1
N0451	Juv seronegative polyarthritis	1
N328	Juv osteochondrosis of spine	1
N0452	Juv arthritis in psoriasis	1
N0453	Juv arthritis in Crohn's dis	1
N0454	Juv arth in ulcerative colitis	1
N0900	Joint effusion-site unspecif.	1
N0901	Joint effusion-shoulder region	1
N0908	Joint effusion-other specif.	1
N09z1	Joint disord.NOS-shoulder	1
N08z	Joint derangement NOS	1
N08zz	Joint derangement NOS	1
N08z0	Joint derange.NOS-site unspec.	1
N08z1	Joint derange.NOS-shoulder	1
N08z7	Joint derange.NOS-other spec.	1
N08z8	Joint derange.NOS-multipl.site	1
N05z.11	Joint degeneration	1
N05z	Joint degeneration	1
N0840	Joint contracture-site unspec	1
N0841	Joint contracture-shoulder	1
N0848	Joint contracture-other specif	1
N0850	Joint ankylosis-site unspecif.	1
N0851	Joint ankylosis-shoulder	1
N0858	Joint ankylosis-other specif.	1
ASDFGJO2	Joint Symptoms	1
ASDFGJO3	Joint Pain	1
N3841	Isthmic spondylolisthesis	1

Read code	Description	Number of studies
S531	Ischiocapsular sprain	1
N23yB	Ischaemic infarction of muscle	1
N123-1	Intervertebral disc prol. NOS	1
N12z	Intervertebral disc lesion NOS	1
OX7259AP	Intervertebral Disc Prolapsed /ox	1
N3y05	Intervert disc sten neur canal	1
N23y0	Interstitial myositis	1
N07z	Internal knee derangement NOS	1
N07	Internal derangement of knee	1
N1350	Intermittent torticollis	1
N090W	Intermittent hydrarthrosis	1
N084E	Int rotat contracture-shoulder	1
N08y	Instability of joint	1
SJ7z	Injury to other nerve NOS	1
SKz	Injury NOS	1
OX9967C	Injury Knee /ox	1
SJ9	Injur/nerv+spinl crd/thorx lev	1
SJB	Inj/nerves/should+upp arm levl	1
S46C	Inj/multipl structures of knee	1
S5Q6	Inj tendon rotator cuff should	1
N2166	Infrapatellar bursitis	1
N10	Inflammatory spondylopathies	1
N04	Inflammatory polyarthropathy	1
N04*	Inflammatory arthropathy	1
N10y0	Inflamm.spondylop.in dis. EC	1
N04z	Inflamm.polyarthropathy NOS	1
S4103	Inferior dislocation shoulder	1
N2302	Infective myositis-shoulder	1
N2300	Infective myositis-neck	1
N2301	Infective myositis-back	1
N01z	Infective arthritis NOS	1
N01zz	Infective arthritis NOS	1
N302B	Infection of thoracic spine	1
N302G	Infection of scapula	1
N302R	Infection of patella	1
N302Z	Infection of multiple bones	1
N302C	Infection of lumbar spine	1
N302F	Infection of clavicle	1
N302A	Infection of cervical spine	1
N01z0	Infect.arthr.NOS-site unspecif	1
N01z1	Infect.arthr.NOS-shoulder reg	1
N01zy	Infect.arthr.NOS-other specifi	1
N01zx	Infect.arthr.NOS-multiple site	1
N12zG	Infect intervert disc - pyogen	1
N01z8	Infec arthritis NOS-shoulder	1
N01zK	Infec arthritis NOS-knee	1
N01z9	Infec arthr NOS-sternoclav jt	1
N01zA	Infec arth NOS-acromioclav jt	1
N23y5	Inappropriate firing of muscle	1
N2124	Impingement syndr of shoulder	1
N2331	Immobility syndrome	1
N2159	Iliotibial band syndrome	1
N1403	Idiopathic th spinal stenosis	1

Read code	Description	Number of studies
N3730	Idiopathic scoliosis	1
N3303	Idiopathic osteoporosis	1
N1407	Idiopathic lu spinal stenosis	1
N3731	Idiopathic kyphoscoliosis	1
N33z7	Idiopathic hypertrophy of bone	1
N1300	Idiopathic Cx spinal stenosis	1
N3316	Idiopath osteopor + path fract	1
N3348	Idiopath asep necrosis of bone	1
N1405	Iatrogenic th spinal stenosis	1
N1409	Iatrogenic lu spinal stenosis	1
N1302	Iatrogenic Cx spinal stenosis	1
C04*	Hypothyroidism	1
N2431	Hypertrophy of knee fat pad	1
N33z4	Hypertrophy of bone	1
N3382	Hypertrophic non-union of #	1
N312	Hypertroph.pulm.osteoarthrop.	1
N02y8	Hydroxyapatite deposition dis	1
N2225	Housemaids knee	1
N0707	Horiz cleavage tear-med menisc	1
N0718	Horiz cleavage tear-lat menisc	1
N094K-2	Hip pain	1
N05z5	Hip osteoarthritis NOS	1
N05z5-1	Hip osteoarthritis NOS	1
N0535	Hip osteoarthitis NOS	1
N0535-2	Hip osteoarthitis NOS	1
N06z5	Hip arthritis NOS	1
N0809	Hill-Sachs lesion	1
N245-5	Heel pain	1
N0507	Heberden's nodes with arthropathy	1
N245-4	Hand pain	1
N2450	Hand pain	1
N0944-1	Hand joint pain	1
N06z4-1	Hand arthritis NOS	1
N068	Haemophilic arthropathy	1
N091B	Haemarthrosis-sternoclav joint	1
N0910	Haemarthrosis-site unspecified	1
N0911	Haemarthrosis-shoulder	1
N0918	Haemarthrosis-other specified	1
N0919	Haemarthrosis-multiple joints	1
N091C	Haemarthrosis-acromioclav jt	1
N0916	Haemarthrosis of the knee	1
N091A	Haemarthrosis of shoulder	1
N091M	Haemarthrosis of knee	1
N091z	Haemarthrosis NOS	1
N091	Haemarthrosis	1
N083M	Habitual sublux shoulder	1
N083L	Habitual disloc shoulder	1
N083r	Habitual disloc - patella	1
14G8	H/O: vertebral fracture	1
14G1	H/O: rheumatoid arthritis	1
14G2	H/O: osteoarthritis	1
14GZ	H/O: musculo-skeletal dis. NOS	1
14G3	H/O: knee problem	1

Read code	Description	Number of studies
14J	H/O: injury	1
14G4	H/O: back problem	1
14T5	H/O: artificial joint	1
14V5	H/O: arthrodesis	1
14G	H/O: arthritis	1
14N30	H/O Spinal surgery	1
16Z2	Growing pains	1
S3z00	Greenstick fracture	1
N0230	Gouty arthritis-site unspecif.	1
N0231	Gouty arthritis-shoulder	1
N023y	Gouty arthritis-other specif.	1
N023x	Gouty arthritis-multiple sites	1
N023z	Gouty arthritis NOS	1
N023	Gouty arthritis	1
N2131-1	Golfer's elbow	1
N200	Gnt cell arter+polymyalg rheum	1
EGTON444	Gluteal Muscle Injury	1
N0873	Glenoid labrum tear	1
N0872	Glenoid labrum detachment	1
N365	Genu recurvatum - acquired	1
N050z	Generalised osteoarthritis NOS	1
N050z00	Generalised osteoarthritis NOS	1
N050	Generalised osteoarthritis - OA	1
N050.0	Generalised osteoarthritis - OA	1
N065A	Generalised arthritis	1
N065A00	Generalised arthritis	1
N05000	Generalised OA-site unspecif.	1
N0500	Generalised OA-site unspecif.	1
N050200	Generalised OA-multiple sites	1
N0502	Generalised OA-multiple sites	1
N0501	Generalised OA-hand	1
N224z	Ganglion/synovial cyst NOS	1
N224	Ganglion/synov.cyst - knee	1
N224-92	Ganglion/synov.cyst - knee	1
N2243	Ganglion unspecified	1
N2245	Ganglion of wrist	1
N2242	Ganglion of tendon sheath	1
N2246	Ganglion of knee	1
N2241	Ganglion of joint	1
N224C	Ganglion of foot	1
EMISR4QFU2	Fusion Of Lumbar Spine	1
EMISR4QFU1	Fusion Of Cervical Spine	1
N2115	Full thickn rotator cuff tear	1
N210-2	Frozen shoulder	1
N331N	Fragility fracture	1
N331M	Fragility # unsp osteoporosis	1
S10B	Fracture/lumbar spine+pelvis	1
S10A1	Fracture/2nd cervical vertebra	1
S10A0	Fracture/1st cervical vertebra	1
S15	Fracture of thoracic vertebra	1
S1z	Fracture of neck and trunk NOS	1
S1	Fracture of neck and trunk	1
S10A	Fracture of neck	1

Read code	Description	Number of studies
S10B0	Fracture of lumbar vertebra	1
N338z	Fracture malunion or nonunion NOS	1
SR1	Fracture involv multi body reg	1
SR10	Fracture involv head with neck	1
S4	Fracture dislocation/subluxat	1
OX8056	Fracture Spine /ox	1
S3z	Fracture NOS	1
OXL8056LV	Fracture Lumbar Vertebra /ox	1
SR16	Fract/thorx wth lw bck+plv+lmb	1
N3317	Fract of bone in neoplast dis	1
N2451	Foot pain	1
N245-3	Foot pain	1
1M11	Foot pain	1
N220B	Flexor tenosynovitis of finger	1
N3660	Flexion deformity of knee	1
N369	Flexion deformity	1
N084A	Flexion contracture-shoulder	1
N084a	Flexion contracture-knee	1
N374E	Flatback syndrome	1
N040T	Flare of rheumatoid arthritis	1
N08yA	Flail joint	1
N09C	Fistula of joint	1
N2450-2	Finger pain	1
N05z4-1	Finger osteoarthritis NOS	1
N2163	Fibular collat.lig.bursitis	1
N3324	Fibrous cortical defect	1
N2405	Fibrositis of neck	1
N00y1	Fibrosclerosis systemic	1
N087	Fibrocartilage lesion of joint	1
MHTBAFH1	Fh: Osteoporosis	1
N041	Felty's syndrome	1
N1y1	Fatigue fracture of vertebra	1
N244	Fasciitis unspecified	1
N024	Familial chondrocalcinosis	1
N14y	Facet joint syndrome	1
12I1	FH: Rheumatoid arthritis	1
1268	FH: Osteoporosis	1
12I2	FH: Osteoarthritis	1
12IZ	FH: Musculo-skeletal dis. NOS	1
12I	FH: Arthritis	1
N220D	Extensor tenosynovitis of wrist	1
N220F	Extensor tenosynovitis of thumb	1
N220E	Extensor tenosynovitis of finger	1
N084B	Extension contracture-shoulder	1
N084F	Ext rotat contracture-shoulder	1
N21z7	Exostosis	1
16C8	Exacerbation of backache	1
N0506	Erosive osteoarthrosis	1
N32z1	Epiphysitis NOS	1
N33z1	Epiphyseal arrest	1
N00y0	Eosinophilic fasciitis	1
N216	Enthesopathy of knee	1
N21z	Enthesopathy NOS	1

Read code	Description	Number of studies
N11y1	Enterobacterial spondylitis	1
1M00-1	Elbow pain	1
1M00	Elbow pain	1
N05z2	Elbow osteoarthritis NOS	1
N06z2	Elbow arthritis NOS	1
N090B	Effusion of sternoclav joint	1
N090A	Effusion of shoulder	1
N0909	Effusion of multiple joints	1
N090M	Effusion of knee	1
N090z	Effusion of joint NOS	1
N090	Effusion of joint	1
N090C	Effusion of acromioclav joint	1
N0906	Effusion - knee joint	1
N0906-99	Effusion - knee joint	1
N3840	Dysplastic spondylolisthesis	1
N2361	Dupuyt dis-palm + nod no cont	1
N3305	Drug-induced osteoporosis	1
N0002	Drug-ind systemic lupus eryth	1
N3315	Drug-ind osteopor + path fract	1
N235	Double-jointed (hypermobility)	1
N1123	Dorsal spondylo w/o myelopath	1
N3370	Disuse atrophy of bone	1
N3304	Dissuse osteoporosis	1
N0000	Disseminated lupus erythemat.	1
N09AX	Disorder of patella unspecified	1
N33zG	Disorder of cartilage, unspec	1
N33zF	Disorder of bone unspecified	1
S46	Dislocation or subluxation of knee	1
S4z	Dislocation or subluxation NOS	1
S41z	Dislocation of shoulder NOS	1
S46z	Dislocation of knee NOS	1
S41	Dislocated shoulder	1
S463-99	Dislocated patella	1
SR20	Disloc,sprns+strns inv hd+neck	1
SR2	Dislc,sprns+strns/mult bdy reg	1
N071B	Discoid lateral meniscus	1
N123	Disc unsp.displ.-no myelopathy	1
N12B	Disc prolapse with myelopathy	1
N12C	Disc prolapse + radiculopathy	1
N12zz	Disc disorders NOS	1
N129z	Disc disorder+myelopathy NOS	1
N00	Diffuse connective tissue dis.	1
N097z	Difficulty in walking NOS	1
N097	Difficulty in walking	1
N23yA	Diastasis of muscle	1
N33z3	Diaphysitis	1
N0300	Diabetic cheiroarthropathy	1
N0301	Diabetic Charcot arthropathy	1
N003X	Dermatopolymyositis, unspec	1
N0031	Dermatopolymyosit,neoplast dis	1
N003	Dermatomyositis	1
SC0z	Delayed union of fracture	1
N3386	Delayed union of fracture	1

Read code	Description	Number of studies
S9030	Degloving injury,shoulder area	1
SA130	Degloving injury knee	1
N3842	Degenerative spondylolisthesis	1
N128	Degenerative disc disease NOS	1
N1404	Degenerativ th spinal stenosis	1
N1408	Degenerativ lu spinal stenosis	1
N1301	Degenerativ Cx spinal stenosis	1
N114	Degeneration of lumbar spine	1
N114-1	Degeneration of lumbar spine	1
N0721	Degen lesion artic cart knee	1
N36y4	Deformity of scapula	1
N36yD	Deformity of patella	1
N36y3	Deformity of clavicle	1
N36y2	Deformity of bone	1
N224D	Cyst of tendon sheath	1
N0722	Cyst of semilunar cartilage	1
N0709	Cyst of medial meniscus	1
N071A	Cyst of lateral meniscus	1
N2244	Cyst of bursa	1
N332z	Cyst of bone NOS	1
N332	Cyst of bone	1
N11A	Cx spondyl + vasc compression	1
N1303	Cx spin stenos due to oth dis	1
N12C0	Cx disc prolapse+radiculopathy	1
N12B0	Cx disc prolapse + myelopathy	1
N3740	Curvature of spine unspecified	1
N37zz	Curvature of spine NOS	1
N37z	Curvature of spine NOS	1
N37	Curvature of spine	1
N02zK	Crystal arthropathy NOS-knee	1
N02zz	Crystal arthropathy NOS	1
N02z	Crystal arthropathy NOS	1
N02z0	Crystal arthr.NOS-site unspec.	1
N02z1	Crystal arthr.NOS-shoulder	1
N02zy	Crystal arthr.NOS-other spec.	1
N02zx	Crystal arthr.NOS-multipl.site	1
N02z9	Cryst arthr NOS-sternoclav jt	1
N02zA	Cryst arthr NOS-acromioclav jt	1
N02z8	Crys arthr NOS-shoulder	1
SF20z	Crushinjuryshlder+uparmNOS	1
SFz	Crushing injury NOS	1
SF	Crushing injury	1
SF40	Crush injury multiple sites NEC	1
SF021	Crush injury larynx	1
SF311	Crush injury knee	1
SF110	Crush injury back	1
SF4z	Crush injury	1
N13y2	Crick in neck	1
N2472	Cramp	1
N051A	Coxarthr from dysplasia, bilat	1
N3216	Coxa plana	1
182B0	Costal margin chest pain	1
N2123	Coracoid impingement	1

Read code	Description	Number of studies
S502	Coracohumeral sprain	1
SE30z	Contusionshlder+uer arm NOS	1
SE44	Contusionlwr limbmlti sites	1
SE00	Contusion, forehead	1
SEz	Contusion with skin intact NOS	1
SE30	Contusion shoulder or upper arm	1
SE300	Contusion shoulder area	1
SE301	Contusion scapular area	1
SE231	Contusion of lower back	1
SE232	Contusion of lower back	1
SE4y	Contusion multiple sites NEC	1
SE41z	Contusion knee and lower leg NOS	1
SE411	Contusion knee	1
SE0z	Contusion face scalp+neck NOS	1
SE304	Contusion clavicular area	1
SE23z	Contusion back NOS	1
SE	Contusion (bruise) with intact skin	1
N22y0	Contracture of tendon sheath	1
N23yC	Contracture of muscle	1
N0849	Contracture of multiple joints	1
N084z	Contracture of joint NOS	1
N084	Contracture of joint	1
N	Connective tissue diseases	1
N3y04	Connect tiss sten neural canal	1
PE1	Congenital sternomastoid torticollis	1
N3y07	Con tis/disc sten intervrt for	1
N33C	Complex regionl pain syndrom I	1
S5C	Complete tear, knee ligament	1
S58z	Complete tear shoulder joint NOS	1
S58	Complete tear shoulder joint	1
N33z8	Complete epiphyseal arrest	1
N33z6	Compensatory hypertrophy-bone	1
SK0y	Compartmentsyndrome	1
S5A0D	Comp tr shrt intr lig non-sp	1
N331D	Collapsed vertebra NOS	1
N331	Collapse of vertebra NOS	1
N331F	Collapse of thoracic vertebra	1
N3310	Collapse of thoracic vertebra	1
N3311	Collapse of lumbar vertebra	1
N331G	Collapse of lumbar vertebra	1
N331E	Collapse of cervical vertebra	1
N331L	Collap vert due osteopor NOS	1
N331J	Collap lumb vert due to osteo	1
N331H	Collap cerv vert due to osteop	1
N00z	Collagen disease NOS	1
N331K	Coll thorac vert due osteopor	1
N147z	Coccyx disorder NOS	1
N1472	Coccygodynia	1
S5E1	Cmplt tr,thyroid region lgmt	1
S5E30	Cmplt tr,sternoclavicular lgmt	1
S5Ez	Cmplt tr,other lgmt NOS	1
S5Cy	Cmplt tr,other knee lgmt	1
S5C1	Cmplt tr,knee,mdl collat lgmt	1

Read code	Description	Number of studies
S5C0	Cmplt tr,knee,lat collat lgmt	1
S5Cz	Cmplt tr,knee lgmt NOS	1
S581	Cmplt tr,coraco-clav lgmt	1
S580	Cmplt tr,acromio-clav lgmt	1
S5A0A	Cmplt tr triang fibrocartilage	1
S5C3	Cmpl tr,knee,ant cruciate lgmt	1
S5y57	Cmpl tear,lumbosacral lgmt	1
S5y56	Cmpl tear,iliolumbar lgmt	1
S5C2	Cmp tr,knee,post cruciate lgmt	1
S2920	Clsd mult fract clav scap hum	1
S1000	Clsd # unsp cerv vertebra	1
S1024	Clsd # thorc vert-trnsvrs prcs	1
S1026	Clsd # thorc vert - tricolumnr	1
S1025	Clsd # thorc vert - post prcs	1
S1020	Clsd # thoracic vert, burst	1
S1021	Clsd # thoracic vert wedge	1
S102z	Clsd # thorac vert NOS	1
S1023	Clsd # thor vert-spinous prcss	1
S1003	Clsd # third cerv vertebra	1
S2102	Clsd # scapula, coracoid	1
S1041	Clsd # lumbar vert wedge	1
S1040	Clsd # lumbar vert burst	1
S1046	Clsd # lumb vert - tricolumnar	1
S1004	Clsd # fourth cerv vertebra	1
S1005	Clsd # fifth cerv vertebra	1
S2001	Clsd # clavicle medial end	1
S2003	Clsd # clavical lateral end	1
S100H	Clsd # cerv vert, wedge	1
S100G	Clsd # cerv vert, burst	1
S100z	Clsd # cerv spine NOS	1
S100F	Clsd # axis, tricolumnar	1
S100D	Clsd # axis, transvrse process	1
S100B	Clsd # axis, spondylolysis	1
S100C	Clsd # axis, spinous process	1
S100E	Clsd # axis, posterior arch	1
S100A	Clsd # axis odontoid process	1
S1009	Clsd # atlas, comminuted	1
S1106	Clsd # C5-C7 unspec cord les	1
S110A	Clsd # C5-C7 post cord lesion	1
S110B	Clsd # C5-C7 incomp cord les	1
S1107	Clsd # C5-C7 complete cord les	1
S1109	Clsd # C5-C7 cent cord lesion	1
S1108	Clsd # C5-C7 ant cord lesion	1
S1100	Clsd # C1-C4 unspec cord les	1
S1104	Clsd # C1-C4 post cord lesion	1
S1105	Clsd # C1-C4 incomp cord les	1
S1101	Clsd # C1-C4 complete cord les	1
S1103	Clsd # C1-C4 cent cord lesion	1
S1102	Clsd # C1-C4 ant cord lesion	1
S4120	Cls trmtc subluxatn shldr jnt	1
S4696	Cls trmtc sublux,head fibula	1
S467	Cls trmtc sublux pat-fem jt	1
S4690	Cls trmtc sublux knee jt,unsp	1

Read code	Description	Number of studies
S4692	Cls trmtc sublux knee jt,post	1
S4694	Cls trmtc sublux knee jt,ltrl	1
S4691	Cls trmtc sublux knee jt,ant	1
S469	Cls trmtc sublux knee jt	1
S4102	Cls trmtc dslctn shldr jtpost	1
S410z	Cls trmtc dislctn shoulder NOS	1
S49E4	Cls trm sublux,st-clav jt,post	1
S49E3	Cls trm sublux,st-clav jt,ant	1
S49E2	Cls trm sublux st-clav jt	1
S4671	Cls trm sublux pat-fem jt,med	1
S4670	Cls trm sublux pat-fem jt,ltrl	1
S49E5	Cls trm sublux laryngl cart	1
S4695	Cls trm sublux knee jt,rotatry	1
S4693	Cls trm sublux knee jt,medial	1
S4121	Cls trm sublux acromio-clav jt	1
S4656	Cls trm dslctn, head fibula	1
S4631	Cls trm dslctn pat-fem jt,med	1
S4630	Cls trm dslctn pat-fem jt,lat	1
S4650	Cls trm dslctn knee, unspec	1
S4654	Cls trm dslctn knee jt,lateral	1
S4652	Cls trm dslctn knee jt, post	1
S4653	Cls trm dslctn knee jt, medial	1
S4651	Cls trm dslctn knee jt, ant	1
S465z	Cls trm dslctn knee NOS	1
S4655	Cls trm dslct knee jt,rotatory	1
S4963	Cls trm dslc,stern-clav jt,ant	1
S4962	Cls trm dslc sterno-clav jt	1
S4965	Cls trm dslc laryngl cartilage	1
S4964	Cls trm dsl,stern-clav jt,post	1
S412	Cls traumtc subluxatn shoulder	1
S410	Cls traumtc disloctn shoulder	1
S4105	Cls traumatic disloctn scapula	1
S49A	Cls sublux thrcic+lumbar spine	1
S49A1	Cls sublux thrcic spine	1
S49Az	Cls sublux thrc+lmbr spine NOS	1
S498x	Cls sublux mlti cerv vertebrae	1
S4980	Cls sublux cervical spine,unsp	1
S498	Cls sublux cervical spine	1
S498z	Cls sublux cerv vertebra NOS	1
S4981	Cls sublux atlanto-occiptl jt	1
S4982	Cls sublux atlanto-axial jt	1
S492C	Cls spnl dslc+cauda equina lsn	1
S4924	Cls spnl dslc+ant thrc crd lsn	1
S4929	Cls spnl dslc+ant lmbr crd lsn	1
S490B	Cls spnl dslc+ant cerv crd lsn	1
S492B	Cls spnl dsl+post lmbr crd lsn	1
S4923	Cls spnl dsl+comp thrc crd lsn	1
S4928	Cls spnl dsl+comp lmbr crd lsn	1
S4922	Cls spn dslc+thrc crd lsn,unsp	1
S4926	Cls spn dslc+post thrc crd lsn	1
S490D	Cls spn dslc+post cerv crd lsn	1
S4925	Cls spn dslc+cent thrc crd lsn	1
S492A	Cls spn dslc+cent lmbr crd lsn	1

Read code	Description	Number of studies
S1140	Cls spn # + unsp lumb crd lesn	1
S1144	Cls spn # + post lumb crd lesn	1
S1141	Cls spn # + comp lumb crd lesn	1
S1143	Cls spn # + cent lumb crd lesn	1
S1145	Cls spn # + cauda equina lesn	1
S1142	Cls spn # + ant lumbr crd lesn	1
SR100	Cls fract invol head with neck	1
S492z	Cls dslc thrcic+lmbr spine NOS	1
S492	Cls dslc thoracic+lumbar spine	1
S4921	Cls dslc thoracic vertebra	1
S4920	Cls dslc lumbar spine	1
S4901	Cls dslc atlanto-occipital jnt	1
S4902	Cls dslc atlanto-axial joint	1
S4F6	Cls #-sublux,patello-fem jt	1
S4J23	Cls #-sublux st-clav jt,post	1
S4J22	Cls #-sublux st-clav jt,ant	1
S4F4	Cls #-dslc,patello-fem jt	1
S4J03	Cls #-dslc st-clav jt,post	1
S4J02	Cls #-dslc st-clav jt,ant	1
S1022	Cls # thorc vert-spondylolysis	1
S1044	Cls # lumbr vert-trnsvrse prcs	1
S1042	Cls # lumbr vert-spondylolysis	1
S1043	Cls # lumbr vert-spinous prcss	1
S1045	Cls # lumb vert-posterior arch	1
S100L	Cls # cerv vert, trnsvrse prcs	1
S100N	Cls # cerv vert, tricolumnar	1
S100J	Cls # cerv vert, spondylolysis	1
S100K	Cls # cerv vert, spinous prcss	1
S100M	Cls # cerv vert, post arch	1
S1008	Cls # atlas-isol arch/art prcs	1
S412z	Closed traumatic subluxation shoulder NOS	1
S496z	Closed traumatic disloctn NOS	1
S4100	Closed traumatic dislocation shoulder joint, unspecified	1
S463	Closed traumatic dislocation of patello-femoral joint	1
S112z	Closed thoracic#+cord lesn.NOS	1
S112	Closed thoracic #+cord lesion	1
S49Cz	Closed subluxation spine NOS	1
S49A0	Closed subluxation lumbar spine	1
S4988	Closed subluxation C7/T1	1
S4987	Closed subluxation C6/C7	1
S4986	Closed subluxation C5/C6	1
S4985	Closed subluxation C4/C5	1
S4984	Closed subluxation C3/C4	1
S4983	Closed subluxation C2/C3	1
S49C0	Closed sublux spine, unsp	1
S49C	Closed sublux other vertebra	1
S026	Closed orbital blow-out fracture	1
S114	Closed lumbar # + cord lesion	1
S102	Closed fracture thoracic vertebra	1
S2104	Closed fracture scapula, blade	1
S2105	Closed fracture scapula spine	1
S2106	Closed fracture scapula neck	1

Read code	Description	Number of studies
S2103	Closed fracture scapula glenoid	1
S2101	Closed fracture scapula acromion	1
S3204	Closed fracture patella, comminuted (stellate)	1
S320	Closed fracture of the patella	1
S1250	Closed fracture larynx	1
S2002	Closed fracture clavicle shaft	1
S1002	Closed fracture axis	1
S1001	Closed fracture atlas	1
S4940	Closed dislocation spine unsp.	1
S494z	Closed dislocation spine NOS	1
S490	Closed dislocation cervical spine	1
S4908	Closed dislocation C7/T1	1
S4907	Closed dislocation C6/C7	1
S4906	Closed dislocation C5/C6	1
S4905	Closed dislocation C4/C5	1
S4904	Closed dislocation C3/C4	1
S4903	Closed dislocation C2/C3	1
S494	Closed disloc.other vertebra	1
S490x	Closed disloc.mult.cerv.vert.	1
S490z	Closed disloc.cervic.vert.NOS	1
S4900	Closed disloc.cerv.spine unsp.	1
S4960	Closed disloc sternoclavic. jt	1
S4104	Closed disloc acromioclavic.jt	1
S110z	Closed cervical#+cord lesn.NOS	1
S110	Closed cervical #+cord lesion	1
S1253	Closed #trachea	1
S1252	Closed #thyroid cartilage	1
S11x	Closed #spine+cord lesn.unsp.	1
S2100	Closed #scapula-unspecified	1
S210z	Closed #scapula NOS	1
S125z	Closed #larynx/trachea NOS	1
S1251	Closed #hyoid bone	1
S2000	Closed #clavicle unspecified	1
S200z	Closed #clavicle NOS	1
S3z0	Closed #bones unspecified	1
S4F2	Closed #-sublux, knee joint	1
S4A20	Closed #-sublux shoulder joint	1
S4A2	Closed #-sublux shoulder	1
S4A21	Closed #-sublux acrom-clav jt	1
S4A00	Closed #-dslc shoulder joint	1
S4A0	Closed #-dslc shoulder	1
S4F0	Closed #-dslc knee joint	1
S4A01	Closed #-dslc acrom-clav joint	1
S10x	Closed # spine unspecif	1
S210	Closed # scapula	1
S3201	Closed # patella,proximal pole	1
S3203	Closed # patella, vertical	1
S3200	Closed # patella transverse	1
S3202	Closed # patella distal pole	1
S200	Closed # clavicle	1
S100	Closed # cervical spine	1
N063000	Climacteric arthr.-site unsp.	1
N0630	Climacteric arthr.-site unsp.	1

Read code	Description	Number of studies
N0631	Climacteric arthr.-shoulder	1
N0638	Climacteric arthr.-other spec.	1
N063800	Climacteric arthr.-other spec.	1
N0639	Climacteric arthr.-multip.site	1
N063900	Climacteric arthr.-multip.site	1
N063z	Climacteric arthr.-NOS	1
N063z00	Climacteric arthr.-NOS	1
N0991	Clicking sternoclavic joint	1
N0990	Clicking shoulder	1
N099C	Clicking knee	1
N099	Clicking joint	1
N0992	Clicking acromioclavicular joint	1
N33A1	Clavicle pain	1
S490A	Cl spnl dslc+comp cerv crd lsn	1
S4909	Cl spnl dslc+cerv crd lsn,unsp	1
S49A2	Cl spn sublx+thrc crd lsn,unsp	1
S49A6	Cl spn sublx+post thrc crd lsn	1
S49AB	Cl spn sublx+post lmbr crd lsn	1
S49A7	Cl spn sublx+lmbr crd lsn,unsp	1
S49A3	Cl spn sublx+comp thrc crd lsn	1
S49A8	Cl spn sublx+comp lmbr crd lsn	1
S498A	Cl spn sublx+comp cerv crd lsn	1
S498C	Cl spn sublx+cntrl crv crd lsn	1
S4989	Cl spn sublx+cerv crd lsn,unsp	1
S49A5	Cl spn sublx+cent thrc crd lsn	1
S49AA	Cl spn sublx+cent lmbr crd lsn	1
S49AC	Cl spn sublx+cauda equina lsn	1
S49A4	Cl spn sublx+ant thrc crd lsn	1
S49A9	Cl spn sublx+ant lmbar crd lsn	1
S498D	Cl spn sublux+post crv crd lsn	1
S498B	Cl spn sublux+ant cerv crd lsn	1
S490C	Cl spn dslc+cntrl cerv crd lsn	1
S4927	Cl spn dsl+lmbr crd lsn unsp	1
S1124	Cl spn #+pst thor crd lsn,T1-6	1
S1123	Cl spn #+cnt thor crd lsn,T1-6	1
S1122	Cl spn #+ant thor crd lsn,T1-6	1
S1120	Cl sp #+unsp thor crd lsn,T1-6	1
S1126	Cl sp #+unsp thor cd lsn,T7-12	1
S112A	Cl sp #+pst thor crd lsn,T7-12	1
S1129	Cl sp #+cnt thor crd lsn,T7-12	1
S1121	Cl sp #+cmpl thor crd lsn,T1-6	1
S1127	Cl sp #+cmp thor crd lsn,T7-12	1
S1128	Cl sp #+ant thor crd lsn,T7-12	1
S1500	Cl multi fractur of thor spine	1
SR160	Cl fract/th wth lw bck+plv+lmb	1
S112B	Cl # T7-12incomp cord lsn NOS	1
S1125	Cl # T1-6 incmpl cord lesn NOS	1
N301B	Chronic osteomyelitis-th spine	1
N301C	Chronic osteomyelitis-lu spine	1
N301A	Chronic osteomyelitis-Cx spine	1
N301	Chronic osteomyelitis	1
C052*	Chronic lymphocytic thyroiditis (Hashimotos's disease)	1

Read code	Description	Number of studies
16C9	Chronic low back pain	1
N090X	Chronic joint effusion	1
N3682	Chronic instability of knee	1
OX7289CB	Chronic Backache /ox	1
N3010	Chron.osteomyelitis-site unsp.	1
N3011	Chron.osteomyelitis-shoulder	1
N3018	Chron.osteomyelitis-other spec	1
N3019	Chron.osteomyelitis-mult.site	1
N301z	Chron.osteomyelitis NOS	1
N301L	Chron multifocal osteomyelitis	1
N301M	Chr osteomyel + draining sinus	1
N074	Chondromalacia patellae	1
N33z2	Chondromalacia NOS	1
N33zB	Chondrolysis	1
N022	Chondrocalcinosis unspecified	1
N022z	Chondrocalcinosis NOS	1
N02	Chondrocalcinosis	1
N0220	Chondrocalc.unsp.-site unspec.	1
N0221	Chondrocalc.unsp.-shoulder reg	1
N022y	Chondrocalc.unsp.-other spec.	1
N022x	Chondrocalc.unsp.-multipl.site	1
N020z	Chondrocalc.dicalc.phos.NOS	1
N021	Chondrocalc.-pyrophosph.cryst.	1
N021z	Chondrocalc.-pyrophosph.NOS	1
N020	Chondrocalc.-dicalc.phos.cryst	1
N0210	Chondroc.-pyrophos.-site unsp.	1
N0211	Chondroc.-pyrophos.-shoulder	1
N021y	Chondroc.-pyrophos.-other spec	1
N021x	Chondroc.-pyrophos.-mult.sites	1
N0216	Chondroc.-pyrophos.-knee	1
N0200	Chondroc.-dical.ph.-site unsp.	1
N0201	Chondroc.-dical.ph.-shoulder	1
N020y	Chondroc.-dical.ph.-other spec	1
N020x	Chondroc.-dical.ph.-mult.sites	1
N33zJ	Chondritis	1
182C	Chest wall pain	1
N035	Charcots arthropathy	1
N132	Cervicocranial syndrome	1
N133	Cervicobrachial syndrome	1
N148A	Cervico-thoracic instability	1
N1483	Cervico-thoracic ankylosis	1
N131	Cervicalgia	1
N138	Cervicalgia	1
N13y0	Cervical syndrome NEC	1
N119	Cervical spondylosis with radiculopathy	1
N11E	Cervical spondylosis	1
N110-1	Cervical spondylosis	1
N110	Cervical spond.- no myelopathy	1
N111	Cervical spond.+ myelopathy	1
N1489	Cervical spine instability	1
N1482	Cervical spine ankylosis	1
N130	Cervical spinal stenosis	1
N13y3	Cervical root syndrome	1

Read code	Description	Number of studies
N12A1	Cervical postlaminectomy syndr	1
N137	Cervical post.long.lig.ossific	1
SJ306	Cervical nerve root injury - C7	1
SJ305	Cervical nerve root injury - C6	1
SJ304	Cervical nerve root injury - C5	1
SJ303	Cervical nerve root injury - C4	1
SJ30	Cervical nerve root injury	1
N1113	Cervical myelopathy	1
N13	Cervical disorder NOS	1
N12z4	Cervical discitis	1
N120	Cervical disc displ.-no myelop	1
N1291	Cervical disc disord.+myelop.	1
N125	Cervical disc degeneration	1
S5701	Cervical ant.longit.lig.sprain	1
N13z	Cervical and neck disorders NOS	1
N12zH	Cerv disc disord + radiculopth	1
N21z0	Capsulitis NOS	1
N04y0	Caplan's syndrome	1
N3202	Calve's vertebral osteochondr.	1
N2454	Calf pain	1
N2226	Calcium deposit in bursa	1
N2111	Calcifying tendinitis shoulder	1
N12zB	Calcification of thoracic disc	1
N22y1	Calcification of tendon NOS	1
N12zF	Calcification of lumbar disc	1
N23y1	Calcification of ligament	1
N12z7	Calcification of cervical disc	1
N23y9	Calcific tendinitis	1
N2177	Calcaneal spur	1
N2315	Calc/ossif musc ass with burns	1
N0011	CREST syndrome	1
182Z.00	CHEST PAIN NOS	1
182..00	CHEST PAIN	1
S1007	C7 closed # - no cord lesion	1
S1006	C6 closed # - no cord lesion	1
1D12.00	C/O: stiffness	1
1D12	C/O: stiffness	1
16C7	C/O - upper back ache	1
1A53	C/O - lumbar pain	1
16C5	C/O - low back pain	1
N2118	Bursitis of shoulder	1
N223	Bursitis NOS	1
N2160-99	Bursitis - knee	1
N2160	Bursitis - knee	1
N221	Bunion	1
16BZ	Bruising symptom NOS	1
16B	Bruising symptom	1
16B2	Bruises easily	1
SE4z	Bruise NOS	1
SE41	Bruise - knee/lower leg	1
SE41-99	Bruise - knee/lower leg	1
SE23	Bruise - back	1
N11y0	Brucella spondylitis	1

Read code	Description	Number of studies
N3325	Brown tumour-hyperparathyroid	1
N301G	Brodie's abscess-thorac spine	1
N301H	Brodie's abscess-lumbar spine	1
N301F	Brodie's abscess-cervic spine	1
N302b	Brodie's abscess	1
N0503	Bouchard's nodes with arthropathy	1
N33z	Bone/cartilage disorder NOS	1
N33z0	Bone/cartilage dis.-unspecif.	1
N21z3	Bone spur NOS	1
N33A	Bone pain	1
N33zz	Bone or cartilage disorders NOS	1
N31z	Bone involvement in dis.EC NOS	1
N30z0	Bone infectn.NOS-site unspecif	1
N30z1	Bone infectn.NOS-shoulder	1
N30z9	Bone infectn.NOS-multiple site	1
N30z	Bone infection NOS	1
N30zz	Bone infection NOS	1
N3320	Bone cyst (localised),unspecif	1
N3y0	Biomec lesn,not elsewh clas	1
N2112	Bicipital tenosynovitis	1
N2134	Biceps tendinitis	1
N0120	Behcet's syndrome arthropathy	1
N2222	Beat knee	1
N0870	Bankart lesion	1
N224A	Baker's cyst	1
N224A-1	Baker's cyst	1
16	Baker's cyst	1
16C3	Backache with radiation	1
16CZ	Backache symptom NOS	1
16C	Backache symptom	1
16C2	Backache	1
N149	Back stiffness	1
S57	Back sprain excl. lumbosacral	1
16...	Back sprain NOS	1
S57z	Back sprain NOS	1
16C4	Back pain worse on sneezing	1
16C6	Back pain without radiat NOS	1
N145-2	Back pain unspecified	1
N143	Back pain - lower	1
UNMAPPC6	Back injury	1
N14	Back disorders - other	1
N14z	Back disorder/symptom NOS	1
OX8479	Back Strain/Sprain /ox	1
OX7289A	Back Pain With Sciatica /ox	1
N2455	Axillary pain	1
N3347	Avascular necrosis-other bone	1
N3345	Avascular necrosis, capitellum	1
N334	Avascular necrosis - bone	1
N334z	Avascular bone necrosis NOS	1
N3340	Avasc.bone necrosis site unsp.	1
N3383	Atrophic non-union of fracture	1
S5703	Atlanto-occipital joint sprain	1
N1487	Atlanto-occipital instability	1

Read code	Description	Number of studies
N1480	Atlanto-occipital ankylosis	1
S5702	Atlanto-axial joint sprain	1
N1488	Atlanto-axial instability	1
N1481	Atlanto-axial ankylosis	1
14OD	At risk of osteoporotic fracture	1
14O9	At risk of osteoporosis	1
N080z	Articular cartilage disord.NOS	1
N080	Articular cart.disor.excl.knee	1
N0800	Artic.cart.dis.-site unspecif.	1
N0801	Artic.cart.dis.-shoulder	1
N0807	Artic.cart.dis.-other specif.	1
N0808	Artic.cart.dis.-multiple sites	1
N080B	Artic cart disord oth j-should	1
N0539	Arthrosis of first carpometacarpal joint, unspecified	1
OX7131C	Arthrosis Spine /ox	1
N0312	Arthropathy-Whipple's disease	1
N034	Arthropathy+respiratory disord	1
N015	Arthropathy+other viral diseas	1
N01y	Arthropathy+other inf./parasit	1
N03y	Arthropathy+other condition EC	1
N015z	Arthropathy+oth.viral dis. NOS	1
N014	Arthropathy+oth.bacterial dis.	1
N017z	Arthropathy+helminthiasis NOS	1
N032	Arthropathy+haematological dis	1
N031	Arthropathy+gastrointestin.dis	1
N030	Arthropathy+endocr./metab.dis	1
N033	Arthropathy+dermatological dis	1
N01	Arthropathy with infections	1
N0310	Arthropathy in ulcerative colitis	1
N069	Arthropathy in neoplastic dis	1
N0302	Arthropathy in amyloidosis	1
N0148	Arthropathy in Whipple's disea	1
N0311	Arthropathy in Crohn's disease	1
N0320	Arthropathy due to haemophilia	1
N06z1	Arthropathy NOS-shoulder	1
N06z4	Arthropathy NOS-hand	1
N06z3	Arthropathy NOS-forearm	1
N06z7	Arthropathy NOS, of the ankle and foot	1
N017	Arthropathy + parasite infectn	1
N016z	Arthropathy + mycoses NOS	1
N016	Arthropathy + mycoses	1
N03z	Arthropathy + disorders EC NOS	1
N03	Arthropathy + disorders EC	1
N0y	Arthropathies OS	1
N0313	Arthropath follow intes bypass	1
N036	Arthrop-hypersensitivity reacn	1
N012	Arthrop+Behcet's syndrome	1
N0121	Arthrop+Behcet's synd-shoulder	1
N012y	Arthrop+Behcet's synd-oth spec	1
N012x	Arthrop+Behcet's synd-multiple	1
N012z	Arthrop+Behcet's synd NOS	1
N2330	Arthrogryposis	1
N0	Arthritis/arthrosis	1

Read code	Description	Number of studies
N11	Arthritis of spine	1
N010A	Arthritis in Lyme disease	1
OX6960T	Arthritis Psoriatic /ox	1
N06z-1	Arthritis	1
N094F	Arthralgia of wrist	1
N094N	Arthralgia of tibio-fibular joint	1
N094A	Arthralgia of shoulder	1
N094M	Arthralgia of knee	1
N094D	Arthralgia of elbow	1
N094P	Arthralgia of ankle	1
N094H	Arthralgia of PIP joint of finger	1
N094G	Arthralgia of MCP joint	1
N094T	Arthralgia of 1st MTP joint	1
N0942	Arthralgia - upper arm	1
N094B	Arthralgia - sternoclav joint	1
N0941	Arthralgia - shoulder	1
N0945	Arthralgia - pelvic/thigh	1
N0944	Arthralgia - hand	1
N0943	Arthralgia - forearm	1
N0947	Arthralgia - ankle/foot	1
N094C	Arthralgia - acromioclav joint	1
EGTON1	Arthralgia	1
N0150	Arthr.+oth.viral dis-site unsp	1
N0151	Arthr.+oth.viral dis-shoulder	1
N0140	Arthr.+oth.bact.dis-site unsp.	1
N0141	Arthr.+oth.bact.dis-shoulder	1
N014y	Arthr.+oth.bact.dis-other spec	1
N014x	Arthr.+oth.bact.dis-mult.sites	1
N014z	Arthr.+oth bact. disease NOS	1
N0160	Arthr.+mycoses-site unspecif.	1
N0161	Arthr.+mycoses-shoulder region	1
N016y	Arthr.+mycoses-other specified	1
N016x	Arthr.+mycoses-multiple sites	1
N0170	Arthr.+helminth.-site unspec.	1
N0171	Arthr.+helminth.-shoulder regn	1
N017y	Arthr.+helminth.-other specif.	1
N017x	Arthr.+helminth.-multiple site	1
N015y	Arthr+oth.viral dis-other spec	1
N015x	Arthr+oth.viral dis-mult.sites	1
N03x0	Arthr assoc oth dis-shoulder	1
N03xB	Arthr assoc oth dis-knee	1
N03x1	Arthr ass oth dis-sternoclav j	1
N03x2	Arthr ass oth dis-acromioclv j	1
N01yz	Arth+other infect./parasit.NOS	1
N01y0	Arth+oth.inf/para-site unspec.	1
N01y1	Arth+oth.inf/para-shoulder reg	1
N01yy	Arth+oth.inf/para-other specif	1
N01yx	Arth+oth.inf/para-multipl.site	1
N245-2	Arm pain	1
N32z0	Apophysitis NOS	1
N006	Antiphospholipid syndrome	1
1M12	Anterior knee pain	1
N094W	Anterior knee pain	1

Read code	Description	Number of studies
S4101	Anterior dislocation of shoulder	1
N12z9	Annular tear of thoracic disc	1
N12zD	Annular tear of lumbar disc	1
N12z5	Annular tear of cervical disc	1
N148	Ankylosis/instab Cx,Th,Lu spin	1
N085B	Ankylosis other joint-shoulder	1
N085P	Ankylosis of the knee joint	1
N085A	Ankylosis of shoulder joint	1
N0859	Ankylosis of multiple joints	1
N085z	Ankylosis of joint NOS	1
N085	Ankylosis of joint	1
N117	Ankylosing verteb.hyperostosis	1
N100*	Ankylosing spondylitis	1
N100	Ankylosing spondylitis	1
N245-1	Ankle pain	1
1M13	Ankle pain	1
N05z7-1	Ankle osteoarthritis NOS	1
N3384	Angular mal-union of fracture	1
N3322	Aneurysmal bone cyst	1
N2320	Amyotrophia NOS	1
N0620	Allergic arthritis-site unsp.	1
N0621	Allergic arthritis-shoulder	1
N0628	Allergic arthritis-other spec.	1
N0629	Allergic arthritis-multip.site	1
N062z	Allergic arthritis-NOS	1
N062	Allergic arthritis	1
N337z	Algoneurodystrophy NOS	1
N3373	Algodystrophy of knee	1
N04y2	Adult-onset Still's disease	1
N32y0	Adult osteochondrosis of spine	1
N005	Adult Still's Disease	1
N370	Adolescent postural kyphosis	1
N3737	Adolescent idiopath scoliosis	1
N210	Adhesive capsulitis - shoulde	1
N2156	Adductor tendinitis	1
N084D	Adduction contracture-shoulder	1
N0431	Acute polyartic.juvenile R.A.	1
N300B	Acute osteomyelitis-thor spine	1
N3000	Acute osteomyelitis-site unsp.	1
N3001	Acute osteomyelitis-shoulder	1
N300G	Acute osteomyelitis-scapula	1
N300R	Acute osteomyelitis-patella	1
N3008	Acute osteomyelitis-other spec	1
N3009	Acute osteomyelitis-mult.site	1
N300C	Acute osteomyelitis-lumb spine	1
N300F	Acute osteomyelitis-clavicle	1
N300A	Acute osteomyelitis-cerv spine	1
N300z	Acute osteomyelitis NOS	1
N300	Acute osteomyelitis	1
S460	Acute meniscal tear medial	1
S461	Acute meniscal tear lateral	1
N090Y	Acute joint effusion	1
N300Z	Acute haematogen osteomyelitis	1

Read code	Description	Number of studies
N145	Acute back pain - unspecified	1
N145-1	Acute back pain - unspecified	1
N141	Acute back pain - thoracic	1
N141-1	Acute back pain - thoracic	1
N142	Acute back pain - lumbar	1
N142-3	Acute back pain - lumbar	1
N12	Acute back pain - disc	1
N143-1	Acute back pain + sciatica	1
N06zA	Acute arthritis	1
EGTONAC1	Acromio-Clavicular Dislocation	1
N384	Acquired spondylolisthesis	1
N3720	Acquired postural lordosis	1
N3710	Acquired postural kyphosis	1
N372z	Acquired lordosis NOS	1
N372	Acquired lordosis	1
N371z	Acquired kyphosis NOS	1
N371	Acquired kyphosis	1
N366	Acquired knee deformity NOS	1
N37z0	Acquired hunchback	1
N3641	Acquired genu varum	1
N364z	Acquired genu valgum/varum NOS	1
N364	Acquired genu valgum/varum	1
N3640	Acquired genu valgum	1
N385	Acquired deformity spine NOS	1
N382	Acquired deformity of neck	1
N38z	Acquired deformity NOS	1
N38y0	Acquired clavicle deformity	1
1DCC.00	Aching muscles	1
1DCC	Aching muscles	1
N220H	Achilles tenosynovitis	1
N2174	Achilles tendinitis	1
N094-1	Ache in joint	1
S4604	Ac mnscl tr,med,periph,dtchmt	1
S4614	Ac mnscl tr,lat,periph,dtchmt	1
S4605	Ac mnscl tear,med,horiz clvge	1
S4615	Ac mnscl tear,lat,horiz clvge	1
S4602	Ac menscl tear,med,bckt hndle	1
S4612	Ac menscl tear,lat,bckt hndle	1
S4603	Ac meniscal tear,med,radial	1
S4601	Ac meniscal tear,med,post horn	1
S4600	Ac meniscal tear,med,ant horn	1
S4613	Ac meniscal tear,lat,radial	1
S4611	Ac meniscal tear,lat,post horn	1
S4610	Ac meniscal tear,lat,ant horn	1
N22y2	Abscess of tendon	1
N22yE	Abscess of bursa-shoulder	1
N22yJ	Abscess of bursa-knee	1
N22y3	Abscess of bursa	1
SD	Abrasions	1
N084C	Abduction contracture-shoulder	1
1969.00	Abdominal pain	1
EGTON251	? Frozen Right Shoulder	1
N11C1	2 lev lumbsac spond+radiculop	1

Read code	Description	Number of studies
N11C0	1 lev lumbsac spond+radiculop	1
HNG0160	(hn) Sports Injury	1
HNG0157	(hn) Spinal Injury	1
HNG0162	(hn) Soft tissue injuries	1
HNG0163	(hn) Rhematic problems	1
S11	#Vertebra + cord lesion	1
S10z	#Spine - no cord lesion - NOS	1
S10	#Spine - no cord lesion	1
S11z	#Spine + cord lesion NOS	1
S21z	#Scapula NOS	1
S21	#Scapula	1
S32z	#Patella NOS	1
S104	#Lumbar spine - no cord lesion	1
S32	#Knee-cap	1
S20z	#Clavicle NOS	1
S20	#Clavicle	1
S3zz	#Bones NOS	1
S4F	#-dslc/subluxation knee	1
S4A	#-dslc or subluxation shoulder	1

Table 10. List of Read codes used in the studies of male sexual dysfunction.

Read code	Description	Number of studies
E227311	Erectile dysfunction	3
K27y100	Impotence of organic origin	2
E227300	Impotence	2
Eu52213	[X]Psychogenic impotence	1
Eu52212	[X]Male erectile disorder	1
E227000	Unspecified psychosexual dysfunction	1
7C25E00	Treatment of erectile dysfunction NEC	1
7A6G000	Revascularisation for impotence	1
8IE8.00	Referral to erectile dysfunction clinic declined	1
8HTj.00	Referral to erectile dysfunction clinic	1
E227z00	Psychosexual dysfunction NOS	1
E227.00	Psychosexual dysfunction	1
E227700	Psychogenic dyspareunia	1
Z9E9.00	Provision of device for impotence	1
E227600	Premature ejaculation	1
7C25F00	Operations on penis for erectile dysfunction NEC	1
7A6G500	Ligation of penile veins for impotence	1
E227.11	Lack of libido	1
E227100	Inhibited sexual desire	1
E227500	Inhibited male orgasm	1
E227z11	Fear of ejaculation	1
K27y700	Erectile dysfunction due to diabetes mellitus	1
66Au.00	Diabetic erectile dysfunction review	1
66Av.00	Diabetic assessment of erectile dysfunction	1
1D1B.00	C/O erectile dysfunction	1
ZG43600	Advice on technique for impotence	1
67IA.00	Advice about impotence	1

Table 11. List of Read codes used in the studies of sleep disorder.

Read code	Description	Number of studies
1B1B.00	Cannot sleep - insomnia	1
1B1B.11	C/O - insomnia	1
1B1B100	Middle insomnia	1
1B1B200	Late insomnia	1
1B1Q.00	Poor sleep pattern	1
E274.00	Non-organic sleep disorders	1
E274.11	Hypersomnia of non-organic origin	1
E274.12	Insomnia due to nonorganic sleep disorder	1
E274000	Unspecified non-organic sleep disorder	1
E274100	Transient insomnia	1
E274111	Insomnia NOS	1
E274200	Persistent insomnia	1
E274300	Transient hypersomnia	1
E274311	Hypersomnia NOS	1
E274400	Persistent hypersomnia	1
E274500	Jet lag syndrome	1
E274600	Shifting sleep-work schedule	1
E274700	Somnambulism - sleep walking	1
E274800	Night terrors	1
E274900	Nightmares	1
E274A00	Sleep drunkenness	1
E274B00	Repeated rapid eye movement sleep interruptions	1
E274C00	Other sleep stage or arousal dysfunction	1
E274D00	Repetitive intrusions of sleep	1
E274D11	Restless sleep	1
E274E00	'Short-sleeper'	1
E274F00	Inversion of sleep rhythm	1
E274y00	Other non-organic sleep disorder	1
E274y11	Dreams	1
E274z00	Non-organic sleep disorder NOS	1
Eu51.00	[X]Nonorganic sleep disorders	1
Eu51000	[X]Nonorganic insomnia	1
Eu51100	[X]Nonorganic hypersomnia	1
Eu51200	[X]Nonorganic disorder of the sleep-wake schedule	1
Fy00.00	Disorders of initiating and maintaining sleep	1
Fy01.00	Disorders of excessive somnolence	1
Fy02.00	Disorders of the sleep-wake schedule	1
R005.00	[D]Sleep disturbances	1
R005.11	[D]Insomnia - symptom	1
R005.12	[D]Sleep rhythm problems	1
R005000	[D]Sleep disturbance, unspecified	1
R005100	[D]Insomnia with sleep apnoea	1
R005200	[D]Insomnia NOS	1
R005300	[D]Hypersomnia with sleep apnoea	1
R005311	[D]Sleep apnoea syndrome	1
R005312	[D]Syndrome sleep apnoea	1
R005400	[D]Hypersomnia NOS	1
R005500	[D]Sleep rhythm inversion	1
R005600	[D]Sleep rhythm irregular	1
R005700	[D]Sleep-wake rhythm non-24-hour cycle	1
R005800	[D]Sleep dysfunction with sleep stage disturbance	1

Read code	Description	Number of studies
R005900	[D]Sleep dysfunction with arousal disturbance	1
R005z00	[D]Sleep dysfunction NOS	1

Table 12. List of Read codes used in the studies of fatal and non-fatal self-harm.

Read code	Description	Number of studies
TK1y.00	Suicide and selfinflicted poisoning by other utility gas	8
TK21.00	Suicide and selfinflicted poisoning by other carbon monoxide	8
TK01000	Suicide and self inflicted injury by Amylobarbitone	8
TK03.00	Suicide + selfinflicted poisoning tranquilliser/psychotropic	8
TK0z.00	Suicide + selfinflicted poisoning by solid/liquid subst NOS	8
TK04.00	Suicide + selfinflicted poisoning by other drugs/medicines	8
TK02.00	Suicide + selfinflicted poisoning by oth sedatives/hypnotics	8
TK20.00	Suicide + selfinflicted poisoning by motor veh exhaust gas	8
TK05.00	Suicide + selfinflicted poisoning by drug or medicine NOS	8
TK07.00	Suicide + selfinflicted poisoning by corrosive/caustic subst	8
TK01.00	Suicide + selfinflicted poisoning by barbiturates	8
TK00.00	Suicide + selfinflicted poisoning by analgesic/antipyretic	8
TK06.00	Suicide + selfinflicted poisoning by agricultural chemical	8
U200.00	[X]Intent self poison/exposure to nonopioid analgesic	7
U20..11	[X]Deliberate drug overdose / other poisoning	7
TK70.00	Suicide+selfinflicted injury-jump from residential premises	7
TK71.00	Suicide+selfinflicted injury-jump from oth manmade structure	7
TK72.00	Suicide+selfinflicted injury-jump from natural sites	7
TK7z.00	Suicide+selfinflicted injury-jump from high place NOS	7
TK61.00	Suicide and selfinflicted injury by stabbing	7
TK51.00	Suicide and selfinflicted injury by shotgun	7
TKx2.00	Suicide and selfinflicted injury by scald	7
TKxy.00	Suicide and selfinflicted injury by other specified means	7
TK54.00	Suicide and selfinflicted injury by other firearm	7
TK52.00	Suicide and selfinflicted injury by hunting rifle	7
TK30.00	Suicide and selfinflicted injury by hanging	7
TK6z.00	Suicide and selfinflicted injury by cutting and stabbing NOS	7
TK60.00	Suicide and selfinflicted injury by cutting	7
TKx5.00	Suicide and selfinflicted injury by crashing motor vehicle	7
TKx1.00	Suicide and selfinflicted injury by burns or fire	7
TK...00	Suicide and selfinflicted injury	7
TK01400	Suicide and self inflicted injury by Phenobarbitone	7
TK01100	Suicide and self inflicted injury by Barbitone	7
TK...14	Suicide and self harm	7
TK0..00	Suicide + selfinflicted poisoning by solid/liquid substances	7
TK2..00	Suicide + selfinflicted poisoning by other gases and vapours	7
TK11.00	Suicide + selfinflicted poisoning by liquified petrol gas	7
TK2z.00	Suicide + selfinflicted poisoning by gases and vapours NOS	7
TK10.00	Suicide + selfinflicted poisoning by gas via pipeline	7
TK1z.00	Suicide + selfinflicted poisoning by domestic gases NOS	7
TKx0000	Suicide + selfinflicted injury-jumping before moving object	7
TK31.00	Suicide + selfinflicted injury by suffocation by plastic bag	7
TK3y.00	Suicide + selfinflicted inj oth mean hang/strangle/suffocate	7
TK3z.00	Suicide + selfinflicted inj by hang/strangle/suffocate NOS	7
TK...13	Poisoning - self-inflicted	7
TK...17	Para-suicide	7
TK...11	Cause of overdose - deliberate	7
TK...15	Attempted suicide	7
U2...13	[X]Suicide	6
U20C.11	[X]Self poisoning with weedkiller	6
U20C.12	[X]Self poisoning with paraquat	6

Read code	Description	Number of studies
U20A.11	[X]Self poisoning from glue solvent	6
U20B.11	[X]Self carbon monoxide poisoning	6
U2...15	[X]Para-suicide	6
U20..00	[X]Intentional self poisoning/exposure to noxious substances	6
U20A.00	[X]Intentional self poison organ solvent,halogen hydrocarb	6
U20y.00	[X]Intent self poison/exposure to unspecif chemical	6
U202.00	[X]Intent self poison/exposure to sedative hypnotic	6
U204.00	[X]Intent self poison/exposure to psychotropic drug	6
U20C.00	[X]Intent self poison/exposure to pesticide	6
U20B.00	[X]Intent self poison/exposure to other gas/vapour	6
U207.00	[X]Intent self poison/exposure to oth autonomic drug	6
U206.00	[X]Intent self poison/exposure to hallucinogen	6
U201.00	[X]Intent self poison/exposure to antiepileptic	6
U20yz00	[X]Intent self poison unspecif chemical unspecif place	6
U202z00	[X]Intent self poison sedative hypnotic unspecif place	6
U204z00	[X]Intent self poison psychotropic drug unspecif place	6
U204100	[X]Intent self poison psychotropic drug at res institut	6
U20Bz00	[X]Intent self poison other gas/vapour unspecif place	6
U208z00	[X]Intent self poison oth/unsp drug/medic unspecif place	6
U207z00	[X]Intent self poison oth autonomic drug unspecif place	6
U200z00	[X]Intent self poison nonopioid analgesic unspecif place	6
U200100	[X]Intent self poison nonopioid analgesic at res institut	6
U205z00	[X]Intent self poison narcotic drug unspecif place	6
U201z00	[X]Intent self poison antiepileptic unspecif place	6
U206400	[X]Intent self pois hallucinogen in street/highway	6
U20y000	[X]Int self poison/exposure to unspecif chemical at home	6
U202000	[X]Int self poison/exposure to sedative hypnotic at home	6
U204000	[X]Int self poison/exposure to psychotropic drug at home	6
U20C000	[X]Int self poison/exposure to pesticide at home	6
U208.00	[X]Int self poison/exposure to other/unspec drug/medicament	6
U20B000	[X]Int self poison/exposure to other gas/vapour at home	6
U207000	[X]Int self poison/exposure to oth autonomic drug at home	6
U200000	[X]Int self poison/exposure to nonopioid analgesic at home	6
U205000	[X]Int self poison/exposure to narcotic drug at home	6
U201000	[X]Int self poison/exposure to antiepileptic at home	6
U20y200	[X]Int self poison unspecif chemical school/pub admin area	6
U202y00	[X]Int self poison sedative hypnotic other spec place	6
U204y00	[X]Int self poison psychotropic drug other spec place	6
U20Cy00	[X]Int self poison pesticide other spec place	6
U20B200	[X]Int self poison other gas/vapour school/pub admin area	6
U20By00	[X]Int self poison other gas/vapour other spec place	6
U208y00	[X]Int self poison oth/unsp drug/medic other spec place	6
U20A400	[X]Int self poison org solvent,halogen hydrocarb,in highway	6
U200y00	[X]Int self poison nonopioid analgesic other spec place	6
U205y00	[X]Int self poison narcotic drug other spec place	6
U2...14	[X]Attempted suicide	6
TKx7.00	Suicide and selfinflicted injury caustic subst, excl poison	6
TKxz.00	Suicide and selfinflicted injury by other means NOS	6
TKx..00	Suicide and selfinflicted injury by other means	6
TK7..00	Suicide and selfinflicted injury by jumping from high place	6
TKx3.00	Suicide and selfinflicted injury by extremes of cold	6
TKx4.00	Suicide and selfinflicted injury by electrocution	6
TK4..00	Suicide and selfinflicted injury by drowning	6

Read code	Description	Number of studies
TK6..00	Suicide and selfinflicted injury by cutting and stabbing	6
TKz..00	Suicide and selfinflicted injury NOS	6
TK1..00	Suicide + selfinflicted poisoning by gases in domestic use	6
TKx0.00	Suicide + selfinflicted injury-jump/lie before moving object	6
TK3..00	Suicide + selfinflicted injury by hang/strangulate/suffocate	6
U2y0.00	[X]Intentionl self harm by oth specif means occurrn at home	5
U2z0.00	[X]Intentional self harm by unspecif means occurrn at home	5
U290.00	[X]Intentional self harm by sharp object occurrence at home	5
U29z.00	[X]Intentional self harm by sharp object occ unspecif place	5
U2A0.00	[X]Intentional self harm by blunt object occurrence at home	5
U270.00	[X]Intention self harm by smoke fire/flames occurrn at home	5
U294.00	[X]Intention self harm by sharp object occ street/highway	5
U29y.00	[X]Intention self harm by sharp object occ oth specif place	5
U205.00	[X]Intent self poison/exposure to narcotic drug	5
U209.00	[X]Intent self poison/exposure to alcohol	5
U209z00	[X]Intent self poison alcohol unspecif place	5
U202400	[X]Intent self pois sedative hypnotic in street/highway	5
U208400	[X]Intent self pois oth/unsp drug/medic in street/highway	5
U20A000	[X]Intent self pois organ solvent,halogen hydrocarb, home	5
U200500	[X]Intent self pois nonopioid analgesic trade/service area	5
U250.00	[X]Intent self harm oth/unspecif firearm disch occ at home	5
U2D6.00	[X]Intent self harm crash motor vehic occ indust/constr area	5
U2zy.00	[X]Intent self harm by unspecif means occ oth specif place	5
U2zz.00	[X]Intent self harm by unspecif means occ at unspecif place	5
U2z2.00	[X]Intent self harm by unspec mean occ sch/ins/pub adm area	5
U27z.00	[X]Intent self harm by smoke fire/flames occ unspecif place	5
U274.00	[X]Intent self harm by smoke fire/flame occ street/highway	5
U291.00	[X]Intent self harm by sharp object occ resident instit'n	5
U2yz.00	[X]Intent self harm by oth specif means occ unspecif place	5
U2y1.00	[X]Intent self harm by oth specif means occ resid instit'n	5
U2B0.00	[X]Intent self harm by jumping from high place occ at home	5
U2B4.00	[X]Intent self harm by jump from high place occ street/h'way	5
U211.00	[X]Intent self harm by hangng strangult/suffoct resid instit	5
U21z.00	[X]Intent self harm by hangng strangul/suffoct unspecif plce	5
U21y.00	[X]Intent self harm by hangng strangul/suffoct oth spec plce	5
U210.00	[X]Intent self harm by hanging strangulat/suffocat occ home	5
U221.00	[X]Intent self harm by drowning/submersn occ resid instit'n	5
U22z.00	[X]Intent self harm by drown/submersn occ unspecified place	5
U22y.00	[X]Intent self harm by drown/submersn occ oth specif place	5
U2D0.00	[X]Intent self harm by crash of motor vehicl occurrn at home	5
U2D4.00	[X]Intent self harm by crash motor vehicl occ street/highway	5
U2A3.00	[X]Intent self harm by blunt object occ sports/athlet area	5
U2A1.00	[X]Intent self harm by blunt object occ resident instit'n	5
U242.00	[X]Int slf hrm rifl s'gun/lrg frarm dis sch/ins/pub adm area	5
U208000	[X]Int self poison/exposure to oth/unsp drug/medicam home	5
U209y00	[X]Int self poison alcohol other spec place	5
U20Az00	[X]Int self pois org solv,halogen hydrocarb, unspec place	5
U241.00	[X]Int self harm rifl s'gun/lrg frarm disch occ resid instit	5
U2C4.00	[X]Int self harm jump/lying befr mov obje occ street/highway	5
U2C1.00	[X]Int self harm jump/lying befr mov obje occ resid instit'n	5
U2Cy.00	[X]Int self harm jump/lying bef mov obje occ oth specif plce	5
U2Bz.00	[X]Int self harm by jump from high place occ unspecif place	5
U2By.00	[X]Int self harm by jump from high place occ oth specif plce	5

Read code	Description	Number of studies
U2B6.00	[X]Int self harm by jump from high place indust/constr area	5
TK5z.00	Suicide and selfinflicted injury by firearms/explosives NOS	5
TK5..00	Suicide and selfinflicted injury by firearms and explosives	5
TKx6.00	Suicide and selfinflicted injury by crashing of aircraft	5
TK60111	Slashed wrists self inflicted	5
TK60100	Self inflicted lacerations to wrist	5
U202.13	[X]Overdose - temazepam	4
U202.11	[X]Overdose - sleeping tabs	4
U202.15	[X]Overdose - nitrazepam	4
U202.12	[X]Overdose - diazepam	4
U202.16	[X]Overdose - benzodiazepine	4
U202.17	[X]Overdose - barbiturate	4
U204.11	[X]Overdose - antidepressant	4
U202.18	[X]Overdose - amobarbital	4
U204.12	[X]Overdose - amitriptyline	4
U204.13	[X]Overdose - SSRI	4
U2z..00	[X]Intentional self harm by unspecified means	4
U27..00	[X]Intentional self harm by smoke, fire and flames	4
U29..00	[X]Intentional self harm by sharp object	4
U2y..00	[X]Intentional self harm by other specified means	4
U2B..00	[X]Intentional self harm by jumping from a high place	4
U26..00	[X]Intentional self harm by explosive material	4
U22..00	[X]Intentional self harm by drowning and submersion	4
U2D..00	[X]Intentional self harm by crashing of motor vehicle	4
U2A..00	[X]Intentional self harm by blunt object	4
U200400	[X]Intent self pois nonopioid analgesic in street/highway	4
U28z.00	[X]Intent self harm by steam hot vapour/obj occ unspec place	4
U280.00	[X]Intent self harm by steam hot vapour/hot obj occ at home	4
U24..00	[X]Intent self harm by rifle shotgun/larger firearm disch	4
U25..00	[X]Intent self harm by other/unspecified firearm discharge	4
U2C..00	[X]Intent self harm by jumping / lying before moving object	4
U21..00	[X]Intent self harm by hanging strangulation / suffocation	4
U212.00	[X]Inten slf harm hang strang/suffc sch oth ins/pub adm area	4
U209000	[X]Int self poison/exposure to alcohol at home	4
U41..00	[X]Hanging strangulation + suffocation undetermined intent	4
U4Bz.00	[X]Fall jump/push frm high plce undt intnt occ unspecif plce	4
U720.00	[X]Sequelae of intentional self-harm	3
U2...11	[X]Self inflicted injury	3
U44..00	[X]Rifle shotgun+larger firearm discharge undetermin intent	3
U40y.00	[X]Poisoning/exposure, ? intent, to unspecif chemical	3
U402.00	[X]Poisoning/exposure, ? intent, to sedative hypnotic	3
U404.00	[X]Poisoning/exposure, ? intent, to psychotropic drug	3
U40C.00	[X]Poisoning/exposure, ? intent, to pesticide	3
U40B.00	[X]Poisoning/exposure, ? intent, to other gas/vapour	3
U400.00	[X]Poisoning/exposure, ? intent, to nonopioid analgesic	3
U405.00	[X]Poisoning/exposure, ? intent, to narcotic drug	3
U409.00	[X]Poisoning/exposure, ? intent, to alcohol	3
U40..00	[X]Poisoning/expos to noxious substance,undetermined intent	3
U408.00	[X]Poison/exposure, ?intent, to other/unspec drug/medicament	3
U408000	[X]Poison/exposure ?intent, to oth/unsp drug/medicam home	3
U409000	[X]Poison/exposure ?intent, to alcohol at home	3
U40y600	[X]Poison/expos ?intent unspec chemic indust/construct area	3
U200.11	[X]Overdose - paracetamol	3

Read code	Description	Number of studies
U200.12	[X]Overdose - ibuprofen	3
U200.13	[X]Overdose - aspirin	3
U45..00	[X]Other+unspecified firearm discharge undetermined intent	3
U2...00	[X]Intentional self-harm	3
U28..00	[X]Intentional self harm by steam hot vapours / hot objects	3
U216.00	[X]Intent self harm by hang strangl/suffc indust/constr area	3
U220.00	[X]Intent self harm by drowning/submersion occurrn at home	3
U206000	[X]Int self poison/exposure to hallucinogen at home	3
U2...12	[X]Injury - self-inflicted	3
U410.00	[X]Hanging strangulat+suffocat undet intent occurrn at home	3
U4B..00	[X]Falling jumping/pushed from high place undeterm intent	3
U30..11	[X]Deliberate drug poisoning	3
ZX1R.00	Throwing self in front of vehicle	3
ZX1Q.00	Throwing self in front of train	3
TK53.00	Suicide and selfinflicted injury by military firearms	3
TK01z00	Suicide and self inflicted injury by barbiturates	3
TK2y.00	Suicide + selfinflicted poisoning by other gases and vapours	3
TKx0z00	Suicide + selfinflicted inj-jump/lie before moving obj NOS	3
ZX1N.00	Stabbing self	3
ZX1M.00	Shooting self	3
ZX1K.12	Setting self alight	3
ZX1K.11	Setting fire to self	3
ZX1H200	Self-suffocation	3
ZX1H100	Self-strangulation	3
ZX1K.00	Self-incineration	3
ZX1J.00	Self-electrocution	3
ZX1H.00	Self-asphyxiation	3
SL...15	Overdose of drug	3
SL...14	Overdose of biological substance	3
TKy..00	Late effects of selfinflicted injury	3
ZX1Q.11	Jumping under train	3
ZX1B.00	Jumping from height	3
ZX1B300	Jumping from cliff	3
ZX1B100	Jumping from building	3
ZX1B200	Jumping from bridge	3
TK...12	Injury - self-inflicted	3
ZX18.00	Hanging self	3
SLHz.00	Drug and medicament poisoning NOS	3
ZX15.00	Drowning self	3
U72..00	[X]Sequel intentn self-harm assault+event of undeterm intent	2
ZX1LD00	[X]Self mutilation	2
U40y000	[X]Poison/exposure ?intent, to unspecif chemical at home	2
U40C000	[X]Poison/exposure ?intent, to pesticide at home	2
U40A.00	[X]Pois/exposure,?intent,to organ solvent,halogen hydrocarb	2
U40y400	[X]Pois/expos ?intent unspecif chemical in street/highway	2
U40yz00	[X]Pois/expos ?intent to unspecif chemical unspecif place	2
U402z00	[X]Pois/expos ?intent to sedative hypnotic unspecif place	2
U408z00	[X]Pois/expos ?intent to oth/unsp drug/medic unspecif place	2
U409z00	[X]Pois/expos ?intent to alcohol unspecif place	2
U40B400	[X]Pois/expos ?intent other gas/vapour in street/highway	2
U409400	[X]Pois/expos ?intent alcohol in street/highway	2
U406y00	[X]Pois/exp ?intent to hallucinogen other spec place	2
U404300	[X]Pois/exp ?intent psychotropic drug in sport/athletic area	2

Read code	Description	Number of studies
U40A300	[X]Pois/exp ?intent org solvent,halogen hydrocarb,sport area	2
U409200	[X]Pois/exp ?intent alcohol school/pub admin area	2
U205.11	[X]Overdose - heroin	2
U2Az.00	[X]Intentional self harm by blunt object occ unspecif place	2
U206z00	[X]Intent self poison hallucinogen unspecif place	2
U209400	[X]Intent self pois alcohol in street/highway	2
U292.00	[X]Intent self harm sharp obj occ sch oth ins/pub adm area	2
U2yy.00	[X]Intent self harm oth specif means occ oth specif place	2
U2y6.00	[X]Intent self harm oth specif means occ indust/constr area	2
U2z1.00	[X]Intent self harm by unspecif means occurrn resid instit'n	2
U27y.00	[X]Intent self harm by smoke fire/flame occ oth specif plce	2
U295.00	[X]Intent self harm by sharp object occ trade/service area	2
U296.00	[X]Intent self harm by sharp object occ indust/constr area	2
U2B1.00	[X]Intent self harm by jump from high place occ resid instit	2
U213.00	[X]Intent self harm by hang strangl/suffc sport/athlet area	2
U2Dz.00	[X]Intent self harm by crash motor vehic occ unspecif place	2
U2A2.00	[X]Intent self harm blunt obj occ sch oth ins/pub adm area	2
U20Ay00	[X]Int self pois org solv,halogen hydrocarb,oth spec place	2
U200600	[X]Int self pois nonopioid analgesic indust/construct area	2
U282.00	[X]Int self harm by steam hot vapor/obj sch/ins/pub adm area	2
U4C..00	[X]Falling lying running befor/into moving obj undet intent	2
U4B6.00	[X]Fall jump/push frm high plce undt intn indust/constr area	2
U47..00	[X]Exposure to smoke, fire and flames, undetermined intent	2
U470.00	[X]Exposure to smoke fire+flame undeterm intent occ at home	2
U42..00	[X]Drowning and submersion, undetermined intent	2
U4D7.00	[X]Crashng of motor vehicle undetermined intent occ on farm	2
U4D0.00	[X]Crashng of motor vehicle undetermined intent occ at home	2
U4D..00	[X]Crashing of motor vehicle, undetermined intent	2
U4D3.00	[X]Crash of motor vehicle undeterm intent sport/athlet area	2
U4A5.00	[X]Contct wth blunt obj undet intent occ trade/service area	2
U4A3.00	[X]Contct wth blunt obj undet intent occ sport/athletic area	2
U4A0.00	[X]Contact wth blunt object undetermined intent occ at home	2
U48..00	[X]Contact with steam hot vapours+objects undetermn intent	2
U49..00	[X]Contact with sharp object, undetermined intent	2
U49z.00	[X]Contact with sharp obj undeterm intent occ unspecif place	2
U4A..00	[X]Contact with blunt object, undetermined intent	2
U4Az.00	[X]Contact with blunt obj undeterm intent occ unspecif place	2
ZX1S.00	Throwing self onto floor	2
1BDA.00	Thoughts of deliberate self harm	2
1BD3.00	Suicidal plans	2
1BD1.00	Suicidal ideation	2
1B19.11	Suicidal - symptom	2
1B19.00	Suicidal	2
ZX19200	Slapping self	2
ZX1I.00	Self-scalding	2
ZX1L300	Self-mutilation of penis	2
ZX1L100	Self-mutilation of hands	2
ZX1L200	Self-mutilation of genitalia	2
ZX1L600	Self-mutilation of ears	2
ZX1L.00	Self-mutilation	2
ZX1G.00	Scratches self	2
ZX19100	Punching self	2
TN...11	Poisoning undetermined - accidentally or purposely inflicted	2

Read code	Description	Number of studies
ZX1E.00	Pinching self	2
ZX1C.00	Nipping self	2
14K1.00	Intentional overdose of prescription only medication	2
TN0..00	Injury ?accidental, poisoning by solid/liquid substances	2
TN0z.00	Injury ?accidental, poisoning by solid or liquid subst NOS	2
TN2y.00	Injury ?accidental, poisoning by other spec gas or vapour	2
TN04.00	Injury ?accidental, poisoning by other spec drug/medicament	2
TN02.00	Injury ?accidental, poisoning by other sedative/hypnotic	2
TN2..00	Injury ?accidental, poisoning by other gases	2
TN21.00	Injury ?accidental, poisoning by other carbon monoxide	2
TN20.00	Injury ?accidental, poisoning by motor vehicle exhaust gas	2
TN11.00	Injury ?accidental, poisoning by liquid petrol gas	2
TN1..00	Injury ?accidental, poisoning by gases in domestic use	2
TN1z.00	Injury ?accidental, poisoning by gas in domestic use NOS	2
TN10.00	Injury ?accidental, poisoning by gas distributed by pipeline	2
TN05.00	Injury ?accidental, poisoning by drug or medicament NOS	2
TN06.00	Injury ?accidental, poisoning by corrosive/caustic substance	2
TN08.00	Injury ?accidental, poisoning by arsenic or its compounds	2
TN00.00	Injury ?accidental, poisoning by analgesic or anti-pyretic	2
TN07.00	Injury ?accidental, poisoning by agricultural chemicals	2
TN3..00	Injury ?accidental, hanging, strangulation and suffocation	2
TN30.00	Injury ?accidental, hanging	2
TN01300	Injury ?accidental poisoning by Pentobarbitone	2
ZRLfC12	HoNOS item 2 - non-accidental self injury	2
ZX19.00	Hitting self	2
ZX13.00	Cutting self	2
ZX13.11	Cuts self	2
ZX12.00	Burning self	2
ZX11.00	Biting self	2
ZX11.11	Bites self	2
SL90.00	Antidepressant poisoning	2
8G6Z.00	Anti-suicide psychotherapy NOS	2
8G6..00	Anti-suicide psychotherapy	2
SL90z00	Anti-depressant poisoning NOS	2
U4z..00	[X]Unspecified event, undetermined intent	1
U4zz.00	[X]Unspecif event undeterm intent occurrn unspecif place	1
U4z3.00	[X]Unspecif event undeterm intent occurrn sport/athlet area	1
U4zy.00	[X]Unspecif event undeterm intent occurrn oth specif place	1
U4z6.00	[X]Unspecif event undeterm intent occurrn indust/constr area	1
SyuG.00	[X]Toxic effects of substances chiefly nonmedicinal source	1
SyuG700	[X]Toxic effects of other specified gases, fumes & vapours	1
SyuGH00	[X]Toxic effect of paints and dyes, NEC	1
SyuGJ00	[X]Toxic effect of other specified substances	1
SyuG900	[X]Toxic effect of other pesticides	1
SyuG800	[X]Toxic effect of other insecticides	1
SyuGC00	[X]Toxic effect of other ingested (parts of) plant(s)	1
SyuG000	[X]Toxic effect of other alcohols	1
U2E..00	[X]Self mutilation	1
U406.00	[X]Poisoning/exposure, ? intent, to hallucinogen	1
SyuFM00	[X]Poisoning by other psychotropic drugs, NEC	1
SyuFB00	[X]Poisoning by other opioids	1
SyuFW00	[X]Poisoning by other laxatives, incl intestin atonia drugs	1
SyuFT00	[X]Poisoning by other antihypertensive drugs, NEC	1

Read code	Description	Number of studies
SyuFD00	[X]Poisoning by other and unspecified narcotics	1
SyuFA00	[X]Poisoning by other analgesics, not elsewhere classified	1
SyuF900	[X]Poisoning by oth nonsteroidal anti-inflamm drugs [NSAID]	1
SyuFc00	[X]Poisoning by oth & unspecif drugs & biologic substances	1
SyuF.00	[X]Poisoning by drugs and biological substances	1
U40Bz00	[X]Pois/expos ?intent to other gas/vapour unspecif place	1
U409100	[X]Pois/expos ?intent to alcohol at res institut	1
U408400	[X]Pois/expos ?intent oth/unsp drug/medic in street/highway	1
U40yy00	[X]Pois/exp ?intent to unspecif chemical other spec place	1
U40By00	[X]Pois/exp ?intent to other gas/vapour other spec place	1
U202.14	[X]Overdose - flurazepam	1
U4y..00	[X]Other specified events, undetermined intent	1
U4y0.00	[X]Other specified event undetermind intent occurrn at home	1
U4y3.00	[X]Oth specif event undetermin intent occ sport/athlet area	1
Eu15000	[X]Mnt/beh dis due oth stim inc caffein: acute intoxication	1
Eu18000	[X]Mental & behav dis due vol solvents: acute intoxication	1
Eu17000	[X]Mental & behav dis due to use tobacco: acute intoxication	1
Eu11000	[X]Mental & behav dis due to use opioids: acute intoxication	1
Eu14000	[X]Mental & behav dis due to use cocaine: acute intoxication	1
Eu10000	[X]Mental & behav dis due to use alcohol: acute intoxication	1
Eu13000	[X]Mental & behav dis due seds/hypntcs: acute intoxication	1
Eu16000	[X]Mental & behav dis due hallucinogens: acute intoxicatn	1
Eu12000	[X]Mental & behav dis due cannabinoids: acute intoxication	1
Eu1A000	[X]Ment behav dis due use crack cocaine: acute intoxication	1
U39..11	[X]Intentionally shot with shotgun	1
U38..11	[X]Intentionally shot with handgun	1
U41z.00	[X]Hangng strangult+suffoct undet intent occ unspecif place	1
U4B0.00	[X]Fallng jumpng/push frm high place undet intent occ home	1
U81..00	[X]Evid of alcohol involv determind by level of intoxication	1
U4D1.00	[X]Crashng of motor vehicle undeterm intent resident instit	1
U466.00	[X]Contct wth explosiv materl undet intnt indust/constr area	1
U4A6.00	[X]Contct wth blunt obj undet intent occ industr/constr area	1
U46..00	[X]Contact with explosive material, undetermined intent	1
U4Ay.00	[X]Contact with blunt obj undeter intent occ oth specif plce	1
U1AC.11	[X]Accidental poisoning with weedkiller	1
U1A2.13	[X]Accidental poisoning with temazepam	1
U1A2.11	[X]Accidental poisoning with sleeping tablets	1
U1AC.12	[X]Accidental poisoning with paraquat	1
U1A0.11	[X]Accidental poisoning with paracetamol	1
U1A2.15	[X]Accidental poisoning with nitrazepam	1
U1A0.12	[X]Accidental poisoning with ibuprofen	1
U1A5.11	[X]Accidental poisoning with heroin	1
U1A2.12	[X]Accidental poisoning with diazepam	1
U1A2.16	[X]Accidental poisoning with benzodiazepine	1
U1A2.17	[X]Accidental poisoning with barbiturate	1
U1A0.13	[X]Accidental poisoning with aspirin	1
U1A4.11	[X]Accidental poisoning with antidepressant	1
U1A4.12	[X]Accidental poisoning with amitriptyline	1
U1A4.13	[X]Accidental poisoning with SSRI	1
U1AA.11	[X]Accidental poisoning from glue solvent	1
U1AD.00	[X]Accidental poisoning by and exposure to amfetamine	1
U1A..00	[X]Accidental poisoning by + exposure to noxious substances	1
U1A..12	[X]Accidental drug overdose / other poisoning	1

Read code	Description	Number of studies
U1A..11	[X]Accidental drug / other poisoning	1
U1AB.11	[X]Accidental carbon monoxide poisoning	1
U1Ay.00	[X]Accident poisoning/exposure to unspecif chemical	1
U1A2.00	[X]Accident poisoning/exposure to sedative hypnotic	1
U1A4.00	[X]Accident poisoning/exposure to psychotropic drug	1
U1AC.00	[X]Accident poisoning/exposure to pesticide	1
U1AB.00	[X]Accident poisoning/exposure to other gas/vapour	1
U1A7.00	[X]Accident poisoning/exposure to oth autonomic drug	1
U1A0.00	[X]Accident poisoning/exposure to nonopioid analgesic	1
U1A5.00	[X]Accident poisoning/exposure to narcotic drug	1
U1A6.00	[X]Accident poisoning/exposure to hallucinogen	1
U1A3.00	[X]Accident poisoning/exposure to antiparkinson drug	1
U1A1.00	[X]Accident poisoning/exposure to antiepileptic	1
U1A9.00	[X]Accident poisoning/exposure to alcohol	1
U1AD000	[X]Accident poisoning by and exposure to amphetamine - home	1
U1Ay700	[X]Accident poison/exposure to unspecif chemical on farm	1
U1Ay000	[X]Accident poison/exposure to unspecif chemical at home	1
U1A2000	[X]Accident poison/exposure to sedative hypnotic at home	1
U1A4000	[X]Accident poison/exposure to psychotropic drug at home	1
U1AC700	[X]Accident poison/exposure to pesticide on farm	1
U1AC000	[X]Accident poison/exposure to pesticide at home	1
U1A8.00	[X]Accident poison/exposure to other/unspec drug/medicament	1
U1AB700	[X]Accident poison/exposure to other gas/vapour on farm	1
U1AB000	[X]Accident poison/exposure to other gas/vapour at home	1
U1A8000	[X]Accident poison/exposure to oth/unsp drug/medicam home	1
U1A7000	[X]Accident poison/exposure to oth autonomic drug at home	1
U1A0000	[X]Accident poison/exposure to nonopioid analgesic at home	1
U1A5000	[X]Accident poison/exposure to narcotic drug at home	1
U1A1000	[X]Accident poison/exposure to antiepileptic at home	1
U1A9000	[X]Accident poison/exposure to alcohol at home	1
U1AA.00	[X]Accid poison/exposure to organ solvent,halogen hydrocarb	1
U1Ay500	[X]Accid poison/expos unspecif chemical trade/service area	1
U1Ayz00	[X]Accid poison/expos to unspecif chemical unspecif place	1
U1Ay100	[X]Accid poison/expos to unspecif chemical at res institut	1
U1A2z00	[X]Accid poison/expos to sedative hypnotic unspecif place	1
U1A4100	[X]Accid poison/expos to psychotropic drug at res institut	1
U1ABz00	[X]Accid poison/expos to other gas/vapour unspecif place	1
U1A8100	[X]Accid poison/expos to oth/unsp drug/medicam res institut	1
U1A8z00	[X]Accid poison/expos to oth/unsp drug/medic unspecif place	1
U1A7z00	[X]Accid poison/expos to oth autonomic drug unspecif place	1
U1A0z00	[X]Accid poison/expos to nonopioid analgesic unspecif place	1
U1A5z00	[X]Accid poison/expos to narcotic drug unspecif place	1
U1A9z00	[X]Accid poison/expos to alcohol unspecif place	1
U1AA000	[X]Accid poison/expos organ solvent,halogen hydrocarb, home	1
U1A3500	[X]Accid poison/expos antiparkinson drug trade/service area	1
U1A9500	[X]Accid poison/expos alcohol trade/service area	1
U1A9400	[X]Accid poison/expos alcohol in street/highway	1
U1Ay200	[X]Acc poison/expos unspecif chemical school/pub admin area	1
U1A4200	[X]Acc poison/expos psychotropic drug school/pub admin area	1
U1AA100	[X]Acc poison/expos org solvent,halogen hydrocarb,res instit	1
U1A9200	[X]Acc poison/expos alcohol school/pub admin area	1
U1ADz00	[X]Acc poison by and exposure to amphetamine - unspec places	1
Sy...00	[X] Injury and poisoning classification terms	1

Read code	Description	Number of studies
U60F412	[X] Adverse reaction to acetylcysteine	1
ZV15600	[V]Personal history of poisoning	1
ZV4C400	[V]Occupational exposure to toxic agents in agriculture	1
ZV71A00	[V]Obs for suspected toxic effect from ingested substance	1
SLC7100	Zinc salt poisoning	1
SM01100	Wood alcohol causing toxic effect	1
SLEz.00	Water, mineral or uric acid metabolism poisoning NOS	1
SLE..00	Water, mineral and urate metabolism poisoning	1
SL42300	Warfarin sodium poisoning	1
SL42400	Warfarin poisoning	1
SL35z00	Vitamin poisoning NOS	1
SL35.00	Vitamin poisoning NEC	1
SL35100	Vitamin D poisoning	1
SL35000	Vitamin A poisoning	1
SL44300	Urokinase poisoning	1
SLE7.00	Uric acid drug poisoning	1
SLE7.11	Urate metabolism drug poisoning	1
SM9z.00	Unspecified substance causing toxic effect NOS	1
SL50000	Unspecified opium poisoning	1
SL30400	Tripelennamine poisoning	1
SL60100	Trimethadione poisoning	1
SL92200	Trifluperidol poisoning	1
SM23100	Trichloroethylene causing toxic effect	1
SL90300	Trazodone poisoning	1
SL95z00	Tranquilliser poisoning NOS	1
SL9..11	Tranquilliser poisoning	1
TE57.00	Toxic reactions caused by other plants	1
F036200	Toxic encephalitis due to thallium	1
F036100	Toxic encephalitis due to mercury	1
F036000	Toxic encephalitis due to lead	1
SMC..00	Toxic effect of tobacco and nicotine	1
SM57.00	Toxic effect of tin and its compounds	1
SM9A.00	Toxic effect of rodenticides	1
SMX..00	Toxic effect of paints and dyes, NEC	1
SM9X.00	Toxic effect of nitroglycerin & oth nitric acids & ester	1
SM79.00	Toxic effect of hydrogen sulfide	1
SM15.00	Toxic effect of homologues of benzene	1
SM98.00	Toxic effect of herbicides and fungicides	1
SMB..00	Toxic effect of formaldehyde	1
SM78.00	Toxic effect of fluorine gas and hydrogen fluoride	1
SM23200	Toxic effect of chloroform	1
SM7A.00	Toxic effect of carbon dioxide	1
SLG7.00	Topical dental drug poisoning	1
SL85.00	Topical and infiltration anaesthetic agent poisoning	1
SL16200	Tiabendazole poisoning	1
SL27.00	Thyroid hormone and thyroid derivatives poisoning	1
SL27z00	Thyroid hormone and thyroid derivative poisoning NOS	1
SL27300	Thyroglobulin poisoning	1
SL91400	Thioridazine poisoning	1
SLE1100	Theophylline poisoning	1
SL04000	Tetracycline poisoning	1
SL04z00	Tetracycline group poisoning NOS	1
SL04.00	Tetracycline group poisoning	1

Read code	Description	Number of studies
SM23000	Tetrachloroethylene causing toxic effect	1
SL85300	Tetracaine poisoning	1
SL21300	Testosterone poisoning	1
SLF5200	Terpin hydrate poisoning	1
SL3z.00	Systemic agent poisoning NOS	1
SLB2.00	Sympathomimetic poisoning	1
SM31200	Sulphuric acid causing toxic effect	1
SM73.00	Sulphur dioxide causing toxic effect	1
SL1x400	Sulphone poisoning	1
SL10.00	Sulphonamide poisoning	1
SL10200	Sulfamethoxazole poisoning	1
SL10000	Sulfadiazine poisoning	1
1BD4.00	Suicide risk	1
ZRn3.00	Suicide intent score subscale - attempt circumstances	1
SN47100	Suffocation by strangulation	1
SL06300	Streptomycin poisoning	1
SL44200	Streptokinase poisoning	1
SL97.11	Stimulant poisoning	1
SM2z.00	Solvents causing toxic effect NOS	1
SLC7000	Sodium morrhuate poisoning	1
SM32200	Sodium hydroxide causing toxic effect	1
SM90100	Sodium cyanide causing toxic effect	1
SM96.00	Soap and detergent causing toxic effect	1
SM96.12	Soap - toxic effect	1
SLF1.00	Smooth muscle relaxant poisoning	1
SL7z.11	Sleeping drug poisoning	1
SLF2.00	Skeletal muscle relaxant poisoning	1
ZX1..00	Self-injurious behaviour	1
ZX...00	Self-harm	1
ZX...11	Self-damage	1
SL7..12	Sedative poisoning	1
SL7z.00	Sedative and hypnotic drug poisoning NOS	1
SL7..00	Sedative and hypnotic drug poisoning	1
SL70500	Secobarbital poisoning	1
SL51100	Salicylic acid salt poisoning	1
SL51z00	Salicylate poisoning NOS	1
SL51.00	Salicylate poisoning	1
SLF7100	Salbutamol poisoning	1
ZX1..12	SIB - Self-injurious behaviour	1
SM02200	Rubbing alcohol causing toxic effect	1
SL06200	Rifampicin poisoning	1
SLF..12	Respiratory system drug poisoning	1
SL14500	Quinine poisoning	1
SLC0300	Quinidine poisoning	1
SL14400	Pyrimethamine poisoning	1
SL53.00	Pyrazole derivative poisoning	1
SL9z.00	Psychotropic agent poisoning NOS	1
SL9..00	Psychotropic agent poisoning	1
SL97.00	Psychostimulant poisoning	1
SL96400	Psilocybin poisoning	1
SL45100	Protamine sulphate poisoning	1
SLF0200	Prostaglandin poisoning	1
SLC0200	Propranolol poisoning	1

Read code	Description	Number of studies
SM70100	Propane causing toxic effect	1
SL91300	Promazine poisoning	1
SL14300	Proguanil poisoning	1
SL22300	Progestogen poisoning	1
SL91200	Prochlorperazine poisoning	1
SLC0100	Procainamide poisoning	1
SL6x000	Primidone poisoning	1
8G61.00	Potential suicide care	1
SM32100	Potassium hydroxide causing toxic effect	1
SM90000	Potassium cyanide causing toxic effect	1
SL25.00	Posterior pituitary hormone poisoning	1
SL94600	Poisoning by temazepam	1
SLD3100	Poisoning by saline and osmotic laxatives	1
SL3..00	Poisoning by primarily systemic agents	1
SLX..00	Poisoning by oth & unspec antipsychotics & neuroleptics	1
SL29.00	Poisoning by mineralocorticoids and their antagonists	1
SLD0200	Poisoning by histamine H2-receptor antagonists	1
SL20300	Poisoning by glucocorticoids and synthetic analogues	1
SL...16	Poisoning by drug and biological substances	1
SL6x100	Poisoning by carbamazepine	1
SLC9.00	Poisoning by calcium-channel blockers	1
SLC6400	Poisoning by angiotensin-converting-enzyme inhibitors	1
SL...00	Poisoning	1
SM82.12	Plants - toxic effect	1
1BDB.00	Plans for deliberate self harm without intent	1
SL16100	Piperazine poisoning	1
SLB0200	Pilocarpine poisoning	1
SM7y000	Phosgene causing toxic effect	1
SM93500	Phosdrin causing toxic effect	1
SL61000	Phenytoin poisoning	1
SL53100	Phenylbutazone poisoning	1
SLB3000	Phenoxybenzamine poisoning	1
SL91z00	Phenothiazine poisoning NOS	1
SL91.00	Phenothiazine poisoning	1
SLD1200	Phenolphthalein poisoning	1
SM30000	Phenol causing toxic effect	1
SL70400	Phenobarbital poisoning	1
SL42200	Phenindione poisoning	1
SL52200	Phenacetin poisoning	1
SLH4.00	Pharmaceutical excipient poisoning	1
SM1z.00	Petroleum product causing toxic effect NOS	1
SM1..00	Petroleum product causing toxic effect	1
SM14.00	Petroleum ether causing toxic effect	1
SM10.00	Petrol unspecified causing toxic effect	1
SL86.00	Peripheral nerve and plexus-blocking anaesthetic poisoning	1
SL70300	Pentobarbitone poisoning	1
SL34000	Penicillinase poisoning	1
SL00z00	Penicillin poisoning NOS	1
SL00.00	Penicillin poisoning	1
SL00300	Penicillin G poisoning	1
E022.00	Pathological drug intoxication	1
E014.00	Pathological alcohol intoxication	1
SM93300	Parathion causing toxic effect	1

Read code	Description	Number of studies
SLB0.00	Parasympathomimetic poisoning	1
SL60000	Paramethadione poisoning	1
SM13.00	Paraffin wax causing toxic effect	1
SL52100	Paracetamol poisoning	1
SL1x300	Para-aminosalicylic acid poisoning	1
SLC5200	Papaverine poisoning	1
SLD4100	Papain poisoning	1
SLD4000	Pancreatin poisoning	1
SL04300	Oxytetracycline poisoning	1
SL21200	Oxymetholone poisoning	1
SL22z00	Ovarian hormone poisoning NOS	1
SL22.00	Ovarian hormone and synthetic substitute poisoning	1
SLC5.00	Other vasodilator poisoning	1
SL95.00	Other tranquilliser poisoning	1
F377.00	Other toxic agent polyneuropathy	1
SL3y.00	Other systemic agent poisoning	1
SM9y.00	Other substance causing toxic effect	1
SL0y.00	Other specific antibiotic poisoning	1
SM2yz00	Other solvents causing toxic effect NOS	1
SM2..00	Other solvents causing toxic effect	1
SM2y.00	Other solvents causing toxic effect	1
SLGx.00	Other skin and mucous membrane drug poisoning	1
SL7y.00	Other sedative and hypnotic poisoning	1
SLFy.00	Other respiratory system drug poisoning	1
SL9y.00	Other psychotropic agent poisoning	1
SM94.00	Other pesticides causing toxic effect NEC	1
SM8y.00	Other noxious substance eaten as food causing toxic effect	1
SM9..00	Other nonmedicinal substances causing toxic effect	1
SL5x.00	Other non-narcotic analgesic poisoning	1
SLF3.00	Other muscle drug poisoning	1
SLE6.00	Other mineral salt poisoning NEC	1
SM5y.00	Other metals causing toxic effect OS	1
SM5yz00	Other metals causing toxic effect NOS	1
SM5..00	Other metals causing toxic effect	1
SLC6.00	Other hypertensive agent poisoning	1
SM71.00	Other hydrocarbon gas causing toxic effect	1
SL2y.00	Other hormone or synthetic derivative poisoning	1
SLDy.00	Other gastrointestinal agent poisoning	1
SM7..00	Other gases, fumes or vapours causing toxic effect	1
SM7y.00	Other gas, fume or vapour causing toxic effect	1
SM7yz00	Other gas, fume and vapour causing toxic effect NOS	1
SLHy.00	Other drug and medicament poisoning OS	1
SLHyz00	Other drug and medicament poisoning NOS	1
SLE4z00	Other diuretic poisoning NOS	1
SLC1100	Other digitalis glycoside poisoning	1
SLAy.00	Other central nervous system stimulant poisoning	1
SLD3.00	Other cathartic poisoning	1
SL93.00	Other antipsychotics/neuroleptics/tranquilliser poisoning	1
SL15.00	Other antiprotozoal drug poisoning	1
SL30x00	Other antihistamine poisoning	1
SL6x.00	Other anticonvulsant poisoning	1
SL1..00	Other anti-infective poisoning	1
SL1y.00	Other anti-infective poisoning	1

Read code	Description	Number of studies
SLH..00	Other and unspecified drug and medicament poisoning	1
SL5yz00	Other analgesic or antipyretic poisoning NOS	1
SL5y.00	Other analgesic and antipyretic poisoning	1
SM0y.00	Other alcohol causing toxic effect	1
SM93z00	Organophosphate and carbamate causing toxic effect NOS	1
SM93.00	Organophosphate and carbamate causing toxic effect	1
SLF1100	Orciprenaline poisoning	1
SL22000	Oral contraceptive poisoning	1
SL50.12	Opiate poisoning	1
SL50z00	Opiate or narcotic poisoning NOS	1
SLA1z00	Opiate antagonist poisoning NOS	1
SLA1.00	Opiate antagonist poisoning	1
SL50.00	Opiate and narcotic poisoning	1
SL03100	Oleandomycin poisoning	1
SL22100	Oestrogen poisoning	1
SL01200	Nystatin poisoning	1
SM8z.00	Noxious substance eaten as food causing toxic effect NOS	1
SM8..00	Noxious substance eaten as food causing toxic effect	1
SLB2100	Noradrenalin poisoning	1
SM...00	Nonmedicinal agent causing toxic effects	1
SL5xz00	Non-narcotic analgesic poisoning NOS	1
SMz..00	Non-medicinal agent causing toxic effect NOS	1
SL82100	Nitrous oxide poisoning	1
SM72.00	Nitrogen oxides causing toxic effect	1
SM72000	Nitrogen dioxide causing toxic effect	1
SL1y100	Nitrofuran derivative poisoning	1
SM31100	Nitric acid causing toxic effect	1
SL94500	Nitrazepam poisoning	1
SLC4100	Nitrate poisoning	1
SM5y300	Nickel compounds causing toxic effect	1
SL50.11	Narcotic poisoning	1
SL54300	Naproxen poisoning	1
SL21100	Nandrolone poisoning	1
SM81.00	Mushrooms causing toxic effect	1
SLF..11	Muscle drug poisoning	1
SL50500	Morphine poisoning	1
1BD2.00	Morbid thoughts	1
SL90200	Monoamine oxidase inhibitor poisoning	1
1BD6.00	Moderate suicide risk	1
SL76.00	Mixed sedative poisoning NEC	1
SL07400	Mitomycin poisoning	1
SL04200	Minocycline poisoning	1
SM01.00	Methyl alcohol causing toxic effect	1
SL80100	Methocarbamol poisoning	1
SL74.00	Methaqualone compound poisoning	1
SM01000	Methanol causing toxic effect	1
SL50200	Methadone poisoning	1
SM5z.00	Metals causing toxic effect NOS	1
SL12300	Mercury compound poisoning	1
SM50.00	Mercury causing toxic effect	1
SL31600	Mercaptopurine poisoning	1
SL95100	Meprobamate poisoning	1
SL50400	Meperidine (pethidine) poisoning	1

Read code	Description	Number of studies
SL54400	Mefenamic acid poisoning	1
SL...13	Medicinal poisoning	1
SL94400	Medazepam poisoning	1
SL96200	Marihuana poisoning	1
SM93200	Malathion causing toxic effect	1
SLD0100	Magnesium trisilicate poisoning	1
SLD3000	Magnesium sulphate poisoning	1
T180.00	MVTA - accid poisoning - exhaust gas of moving motor vehicle	1
SL90211	MAOI - monoamine oxidase inhibitor poisoning	1
SL96100	Lysergide (LSD) poisoning	1
1BD7.00	Low suicide risk	1
SL94300	Lorazepam poisoning	1
SLG2.12	Local detergent poisoning	1
SLG2.11	Local astringent poisoning	1
SLG2.00	Local astringent and detergent poisoning	1
SLG0.00	Local anti-infective and anti-inflammatory poisoning	1
SL8z.00	Local anaesthetic poisoning NOS	1
SLA0000	Lobeline poisoning	1
44W8100	Lithium level high - toxic	1
SM70.00	Liquefied petrol gas causing toxic effect	1
SL85100	Lidocaine poisoning	1
SL27100	Levothyroxine sodium poisoning	1
SL6y200	Levodopa (L-dopa) poisoning	1
SL12200	Lead compound poisoning	1
SM4z.00	Lead compound causing toxic effect NOS	1
SM4..00	Lead and lead compounds causing toxic effect	1
SM41000	Lead acetate causing toxic effect	1
TH02.00	Late effects of accidental poisoning	1
SC...00	Late effects injury/poisoning/toxic effects/external causes	1
SC41.00	Late effect of poison due to nonmedical substance	1
SC40.00	Late effect of poison drug/medicament/biological substance	1
SC41.11	Late effect of poison	1
SCz..00	Late effect injury/poison/toxin effect/external cause NOS	1
SL83000	Ketamine poisoning	1
SM12.00	Kerosene causing toxic effect	1
SLG4.12	Keratoplastic poisoning	1
SL1x200	Isoniazid poisoning	1
SLD1.00	Irritant cathartic poisoning	1
SM5y200	Iron compounds causing toxic effect	1
SL40z00	Iron and iron compound poisoning NOS	1
SL40.00	Iron and iron compound poisoning	1
SL28000	Iodide poisoning	1
SP35000	Intoxication by serum	1
E250.14	Intoxication - alcohol	1
1BDC.00	Intent of deliberate self harm with detailed plans	1
SL23.00	Insulins and antidiabetic poisoning	1
SL23400	Insulin poisoning	1
TN...00	Injury undetermined whether accidentally/purposely inflicted	1
TNz..00	Injury undetermined accidental or purposely inflicted NOS	1
TM21.00	Injury due to legal intervention by poisoning by gas	1
Sz...00	Injury and poisoning NOS	1
S....00	Injury and poisoning	1
TN3y.00	Injury ?accidental, other means of hang/strangle/suffocate	1

Read code	Description	Number of studies
TN9..00	Injury ?accidental, late effects	1
TN3z.00	Injury ?accidental, hanging/strangulation/suffocation NOS	1
TN70.00	Injury ?accidental, fall from residential premises	1
TN71.00	Injury ?accidental, fall from other man-made structure	1
TN72.00	Injury ?accidental, fall from natural site	1
TN7z.00	Injury ?accidental, fall from high place NOS	1
TN7..00	Injury ?accidental, fall from high place	1
TN4..00	Injury ?accidental, drowning	1
TN61.00	Injury ?accidental, by stabbing instrument	1
TN51.00	Injury ?accidental, by shotgun	1
TN82.00	Injury ?accidental, by scald	1
TN8y.00	Injury ?accidental, by other specified means	1
TN8..00	Injury ?accidental, by other means	1
TN54.00	Injury ?accidental, by other firearm	1
TN8z.00	Injury ?accidental, by means NOS	1
TN80100	Injury ?accidental, by lying before moving object	1
TN80.00	Injury ?accidental, by jumping or lying before moving object	1
TN80000	Injury ?accidental, by jumping before moving object	1
TN52.00	Injury ?accidental, by hunting rifle	1
TN50.00	Injury ?accidental, by handgun	1
TN5..00	Injury ?accidental, by firearms and explosives	1
TN5z.00	Injury ?accidental, by firearm or explosive NOS	1
TN83.00	Injury ?accidental, by extremes of cold	1
TN55.00	Injury ?accidental, by explosive	1
TN84.00	Injury ?accidental, by electrocution	1
TN6z.00	Injury ?accidental, by cutting or stabbing instrument NOS	1
TN60.00	Injury ?accidental, by cutting instrument	1
TN6..00	Injury ?accidental, by cutting and stabbing instruments	1
TN85.00	Injury ?accidental, by crashing of motor vehicle	1
TN86.00	Injury ?accidental, by crashing of aircraft	1
TN87.00	Injury ?accidental, by caustic substances, except poisoning	1
TN81.00	Injury ?accidental, by burns or fire	1
SL54100	Indometacin poisoning	1
SL31.12	Immunosuppressive poisoning	1
SL90100	Imipramine poisoning	1
SL54200	Ibuprofen poisoning	1
SL7..11	Hypnotic poisoning	1
SLC6z00	Hypertensive agent poisoning NOS	1
SLB1200	Hyoscine poisoning	1
SL95000	Hydroxyzine poisoning	1
SL13.11	Hydroxyquinoline poisoning	1
SM31000	Hydrochloric acid causing toxic effect	1
SL61.00	Hydantoin derivative poisoning	1
SL47100	Human fibrinogen poisoning	1
SL2z.00	Hormone or synthetic substitute poisoning NOS	1
SL2..00	Hormone and synthetic substitute poisoning	1
1BD5.00	High suicide risk	1
SL50100	Heroin poisoning	1
SL42100	Heparin poisoning	1
SL12z00	Heavy metal anti-infective poisoning NOS	1
SL12.00	Heavy metal anti-infective poisoning	1
SL3y000	Heavy metal agonist poisoning	1
1BD..00	Harmful thoughts	1

Read code	Description	Number of studies
SL81.00	Halothane poisoning	1
SL92000	Haloperidol poisoning	1
SL96z00	Hallucinogen poisoning NOS	1
SL96.00	Hallucinogen poisoning	1
SLG4.00	Hair treatment poisoning	1
14K0.00	H/O: repeated overdose	1
14K..00	H/O: poisoning	1
SL24211	Growth hormone poisoning	1
SL01100	Griseofulvin poisoning	1
SL24100	Gonadotrophin poisoning	1
SL54000	Gold salt poisoning	1
SLDz.00	Gastrointestinal agent poisoning NOS	1
SLD..00	Gastrointestinal agent poisoning	1
SM7z.00	Gases, fumes or vapours causing toxic effect NOS	1
SLC3z00	Ganglion-blocker poisoning NOS	1
SLC3.00	Ganglion-blocker poisoning	1
SM03z00	Fusel oil causing toxic effect NOS	1
SLE4100	Furosemide poisoning	1
SM74.00	Freon causing toxic effect	1
SL41000	Folic acid poisoning	1
SL94200	Flurazepam poisoning	1
SL91100	Fluphenazine poisoning	1
SL31500	Fluorouracil poisoning	1
SL1y000	Flucytosine poisoning	1
SL40100	Ferrous sulphate poisoning	1
SL40000	Ferric salt poisoning	1
SLG..00	Eye, otorhinolaryngological, skin and dental drug poisoning	1
SLG5z00	Eye drug poisoning NOS	1
SLG5.00	Eye drug poisoning NEC	1
SLG..12	Eye drug poisoning	1
SLF5.00	Expectorant poisoning	1
SM00z00	Ethyl alcohol causing toxic effect NOS	1
SM00.00	Ethyl alcohol causing toxic effect	1
SL6y100	Ethopropazine poisoning	1
SL82000	Ether poisoning	1
SM00000	Ethanol causing toxic effect	1
SL1x000	Ethambutol poisoning	1
SLE4000	Ethacrynic acid poisoning	1
SL03000	Erythromycin poisoning	1
SL03.00	Erythromycin and macrolide poisoning	1
SLF0000	Ergot alkaloid poisoning	1
E230200	Episodic acute alcoholic intoxication in alcoholism	1
SL34.00	Enzyme poisoning NEC	1
SLG3.00	Emollients, demulcents and protectant poisoning	1
SLD2.00	Emollient cathartic poisoning	1
SLD6.00	Emetic drug poisoning	1
SLE5.00	Electrolyte agent poisoning	1
SL97200	Ecstasy poisoning	1
SLG6.00	Ear, nose and throat drug poisoning NEC	1
SLz..00	Drug, medicament or biological substance poisoning NOS	1
SL...12	Drug poisoning	1
SL04100	Doxycycline poisoning	1
SLE..11	Diuretic poisoning	1

Read code	Description	Number of studies
SLC4000	Dipyridamole poisoning	1
SL30100	Diphenhydramine poisoning	1
SLD2000	Dioctyl sulphosuccinate poisoning	1
SM02000	Dimethyl carbinol causing toxic effect	1
SL50700	Dihydrocodeine poisoning	1
SLC1000	Digoxin poisoning	1
SLD4.00	Digestant poisoning	1
SL94100	Diazepam poisoning	1
SLHy100	Diagnostic agent NEC, poisoning	1
SL50600	Dextropropoxyphene poisoning	1
SLF4000	Dextromethorphan poisoning	1
SM96.11	Detergent toxic effect	1
SM00100	Denatured alcohol causing toxic effect	1
ZX1..13	Deliberate self-harm	1
SL07300	Daunorubicin poisoning	1
SL07100	Dactinomycin poisoning	1
SM92200	DDT causing toxic effect	1
SL31400	Cytarabine poisoning	1
SL31300	Cyclophosphamide poisoning	1
SLC5000	Cyclandelate poisoning	1
SM90z00	Cyanides causing toxic effect NOS	1
SM90.00	Cyanides and hydrocyanic acid causing toxic effect	1
ZX13100	Cutting own wrists	1
SL42000	Coumarin poisoning	1
SL20000	Cortisone derivative poisoning	1
SL24000	Corticotropin poisoning	1
SM3..00	Corrosives/acids/caustic alkalis causing toxic effect	1
SM3z.00	Corrosive/acid/caustic alkali causing toxic effect NOS	1
SM30z00	Corrosive aromatics causing toxic effect NOS	1
SLC4.00	Coronary vasodilator poisoning	1
SM5y100	Copper salts causing toxic effect	1
E230100	Continuous acute alcoholic intoxication in alcoholism	1
SL22200	Combined oestrogen and progesterone poisoning	1
SLE7100	Colchicine poisoning	1
SL50300	Codeine (methylmorphine) poisoning	1
SL85000	Cocaine poisoning	1
SLC6000	Clonidine poisoning	1
SM56.00	Chromium causing toxic effect	1
SLB0z00	Cholinergic poisoning NOS	1
SLB0.11	Cholinergic poisoning	1
SL91000	Chlorpromazine poisoning	1
SL30000	Chlorphenamine poisoning	1
SL14000	Chloroquine poisoning	1
SM75100	Chloroacetophenone causing toxic effect	1
SM76.00	Chlorine gas causing toxic effect	1
SM92.00	Chlorinated hydrocarbon causing toxic effect	1
SL94000	Chlordiazepoxide poisoning	1
SL02000	Chloramphenicol poisoning	1
SL31200	Chlorambucil poisoning	1
SL71.00	Chloral hydrate poisoning	1
SL13000	Chiniofon poisoning	1
SLH2.11	Chelating agent poisoning	1
SLAz.00	Central nervous system stimulant poisoning NOS	1

Read code	Description	Number of studies
SLA..00	Central nervous system stimulant poisoning	1
SL80z00	Central nervous system muscle-tone depressant poisoning NOS	1
SL80.00	Central nervous system muscle-tone depressant poisoning	1
SL8..00	Central nervous system depressants and anaesthetic poisoning	1
SLH0000	Central appetite depressant poisoning	1
SL05000	Cefalexin poisoning	1
SM32z00	Caustic alkalis causing toxic effect NOS	1
SM32.00	Caustic alkalis causing toxic effect	1
Tz...00	Causes of injury and poisoning NOS	1
T....00	Causes of injury and poisoning	1
T8...11	Cause of overdose - accidental	1
SLC..00	Cardiovascular drug poisoning	1
SLCz.00	Cardiovascular agent poisoning NOS	1
SLC0.00	Cardiac rhythm drug poisoning	1
SLC1.00	Cardiac glycoside poisoning	1
SM21.00	Carbon tetrachloride causing toxic effect	1
SM6..00	Carbon monoxide causing toxic effect	1
SM93000	Carbaryl causing toxic effect	1
SL80000	Carbamate poisoning	1
SLB1400	Caramiphen poisoning	1
SL96000	Cannabis poisoning	1
SL97100	Caffeine poisoning	1
SM55.00	Cadmium causing toxic effect	1
SM70000	Butane causing toxic effect	1
SL70200	Butabarbitone poisoning	1
SL31100	Busulfan poisoning	1
SL07200	Bleomycin poisoning	1
SL...11	Biological substance poisoning	1
SL23100	Biguanide poisoning	1
SLC0400	Beta blocker poisoning	1
SM53.00	Beryllium causing toxic effect	1
SM82.00	Berries and other plants causing toxic effect	1
SM82.11	Berries - toxic effect	1
SLE3000	Benzothiazide poisoning	1
SL94z00	Benzodiazepine poisoning NOS	1
SL94.00	Benzodiazepine poisoning	1
SM20.00	Benzene causing toxic effect	1
SL70z00	Barbiturate poisoning NOS	1
SL70.00	Barbiturate poisoning	1
SL70100	Barbitone poisoning	1
SL31000	Azathioprine poisoning	1
SLB..00	Autonomic nervous system drug poisoning	1
SLB1000	Atropine poisoning	1
1BD8.00	At risk of DSH - deliberate self harm	1
TL22.00	Assault by poisoning by other gases or vapours	1
TL20.00	Assault by poisoning by drugs or medicines	1
TL2z.00	Assault by poisoning NOS	1
TL2..00	Assault by poisoning	1
SL51000	Aspirin poisoning	1
SL11.00	Arsenical anti-infective poisoning	1
SM51.00	Arsenic causing toxic effect	1
SL52z00	Aromatic analgesic poisoning NOS	1
SL52.00	Aromatic analgesic poisoning NEC	1

Read code	Description	Number of studies
SLF4z00	Antitussive poisoning NOS	1
SL28.00	Antithyroid agent poisoning	1
SL54z00	Antirheumatic poisoning NOS	1
SL54.00	Antirheumatic poisoning	1
SL5y200	Antipyretic poisoning, NEC	1
SL5..12	Antipyretic poisoning	1
SL6y.00	Antiparkinsonism drug poisoning	1
SL6yz00	Antiparkinsonian drug poisoning NOS	1
SL31z00	Antineoplastic or immunosuppressive poisoning NOS	1
SL07.00	Antineoplastic antibiotic poisoning	1
SL31.00	Antineoplastic and immunosuppressive poisoning	1
SL14z00	Antimalarial drug poisoning NOS	1
SL14.00	Antimalarial drug poisoning	1
SLC2.00	Antilipaemic and antiarteriosclerotic poisoning	1
SL30.13	Antihistamine poisoning	1
SL01.00	Antifungal antibiotic poisoning	1
SL30.12	Antiemetic poisoning	1
SLD5z00	Antidiarrhoeal poisoning NOS	1
SLD5.00	Antidiarrhoeal poisoning	1
SL6xz00	Anticonvulsant poisoning NOS	1
SL6..11	Anticonvulsant poisoning	1
SL6z.00	Anticonvulsant or antiparkinsonian drug poisoning NOS	1
SL6..00	Anticonvulsant and antiParkinsonian drug poisoning	1
SL42z00	Anticoagulant poisoning NOS	1
SL42.00	Anticoagulant poisoning	1
SL45z00	Anticoagulant agonist poisoning NOS	1
SL45.00	Anticoagulant agonist poisoning	1
SLB0100	Anticholinesterase poisoning	1
SL0z.00	Antibiotic poisoning NOS	1
SL0..00	Antibiotic poisoning	1
SLF7z00	Antiasthmatic poisoning NOS	1
SL30.00	Antiallergic and antiemetic drug poisoning	1
SL1z.00	Anti-infective poisoning NOS	1
SLD0.00	Anti-gastric acid drug poisoning	1
SLF6.00	Anti-common cold drug poisoning	1
SL16.00	Anthelmintic drug poisoning	1
SL24.00	Anterior pituitary hormone poisoning	1
SLD0z00	Antacid drug poisoning NOS	1
SLD0.11	Antacid drug poisoning	1
SL21.12	Androgen poisoning	1
SL21z00	Androgen or anabolic poisoning NOS	1
SL21.00	Androgen and anabolic poisoning	1
SL5z.00	Analgesic, antipyretic or antirheumatic poisoning NOS	1
SL5..00	Analgesic, antipyretic and antirheumatic drug poisoning	1
SL5y100	Analgesic poisoning, NEC	1
SL5..11	Analgesic poisoning	1
SLA0.00	Analeptic poisoning	1
SL8..11	Anaesthetic poisoning	1
SL21.11	Anabolic steroid poisoning	1
SM03000	Amyl alcohol causing toxic effect	1
SL00000	Ampicillin poisoning	1
SL01000	Amphotericin B poisoning	1
SL70000	Amobarbital poisoning	1

Read code	Description	Number of studies
SL90000	Amitriptyline poisoning	1
SLF7000	Aminophylline poisoning	1
SL97000	Amfetamine poisoning	1
SL6y000	Amantadine poisoning	1
SLD0000	Aluminium hydroxide poisoning	1
SLE7000	Allopurinol poisoning	1
SLH3.00	Alcohol deterrent poisoning	1
E230.11	Alcohol dependence with acute alcoholic intoxication	1
SM0z.00	Alcohol causing toxic effect NOS	1
SM0..00	Alcohol causing toxic effect	1
SL4..00	Agents affecting blood constituents, causing poisoning	1
TJF5100	Adverse reaction to ipecacuanha	1
TJF5000	Adverse reaction to acetylcysteine	1
SLC8000	Adrenochrome poisoning	1
SLB2.11	Adrenergic poisoning	1
SL20z00	Adrenal cortico-steroid poisoning NOS	1
SL20.00	Adrenal cortico-steroid poisoning	1
761H300	Administration of activated charcoal	1
E230000	Acute alcoholic intoxication, unspecified, in alcoholism	1
E230z00	Acute alcoholic intoxication in alcoholism NOS	1
E230.00	Acute alcoholic intoxication in alcoholism	1
SM31z00	Acids causing toxic effect NOS	1
SM31.00	Acids causing toxic effect	1
SL32.00	Acidifying agent poisoning	1
SLB0000	Acetylcholine poisoning	1
SM2y000	Acetone causing toxic effect	1
SL23000	Acetohexamide poisoning	1
SLE2000	Acetazolamide poisoning	1
T80yz00	Accidental poisoning-oth analgesic,antipyretic,antirheum NOS	1
T830z00	Accidental poisoning- phenothiazine-based tranquillisers NOS	1
T832z00	Accidental poisoning- benzodiazepine-based tranquilliser NOS	1
T953100	Accidental poisoning from seeds	1
T954.00	Accidental poisoning from other plants	1
T955y00	Accidental poisoning from other fungi	1
T955.00	Accidental poisoning from mushrooms and other fungi	1
T955z00	Accidental poisoning from mushrooms and fungi NOS	1
T955000	Accidental poisoning from mushrooms	1
T95..00	Accidental poisoning from foodstuffs and poisonous plants	1
T953z00	Accidental poisoning from berries or seeds NOS	1
T953.00	Accidental poisoning from berries and seeds	1
T953000	Accidental poisoning from berries	1
T937400	Accidental poisoning by zinc phosphide	1
T916300	Accidental poisoning by white washes	1
T885.00	Accidental poisoning by water,mineral,uric acid metab drugs	1
T937300	Accidental poisoning by warfarin	1
T981z00	Accidental poisoning by utility gas NOS	1
T88z.00	Accidental poisoning by unspecified drugs	1
T83z.00	Accidental poisoning by tranquillisers NOS	1
T83..00	Accidental poisoning by tranquillisers	1
T930500	Accidental poisoning by toxaphene	1
T964700	Accidental poisoning by thallium compounds	1
T937200	Accidental poisoning by thallium	1
T993.00	Accidental poisoning by tear gas	1

Read code	Description	Number of studies
T910.00	Accidental poisoning by synthetic detergents and shampoos	1
T941200	Accidental poisoning by sulphuric acid	1
T991.00	Accidental poisoning by sulphur dioxide	1
T937100	Accidental poisoning by squill and derivatives	1
T92z.00	Accidental poisoning by solvent NOS	1
T96z.00	Accidental poisoning by solid and liquid substances NOS	1
T942000	Accidental poisoning by sodium hydroxide	1
T911.00	Accidental poisoning by soap products	1
T887z00	Accidental poisoning by skin, eye, ENT and dental drug NOS	1
T887000	Accidental poisoning by skin drugs	1
T82z.00	Accidental poisoning by sedatives and hypnotics NOS	1
T903300	Accidental poisoning by secondary propyl alcohol	1
T913000	Accidental poisoning by scouring agents	1
T803z00	Accidental poisoning by salicylates NOS	1
T803.00	Accidental poisoning by salicylates	1
T937z00	Accidental poisoning by rodenticides NOS	1
T937.00	Accidental poisoning by rodenticides	1
T815.00	Accidental poisoning by quinalbarbitone	1
T805.00	Accidental poisoning by pyrazole derivatives	1
T84z.00	Accidental poisoning by psychotropic agents NOS	1
T841400	Accidental poisoning by psilocin	1
T932200	Accidental poisoning by propoxur	1
T980200	Accidental poisoning by propane	1
T830300	Accidental poisoning by promazine	1
T830200	Accidental poisoning by prochlorperazine	1
T881.00	Accidental poisoning by primarily systemic agents	1
T912.00	Accidental poisoning by polishes	1
T965z00	Accidental poisoning by plant foods and fertilisers NOS	1
T965.00	Accidental poisoning by plant foods and fertilisers	1
T965000	Accidental poisoning by plant food	1
T973.00	Accidental poisoning by piped natural gas	1
T938200	Accidental poisoning by phosphine	1
T805100	Accidental poisoning by phenylbutazone	1
T830.00	Accidental poisoning by phenothiazine-based tranquillisers	1
T940011	Accidental poisoning by phenol	1
T814.00	Accidental poisoning by phenobarbitone	1
T804200	Accidental poisoning by phenacetin	1
T920.00	Accidental poisoning by petroleum solvents	1
T923.00	Accidental poisoning by petroleum solids	1
T920200	Accidental poisoning by petroleum naphtha	1
T921.12	Accidental poisoning by petroleum fuels	1
T920z00	Accidental poisoning by petrol solvents NOS	1
T923z00	Accidental poisoning by petrol solids NOS	1
T92..00	Accidental poisoning by petrol products	1
T921.00	Accidental poisoning by petrol fuels and cleaners	1
T921z00	Accidental poisoning by petrol fuel or cleaner NOS	1
T921200	Accidental poisoning by petrol	1
T802100	Accidental poisoning by pethidine	1
T813.00	Accidental poisoning by pentobarbitone	1
T80y000	Accidental poisoning by pentazocine	1
T935500	Accidental poisoning by paraquat	1
T923000	Accidental poisoning by paraffin wax	1
T804100	Accidental poisoning by paracetamol	1

Read code	Description	Number of studies
T916z00	Accidental poisoning by paint or varnish NOS	1
T887300	Accidental poisoning by otorhinolaryngological drugs	1
T98..00	Accidental poisoning by other utility gas + carbon monoxide	1
T981.00	Accidental poisoning by other utility gas	1
T83yz00	Accidental poisoning by other tranquillisers NOS	1
T83y.00	Accidental poisoning by other tranquillisers	1
T924z00	Accidental poisoning by other solvents NOS	1
T924.00	Accidental poisoning by other solvents	1
T96y.00	Accidental poisoning by other solid and liquid substances OS	1
T96..00	Accidental poisoning by other solid and liquid substances	1
T82y.00	Accidental poisoning by other sedatives and hypnotics OS	1
T82..00	Accidental poisoning by other sedatives and hypnotics	1
T84..00	Accidental poisoning by other psychotropic agents	1
T916.00	Accidental poisoning by other paints and varnishes	1
T802z00	Accidental poisoning by other opiates NOS	1
T802.00	Accidental poisoning by other opiates	1
T807.00	Accidental poisoning by other non-narcotic analgesics	1
T9...00	Accidental poisoning by other non-drug substances	1
T964.00	Accidental poisoning by other metals + compounds and fumes	1
T934.00	Accidental poisoning by other insecticides	1
T99y.00	Accidental poisoning by other gases and vapours OS	1
T99yz00	Accidental poisoning by other gases and vapours NOS	1
T99..00	Accidental poisoning by other gases and vapours	1
T95y.00	Accidental poisoning by other foods	1
T901.00	Accidental poisoning by other ethyl alcohol and its products	1
T85..00	Accidental poisoning by other drugs acting on nervous system	1
T88y.00	Accidental poisoning by other drugs OS	1
T88yz00	Accidental poisoning by other drugs NOS	1
T88..00	Accidental poisoning by other drugs	1
T94y.00	Accidental poisoning by other corrosives and caustics	1
T913z00	Accidental poisoning by other cleaning agents NOS	1
T913.00	Accidental poisoning by other cleaning agents	1
T90y.00	Accidental poisoning by other alcohols	1
T966y00	Accidental poisoning by other adhesives	1
T851.00	Accidental poisoning by oth central nervous syst depressants	1
T80y.00	Accidental poisoning by oth analgesics,antipyretic,antirheum	1
T931z00	Accidental poisoning by organophosphorus insecticides NOS	1
T931.00	Accidental poisoning by organophosphorus insecticides	1
T930.00	Accidental poisoning by organochlorine insecticides	1
T936000	Accidental poisoning by organic mercurials	1
T802300	Accidental poisoning by opium	1
T843100	Accidental poisoning by opiate antagonists	1
T887200	Accidental poisoning by ophthalmological drugs	1
T855100	Accidental poisoning by noradrenalin	1
T807z00	Accidental poisoning by non-narcotic analgesics NOS	1
T916200	Accidental poisoning by non-lead paints	1
T990.00	Accidental poisoning by nitrogen oxides	1
T941100	Accidental poisoning by nitric acid	1
T832500	Accidental poisoning by nitrazepam	1
T964600	Accidental poisoning by nickel compounds	1
T806200	Accidental poisoning by naproxen	1
T886.00	Accidental poisoning by muscle + respiratory system drugs	1
T887100	Accidental poisoning by mucous membrane drugs	1

Read code	Description	Number of studies
T982.00	Accidental poisoning by motor vehicle exhaust gas	1
T802200	Accidental poisoning by morphine	1
T840200	Accidental poisoning by monoamine oxidase inhibitors	1
T933.00	Accidental poisoning by mixtures of insecticides	1
T935400	Accidental poisoning by mixtures herbicides+plant food etc	1
T825.00	Accidental poisoning by mixed sedatives NEC	1
T901100	Accidental poisoning by methylated spirit	1
T938100	Accidental poisoning by methyl bromide	1
T902.00	Accidental poisoning by methyl alcohol	1
T823.00	Accidental poisoning by methaqualone compounds	1
T902000	Accidental poisoning by methanol	1
T801.00	Accidental poisoning by methadone	1
T964z00	Accidental poisoning by metals + compounds and fumes NOS	1
T961000	Accidental poisoning by mercury, unspecified	1
T961z00	Accidental poisoning by mercury, NOS	1
T961200	Accidental poisoning by mercury fumes	1
T961.00	Accidental poisoning by mercury and its compounds and fumes	1
T832400	Accidental poisoning by medazepam	1
T964500	Accidental poisoning by manganese and its compounds	1
T931300	Accidental poisoning by malathion	1
T841100	Accidental poisoning by lysergide, LSD	1
T922.00	Accidental poisoning by lubricating oils	1
T832300	Accidental poisoning by lorazepam	1
T852.00	Accidental poisoning by local anaesthetic	1
T852100	Accidental poisoning by lignocaine	1
T960000	Accidental poisoning by lead, unspecified	1
T960z00	Accidental poisoning by lead, NOS	1
T915.00	Accidental poisoning by lead paints	1
T960.00	Accidental poisoning by lead and its compounds and fumes	1
T916000	Accidental poisoning by lacquers	1
T806400	Accidental poisoning by ketoprofen	1
T921300	Accidental poisoning by kerosene	1
T903z00	Accidental poisoning by isopropyl alcohol NOS	1
T903100	Accidental poisoning by isopropanol	1
T964400	Accidental poisoning by iron compounds	1
T934z00	Accidental poisoning by insecticides NOS	1
T806100	Accidental poisoning by indomethacin	1
T840100	Accidental poisoning by imipramine	1
T806300	Accidental poisoning by ibuprofen	1
T854200	Accidental poisoning by hyoscine	1
T941000	Accidental poisoning by hydrochloric acid	1
T850100	Accidental poisoning by hydantoin derivatives	1
T91z.00	Accidental poisoning by household agents NOS	1
T91..00	Accidental poisoning by household agents	1
T880.00	Accidental poisoning by hormones and synthetic substitutes	1
T800.00	Accidental poisoning by heroin	1
T935z00	Accidental poisoning by herbicides NOS	1
T935.00	Accidental poisoning by herbicides	1
T981300	Accidental poisoning by heating gas NOS	1
T831000	Accidental poisoning by haloperidol	1
T851200	Accidental poisoning by halogenated hydrocarbon derivatives	1
T841.00	Accidental poisoning by hallucinogens	1
T841z00	Accidental poisoning by hallucinogen NOS	1

Read code	Description	Number of studies
T806000	Accidental poisoning by gold salts	1
T966z00	Accidental poisoning by glues and adhesives NOS	1
T966.00	Accidental poisoning by glues and adhesives	1
T966000	Accidental poisoning by glues	1
T884.00	Accidental poisoning by gastrointestinal system drugs	1
T470.00	Accidental poisoning by gases or fumes on ship	1
T99z.00	Accidental poisoning by gases and vapours NOS	1
T921100	Accidental poisoning by gas oils	1
T97..00	Accidental poisoning by gas distributed by pipeline	1
T936z00	Accidental poisoning by fungicides NOS	1
T936.00	Accidental poisoning by fungicides	1
T938.00	Accidental poisoning by fumigants	1
T95z.00	Accidental poisoning by foodstuffs and poisonous plants NOS	1
T965100	Accidental poisoning by fertilisers	1
T982000	Accidental poisoning by exhaust gas-stationary farm tractor	1
T982z00	Accidental poisoning by exhaust gas from motor vehicle NOS	1
T982100	Accidental poisoning by exhaust gas from gas engine	1
T901z00	Accidental poisoning by ethyl alcohol NOS	1
T901300	Accidental poisoning by ethanol, NOS	1
T8...00	Accidental poisoning by drugs, medicines and biologicals	1
T882.00	Accidental poisoning by drugs affecting blood constituents	1
T85z.00	Accidental poisoning by drugs acting on nervous system NOS	1
T8z..00	Accidental poisoning by drugs NOS	1
T914.00	Accidental poisoning by disinfectants	1
T935300	Accidental poisoning by diquat	1
T930300	Accidental poisoning by dieldrin	1
T832100	Accidental poisoning by diazepam	1
T800.11	Accidental poisoning by diamorphine	1
T887400	Accidental poisoning by dental drugs	1
T901000	Accidental poisoning by denatured alcohol	1
T938000	Accidental poisoning by cyanides	1
T967.00	Accidental poisoning by cosmetics	1
T94z.00	Accidental poisoning by corrosives and caustics NOS	1
T94..00	Accidental poisoning by corrosives and caustics NEC	1
T940.00	Accidental poisoning by corrosive aromatics	1
T964300	Accidental poisoning by copper salts	1
T981400	Accidental poisoning by cooking gas NOS	1
T802000	Accidental poisoning by codeine	1
T852000	Accidental poisoning by cocaine	1
T971.00	Accidental poisoning by coal gas NOS	1
T853.00	Accidental poisoning by cholinergics	1
T830000	Accidental poisoning by chlorpromazine	1
T99y000	Accidental poisoning by chlorine	1
T832000	Accidental poisoning by chlordiazepoxide	1
T930100	Accidental poisoning by chlordane	1
T935200	Accidental poisoning by chlorates	1
T820.00	Accidental poisoning by chloral hydrate	1
T88y000	Accidental poisoning by central appetite depressants	1
T942z00	Accidental poisoning by caustic alkalis NOS	1
T942.00	Accidental poisoning by caustic alkalis	1
T883.00	Accidental poisoning by cardiovascular system drugs	1
T983.00	Accidental poisoning by carbon monoxide-other domestic fuel	1
T970.00	Accidental poisoning by carbon monoxide from piped gas	1

Read code	Description	Number of studies
T98y.00	Accidental poisoning by carbon monoxide from other sources	1
T98yz00	Accidental poisoning by carbon monoxide from oth source NOS	1
T98z.00	Accidental poisoning by carbon monoxide NOS	1
T983z00	Accidental poisoning by carbon monoxide - domestic fuel NOS	1
T940000	Accidental poisoning by carbolic acid	1
T932100	Accidental poisoning by carbaryl	1
T932.00	Accidental poisoning by carbamates	1
T841000	Accidental poisoning by cannabis derivatives	1
T842100	Accidental poisoning by caffeine	1
T964200	Accidental poisoning by cadmium and its compounds	1
T980100	Accidental poisoning by butane	1
T993000	Accidental poisoning by bromobenzyl cyanide	1
T822.00	Accidental poisoning by bromine compounds	1
T822000	Accidental poisoning by bromides	1
T964100	Accidental poisoning by brass fumes	1
T964000	Accidental poisoning by beryllium and its compounds	1
T832.00	Accidental poisoning by benzodiazepine-based tranquillisers	1
T930000	Accidental poisoning by benzene hexachlorine	1
T924000	Accidental poisoning by benzene	1
T81z.00	Accidental poisoning by barbiturates NOS	1
T81..00	Accidental poisoning by barbiturates	1
T811.00	Accidental poisoning by barbitone	1
T854000	Accidental poisoning by atropine	1
T803000	Accidental poisoning by aspirin	1
T963000	Accidental poisoning by arsenic, unspecified	1
T963100	Accidental poisoning by arsenic compounds	1
T963.00	Accidental poisoning by arsenic and its compounds and fumes	1
T804.00	Accidental poisoning by aromatic analgesics NEC	1
T806z00	Accidental poisoning by antirheumatics NOS	1
T806.00	Accidental poisoning by antirheumatics	1
T840z00	Accidental poisoning by antidepressants NOS	1
T840.00	Accidental poisoning by antidepressants	1
T850z00	Accidental poisoning by anticonvulsant/anti-parkin drug NOS	1
T850.00	Accidental poisoning by anticonvulsant + anti-parkinson drug	1
T850.11	Accidental poisoning by anticonvulsant	1
T854.00	Accidental poisoning by anticholinergics	1
T86..00	Accidental poisoning by antibiotics	1
T850.12	Accidental poisoning by anti-parkinsonism drug	1
T87..00	Accidental poisoning by anti-infectives	1
T80..00	Accidental poisoning by analgesics,antipyretic,antirheumatic	1
T80z.00	Accidental poisoning by analgesics,antipyretic,antirheum NOS	1
T842000	Accidental poisoning by amphetamine	1
T840000	Accidental poisoning by amitriptyline	1
T900.00	Accidental poisoning by alcoholic beverages	1
T90..00	Accidental poisoning by alcohol, NEC	1
T90z.00	Accidental poisoning by alcohol NOS	1
T93..00	Accidental poisoning by agricultural chemical preparations	1
T855.00	Accidental poisoning by adrenergics	1
T855000	Accidental poisoning by adrenalin	1
T941z00	Accidental poisoning by acids NOS	1
T941.00	Accidental poisoning by acids	1
T981000	Accidental poisoning by acetylene	1
T930200	Accidental poisoning by DDT	1

Read code	Description	Number of studies
T983300	Accidental poisoning by CO- kerosene in domestic stove/fire	1
T983100	Accidental poisoning by CO- coke in domestic stove/fireplace	1
T983000	Accidental poisoning by CO- coal in domestic stove/fireplace	1
T98y100	Accidental poisoning by CO - kiln vapour	1
T98y200	Accidental poisoning by CO - fuels in industrial use	1
T98y000	Accidental poisoning by CO - blast furnace gas	1
T93z.00	Accidental poisoning agricultural chemical preparations NOS	1
T9z..00	Accidental poisoning NOS	1
T77z.00	Accident/poisoning occurred in residential institution NOS	1
T85y.00	Accid. poisoning by other drugs acting on nervous system OS	1
T470500	Accid poison gas/fume on ship - swimmer injured	1
SL24011	ACTH - adrenocorticotropic hormone poisoning	1

Table 13. ICD codes used in the studies of fatal self-harm.

Study	ICD version	List of codes
Carr, 2017	ICD-10	V01-Y98
Coupland, 2015	ICD-10	not provided
Doyle, 2016	ICD-10	X60-X84, Y10-34 (excluding Y33.9), Y87.0 and Y87.2
Lalmohamed, 2012	ICD-10	V01-Y99
Meier, 2004	ICD-10	not provided
Schuerch, 2016	ICD-10	X60-X84, Y10-Y34
Thomas, 2013	ICD-10	X60-X84, Y10-34 (excluding Y33.9)
Webb, 2012	ICD-10	X60-X84, Y10-34 (excluding Y33.9)
Windfuhr, 2016	ICD-10	X60-X84, Y10-34 (excluding Y33.9), Y87.0 and Y87.2

ICD - International Classification of Diseases
